# Design, Synthesis, and Antitumor Biological Evaluation of Galaxamide and Its Analogs

**DOI:** 10.3390/molecules30112362

**Published:** 2025-05-29

**Authors:** Yanyan Guo, Huixia Fan, Zhiqiang Luo, Jian Yang, Guodu Liu

**Affiliations:** 1 Inner Mongolia Key Laboratory of Synthesis and Application of Organic Functional Molecules, College of Chemistry and Chemical Engineering, Inner Mongolia University (South Campus), 24 Zhaojun Road, Hohhot 010030, China; 15754931286@163.com; 2 State Key Laboratory for Quality Ensurance and Sustainable Use of Dao-Di Herbs, National Resource Center for Chinese Materia Medica, China Academy of Chinese Medical Sciences, Beijing 100700, China; fhx165679@163.com; 3 Research Center for Quality Evaluation of Dao-Di Herbs, Ganjiang New District, Nanchang 330000, China; 4 Inner Mongolia Academy of Science and Technology, 2 Shandan Street, Hohhot 010010, China

**Keywords:** galaxamide, cyclopeptide, liquid-phase peptide synthesis, marine natural product, antitumor agent

## Abstract

Galaxamide, an *N*-methylated cyclo-pentapeptide containing five *D*-leucines isolated from *Galaxaura filamentosa*, has shown significant antitumor activity. This unique cyclo-pentapeptide offered a fresh skeleton for structural modifications. Herein, galaxamide and its 23 analogs (**Gala01**~**Gala24**) were designed and synthesized by substituting *D*-leucine with various proteinogenic amino acids or altering the amino acid configuration using the “3 + 2” strategy, and the in vitro antitumor activity of these cyclopeptides was studied utilizing the CCK-8 assay against two human tumor cell lines (A549 and K562) and one human normal cell line (293T). The total yields of galaxamide and its analogs reached 9.7% and 9.1–16.0%, respectively. CCK-8 assays demonstrated that these compounds showed broad-spectrum antitumor activity, with **Gala04** exhibiting outstanding activity against K562 cells (IC_50_ = 4.2 µM). The anticancer efficacy of galaxamide analogs against tumor cell lines was significantly influenced by the quantity of D-leucines and the *D*-leucine position.

## 1. Introduction

The range of potency, specificity, and safety of the peptide chain strengthened by cyclization has shown the essential properties of cyclic peptides since insulin first began to be utilized in the clinic about a century ago [1]. Two thirds of the 460 peptides that have received FDA and EMA approval are in the cyclic form and are significant players in the contemporary pharmaceutical market [2]. The peptide chain becomes more conformationally stable due to the constraint imposed by cyclization, increasing the target protein binding affinity and lowering nonspecific binding because there are fewer possible configurations [3]. Reduced conformational flexibility reduces the possibility of molecules fitting into the protease catalytic site and improves proteome resistance [4]. By creating a bigger contact surface for interfering protein–protein interactions, cyclization also improves the effectiveness of peptide chains [5]. All things considered, peptide chain cyclization results in cyclic peptides that are fundamentally distinct from linear peptides [6].

It has been discovered that *N*-methylation significantly improves therapeutic efficiency and receptor selectivity while providing bioactive peptides with metabolic stability [7]. By decreasing the quantity of hydrogen-bond donors and inhibiting the creation of both intramolecular and intermolecular hydrogen bonds, *N*-methylation increases the hydrophobicity of peptides [8]. Furthermore, *N*-methylation promotes the creation of cis-peptide bonds; this not only impacts the structural properties of the antecedent and subsequent residue but also affects cyclic peptides’ backbone conformation [9]. Multiple *N*-methylations alter the structure of cyclic peptides in a regular pattern. The steric interactions between the inserted *N*-methyl group and the carbonyl oxygen and/or the α-substitution of the constituent amino acids determine the conformation of cyclic peptides, rather than the side chain’s function. This makes it possible to create peptides with stiff structures that, through altering their amino acid composition, have distinct functions [10].

**Figure 1:**
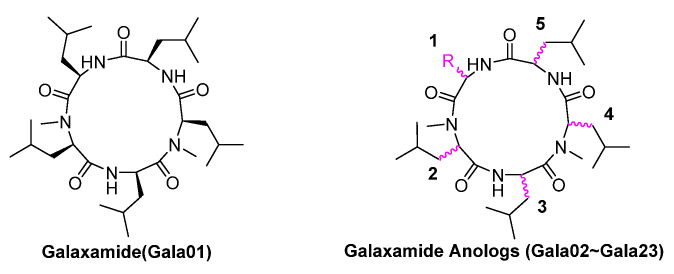
Structure of galaxamide and galaxamide analogs.

Cancer is an atypical proliferation of cells and tissues that impacts individuals across all age groups, and the dangers connected with it may escalate as patients age. More than 277 kinds of cancer have been found and diagnosed to date, with the most common ones being bladder, prostate, lung, colon, rectum, bronchus, and lung cancers [11]. The vast majority of anticancer medications on the market are natural products, or mimics of natural products. In 2008, a unique cytotoxic cyclic pentapeptide known as galaxamide was discovered from a marine algae, *Galaxaura filamentosa*, that was collected from Xisha Island in the South China Sea [12]. According to the comparison of the structure and NMR data of galaxamide in [12], galaxamide is made up of five *D*-leucines, two of which are *N*-methylated, which is defined as cyclo-(*D*-Leu-*D*-*N*-Me-Leu-*D*-Leu-*D*-Leu -*D*-*N*-Me-Leu-*D*-Leu) (Figure 1). *N*-methylated amino acid was found to be an essential element providing cyclopeptides their anticancer potential [13]. Galaxamide demonstrated remarkable half-maximal inhibitory concentration (IC_50_) values of 4.26 and 4.63 µg/mL, respectively, against human renal cell carcinoma GRC-1 and human hepatocellular carcinoma Hep G2 cell lines, also demonstrating substantial in vitro antiproliferative properties. Furthermore, our data demonstrated that galaxamide could induce cell apoptosis through a mitochondria-mediated apoptosis pathway [14]. By substituting *D*-leucine with other amino acids or altering the *D*-leucine configuration, the galaxamide analogs were created [15]. A better understanding of the structure–activity relationship for galaxamide is urgently required to investigate its promising anticancer potential, as well for the structure-based design of new anticancer drugs.

Here, galaxamide and its analogs were designed and synthesized by substituting *D*-leucine with various natural amino acids and altering the amino acid configuration at five positions (Figure 1, **Gala01**~**Gala24**). The in vitro antitumor activities were investigated utilizing the CCK-8 assay against two human tumor cell lines, A549 and K562, and one human normal cell line (293T). **Gala04** was found to have the most superior antitumor activity against K562 (IC_50_ = 4.188 µM), compared with the activity of the normal cell line 293T (IC_50_ = 16.3 µM), and is worthy of further development.

## 2. Results and Discussion

### 2.1. Chemistry

**Scheme 1:**
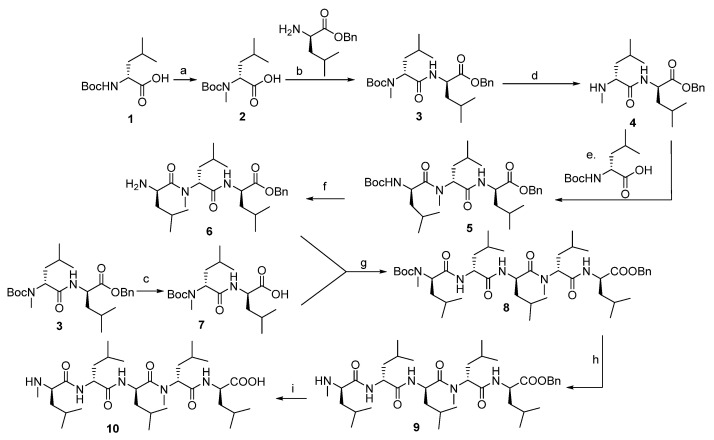
Synthesis of the precursor of galaxamide pentapeptide **10** ^a^. Reagents and conditions: a, NaH, CH_3_I, THF, 0 °C, 12 h, 90%; b, EDCI, HOBt, DIPEA, DCM, 25 °C, 3 h, 85%; c, H_2_, 10% Pd/C, EtOH, 25 °C, 6 h, 90%; d, TFA, DCM, 0 °C, 3 h, 90%; e, EDCI, HOBt, DIPEA, DCM, 25 °C, 3 h, 85%; f, TFA, DCM, 0 °C, 3 h, 90%; g, EDCI, HOBt, DIPEA, DCM, 25 °C, 3 h, 85%; h, TFA, DCM 0 °C, 3 h, 90%; i, H_2_, 10% Pd/C, EtOH, 25 °C, 6 h, 90%.

The “3 + 2” synthesis strategy divides the synthesis of cyclic pentapeptide into two fragments. One is a fragment containing three amino acids, and the other is a fragment containing two amino acids. Then, these two fragments are connected by chemical methods, and finally, a pentapeptide chain is formed. This method can improve the synthesis efficiency and the purity of the product. First of all, the traditional “3 + 2” strategy was used in the current effort to design and synthesize galaxamide. As showed in Scheme 1, readily accessible *N*-Boc-*D*-Leu-OH **1** was the starting material of our synthesis. It reacted with CH_3_I/NaH in THF to produce *N*-Boc-Me-*D*-Leu-OH **2** (90% yield). Dipeptide **3** was obtained via the condensation reaction of *N*-Boc-Me-*D*-Leu-OH and *D*-Leu-OBn·TosOH (85% yield). The condensation reagent utilized in this reaction was 1-(3-dimethylaminopropyl)-3-ethylcarbodiimide hydrochloride (EDCI), 1-hydroxybenzotriazole (HOBt), and *N,N*-diisopropylethylamine (DIPEA). The *N*-terminal Boc group was removed using trifluoroacetic acid (TFA) from dipeptide **3** to obtain the deprotected dipeptide **4** (90% yield). Tripeptide **5** was then synthesized by coupling of deprotected dipeptide **4** with *N*-Boc-D-Leu-OH **1** (85% yield). Subsequent removal of the *N*-terminal Boc group of **5** with TFA yielded the deprotected tripeptide **6** (90% yield). Deprotective dipeptide **7** was achieved by the removal of the C-terminal Bn group from dipeptide **3** through hydrogenation with Pd/C (90% yield). Then, *N*-terminal-Boc-protected dipeptide **7** and C-terminal-Bn-protected tripeptide **6** were condensation-reacted to produce the linear pentapeptide **8** (85% yield). The dried, unpurified free amine/free acid linear pentapeptide **10** was obtained from pentapeptide **8** (81% yield) by hydrogen deprotection of the Bn group with Pd/C and TFA removal of the Boc group, respectively. Scheme 1 displays the detailed general chemical synthesis of the precursor of galaxamide pentapeptide **10**.

A crucial step for the synthesis of galaxamide is macrolactamization. A preliminary analysis of peptide coupling agents for the production of galaxamide was carried out by the macrolactamization of **10** (Table 1). Both (2-(1H-benzotriazol-1-yl)-1,1,3,3-tetramethyluronium hexafluorop phosphat (HATU) and propanephosphonic acid anhydride (T_3_P) demonstrated poor conversion of the reaction (entries 1 and 2). It is interesting to note that using diphenylphosphoryl azide (DPPA) and pentafluorophenyl diphenylphosphinate (FDPP) yielded an incredibly clean profile for macrocycle galaxamide, leading to macrolactamization product in moderate yield (entry 4 and 5). Benzotriazol-1-yl-oxytripyrrolidino-phosphonium hexafluorophosphate (PyBOP) (entry 3) primarily yielded the expected product galaxamide. Additional reaction dilution reduced the generation of dimers. Galaxamide was produced in 50% yield when the reaction was carried out at a lower concentration (0.005 vs. 0.1 M) treatment with PyBOP and DMAP in CH_3_CN at room temperature. Moreover, a high dilution environment was simulated by slowly dosing 10 over 4 h, which prevented the production of dimers and allowed for the use of a more realistic reaction concentration of 0.1 M. Finally, silica gel column flash chromatography was used to separate and purify the final macrolactamization natural product galaxamide (**Gala01**).

With the optimized synthetic process of galaxamide (**Gala01**), 23 galaxamide analogs (**Gala02**~**Gala24**) were designed and synthesized (Figure 1). On the one hand, compared to natural galaxamide, galaxamide analogs containing *L*-leucine might show greater suppression of cancer cells. A greater quantity of *L*-leucine incorporated in galaxamide would lead to improved anticancer activity. On the other hand, introduction of other natural amino acids to galaxamide by substituting for one of the leucine might have good effects on their antitumor activities. Using the similar “3 + 2” strategy and following the same procedures, various accessible natural amino acids were replaced in the different positions of galaxamide.

**Figure 2:**
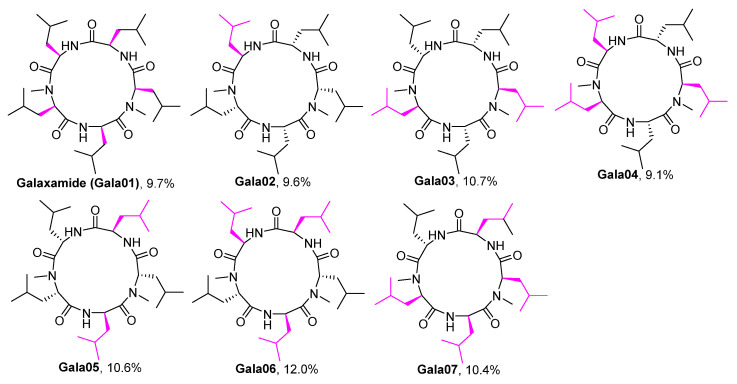
The first generation of galaxamide derivatives ^a^.^a^ Total yield of all steps.

**Figure 3:**
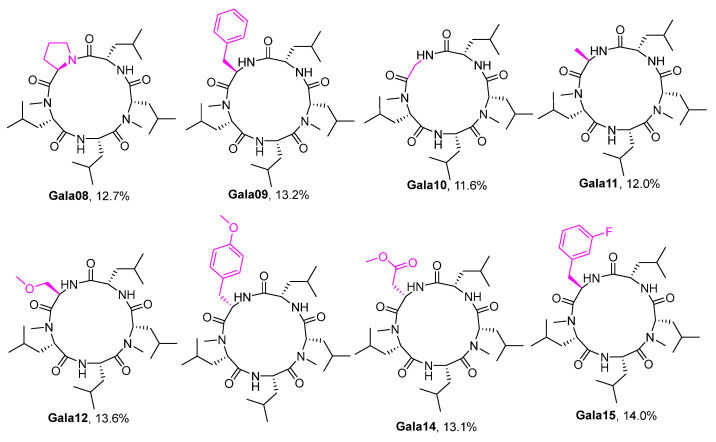

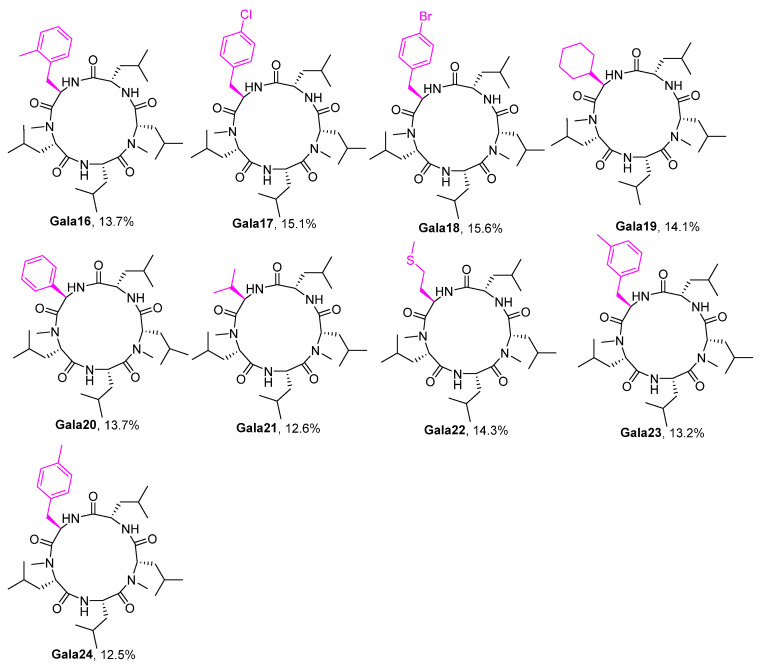
The second generation of galaxamide derivatives ^a^.^a^ Total yield of all steps.

Two generations of galaxamide derivatives have been designed and synthesized, where the first generation primarily involved changing *D*-leucine to *L*-leucine and varying the *D*-leucine position and quantity (**Gala02**~**Gala08**, Figure 2), and the second generation substituted one of the leucine with other natural amino acids (**Gala09**~**Gala26**, Figure 3), including *D*-Proline, *D*-Phenylalanine, Glycine, *D*-Alanine, *O*-Methyl-*D*-Serine, *O*-Methyl-*D*-tyrosine, Methyl ester-*D*-Aspartic acid, 3-Fluoro-*D*-Phenylalanine, 2-Methyl-*D*-Phenylalanine, 4-Chloro-*D*-Phenylalanine, 4-Bromo-*D*-Phenylalanine, *D*-Cyclohexyl-Glycine, *D*-Phenyl-Glycine, *D*-Valine, *D*-Methionine, 3-Methyl-*D*-Phenylalanine, and 4-Methyl-*D*-Phenylalanine. All these analogs were synthesized with good total yields (9.1–16.0%), which is shown in detail in Figure 2 and Figure 3.

### 2.2. In Vitro Activity of Galaxamide and Its Analogs

Since the antitumor activity of natural galaxamide is good, we are interested in the antitumor potential of the obtained new analogs. To examine the anticancer activities of galaxamide and its analogs, two different human cancer cell lines, human non-small-cell lung cancer cells (A549) and human chronic myelogenous leukemia (K562), and one normal human cell line, human immortal embryonic kidney cells (293T), were selected for the in vitro cell models. IC_50_ values of galaxamide and its analogs on the three cell lines are shown in Table 2. In order to have a reference of the potency of the activity of the compounds studied, the results of the activity tests against (−)-β-elemene with recognized activity and/or used as a drug against the cell lines studied are usually added in Table 2. To our surprise, the results showed that multiple compounds had better anticancer activity than (−)-β-elemene, which was consistent with our original assumptions and proved the practicality of our developed method [16]. The synthetic galaxamide (**Gala01**) was also tested as the standard. The results showed that, for cancer cell A549, IC_50_ values of **Gala01** (standard)**, Gala04**, **Gala08, Gala11**, **Gala12**, **Gala18**, and **Gala22** were all less than 20 µM, indicating that these cyclopeptides have superior anticancer activity than other analogs. For cancer cell K562, IC_50_ values of **Gala02**, **Gala04**, **Gala05**, **Gala08, Gala15**, **Gala22**, **Gala23**, and **Gala24** were from 4.2 µM to 16.8 µM, implying that these analogs have better anticancer activity than other ones. For the normal cell 293T, most of the analogs showed no inhibiting effect as their IC_50_ was much higher. Furthermore, among these compounds, **Gala04** was found to have the most superior activity against K562 (IC_50_ = 4.2 µM), compared with the activity of the normal cell line 293T (IC_50_ = 16.3 µM). It was also found that **Gala04** has the best selectivity and highest safety and is thus worthy of further development for anticancer drugs for K562.

Surprisingly, the anticancer efficacy of galaxamide analogs against tumor cell lines was significantly influenced by the quantity of D-leucines. With IC_50_ values of 14.0 µM, **Gala01** demonstrated noteworthy anticancer activity against the A549 cell line. Meanwhile, the IC_50_ values of **Gala03** to **Gala07** were >20.0, 19.0, >20.0, >20.0, and >20.0 μM, respectively. Compared to the other analogs, **Gala01** was more potent, indicating that compounds containing five D-amino acids have superior activity to analogs containing fewer D-amino acids than **Gala03**~**Gala07**. Furthermore, the anticancer efficacy of the analogs was significantly impacted by changing the D-leucine position. The best analog for preventing the proliferation of all cancer cells is **Gala04**, which has the D-leucine at position 1, 2, and 4. Its IC_50_ values are almost half that of **Gala01**, with 4.2 μM against K562 cell line. **Gala02**, which has a D-amino acid at position 1, exhibited a strong inhibitory impact against K562, with an IC_50_ value of 8.6 μM, in addition to **Gala04**. The reason behind this could be that a more stable and confined cyclic structure of galaxamide was attained when the D-amino acid was positioned at positions 1, 2, and 4. **Gala04** showed the most stable cyclic configuration with the lowest energy and the most active amino acid residues for targeting cancer cells. **Gala05** showed lower antitumor efficacy with D-amino acid in positions 3 and 5. Specifically, **Gala04** showed twice as much anticancer potential as **Gala05** on the K562 cell line with IC_50_ values of 10.2 μM, respectively. **Gala06** (D-amino acid at positions 1, 3, and 5) showed a slightly altered antitumor efficacy compared to **Gala04** against K562 with IC_50_ values of 28.8 μM. Since **Gala05** and **Gala06** showed less anticancer impact on the examined cancer cells, the restricted structure of the galaxamide analog might be partially broken when the D-amino acid was positioned at positions 3 and 5. **Gala18** contains a bromine (-Br) group at the p-position of the benzyl ring and showed a value of 15.5 μM against the A549 cell line. Overall, the compounds with good anticancer activity mostly contain the bromine (-Br) group at the p-position of the benzyl ring, which is one spot worth paying attention to.

## 3. Materials and Methods

Commercially available reagents (TCI, Zwijndrecht, Belgium; InnoChem, LAAJOO, Beijing, China; Sigma-Aldrich, Steinheim, Germany) were used without further purification. If necessary, the reaction was carried out under nitrogen or argon atmosphere and dry solvents. All air-sensitive procedures were conducted in a nitrogen-filled glovebox or by Schlenk techniques under nitrogen. Column chromatography was carried out on 200–300 mesh silica gel using pressurized air. ^1^H NMR spectra and ^13^C NMR spectra were measured on Bruker DRX 500 and Bruker DRX 600 instruments (Bruker, Billerica, MA, USA). Chemical shifts δ are reported in parts per million (ppm) and relative to the undeuterated solvent residual signals of CDCl_3_ (^1^H: δ = 7.26 ppm; ^13^C: δ = 7.16 ppm) and DMSO-*d*_6_ (^1^H: δ = 2.5 ppm; ^13^C: δ = 39.5 ppm). Coupling constants J are given in Hertz (Hz) and refer to H–H-coupling. Multiplicities are designated as follows: s = singlet, d = doublet, t = triplet, q = quartet, and dd = doublet of doublet. When the multiplicity could not be identified, the chemical shift range of the signal was given (m = multiplet). HPLC analyses and purifications were performed on a Thermo Fisher LC system (Thermo Fisher Scientific, Waltham, MA, USA) with UV/Vis detector using CHIRALCEL columns. HRMS was performed on a XEVO G2-XS QTOF instrument (Waters Corporation, Milford, MA, USA).

### 3.1. Chemical Synthesis

#### 3.1.1. Synthesis of **2**

NaH (60% dispersion in mineral oil, 137.5 mmol, 3.5 equiv) was added in parts at 0 °C to a solution of *L(D)*-Leu (38.9 mmol, 1.0 equiv) in THF (0.5 M). Subsequently, CH_3_I (177.3 mmol, 4.5 equiv) was added after 30 min of stirring at 0 °C. Before being quenched with 50 mL of H_2_O, the reaction was agitated for 30 min at 0 °C and for an additional 4 h at room temperature. EtOAc was used to extract the mixture. After that, the aqueous solution was acidified to pH 5 with 1N HCl solution and extracted using EtOAc. Following a wash with brine, the mixed EtOAc extracts were dried over Na_2_SO_4_, filtered, and evaporated to produce **2** as a colorless oil [17]. The extra peaks are generated because the nitrogen atom in these compounds is bonded to three different groups; C–N bond rotation leads to the emergence of rotamers and consequent splitting in the NMR spectra, as referenced in the literature [18].

*N-Boc-Me-L-Leu-OH* **2a**. The **1a** was synthesized according to the method of **2** from L-Leu (9.0 g, 38.9 mmol, 1.0 equiv), CH_3_I (25 g, 177.3 mmol, 4.5 equiv), and NaH (60% dispersion in mineral oil, 5.5 g, 137.5 mmol, 3.5 equiv). Following a wash with brine, the mixed EtOAc extracts were dried over Na_2_SO_4_, filtered, and evaporated to produce **2a** (9.0 g, 36.7 mmol, 94% yield) as a white solid. [α]^25^_D_ = −36.2 (*c* = 1.0, CHCl_3_). m.p. 55–56 °C. ^1^H NMR (600 MHz, CDCl_3_) δ 4.83 (t, *J* = 7.9 Hz, 1H, major), 4.71–4.52 (m, 1H, minor), 2.85 (s, 3H, major), 2.82 (s, 3H, minor), 1.75 (t, *J* = 7.8 Hz, 2H), 1.66–1.56 (m, 1H), 1.49 (s, 9H, major), 1.48 (s, 9H, minor), 0.97 (dd, *J* = 10.8, 6.6 Hz, 6H). ^13^C NMR (151 MHz, CDCl_3_) δ 178.0, 156.6 (major), 155.7 (minor), 57.0 (major), 56.2 (minor), 37.8 (major), 37.3 (minor), 30.7 (major), 30.6 (minor), 28.3, 24.9 (major), 24.7 (minor), 23.2 (major), 23.1 (minor), 21.3 (major), 21.1 (minor). HRMS (ESI^+^) calcd. for C_12_H_23_NO_4_ [M + Na]^+^: 268.1520; found: 268.1527.
*N-Boc-Me-D-Leu-OH* **2b**. The **1b** was synthesized according to the method of **2** from D-Leu (9.0 g, 38.9 mmol, 1.0 equiv), CH_3_I (25 g, 177.3 mmol, 4.5 equiv), and NaH (60% dispersion in mineral oil, 5.5 g, 137.5 mmol, 3.5 equiv). Following a wash with brine, the mixed EtOAc extracts were dried over Na_2_SO_4_, filtered, and evaporated to produce **2b** (8.8 g, 35.9 mmol, 91% yield) as a white solid. [α]^25^_D_ = +36.2 (*c* = 1.0, CHCl_3_). m.p. 55–56 °C. ^1^H NMR (600 MHz, Chloroform-*d*) δ 4.87 (t, *J* = 7.7 Hz, 1H, major), 4.63 (dd, *J* = 11.2, 5.1 Hz, 1H, minor), 2.84 (s, 3H, major), 2.81 (s, 3H, minor), 1.79–1.64 (m, 2H), 1.64–1.53 (m, 1H), 1.48 (s, 9H, major), 1.47 (s, minor), 0.96 (dd, *J* = 11.4, 6.3 Hz, 6H). ^13^C NMR (151 MHz, Chloroform-*d*) δ 177.9 (major), 177.7 (minor), 156.5 (major), 155.7 (minor), 80.6 (major), 80.3 (minor), 57.1 (major), 56.1 (minor), 37.8 (major), 37.3 (minor), 30.6, 28.3 (major), 28.3 (minor), 24.9 (major), 24.6 (minor), 23.2 (major), 23.1 (minor), 21.2 (major), 21.1 (minor). HRMS (ESI^+^) calcd. for C_12_H_23_NO_4_ [M + Na]^+^: 268.1520; found: 268.1527.

#### 3.1.2. Synthesis of Dipeptide **3**

**2** (40.8 mmol, 1.0 equiv) and *L(D)*-Leu-OBn·TosOH (44.9 mmol, 1.1 equiv) and HOBT (44.9 mmol, 1.1 equiv) were added to dry DCM (0.5 M) at 0 °C. At this temperature, the EDCI (44.9 mmol, 1.1 equiv) was slowly added. The reaction mixture was warmed to 23 °C and stirred for 12 h. After most of the DCM was removed in vacuo, the reaction solution was washed with two 800 mL portions of 0.5 N hydrochloric acid and two portions of saturated Na_2_CO_3_ solution. Then, the layers were separated, and the combined organic layer was dried over Na_2_SO_4_. The solvent was removed in vacuo to obtain dipeptide. The crude product was purified by silica gel chromatography (hexane/EtOAc = 98:2, *v*/*v*)) to obtain 3 as a white solid. The extra peaks are generated because the nitrogen atom in these compounds is bonded to three different groups, and C–N bond rotation leads to the emergence of rotamers and consequent splitting in the NMR spectra, as referenced in the literature [18].

*N-Boc-Me-L-Leu-L-Leu-OBn* **3a**. The **3a** was synthesized according to the method of **3** from **2a** (10.0 g, 40.8 mmol, 1.0 equiv) and *L*-Leu-OBn·TosOH (9.9 g, 44.9 mmol, 1.1 equiv), HOBT (6.0 g, 44.9 mmol, 1.1 equiv), and EDCI (8.5 g, 44.9 mmol, 1.1 equiv). The crude product was purified by silica gel chromatography (hexane/EtOAc = 90:10, *v*/*v*) to obtain **3a** (15.0 g, 33.4 mmol, 82% yield) as a white solid. [α]^25^_D_ = −272.8 (*c* = 1.0, CHCl_3_). m.p. 80–81 °C. ^1^H NMR (500 MHz, DMSO-*d*_6_) δ 8.23 (d, *J* = 7.8 Hz, 1H), 7.41–7.29 (m, 5H), 5.14–5.01 (m, 2H), 4.64 (s, 1H, major), 4.47 (s, 1H, minor), 4.43–4.27 (m, 1H), 2.70 (s, 3H), 1.69–1.40 (m, 6H), 1.39 (s, 9H, major), 1.36 (s, 9H, minor)., 0.95–0.70 (m, 12H). ^13^C NMR (151 MHz, DMSO-*d*_6_) δ 172.6, 171.9, 136.4, 128.8, 128.4, 128.3, 79.3, 66.4, 50.8, 30.2, 28.5, 24.9, 24.7, 23.2, 21.6. HRMS (ESI) *m*/*z* calcd. for C_25_H_40_N_2_O_5_ [M + Na]^+^: 471.2830; found: 471.2838.
*N-Boc-Me-D-Leu-L-Leu-OBn* **3b**. The **3b** was synthesized according to the method of **3** from **2b** (10.0 g, 40.8 mmol, 1.0 equiv) and *L*-Leu-OBn·TosOH (9.9 g, 44.9 mmol, 1.1 equiv), HOBT (6.0 g, 44.9 mmol, 1.1 equiv), and EDCI (8.5 g, 44.9 mmol, 1.1 equiv). The crude product was purified by silica gel chromatography (hexane/EtOAc = 90:10, *v*/*v*) to obtain **3b** (14.7 g, 32.8 mmol, 80% yield) as a white solid. [α]^25^_D_ = −245.8 (*c* = 1.0, CHCl_3_). m.p. 80–81 °C. ^1^H NMR (600 MHz, DMSO-*d*_6_) δ 8.21 (d, *J* = 37.5 Hz, 1H), 7.44–7.18 (m, 5H), 5.12 (s, 2H), 4.74 (s, 1H, major), 4.50 (s, 1H, minor), 4.37 (s, 1H), 2.70 (s, 3H), 1.59 (m, 6H), 1.40 (s, 9H), 0.98–0.72 (m, 12H). ^13^C NMR (151 MHz, DMSO-*d*_6_) δ 172.7, 171.6, 136.4, 128.8, 128.4, 128.3, 79.3, 66.4, 50.8, 38.0, 28.5, 24.8, 23.2, 21.6. HRMS (ESI) *m*/*z* calcd. for C_25_H_40_N_2_O_5_ [M + Na]^+^: 471.2830; found: 471.2838.
*N-Boc-Me-L-Leu-D-Leu-OBn* **3c**. The **3c** was synthesized according to the method of **3** from **2a** (10.0 g, 40.8 mmol, 1.0 equiv) and *D*-Leu-OBn·TosOH (9.9 g, 44.9 mmol, 1.1 equiv), HOBT (6.0 g, 44.9 mmol, 1.1 equiv), and EDCI (8.5 g, 44.9 mmol, 1.1 equiv). The crude product was purified by silica gel chromatography (hexane/EtOAc = 90:10, *v*/*v*) to obtain **3c** (15.2 g, 33.9 mmol, 82% yield) as a white solid. [α]^25^_D_ = −427.9 (*c* = 1.0, CHCl_3_). m.p. 80–81 °C. ^1^H NMR (600 MHz, DMSO-*d*_6_) δ 8.42–8.16 (m, 1H), 7.44–7.26 (m, 5H), 5.11 (d, *J* = 4.2 Hz, 2H), 4.71 (d, *J* = 8.5 Hz, 1H, major), 4.49 (d, *J* = 8.4 Hz, 1H, minor), 4.39–4.23 (m, 1H), 2.69 (s, 3H, major), 2.67 (s, 3H, minor), 1.73–1.41 (m, 6H), 1.40 (s, 9H, major), 1.38 (s, 9H, minor), 0.94–0.76 (m, 12H). ^13^C NMR (151 MHz, DMSO-*d*_6_) δ 172.7, 171.7, 136.4, 128.9, 128.5, 128.3, 79.3, 66.4, 50.80, 38.0, 28.5, 24.8, 23.3, 21.6. HRMS (ESI) *m*/*z* calcd. for C_25_H_40_N_2_O_5_ [M + Na]^+^: 471.2830; found: 471.2838.
*N-Boc-Me-D-Leu-D-Leu-OBn* **3d**. The **3d** was synthesized according to the method of **3** from **2b** (10.0 g, 40.8 mmol, 1.0 equiv) and *D*-Leu-OBn·TosOH (9.9 g, 44.9 mmol, 1.1 equiv), HOBT (6.0 g, 44.9 mmol, 1.1 equiv), and EDCI (8.5 g, 44.9 mmol, 1.1 equiv). The crude product was purified by silica gel chromatography (hexane/EtOAc = 90:10, *v*/*v*) to obtain **3d** (14.8 g, 33.0 mmol, 79% yield) as a white solid. [α]^25^_D_ = −215.6 (*c* = 1.0, CHCl_3_). m.p. 80–81 °C. ^1^H NMR (600 MHz, DMSO-*d*_6_) δ 8.20 (d, *J* = 7.8 Hz, 1H), 7.54–7.21 (m, 5H), 5.11 (q, *J* = 12.6 Hz, 2H), 4.67 (s, 1H, major), 4.49 (s, 1H, minor), 4.37 (d, *J* = 15.0 Hz, 1H), 2.73 (s, 3H), 1.73–1.43 (m, 6H), 1.40 (s, 9H), 0.94–0.73 (m, 12H). ^13^C NMR (151 MHz, DMSO-*d*_6_) δ 172.6, 171.9, 136.4, 128.8, 128.5, 128.3, 79.3, 66.4, 50.8, 28.5, 24.9, 24.7, 23.2, 21.5. HRMS (ESI) *m*/*z* calcd. for C_25_H_40_N_2_O_5_ [M + Na]^+^: 471.2830; found: 471.2838.

#### 3.1.3. Synthesis of **4**

The product **3** (33.4 mmol, 1.0 equiv) was dissolved in DCM (0.5 M), followed by addition of TFA (0.12 M). The reaction mixture was stirred at room temperature for 1 h. After that excess TFA and DCM was removed in vacuo to produce **4** as a colorless oil [19]. Such extra peaks are generated because the nitrogen atom in these compounds is bonded to three different groups, C–N bond rotation leads to the emergence of rotamers and consequent splitting in the NMR spectra, as referenced in the literature [18].

*N-Me-L-Leu-L-Leu-OBn* **4a**. The **4a** was synthesized according to the method of **4** from **3a** (15.0 g, 33.4 mmol, 1.0 equiv), TFA (10 mL), DCM (40 mL). After that excess TFA and DCM was removed in vacuo to produce **5a** (10.0 g, 28.7 mmol, 86% yield) as a colorless oil. [α]^25^_D_ = −272.8 (*c* = 1.0, CHCl_3_). ^1^H NMR (600 MHz, DMSO-*d*_6_) δ 8.20 (d, *J* = 8.2 Hz, 1H), 7.52–7.17 (m, 5H), 5.11 (d, *J* = 3.8 Hz, 2H), 4.42 (ddd, *J* = 10.5, 8.1, 4.5 Hz, 1H), 2.93 (t, *J* = 7.2 Hz, 1H), 2.15 (s, 3H), 1.64–1.59 (m, 2H), 1.55–1.48 (m, 1H), 1.27 (m, 2H), 1.04–0.69 (m, 12H). ^13^C NMR (151 MHz, DMSO-*d*_6_) δ 175.2, 172.8, 136.4, 128.9, 128.5, 128.4, 66.44, 62.7, 50.3, 43.1, 34.6, 24.8, 24.7, 23.3, 23.3, 22.9, 21.5. HRMS (ESI) *m*/*z* calcd. for C_20_H_32_N_2_O_3_ [M + H]^+^: 349.2486; found: 349.2487.
*N-Me-D-Leu-L-Leu-OBn* **4b**. The **4b** was synthesized according to the method of **4** from **3b** (15.0 g, 33.4 mmol, 1.0 equiv), TFA (10 mL), DCM (40 mL). After that excess TFA and DCM was removed in vacuo to produce **5b** (9.8 g, 28.1 mmol, 84% yield) as a colorless oil. [α]^25^_D_ = −245.8 (*c* = 1.0, CHCl_3_). ^1^H NMR (600 MHz, DMSO-*d*_6_) δ 8.25 (d, *J* = 8.0 Hz, 1H), 7.44–7.23 (m, 5H), 5.12 (s, 2H), 4.40 (dd, *J* = 11.2, 6.7 Hz, 1H), 2.97 (t, *J* = 7.3 Hz, 1H), 2.19 (s, 3H), 1.65 (m, 3H), 1.55 (d, *J* = 10.8 Hz, 1H), 1.31 (dt, *J* = 15.8, 6.9 Hz, 2H), 0.98–0.72 (m, 12H). ^13^C NMR (151 MHz, DMSO-d6) δ 175.4, 172.8, 136.4, 128.8, 128.5, 128.3, 66.4, 62.7, 50.6, 42.9, 34.7, 24.8, 24.8, 23.3, 23.1, 22.9, 21.4. HRMS (ESI) *m*/*z* calcd. for C_20_H_32_N_2_O_3_ [M + H]^+^: 349.2486; found: 349.2487.
*N-Me-L-Leu-D-Leu-OBn* **4c**. The **4c** was synthesized according to the method of **4** from **3c** (15.0 g, 33.4 mmol, 1.0 equiv), TFA (10 mL), DCM (40 mL). After that excess TFA and DCM was removed in vacuo to produce **5c** (10.4 g, 29.8 mmol, 89% yield) as a colorless oil. [α]^25^_D_ = −427.9 (*c* = 1.0, CHCl_3_). ^1^H NMR (600 MHz, DMSO-*d*_6_) δ 8.27 (d, *J* = 8.0 Hz, 1H), 7.44–7.25 (m, 5H), 5.11 (s, 2H), 4.36 (ddt, *J* = 7.5, 5.2, 2.8 Hz, 1H), 3.02–2.86 (m, 1H), 2.16 (d, *J* = 1.7 Hz, 3H), 1.70–1.55 (m, 3H), 1.53 (dt, *J* = 9.5, 3.6 Hz, 1H), 1.34–1.20 (m, 2H), 0.97–0.71 (m, 12H). ^13^C NMR (151 MHz, DMSO-*d*_6_) δ 175.5, 172.9, 136.4, 128.9, 128.5, 128.4, 66.4, 62.6, 50.6, 42.9, 34.6, 24.8, 24.8, 23.3, 23.1, 23.0, 21.4. HRMS (ESI) *m*/*z* calcd. for C_20_H_32_N_2_O_3_ [M + H]^+^: 349.2486; found: 349.2487.
*N-Me-D-Leu-D-Leu-OBn* **4d**. The **4d** was synthesized according to the method of **4** from **3d** (15.0 g, 33.4 mmol, 1.0 equiv), TFA (10 mL), DCM (40 mL). After that excess TFA and DCM was removed in vacuo to produce **5d** (9.5 g, 27.3 mmol, 81% yield) as a colorless oil. [α]^25^_D_ = −215.6 (*c* = 1.0, CHCl_3_). ^1^H NMR (600 MHz, DMSO-*d*_6_) δ 8.17 (d, *J* = 8.2 Hz, 1H), 7.35 (q, *J* = 8.5 Hz, 5H), 5.12 (d, *J* = 3.3 Hz, 2H), 4.46 (q, *J* = 9.4, 7.5 Hz, 1H), 2.93 (t, *J* = 7.4 Hz, 1H), 2.17 (s, 3H), 1.64 (m, 3H), 1.55 (d, *J* = 11.8 Hz, 1H), 1.38–1.17 (m, 2H), 1.03–0.67 (m, 12H). ^13^C NMR (151 MHz, DMSO-*d*_6_) δ 175.2, 172.8, 136.4, 128.9, 128.5, 128.4, 66.4, 62.8, 50.3, 43.1, 34.6, 24.8, 24.7, 23.3, 23.3, 22.9, 21.4. HRMS (ESI) *m*/*z* calcd. for C_20_H_32_N_2_O_3_ [M + H]^+^: 349.2486; found: 349.2487.

#### 3.1.4. Synthesis of **7**

Catalytic hydrogenation of **3** (33.4 mmol, 1.0 equiv) over 10% Pd/C (4.7 mmol, 0.1 equiv) was carried out at atmospheric pressure in methanol for 2 h. The mixture was filtered and concentrated in vacuo to obtain **7** [20]. The extra peaks are generated because the nitrogen atom in these compounds is bonded to three different groups, and C–N bond rotation leads to the emergence of rotamers and consequent splitting in the NMR spectra, as referenced in the literature [18].

*N-Boc-Me-L-Leu-L-Leu-OH* **7a**. The **7a** was synthesized according to the method of **7** from **3a** (15.0 g, 33.4 mmol, 1.0 equiv), 10% Pd/C (5 g, 4.7 mmol, 0.1 equiv), H_2_ (1 atm), 2 h. The mixture was filtered and concentrated in vacuo to give **7a** (10.0 g, 27.9 mmol, 84% yield) as a colorless oil. [α]25D = −272.8 (*c* = 1.0, CHCl_3_). ^1^H NMR (600 MHz, DMSO-*d*_6_) δ 12.48 (s, 1H), 7.99 (d, *J* = 12.9 Hz, 1H), 4.63 (t, *J* = 8.0 Hz, 1H, major), 4.47 (d, *J* = 10.4 Hz, 1H, minor), 4.31–4.00 (m, 1H), 2.71 (s, 3H), 1.78–1.47 (m, 5H), 1.45–1.27 (m, 9H), 1.10–0.57 (m, 12H). ^13^C NMR (151 MHz, DMSO-*d*_6_) δ 174.4, 171.6, 152.2, 79.3, 50.5, 31.0, 28.5, 25.0, 24.8, 23.6, 23.4, 21.6. HRMS (ESI) *m*/*z* calcd. for C_18_H_34_N_2_O_5_ [M + Na]^+^: 381.2360; found: 381.2261.
*N-Boc-Me-D-Leu-L-Leu-OH* **7b**. The **7b** was synthesized according to the method of **7** from **3b** (15.0 g, 33.4 mmol, 1.0 equiv), 10% Pd/C (5 g, 4.7 mmol, 0.1 equiv), H_2_ (1 atm), 2 h. The mixture was filtered and concentrated in vacuo to give **7b** (9.8 g, 27.3 mmol, 82% yield) as a colorless oil. [α]^25^_D_ = −245.8 (*c* = 1.0, CHCl_3_). ^1^H NMR (600 MHz, DMSO-d_6_) δ 7.93 (dd, J = 25.2, 9.2 Hz, 1H), 4.63 (s, 1H, major), 4.47 (d, J = 10.6 Hz, 1H, minor), 4.35–4.10 (m, 1H), 2.72 (s, 3H), 1.68–1.47 (m, 5H), 1.40 (s, 9H), 1.00–0.64 (m, 12H). ^13^C NMR (151 MHz, DMSO-d_6_) δ 174.5, 171.6, 79.3, 56.8, 50.7, 38.0, 37.6, 30.3, 28.5, 25.0, 24.8, 23.6, 23.4, 21.6. HRMS (ESI) *m*/*z* calcd. for C_18_H_34_N_2_O_5_ [M + Na]^+^: 381.2360; found: 381.2261.
*N-Boc-Me-L-Leu-D-Leu-OH* **7c**. The **7c** was synthesized according to the method of **7** from **3c** (15.0 g, 33.4 mmol, 1.0 equiv), 10% Pd/C (5 g, 4.7 mmol, 0.1 equiv), H_2_ (1 atm), 2 h. The mixture was filtered and concentrated in vacuo to give **7c** (9.5 g, 26.5 mmol, 79% yield) as a colorless oil. [α]^25^_D_ = −427.9 (*c* = 1.0, CHCl_3_). ^1^H NMR (600 MHz, DMSO-*d*_6_) δ 12.47 (s, 1H), 7.98 (d, *J* = 12.9 Hz, 1H), 4.62 (t, *J* = 8.0 Hz, 1H, major), 4.46 (d, *J* = 10.4 Hz, 1H, minor), 4.31–4.00 (m, 1H), 2.71 (s, 3H), 1.78–1.47 (m, 5H), 1.45–1.27 (m, 9H), 1.10–0.57 (m, 12H). ^13^C NMR (151 MHz, DMSO-*d*_6_) δ 174.4, 171.6, 152.2, 79.3, 50.5, 31.0, 28.5, 25.0, 24.8, 23.6, 23.4, 21.6. HRMS (ESI) *m*/*z* calcd. for C_18_H_34_N_2_O_5_ [M + Na]^+^: 381.2360; found: 381.2261.
*N-Boc-Me-D-Leu-D-Leu-OH* **7d**. The **7d** was synthesized according to the method of **7** from **3d** (15.0 g, 33.4 mmol, 1.0 equiv), 10% Pd/C (5 g, 4.7 mmol, 0.1 equiv), H_2_ (1 atm), 2 h. The mixture was filtered and concentrated in vacuo to give **7d** (10.3 g, 28.8 mmol, 86% yield) as a colorless oil. [α]^25^_D_ = −215.6 (*c* = 1.0, CHCl_3_). ^1^H NMR (600 MHz, DMSO-*d*_6_) δ 7.92 (dd, *J* = 25.2, 9.2 Hz, 1H), 4.62 (s, 1H, major), 4.46 (d, *J* = 10.6 Hz, 1H, minor), 4.35–4.11 (m, 1H), 2.72 (s, 3H), 1.68–1.47 (m, 5H), 1.40 (s, 9H), 1.00–0.64 (m, 12H). ^13^C NMR (151 MHz, DMSO-*d*_6_) δ 174.5, 171.6, 79.3, 56.8, 50.7, 38.0, 37.6, 30.3, 28.5, 25.0, 24.8, 23.6, 23.4, 21.6. HRMS (ESI) *m*/*z* calcd. for C_18_H_34_N_2_O_5_ [M + Na]^+^: 381.2360; found: 381.2261.

#### 3.1.5. Synthesis of **5**

The product **4** (28.7 mmol, 1.0 equiv) and *N*-Boc-AA-OH (31.5 mmol, 1.1 equiv) and HOBT (31.5 mmol, 1.1 equiv) were added to dry DCM (0.5 M) at 0 °C. At this temperature, the EDCI (31.5 mmol, 1.1 equiv) was slowly added. The reaction mixture was warmed to 23 °C and stirred for 12 h. After most of the DCM was removed in vacuo, the reaction solution was washed with two portions of 0.5 N hydrochloric acid and two portions of saturated Na_2_CO_3_ solution. Then, the layers were separated, and the combined organic layer was dried over Na_2_SO_4_. The solvent was removed in vacuo to obtain dipeptide. The crude product was purified by silica gel chromatography (EtOAc/petroleum ether = 80:20, *v*/*v*) to obtain **5** as a white solid. The extra peaks are generated because the nitrogen atom in these compounds is bonded to three different groups, and C–N bond rotation leads to the emergence of rotamers and consequent splitting in the NMR spectra, as referenced in the literature [18].

*N-Boc-D-Leu-Me-D-Leu-D-Leu-OBn* **5a**. The **5a** was synthesized according to the method of **5** from **4d** (10.0 g, 28.7 mmol, 1.0 equiv) and *N*-Boc-*D*-Leu-OH (7.2 g, 31.5 mmol, 1.1 equiv), HOBT (4.2 g, 31.5 mmol, 1.1 equiv), and EDCI (6.0 g, 31.5 mmol, 1.1 equiv). The crude product was purified by silica gel chromatography (hexane/EtOAc = 80:20, *v*/*v*) to obtain **5h** (12.8 g, 22.8 mmol, 78% yield) as a white solid. [α]^25^_D_ = −267.6 (*c* = 1.0, CHCl_3_). m.p. 117–118 °C. ^1^H NMR (600 MHz, CDCl_3_) δ 7.28 (td, *J* = 13.7, 6.9 Hz, 5H), 6.45 (d, *J* = 8.3 Hz, 1H), 5.33–5.18 (m, 1H), 5.15–4.99 (m, 2H), 4.71–4.43 (m, 2H), 2.88 (s, 3H, major), 2.67 (s, 3H, minor), 1.77–1.49 (m, 9H), 1.40 (s, 9H, major), 1.35 (s, 9H, minor), 1.06–0.73 (m, 18H). ^13^C NMR (151 MHz, CDCl_3_) δ 174.6, 172.4, 170.5, 155.7, 135.6, 128.5, 128.5, 128.3, 128.1, 128.1, 66.8, 54.3, 50.7, 49.2, 41.6, 40.9, 35.6, 30.1, 28.3, 28.2, 24.8, 24.7, 24.6, 23.4, 23.0, 22.8, 21.9, 21.7, 21.6. HRMS (ESI) *m*/*z* calcd. for C_31_H_51_N_3_O_6_ [M + Na]^+^: 584.3670; found: 584.3672.
*N-Boc-D-Leu-Me-L-Leu-L-Leu-OBn* **5b**. The **5b** was synthesized according to the method of **5** from **4a** (10.0 g, 28.7 mmol, 1.0 equiv) and *N*-Boc-*D*-Leu-OH (7.2 g, 31.5 mmol, 1.1 equiv), HOBT (4.2 g, 31.5 mmol, 1.1 equiv), and EDCI (6.0 g, 31.5 mmol, 1.1 equiv). The crude product was purified by silica gel chromatography (hexane/EtOAc = 80:20, *v*/*v*) to obtain **5b** (13.8 g, 24.6 mmol, 85% yield) as a white solid. [α]^25^_D_ = −388.8 (*c* = 1.0, CHCl_3_). m.p. 117–118 °C. ^1^H NMR (600 MHz, DMSO-*d*_6_) δ 8.44 (d, *J* = 7.5 Hz, 1H, minor), 7.73 (d, *J* = 7.6 Hz, 1H, major), 7.45–7.17 (m, 5H), 7.09 (d, *J* = 7.0 Hz, 1H, major), 6.76 (d, *J* = 8.6 Hz, 1H, minor), 5.22–4.95 (m, 3H), 4.57–4.20 (m, 2H), 2.90 (s, 3H, major), 2.76 (s, 3H, minor), 1.76–1.39 (m, 7H), 1.34 (s, 9H), 1.26 (q, *J* = 7.0, 5.0 Hz, 2H), 1.00–0.65 (m, 18H). ^13^C NMR (151 MHz, DMSO-*d*_6_) δ 173.9, 172.5, 171.3, 156.4, 136.4, 128.9, 128.4, 128.2, 78.6, 66.3, 54.3, 51.1, 49.6, 36.9, 30.9, 28.6, 28.6, 24.9, 24.7, 24.7, 23.7, 23.5, 23.2, 22.0, 21.6, 21.5. HRMS (ESI) *m*/*z* calcd. for C_31_H_51_N_3_O_6_ [M + Na]^+^: 584.3670; found: 584.3672.
*N-Boc-L-Leu-Me-D-Leu-L-Leu-OBn* **5c**. The **5c** was synthesized according to the method of **5** from **4b** (10.0 g, 28.7 mmol, 1.0 equiv) and *N*-Boc-*L*-Leu-OH (7.2 g, 31.5 mmol, 1.1 equiv), HOBT (4.2 g, 31.5 mmol, 1.1 equiv), and EDCI (6.0 g, 31.5 mmol, 1.1 equiv). The crude product was purified by silica gel chromatography (hexane/EtOAc = 80:20, *v*/*v*) to obtain **5c** (14.2 g, 25.3 mmol, 87% yield) as a white solid. [α]^25^_D_ = −375.6 (*c* = 1.0, CHCl_3_). m.p. 117–118 °C. ^1^H NMR (600 MHz, DMSO-*d*_6_) δ 8.34 (d, *J* = 7.5 Hz, 1H, minor), 7.73 (d, *J* = 7.6 Hz, 1H, major), 7.50–7.17 (m, 5H), 7.17–6.67 (m, 1H), 5.27–4.83 (m, 3H), 4.64–4.16 (m, 2H), 2.90 (s, 3H, major), 2.76 (s, 3H, minor), 1.81–1.38 (m, 7H), 1.34 (s, 9H), 1.26 (q, *J* = 6.1, 5.0 Hz, 2H), 1.01–0.70 (m, 18H). ^13^C NMR (151 MHz, DMSO-*d*_6_) δ 173.8, 172.4, 171.2, 156.3, 136.3, 128.8, 128.3, 128.1, 78.6, 66.2, 54.19, 51.0, 49.5, 36.8, 30.8, 28.5, 28.5, 24.8, 24.6, 24.6, 23.6, 23.4, 23.1, 21.9, 21.5, 21.4. HRMS (ESI) *m*/*z* calcd. for C_31_H_51_N_3_O_6_ [M + Na]^+^: 584.3670; found: 584.3672.
*N-Boc-D-Leu-Me-D-Leu-L-Leu-OBn* **5d**. The **5d** was synthesized according to the method of **5** from **4b** (10.0 g, 28.7 mmol, 1.0 equiv) and *N*-Boc-*D*-Leu-OH (7.2 g, 31.5 mmol, 1.1 equiv), HOBT (4.2 g, 31.5 mmol, 1.1 equiv), and EDCI (6.0 g, 31.5 mmol, 1.1 equiv). The crude product was purified by silica gel chromatography (hexane/EtOAc = 80:20, *v*/*v*) to obtain **5d** (13.5 g, 24.1 mmol, 83% yield) as a white solid. [α]^25^_D_ = −255.8.6 (*c* = 1.0, CHCl_3_). m.p. 117–118 °C. ^1^H NMR (600 MHz, DMSO-*d*_6_) δ 8.21 (d, *J* = 7.7 Hz, 1H, minor), 8.12 (d, *J* = 7.6 Hz, 1H, major), 7.34 (m, 5H), 6.96 (d, *J* = 8.2 Hz, 1H), 5.14 (m, 1H), 5.10 (s, 2H), 4.69–4.10 (m, 2H), 2.94 (s, 3H, major), 2.55 (s, 3H, minor), 1.78–1.44 (m, 7H), 1.36 (s, 9H), 1.31–1.15 (m, 2H), 0.83 (m, 18H). ^13^C NMR (151 MHz, DMSO-*d*_6_) δ 173.8, 172.6, 171.5, 156.0, 136.4, 128.9, 128.4, 128.2, 78.3, 66.3, 53.9, 50.8, 49.4, 37.6, 30.8, 28.6, 28.5, 24.7, 24.7, 24.6, 23.7, 23.6, 23.2, 21.9, 21.6. HRMS (ESI) *m*/*z* calcd. for C_31_H_51_N_3_O_6_ [M + Na]^+^: 584.3670; found: 584.3672.
*N-Boc-L-Leu-Me-L-Leu-D-Leu-OBn* **5e**. The **5e** was synthesized according to the method of **5** from **4c** (10.0 g, 28.7 mmol, 1.0 equiv) and *N*-Boc-*L*-Leu-OH (7.2 g, 31.5 mmol, 1.1 equiv), HOBT (4.2 g, 31.5 mmol, 1.1 equiv), and EDCI (6.0 g, 31.5 mmol, 1.1 equiv). The crude product was purified by silica gel chromatography (hexane/EtOAc = 80:20, *v*/*v*) to obtain **5e** (13.6 g, 24.2 mmol, 84% yield) as a white solid. [α]^25^_D_ = −365.6 (*c* = 1.0, CHCl_3_). m.p. 117–118 °C. ^1^H NMR (600 MHz, DMSO-*d*_6_) δ 8.42 (dd, *J* = 14.4, 7.5 Hz, 1H, minor), 7.78 (d, *J* = 7.8 Hz, 1H, major), 7.34 (m, 5H), 7.05 (d, *J* = 7.0 Hz, 1H, major), 6.78 (d, *J* = 8.6 Hz, 1H, minor), 5.21–5.00 (m, 3H), 4.35 (d, *J* = 7.5 Hz, 2H), 2.83 (s, 3H, major), 2.73 (s, 3H, minor), 1.72–1.43 (m, 7H), 1.36 (s, 9H), 1.31 (s, 2H), 0.84 (m, 18H). ^13^C NMR (151 MHz, DMSO-*d*_6_) δ 173.8, 172.5, 171.2, 156.3, 136.3, 128.9, 128.5, 128.4, 128.4, 78.60, 66.4, 54.1, 50.9, 49.7, 37.1, 30.8, 28.6, 24.9, 24.7, 24.7, 23.7, 23.6, 23.0, 21.9, 21.8, 21.6. HRMS (ESI) *m*/*z* calcd. for C_31_H_51_N_3_O_6_ [M + Na]^+^: 584.3670; found: 584.3672.
*N-Boc-D-Leu-Me-L-Leu-D-Leu-OBn* **5f**. The **5f** was synthesized according to the method of **5** from **4c** (10.0 g, 28.7 mmol, 1.0 equiv) and *N*-Boc-*D*-Leu-OH (7.2 g, 31.5 mmol, 1.1 equiv), HOBT (4.2 g, 31.5 mmol, 1.1 equiv), and EDCI (6.0 g, 31.5 mmol, 1.1 equiv). The crude product was purified by silica gel chromatography (hexane/EtOAc = 80:20, *v*/*v*) to obtain **5f** (13.0 g, 23.2 mmol, 80% yield) as a white solid. [α]^25^_D_ = −369.6 (*c* = 1.0, CHCl_3_). m.p. 117–118 °C. ^1^H NMR (600 MHz, DMSO-*d*_6_) δ 8.43 (dd, *J* = 14.6, 7.4 Hz, 1H, minor), 7.79 (d, *J* = 7.7 Hz, 1H, major), 7.35 (q, *J* = 9.1, 8.7 Hz, 5H), 7.06 (d, *J* = 7.0 Hz, 1H, major), 6.79 (d, *J* = 8.6 Hz, 1H, minor), 5.12 (dd, *J* = 14.7, 5.5 Hz, 3H), 4.59–4.14 (m, 2H), 2.84 (s, 3H, major), 2.74 (s, 3H, minor), 1.83–1.43 (m, 7H), 1.37 (s, 9H), 1.22 (s, 2H), 0.85 (m, 18H). ^13^C NMR (151 MHz, DMSO-*d*_6_) δ 172.4, 128.8, 78.6, 54.1, 50.9, 49.6, 37.1, 30.7, 28.6, 24.9, 24.7, 24.6, 23.7, 23.6, 23.0, 21.9, 21.8, 21.5. HRMS (ESI) *m*/*z* calcd. for C_31_H_51_N_3_O_6_ [M + Na]^+^: 584.3670; found: 584.3672.
*N-Boc-L-Leu-Me-D-Leu-D-Leu-OBn* **5g**. The **5g** was synthesized according to the method of **5** from **4d** (10.0 g, 28.7 mmol, 1.0 equiv) and *N*-Boc-L-Leu-OH (7.2 g, 31.5 mmol, 1.1 equiv), HOBT (4.2 g, 31.5 mmol, 1.1 equiv), and EDCI (6.0 g, 31.5 mmol, 1.1 equiv). The crude product was purified by silica gel chromatography (hexane/EtOAc = 80:20, *v*/*v*) to obtain **5g** (13.2 g, 23.5 mmol, 81% yield) as a white solid. [α]^25^_D_ = −323.6 (*c* = 1.0, CHCl_3_). m.p. 117–118 °C. ^1^H NMR (600 MHz, CDCl_3_) δ 7.37–7.17 (m, 5H), 6.74 (d, *J* = 8.1 Hz, 1H), 5.16 (d, *J* = 15.8 Hz, 1H), 5.15–5.03 (m, 3H), 4.52 (td, *J* = 9.1, 4.6 Hz, 2H), 2.93 (s, 3H), 1.81–1.43 (m, 9H), 1.36 (s, 9H), 1.00–0.75 (m, 18H). ^13^C NMR (151 MHz, CDCl_3_) δ 174.5, 172.3, 170.5, 156.0, 135.6, 128.5, 128.1, 128.0, 79.7, 66.7, 54.7, 51.0, 49.3, 41.7, 40.4, 36.0, 30.4, 28.3, 24.9, 24.8, 24.7, 23.2, 23.2, 22.8, 21.8, 21.6, 21.5. HRMS (ESI) *m*/*z* calcd. for C_31_H_51_N_3_O_6_ [M + Na]^+^: 584.3670; found: 584.3672.
*N-Boc-D-Pro-Me-L-Leu-L-Leu-OBn* **5h**. The **5h** was synthesized according to the method of **5** from **4a** (10.0 g, 28.7 mmol, 1.0 equiv) and *N*-Boc-*L*-Pro-OH (6.7 g, 31.5 mmol, 1.1 equiv), HOBT (4.2 g, 31.5 mmol, 1.1 equiv), and EDCI (6.0 g, 31.5 mmol, 1.1 equiv). The crude product was purified by silica gel chromatography (hexane/EtOAc = 80:20, *v*/*v*) to obtain **5h** (13.0 g, 23.8 mmol, 83% yield) as a white solid. [α]^25^_D_ = −175.6 (*c* = 1.0, CHCl_3_). m.p. 158–159 °C. ^1^H NMR (600 MHz, CDCl_3_) δ7.39–7.26 (m, 5H, major), 7.17 (d, *J* = 7.9 Hz, 5H, minor), 5.40 (dd, *J* = 11.0, 4.5 Hz, 1H), 5.12 (q, *J* = 12.6 Hz, 2H), 4.64–4.49 (m, 2H), 3.65–3.50 (m, 1H), 3.47 (dd, *J* = 5.0, 2.1 Hz, 1H), 3.03 (s, 3H, minor)2.94 (s, 3H, major), 2.22–2.03 (m, 2H), 1.98–1.84 (m, 2H), 1.84–1.53 (m, 4H), 1.39 (s, 9H), 1.01–0.79 (m, 12H). ^13^C NMR (151 MHz, CDCl_3_) δ 174.0, 172.3, 171.2, 154.8, 136.0, 128.4, 127.9, 127.9, 66.4, 55.8, 55.0, 51.4, 47.0, 39.8, 35.7, 30.8, 29.5, 28.4, 25.0, 24.9, 23.4, 22.9, 21.6, 21.4. HRMS (ESI) *m*/*z* calcd. for C_30_H_47_N_3_O_6_ [M + Na]^+^: 586.3357; found: 586.3357.
*N-Boc-D-Phe-Me-L-Leu-L-Leu-OBn* **5i**. The **5i** was synthesized according to the method of **5** from **4a** (10.0 g, 28.7 mmol, 1.0 equiv) and *N*-Boc-*D*-Phe-OH (8.3 g, 31.5 mmol, 1.1 equiv), HOBT (4.2 g, 31.5 mmol, 1.1 equiv), and EDCI (6.0 g, 31.5 mmol, 1.1 equiv). The crude product was purified by silica gel chromatography (hexane/EtOAc = 80:20, *v*/*v*) to obtain **5i** (15.0 g, 25.2 mmol, 88% yield) as a white solid. [α]^25^_D_ = −333.0 (*c* = 1.0, CHCl_3_). m.p. 119–120 °C. ^1^H NMR (600 MHz, Chloroform-*d*) δ 7.45–7.11 (m, 10H), 6.77 (d, *J* = 8.1 Hz, 1H), 5.25 (d, *J* = 7.6 Hz, 1H), 5.19–5.02 (m, 3H), 4.78 (d, *J* = 7.6 Hz, 1H), 4.54 (d, *J* = 7.4 Hz, 1H), 2.98 (d, *J* = 7.4 Hz, 2H), 2.66 (s, 3H), 1.80–1.53 (m, 4H), 1.40 (d, *J* = 2.7 Hz, 9H), 0.97–0.75 (m, 12H). ^13^C NMR (151 MHz, Chloroform-*d*) δ 173.2, 172.2, 170.4, 155.5, 135.9, 135.7, 129.3, 128.6, 128.5, 128.2, 128.0, 127.1, 66.7, 54.9, 52.0, 51.0, 40.4, 39.2, 35.9, 30.5, 28.3, 24.9, 24.4, 23.1, 22.8, 21.9, 21.5. HRMS (ESI) *m*/*z* calcd. for C_34_H_49_N_3_O_6_ [M + Na]^+^: 618.3514; found: 618.3514.
*N-Boc-Gly-Me-L-Leu-L-Leu-OBn* **5j**. The **5j** was synthesized according to the method of **5** from **4a** (10.0 g, 28.7 mmol, 1.0 equiv) and *N*-Boc-Gly-OH (4.9 g, 31.5 mmol, 1.1 equiv), HOBT (4.2 g, 31.5 mmol, 1.1 equiv), and EDCI (6.0 g, 31.5 mmol, 1.1 equiv). The crude product was purified by silica gel chromatography (hexane/EtOAc = 80:20, *v*/*v*) to obtain **5j** (11.5 g, 24.7 mmol, 79% yield) as a white solid. [α]^25^_D_ = −205.3 (*c* = 1.0, CHCl_3_). m.p. 117–118 °C. ^1^H NMR (600 MHz, DMSO-*d*_6_) δ 8.20 (d, *J* = 7.6 Hz, 1H), 7.52–7.04 (m, 5H), 6.70 (t, *J* = 5.8 Hz, 1H), 5.11 (t, *J* = 13.2 Hz, 2H), 5.06 (s, 1H), 4.30 (ddd, *J* = 10.3, 7.5, 4.9 Hz, 1H), 3.93–3.65 (m, 2H), 2.82 (s, 3H, major), 2.77 (s, 3H, minor), 1.70–1.44 (m, 5H), 1.38 (s, 9H), 1.33 (d, *J* = 3.9 Hz, 1H), 0.96–0.64 (m, 12H). ^13^C NMR (151 MHz, DMSO-*d*_6_) δ 172.6, 171.6, 170.2, 156.2, 136.4, 128.9, 128.5, 128.4, 128.3, 78.5, 78.4, 66.5, 66.4, 54.2, 51.0, 42.5, 37.5, 30.1, 28.7, 24.7, 23.6, 23.2, 21.9, 21.6. HRMS (ESI) *m*/*z* calcd. for C_27_H_43_N_3_O_6_ [M + Na]^+^: 528.3044; found: 528.3043.
*N-Boc-D-Ala-Me-L-Leu-L-Leu-OBn* **5k**. The **5k** was synthesized according to the method of **5** from **4a** (10.0 g, 28.7 mmol, 1.0 equiv) and *N*-Boc-*D*-Ala-OH (5.9 g, 31.5 mmol, 1.1 equiv), HOBT (4.2 g, 31.5 mmol, 1.1 equiv), and EDCI (6.0 g, 31.5 mmol, 1.1 equiv). The crude product was purified by silica gel chromatography (hexane/EtOAc = 80:20, *v*/*v*) to obtain **5k** (12.5 g, 24.3 mmol, 84% yield) as a white solid. [α]^25^_D_ = −555.6 (*c* = 1.0, CHCl_3_). m.p. 105–106 °C. ^1^H NMR (600 MHz, Chloroform-*d*) δ 7.32 (dp, *J* = 14.9, 7.1, 6.5 Hz, 5H), 6.64 (d, *J* = 8.1 Hz, 1H), 5.34 (d, *J* = 7.4 Hz, 1H), 5.23–5.05 (m, 3H), 4.55 (t, *J* = 7.3 Hz, 2H), 2.93 (s, 3H), 1.74 (d, *J* = 8.1 Hz, 1H), 1.69–1.53 (m, 4H), 1.40 (s, 9H), 1.29 (d, *J* = 6.8 Hz, 3H), 1.01–0.75 (m, 12H). ^13^C NMR (151 MHz, Chloroform-*d*) δ 174.5, 172.3, 170.4, 155.6, 135.6, 128.5, 128.2, 128.1, 79.7, 66.8, 54.9, 51.0, 46.8, 40.6, 35.9, 30.5, 28.3, 24.9, 24.9, 23.1, 22.7, 21.7, 21.6, 18.0. HRMS (ESI) *m*/*z* calcd. for C_28_H_45_N_3_O_6_ [M + Na]^+^: 542.3201; found: 542.3209.
*N-Boc-O-Me-D-Ser-Me-L-Leu-L-Leu-OBn* **5l**. The **5l** was synthesized according to the method of **5** from **4a** (10.0 g, 28.7 mmol, 1.0 equiv) and *N*-Boc-*O*-Me-*D*-Ser-OH (6.9 g, 31.5 mmol, 1.1 equiv), HOBT (4.2 g, 31.5 mmol, 1.1 equiv), and EDCI (6.0 g, 31.5 mmol, 1.1 equiv). The crude product was purified by silica gel chromatography (hexane/EtOAc = 80:20, *v*/*v*) to obtain **5l** (14.0 g, 25.5 mmol, 89% yield) as a white solid. [α]^25^_D_ = −275.6 (*c* = 1.0, CHCl_3_). m.p. 121–122 °C. ^1^H NMR (600 MHz, DMSO-*d*_6_) δ 7.72 (d, *J* = 7.7 Hz, 1H), 7.46–7.24 (m, 5H), 7.12 (d, *J* = 6.7 Hz, 1H), 5.15–5.08 (m, 2H), 5.06 (d, *J* = 12.6 Hz, 1H), 4.58 (d, *J* = 6.8 Hz, 1H), 4.41–4.24 (m, 1H), 3.44 (t, *J* = 6.8 Hz, 2H), 3.22 (s, 3H), 2.91 (s, 3H, major), 2.77 (s, 3H, minor), 1.77–1.38 (m, 6H), 1.34 (s, 9H), 1.03–0.60 (m, 12H). ^13^C NMR (151 MHz, DMSO-*d*_6_) δ 172.5, 172.0, 171.3, 156.0, 136.4, 128.9, 128.4, 128.2, 78.9, 72.1, 66.3, 58.9, 54.5, 51.1, 50.7, 39.5, 36.9, 31.1, 28.5, 24.7, 24.5, 23.8, 23.2, 21.7, 21.5. HRMS (ESI) *m*/*z* calcd. for C_29_H_47_N_3_O_7_ [M + Na]^+^: 572.3306; found: 572.3307.
*N-Boc-O-Me-D-Tyr-Me-L-Leu-L-Leu-OBn* **5m**. The **5m** was synthesized according to the method of **5** from **4a** (10.0 g, 28.7 mmol, 1.0 equiv) and *N*-Boc-*O*-Me-*D*-Tyr-OH (9.3 g, 31.5 mmol, 1.1 equiv), HOBT (4.2 g, 31.5 mmol, 1.1 equiv), and EDCI (6.0 g, 31.5 mmol, 1.1 equiv). The crude product was purified by silica gel chromatography (hexane/EtOAc = 80:20, *v*/*v*) to obtain **5m** (17.0 g, 27.2 mmol, 95% yield) as a white solid. [α]^25^_D_ = −157.3 (*c* = 1.0, CHCl_3_). m.p. 120–121 °C. ^1^H NMR (600 MHz, Chloroform-*d*) δ 7.34 (ddd, *J* = 12.7, 7.5, 3.6 Hz, 5H), 7.12 (d, *J* = 8.2 Hz, 2H), 6.82 (d, *J* = 8.4 Hz, 3H), 5.29–5.00 (m, 4H), 4.74 (d, *J* = 7.4 Hz, 1H), 4.54 (td, *J* = 8.2, 6.1 Hz, 1H), 3.79 (s, 3H), 2.93 (d, *J* = 7.5 Hz, 2H), 2.68 (s, 3H), 1.84–1.52 (m, 4H), 1.40 (s, 9H), 1.35–1.24 (m, 2H), 0.95–0.76 (m, 12H). ^13^C NMR (151 MHz, Chloroform-*d*) δ 173.3, 172.2, 170.4, 158.8, 155.5, 130.3, 128.5, 128.0, 114.0, 66.7, 55.2, 54.9, 52.1, 51.0, 40.4, 38.3, 35.9, 30.5, 28.3, 24.9, 24.4, 23.1, 22.8, 22.0, 21.5. HRMS (ESI) *m*/*z* calcd. for C_35_H_51_N_3_O_7_ [M + Na]^+^: 648.3620; found: 648.3621.
*N-Boc-Methyl ester-D-Asp-Me-L-Leu-L-Leu-OBn* **5n**. The **5n** was synthesized according to the method of **5** from **4a** (10.0 g, 28.7 mmol, 1.0 equiv) and *N*-Boc-Methyl ester-*D*-Asp-OH (7.8 g, 31.5 mmol, 1.1 equiv), HOBT (4.2 g, 31.5 mmol, 1.1 equiv), and EDCI (6.0 g, 31.5 mmol, 1.1 equiv). The crude product was purified by silica gel chromatography (hexane/EtOAc = 80:20, *v*/*v*) to obtain **5n** (14.0 g, 24.3 mmol, 84% yield) as a white solid. [α]^25^_D_ = −269.7 (*c* = 1.0, CHCl_3_). m.p. 124–125 °C. ^1^H NMR (600 MHz, DMSO-*d*_6_) δ 7.80 (d, *J* = 7.7 Hz, 1H, minor), 7.74 (d, *J* = 7.8 Hz, 1H, major), 7.34 (m, 6H), 5.18–4.95 (m, 3H), 4.80–4.52 (m, 1H), 4.47–4.21 (m, 1H), 3.54 (d, *J* = 14.7 Hz, 3H), 3.00 (s, 3H, major), 2.91 (s, 3H, minor), 2.89–2.68 (m, 1H), 2.65–2.52 (m, 1H), 1.60 (m, 5H), 1.36 (s, 9H), 0.98–0.64 (m, 12H). ^13^C NMR (151 MHz, DMSO-*d*_6_) δ 172.50, 172.03, 171.22, 171.02, 155.39, 136.40, 128.85, 128.46, 128.19, 78.77, 66.34, 60.19, 51.93, 51.84, 51.07, 50.94, 47.07, 36.94, 36.64, 36.32, 30.95, 30.77, 28.49, 24.70, 23.74, 23.22, 21.82, 21.55. HRMS (ESI) *m*/*z* calcd. for C_30_H_47_N_3_O_8_ [M + Na]^+^: 600.3256; found: 600.3265.
*N-Boc-3-Flu-D-Phe-Me-L-Leu-L-Leu-OBn* **5o**. The **5o** was synthesized according to the method of **5** from **4a** (10.0 g, 28.7 mmol, 1.0 equiv) and *N*-Boc-3-Flu-*D*-Phe-OH (9.3 g, 31.5 mmol, 1.1 equiv), HOBT (4.2 g, 31.5 mmol, 1.1 equiv), and EDCI (6.0 g, 31.5 mmol, 1.1 equiv). The crude product was purified by silica gel chromatography (hexane/EtOAc = 80:20, *v*/*v*) to obtain **5o** (15.5 g, 25.2 mmol, 88% yield) as a white solid. [α]^25^_D_ = −555.6 (*c* = 1.0, CHCl_3_). m.p. 121–122 °C. ^1^H NMR (600 MHz, DMSO-*d*_6_) δ 7.66 (d, *J* = 7.8 Hz, 1H), 7.42 (d, *J* = 6.2 Hz, 1H), 7.38–7.24 (m, 5H), 7.20–6.88 (m, 3H), 5.16–5.06 (m, 2H), 5.04 (d, *J* = 12.4 Hz, 1H), 4.66 (q, *J* = 7.2 Hz, 1H), 4.31 (ddd, *J* = 12.0, 7.7, 5.0 Hz, 1H), 2.95–2.83 (m, 2H), 2.73 (s, 3H), 1.80–1.41 (m, 4H), 1.31 (s, 9H), 1.26–1.10 (m, 2H), 0.97–0.52 (m, 12H). ^13^C NMR (151 MHz, DMSO-*d*_6_) δ 172.8, 172.5, 171.2, 163.3, 161.7, 156.2, 136.4, 130.5, 130.4, 128.9, 128.5, 128.4, 128.3, 128.1, 126.1, 116.7, 116.6, 113.8, 113.7, 78.9, 66.2, 60.2, 54.4, 52.0, 51.1, 37.3, 36.7, 30.9, 28.5, 24.7, 24.1, 23.7, 23.2, 21.7, 21.4, 21.2. HRMS (ESI) *m*/*z* calcd. for C_34_H_48_FN_3_O_6_ [M + Na]^+^: 636.3420; found: 636.3421.
*N-Boc-2-Me-D-Phe-Me-L-Leu-L-Leu-OBn* **5p**. The **5p** was synthesized according to the method of **5** from **4a** (10.0 g, 28.7 mmol, 1.0 equiv) and *N*-Boc-2-Me-*D*-Ser-OH (8.8 g, 31.5 mmol, 1.1 equiv), HOBT (4.2 g, 31.5 mmol, 1.1 equiv), and EDCI (6.0 g, 31.5 mmol, 1.1 equiv). The crude product was purified by silica gel chromatography (hexane/EtOAc = 80:20, *v*/*v*) to obtain **5p** (15.0 g, 24.6 mmol, 86% yield) as a white solid. [α]^25^_D_ = −505.6 (*c* = 1.0, CHCl_3_). m.p. 142–143 °C. ^1^H NMR (600 MHz, Chloroform-*d*) δ 7.41–7.26 (m, 5H), 7.09 (s, 4H), 6.79 (d, *J* = 8.0 Hz, 1H), 5.21 (d, *J* = 7.5 Hz, 1H), 5.19–5.04 (m, 3H), 4.75 (d, *J* = 7.5 Hz, 1H), 4.54 (d, *J* = 7.4 Hz, 1H), 3.02–2.84 (m, 2H), 2.67 (s, 3H), 2.32 (s, 3H), 1.73–1.51 (m, 4H), 1.39 (s, 9H), 1.31 (td, *J* = 11.2, 10.3, 5.9 Hz, 1H), 1.05 (s, 1H), 1.01–0.70 (m, 12H). ^13^C NMR (151 MHz, Chloroform-*d*) δ 173.2, 172.2, 170.5, 155.6, 136.7, 135.7, 132.8, 129.2, 129.2, 128.5, 128.1, 128.0, 66.7, 54.9, 52.0, 51.0, 40.3, 38.7, 35.9, 30.6, 28.3, 24.9, 24.4, 23.1, 22.8, 21.9, 21.5, 21.0. HRMS (ESI) *m*/*z* calcd. for C_35_H_51_N_3_O_6_ [M + Na]^+^: 632.3420; found: 632.3421.
*N-Boc-4-Chl-D-Phe-Me-L-Leu-L-Leu-OBn* **5q**. The **5q** was synthesized according to the method of **5** from **4a** (10.0 g, 28.7 mmol, 1.0 equiv) and *N*-Boc-3-Chl-*D*-Phe-OH (9.4 g, 31.5 mmol, 1.1 equiv), HOBT (4.2 g, 31.5 mmol, 1.1 equiv), and EDCI (6.0 g, 31.5 mmol, 1.1 equiv). The crude product was purified by silica gel chromatography (hexane/EtOAc = 80:20, *v*/*v*) to obtain **5q** (16.0 g, 25.4 mmol, 89% yield) as a white solid. [α]^25^_D_ = −399.6 (*c* = 1.0, CHCl_3_). m.p. 151–152 °C. ^1^H NMR (600 MHz, DMSO-*d*_6_) δ 7.64 (d, *J* = 7.7 Hz, 1H), 7.42 (d, *J* = 6.1 Hz, 1H), 7.39–7.20 (m, 8H), 5.21–5.04 (m, 2H), 5.02 (d, *J* = 9.1 Hz, 1H), 4.66 (d, *J* = 7.2 Hz, 1H), 4.54–4.18 (m, 1H), 2.97–2.80 (m, 2H), 2.74 (s, 3H), 1.81–1.43 (m, 4H), 1.30 (s, 9H), 1.25 (m, 2H), 0.93–0.61 (m, 12H). ^13^C NMR (151 MHz, DMSO-*d*_6_) δ 172.8, 172.4, 171.2, 156.2, 136.4, 131.8, 131.8, 128.9, 128.5, 128.4, 128.1, 78.9, 66.2, 54.4, 52.0, 51.1, 36.9, 36.7, 31.0, 28.5, 24.7, 24.1, 23.7, 23.2, 21.8, 21.4. HRMS (ESI) *m*/*z* calcd. for C_34_H_48_ClN_3_O_6_ [M + Na]^+^: 652.3124; found: 652.3132.
*N-Boc-4-Bro-D-Phe-Me-L-Leu-L-Leu-OBn* **5r**. The **5r** was synthesized according to the method of **5** from **4a** (10.0 g, 28.7 mmol, 1.0 equiv) and *N*-Boc-4-Bro-*D*-Phe-OH (10.8 g, 31.5 mmol, 1.1 equiv), HOBT (4.2 g, 31.5 mmol, 1.1 equiv), and EDCI (6.0 g, 31.5 mmol, 1.1 equiv). The crude product was purified by silica gel chromatography (hexane/EtOAc = 80:20, *v*/*v*) to obtain **5r** (17.0 g, 25.2 mmol, 88% yield) as a white solid. [α]^25^_D_ = −375.6 (*c* = 1.0, CHCl_3_). m.p. 165–166 °C. ^1^H NMR (600 MHz, CDCl_3_) δ 7.48–7.22 (m, 6H), 7.08 (d, *J* = 7.9 Hz, 2H), 6.75 (d, *J* = 8.1 Hz, 1H), 5.28 (d, *J* = 7.7 Hz, 1H), 5.20–5.01 (m, 3H), 4.77 (q, *J* = 7.6 Hz, 1H), 4.53 (td, *J* = 8.5, 5.6 Hz, 1H), 2.93 (dd, *J* = 7.4, 3.9 Hz, 2H), 2.73 (s, 3H), 1.73–1.46 (m, 4H), 1.38 (s, 9H), 1.01–0.67 (m, 12H). ^13^C NMR (151 MHz, CDCl_3_) δ 172.8, 172.3, 170.3, 155.4, 135.6, 135.0, 131.7, 131.2, 131.1, 128.6, 128.5, 128.3, 128.2, 128.1, 121.1, 80.2, 66.8, 54.9, 51.7, 51.0, 40.4, 38.5, 36.0, 30.7, 28.3, 24.9, 24.5, 23.1, 22.8, 21.9, 21.5. HRMS (ESI) *m*/*z* calcd. for C_34_H_48_BrN_3_O_6_ [M + Na]^+^: 696.2619; found: 696.2614.
*N-Boc-D-Cyclohexyl-Gly-Me-L-Leu-L-Leu-OBn* **5s**. The **5s** was synthesized according to the method of **5** from **4a** (10.0 g, 28.7 mmol, 1.0 equiv) and *N*-Boc *D*-Cyclohexyl-Gly-OH (8.1 g, 31.5 mmol, 1.1 equiv), HOBT (4.2 g, 31.5 mmol, 1.1 equiv), and EDCI (6.0 g, 31.5 mmol, 1.1 equiv). The crude product was purified by silica gel chromatography (hexane/EtOAc = 80:20, *v*/*v*) to obtain **5s** (15.0 g, 25.5 mmol, 89% yield) as a white solid. [α]^25^_D_ = −275.6 (*c* = 1.0, CHCl_3_). m.p. 122–123 °C. ^1^H NMR (600 MHz, DMSO-*d*_6_) δ 8.62 (d, *J* = 7.5 Hz, 1H, minor), 7.75 (d, *J* = 7.6 Hz, 1H, major), 7.34 (m, 5H), 7.01 (d, *J* = 7.0 Hz, 1H), 5.11 (dd, *J* = 14.4, 6.5 Hz, 2H), 5.05 (d, *J* = 12.7 Hz, 1H), 4.48–4.21 (m, 1H), 4.18 (t, *J* = 7.6 Hz, 1H), 2.92 (s, 3H, major), 2.83 (s, 3H, minor), 1.75–1.48 (m, 10H), 1.34 (d, *J* = 18.2 Hz, 10H), 1.25–0.93 (m, 5H), 0.84 (m, 12H). ^13^C NMR (151 MHz, DMSO-*d*_6_) δ 173.2, 172.5, 171.4, 156.5, 136.4, 128.9, 128.9, 128.4, 128.1, 78.7, 66.3, 55.8, 54.3, 51.1, 37.1, 31.3, 29.2, 28.9, 28.6, 26.2, 26.2, 26.0, 24.8, 24.7, 23.8, 23.2, 21.5, 21.4. HRMS (ESI) *m*/*z* calcd. for C_33_H_53_N_3_O_6_ [M + Na]^+^: 610.3827; found: 610.3833.
*N-Boc-D-Phenyl-Gly-Me-L-Leu-L-Leu-OBn* **5t**. The **5t** was synthesized according to the method of **5** from **4a** (10.0 g, 28.7 mmol, 1.0 equiv) and *N*-Boc-*D*-Phenyl-Gly-OH (7.9 g, 31.5 mmol, 1.1 equiv), HOBT (4.2 g, 31.5 mmol, 1.1 equiv), and EDCI (6.0 g, 31.5 mmol, 1.1 equiv). The crude product was purified by silica gel chromatography (hexane/EtOAc = 80:20, *v*/*v*) to obtain **5t** (15.0 g, 25.8 mmol, 90% yield) as a white solid. [α]^25^_D_ = −335.6 (*c* = 1.0, CHCl_3_). m.p. 121–122 °C. ^1^H NMR (600 MHz, DMSO-*d*_6_) δ 8.05 (d, *J* = 7.6 Hz, 1H), 7.57–6.88 (m, 10H), 5.52 (d, *J* = 7.4 Hz, 1H), 5.11 (dd, *J* = 17.0, 8.8 Hz, 2H), 5.07 (s, 1H), 4.33 (dd, *J* = 7.6, 3.5 Hz, 1H), 2.87–2.65 (m, 3H), 1.75–1.38 (m, 5H), 1.36 (d, *J* = 7.6 Hz, 9H), 1.02–0.49 (m, 12H). ^13^C NMR (151 MHz, DMSO-*d*_6_) δ 172.6, 171.5, 171.3, 136.4, 128.9, 128.8, 128.6, 128.5, 128.4, 128.3, 78.9, 66.4, 55.9, 54.4, 51.0, 37.2, 30.9, 28.6, 24.7, 24.5, 23.6, 23.3, 21.6, 21.5. HRMS (ESI) *m*/*z* calcd. for C_33_H_47_N_3_O_6_ [M + Na]^+^: 604.3357; found: 604.3367.
*N-Boc-D-Val-Me-L-Leu-L-Leu-OBn* **5u**. The **5u** was synthesized according to the method of **5** from **4a** (10.0 g, 28.7 mmol, 1.0 equiv) and *N*-Boc-*D*-Val-OH (6.8 g, 31.5 mmol, 1.1 equiv), HOBT (4.2 g, 31.5 mmol, 1.1 equiv), and EDCI (6.0 g, 31.5 mmol, 1.1 equiv). The crude product was purified by silica gel chromatography (hexane/EtOAc = 80:20, *v*/*v*) to obtain **5u** (13.5 g, 24.7 mmol, 86% yield) as a white solid. [α]^25^_D_ = −275.6 (*c* = 1.0, CHCl_3_). m.p. 108–109 °C. ^1^H NMR (600 MHz, DMSO-*d*_6_) δ 8.23 (d, *J* = 7.7 Hz, 1H), 7.44–7.18 (m, 5H), 6.90 (d, *J* = 8.4 Hz, 1H), 5.16 (dd, *J* = 10.7, 4.5 Hz, 1H), 5.13–5.04 (m, 2H), 4.38–4.21 (m, 1H), 4.09 (t, *J* = 8.7 Hz, 1H), 2.99 (s, 3H, major), 2.67 (s, 3H, minor), 2.00–1.79 (m, 1H), 1.69–1.37 (m, 6H), 1.36 (s, 9H), 0.95–0.66 (m, 18H). ^13^C NMR (151 MHz, DMSO-*d*_6_) δ 173.4, 172.6, 171.5, 156.1, 136.4, 128.9, 128.5, 128.3, 78.4, 66.4, 56.3, 53.6, 50.8, 37.5, 31.1, 30.1, 28.6, 28.6, 24.6, 24.5, 23.7, 23.3, 22.0, 21.4, 19.2. HRMS (ESI) *m*/*z* calcd. for C_30_H_49_N_3_O_6_ [M + Na]^+^: 570.3514; found: 570.3524.
*N-Boc-D-Met-Me-L-Leu-L-Leu-OBn* **5v**. The **5v** was synthesized according to the method of **5** from **4a** (10.0 g, 28.7 mmol, 1.0 equiv) and *N*-Boc-*D*-Met-OH (7.8 g, 31.5 mmol, 1.1 equiv), HOBT (4.2 g, 31.5 mmol, 1.1 equiv), and EDCI (6.0 g, 31.5 mmol, 1.1 equiv). The crude product was purified by silica gel chromatography (hexane/EtOAc = 80:20, *v*/*v*) to obtain **5v** (14.0 g, 23.6 mmol, 82% yield) as a white solid. [α]^25^_D_ = −275.6 (*c* = 1.0, CHCl_3_). m.p. 95–96 °C. ^1^H NMR (600 MHz, DMSO-*d*_6_) δ 7.69 (d, *J* = 7.7 Hz, 1H), 7.35 (m, 5H), 7.22 (d, *J* = 6.7 Hz, 1H), 5.12 (d, *J* = 12.6 Hz, 2H), 5.06 (d, *J* = 12.7 Hz, 1H), 4.49 (d, *J* = 7.1 Hz, 1H), 4.32 (d, *J* = 10.3 Hz, 1H), 2.93 (s, 3H, major), 2.78 (s, 3H, minor), 2.47 (d, *J* = 7.4 Hz, 2H), 2.01 (s, 3H), 1.86–1.73 (m, 2H), 1.62 (td, *J* = 35.0, 31.7, 19.2 Hz, 6H), 1.36 (d, J = 14.4 Hz, 9H), 0.85 (m, 12H). ^13^C NMR (151 MHz, DMSO-*d*_6_) δ 173.2, 172.5, 171.2, 156.3, 136.4, 128.9, 128.4, 128.1, 78.8, 66.3, 54.4, 51.1, 50.2, 36.8, 31.1, 30.9, 30.0, 28.6, 28.6, 24.9, 24.7, 23.7, 23.2, 21.7, 21.5, 15.1. HRMS (ESI) *m*/*z* calcd. for C_30_H_49_N_3_O_6_S [M + Na]^+^: 602.3234; found: 602.3244.
*N-Boc-3-Me-D-Phe-Me-L-Leu-L-Leu-OBn* **5w**. The **5w** was synthesized according to the method of **5** from **4a** (10.0 g, 28.7 mmol, 1.0 equiv) and *N*-Boc-3-Me-*D*-Phe-OH (8.8 g, 31.5 mmol, 1.1 equiv), HOBT (4.2 g, 31.5 mmol, 1.1 equiv), and EDCI (6.0 g, 31.5 mmol, 1.1 equiv). The crude product was purified by silica gel chromatography (hexane/EtOAc = 80:20, *v*/*v*) to obtain **5w** (15.0 g, 24.6 mmol, 86% yield) as a white solid. [α]^25^_D_ = −475.4 (*c* = 1.0, CHCl_3_). m.p. 121–122 °C. ^1^H NMR (600 MHz, DMSO-*d*_6_) δ 7.64 (d, *J* = 7.7 Hz, 1H), 7.49–7.22 (m, 5H), 7.24–6.82 (m, 4H), 5.10 (t, *J* = 14.0 Hz, 2H), 5.04 (d, *J* = 12.5 Hz, 1H), 4.65 (t, *J* = 7.1 Hz, 1H), 4.42–4.15 (m, 1H), 2.97–2.73 (m, 2H), 2.67 (s, 3H), 2.25 (s, 3H), 1.82–1.44 (m, 4H), 1.30 (d, *J* = 21.2 Hz, 9H), 1.22 (m, 2H), 0.99–0.56 (m, 12H). ^13^C NMR (151 MHz, DMSO-*d*_6_) δ 173.1, 172.5, 171.2, 156.3, 137.6, 137.2, 136.4, 130.4, 128.9, 128.49, 128.4, 128.1, 127.6, 126.9, 78.9, 66.1, 54.4, 52.2, 51.1, 37.6, 36.7, 30.9, 28.5, 24.7, 24.0, 23.8, 23.3, 21.8, 21.5, 21.4. HRMS (ESI) *m*/*z* calcd. for C_35_H_51_N_3_O_6_ [M + Na]^+^: 632.3671; found: 632.3678.
*N-Boc-4-Me-D-Phe-Me-L-Leu-L-Leu-OBn* **5x**. The **5x** was synthesized according to the method of **5** from **4a** (10.0 g, 28.7 mmol, 1.0 equiv) and *N*-Boc-2-Me-*D*-Phe-OH (8.8 g, 31.5 mmol, 1.1 equiv), HOBT (4.2 g, 31.5 mmol, 1.1 equiv), and EDCI (6.0 g, 31.5 mmol, 1.1 equiv). The crude product was purified by silica gel chromatography (hexane/EtOAc = 80:20, *v*/*v*) to obtain **5x** (14.5 g, 23.8 mmol, 83% yield) as a white solid. [α]^25^_D_ = −356.7 (*c* = 1.0, CHCl_3_). m.p. 161–162 °C. ^1^H NMR (600 MHz, DMSO-*d*_6_) δ 7.61 (d, *J* = 7.6 Hz, 1H), 7.43 (d, *J* = 5.7 Hz, 1H), 7.40–7.23 (m, 4H), 7.09 (m, 4H), 5.28–4.93 (m, 3H), 4.62 (q, *J* = 6.9 Hz, 1H), 4.41–4.24 (m, 1H), 2.97–2.73 (m, 2H), 2.66 (s, 3H), 2.25 (s, 3H), 1.76–1.38 (m, 4H), 1.37–1.07 (m, 9H), 0.97–0.43 (m, 12H). ^13^C NMR (151 MHz, DMSO-*d*_6_) δ 173.1, 172.4, 171.2, 156.3, 136.4, 136.0, 134.1, 129.7, 129.2, 128.9, 128.4, 128.1, 78.9, 66.2, 54.4, 52.2, 51.1, 39.3, 37.2, 36.6, 30.9, 28.5, 24.7, 23.9, 23.7, 23.2, 21.8, 21.4, 21.1. HRMS (ESI) *m*/*z* calcd. for C_35_H_51_N_3_O_6_ [M + Na]^+^: 632.3671; found: 632.3678.

#### 3.1.6. Synthesis of **6**

The product **5** (24.6 mmol, 1.0 equiv) was dissolved in DCM (0.5 M), followed by addition of TFA (0.1 M). The reaction mixture was stirred at room temperature for 1 h. After that, excess TFA and DCM was removed in vacuo to produce **6** as a colorless oil.

#### 3.1.7. Synthesis of **8**

Products **6** (30.7 mmol, 1.1 equiv) and **7** (27.9 mmol, 1.0 equiv) and HOBT (30.7 mmol, 1.1 equiv) were added to dry DCM (0.5 M) at 0 °C. At this temperature, the EDCI (30.7 mmol, 1.1 equiv) was slowly added. The reaction mixture was warmed to 23 °C and stirred for 12 h. After most of the DCM was removed in vacuo, the reaction solution was washed with two 800 mL portions of 0.5 N hydrochloric acid and two 800 mL portions of saturated Na_2_CO_3_ solution. Then, the layers were separated, and the combined organic layer was dried over Na_2_SO_4_. The solvent was removed in vacuo to obtained dipeptide. The crude product was purified by silica gel chromatography (EtOAc/petroleum ether = 70:30, *v*/*v*) to obtain **8** as a white solid. The extra peaks are generated because the nitrogen atom in these compounds is bonded to three different groups, and C–N bond rotation leads to the emergence of rotamers and consequent splitting in the NMR spectra, as referenced in the literature [18].

*N-Boc-Me-D-Leu-D-Leu-D-Leu-Me-D-Leu-D-Leu-OBn* **8a**. The **8a** was synthesized according to the method of **8** from **7d** (10.0 g, 27.9 mmol, 1.0 equiv) and **6a** (14.1 g, 30.7 mmol, 1.1 equiv), HOBT (4.1 g, 30.7 mmol, 1.1 equiv), and EDCI (5.9 g, 30.7 mmol, 1.1 equiv). The crude product was purified by silica gel chromatography (hexane/EtOAc = 70:30, *v*/*v*) to obtain **8a** (19.4 g, 24.2 mmol, 84% yield) as a white solid. [α]^25^_D_ = −175.6 (*c* = 1.0, CHCl_3_). m.p. 138–139 °C. ^1^H NMR (600 MHz, DMSO-*d*_6_) δ 8.24 (d, *J* = 8.0 Hz, 1H), 8.21–8.02 (m, 1H), 7.83 (m, 1H), 7.34 (m, 5H), 5.10 (d, *J* = 8.0 Hz, 3H), 4.82–4.43 (m, 2H), 4.43–4.06 (m, 2H), 2.92 (s, 3H, major), 2.67 (d, *J* = 18.0 Hz, 3H), 2.57 (s, 3H, minor), 1.79–1.19 (m, 25H), 0.84 (m, 30H). ^13^C NMR (151 MHz, DMSO-*d*_6_) δ 172.8, 172.6, 172.1, 171.3, 136.4, 128.9, 128.5, 128.2, 79.4, 66.3, 53.95, 50.8, 47.3, 41.6, 37.8, 37.7, 30.9, 28.5, 24.8, 24.7, 24.5, 23.6, 23.5, 23.4, 23.3, 22.1, 22.0, 22.0, 21.6. HRMS (ESI) *m*/*z* calcd. for C_44_H_75_N_5_O_8_ [M + Na]^+^: 824.5508; found: 824.5507.
*N-Boc-Me-L-Leu-L-Leu-D-Leu-Me-L-Leu-L-Leu-OBn* **8b**. The **8b** was synthesized according to the method of **8** from **7a** (10.0 g, 27.9 mmol, 1.0 equiv) and **6b** (14.1 g, 30.7 mmol, 1.1 equiv), HOBT (4.1 g, 30.7 mmol, 1.1 equiv), and EDCI (5.9 g, 30.7 mmol, 1.1 equiv). The crude product was purified by silica gel chromatography (hexane/EtOAc = 70:30, *v*/*v*) to obtain 8b (19.5 g, 24.3 mmol, 87% yield) as a white solid. [α]^25^_D_ = −164.7 (*c* = 1.0, CHCl_3_). m.p. 138–139 °C. ^1^H NMR (600 MHz, DMSO-*d*_6_) δ 8.13 (m, 1H), 8.01–7.84 (m, 1H), 7.68 (m, 1H), 7.35 (m, 5H), 5.16–5.06 (m, 2H), 4.87 (d, *J* = 198.8 Hz, 1H), 4.68–4.61 (m, 1H), 4.58–4.42 (m, 1H), 4.42–4.23 (m, 2H), 2.96 (s, 3H, major), 2.80 (s, 3H, minor), 2.69 (d, *J* = 5.1 Hz, 3H), 1.82–1.43 (m, 11H), 1.43–1.28 (m, 13H), 0.85 (m, 30H). ^13^C NMR (151 MHz, DMSO-*d*_6_) δ 172.6, 172.6, 172.0, 171.3, 171.0, 155.4, 136.4, 128.8, 128.5, 128.3, 79.4, 66.4, 55.3, 53.8, 50.8, 47.3, 41.3, 37.4, 30.7, 30.1, 28.4, 24.9, 24.7, 24.6, 24.6, 24.5, 23.6, 23.5, 23.3, 22.2, 22.1, 22.0, 21.6, 21.4. HRMS (ESI) *m*/*z* calcd. for C_44_H_75_N_5_O_8_ [M + Na]^+^: 824.5508; found: 824.5507.
*N-Boc-Me-D-Leu-L-Leu-L-Leu-Me-D-Leu-L-Leu-OBn* **8c**. The **8c** was synthesized according to the method of **8** from **7b** (10.0 g, 27.9 mmol, 1.0 equiv) and **6c** (14.1 g, 30.7 mmol, 1.1 equiv), HOBT (4.1 g, 30.7 mmol, 1.1 equiv), and EDCI (5.9 g, 30.7 mmol, 1.1 equiv). The crude product was purified by silica gel chromatography (hexane/EtOAc = 70:30, *v*/*v*) to obtain **8c** (18.7 g, 23.3 mmol, 83% yield) as a white solid. [α]^25^_D_ = −154.2 (*c* = 1.0, CHCl_3_). m.p. 138–139 °C. ^1^H NMR (600 MHz, DMSO-*d*_6_) δ 8.46 (d, *J* = 7.6 Hz, 1H, minor), 8.28–7.98 (m, 1H, major), 7.98–7.50 (m, 2H), 7.34 (m, 5H), 5.10 (d, *J* = 6.8 Hz, 3H), 4.71–4.43 (m, 2H), 4.43–4.25 (m, 2H), 2.84 (s, 3H, major), 2.74 (s, 3H, minor), 2.70 (d, *J* = 15.3 Hz, 3H), 1.84–1.11 (m, 24H), 0.84 (m, 30H). ^13^C NMR (151 MHz, DMSO-*d*_6_) δ 173.0, 172.6, 172.5, 171.1, 136.3, 128.9, 128.5, 128.4, 128.4, 79.41, 66.4, 54.2, 51.0, 50.9, 48.2, 37.8, 30.9, 28.4, 25.0, 24.9, 24.7, 24.6, 23.7, 23.6, 23.5, 23.3, 22.0, 21.9, 21.6, 21.5. HRMS (ESI) *m*/*z* calcd. for C_44_H_75_N_5_O_8_ [M + Na]^+^: 824.5508; found: 824.5507.
*N-Boc-Me-D-Leu-L-Leu-D-Leu-Me-D-Leu-L-Leu-OBn* **8d**. The **8d** was synthesized according to the method of **8** from **7b** (10.0 g, 27.9 mmol, 1.0 equiv) and **6d** (14.1 g, 30.7 mmol, 1.1 equiv), HOBT (4.1 g, 30.7 mmol, 1.1 equiv), and EDCI (5.9 g, 30.7 mmol, 1.1 equiv). The crude product was purified by silica gel chromatography (hexane/EtOAc = 70:30, *v*/*v*) to obtain **8d** (17.9 g, 22.3 mmol, 79% yield) as a white solid. [α]^25^_D_ = −145.6 (*c* = 1.0, CHCl_3_). m.p. 138–139 °C ^1^H NMR (600 MHz, DMSO-*d*_6_) δ 8.54–7.95 (m, 1H), 7.90–7.55 (m, 2H), 7.34 (m, 5H), 5.11 (t, *J* = 9.7 Hz, 3H), 4.77–4.42 (m, 2H), 4.42–4.22 (m, 2H), 2.79 (d, *J* = 64.4 Hz, 3H), 2.70 (d, *J* = 15.3 Hz, 3H), 1.79–1.44 (m, 11H), 1.38 (s, 13H), 0.99–0.66 (m, 30H). ^13^C NMR (151 MHz, DMSO-*d*_6_) δ 172.9, 172.6, 172.5, 171.1, 136.3, 128.9, 128.5, 128.4, 128.4, 79.4, 66.4, 54.2, 50.9, 50.9, 48.2, 39.6, 37.8, 30.9, 28.4, 25.0, 24.9, 24.7, 24.6, 23.7, 23.6, 23.5, 23.3, 22.0, 21.9, 21.6, 21.5. HRMS (ESI) *m*/*z* calcd. for C_44_H_75_N_5_O_8_ [M + Na]^+^: 824.5508; found: 824.5507.
*N-Boc-Me-L-Leu-D-Leu-L-Leu-Me-L-Leu-D-Leu-OBn* **8e**. The **8e** was synthesized according to the method of **8** from **7c** (10.0 g, 27.9 mmol, 1.0 equiv) and **6e** (14.1 g, 30.7 mmol, 1.1 equiv), HOBT (4.1 g, 30.7 mmol, 1.1 equiv), and EDCI (5.9 g, 30.7 mmol, 1.1 equiv). The crude product was purified by silica gel chromatography (hexane/EtOAc = 70:30, *v*/*v*) to obtain **8e** (19.1 g, 23.8 mmol, 84% yield) as a white solid. [α]^25^_D_ = −134.6 (*c* = 1.0, CHCl_3_). m.p. 138–139 °C. ^1^H NMR (600 MHz, DMSO-*d*_6_) δ 8.24 (d, *J* = 8.0 Hz, 1H), 8.19–8.01 (m, 1H), 7.83 (m, 1H), 7.34 (m, 5H), 5.10 (d, *J* = 8.0 Hz, 3H), 4.72 (q, *J* = 7.9 Hz, 1H), 4.68–4.44 (m, 1H), 4.44–4.32 (m, 1H), 4.32–4.23 (m, 1H), 2.92 (s, 3H), 2.69–2.54 (m, 3H), 1.70–1.42 (m, 11H), 1.42–1.24 (m, 12H), 0.84 (m, 30H). ^13^C NMR (151 MHz, DMSO-*d*_6_) δ 172.8, 172.6, 172.1, 171.3, 136.4, 128.9, 128.5, 128.2, 79.4, 66.3, 53.9, 50.8, 47.3, 41.6, 37.8, 37.7, 30.9, 28.5, 24.8, 24.7, 24.6, 23.6, 23.5, 23.4, 23.3, 22.1, 22.0, 22.0, 21.6. HRMS (ESI) *m*/*z* calcd. for C_44_H_75_N_5_O_8_ [M + Na]^+^: 824.5508; found: 824.5507.
*N-Boc-Me-L-Leu-D-Leu-D-Leu-Me-L-Leu-D-Leu-OBn* **8f**. The **8f** was synthesized according to the method of **8** from **4c** (10.0 g, 27.9 mmol, 1.0 equiv) and **6f** (14.1 g, 30.7 mmol, 1.1 equiv), HOBT (4.1 g, 30.7 mmol, 1.1 equiv), and EDCI (5.9 g, 30.7 mmol, 1.1 equiv). The crude product was purified by silica gel chromatography (hexane/EtOAc = 70:30, *v*/*v*) to obtain **8f** (19.7 g, 24.6 mmol, 86% yield) as a white solid. [α]^25^_D_ = −165.6 (*c* = 1.0, CHCl_3_). m.p. 138–139 °C. ^1^H NMR (600 MHz, DMSO-*d*_6_) δ 8.56–8.01 (m, 1H), 7.97–7.53 (m, 2H), 7.35 (dp, *J* = 15.6, 7.2 Hz, 5H), 5.11 (t, *J* = 9.7 Hz, 3H), 4.78–4.43 (m, 2H), 4.43–4.26 (m, 2H), 2.84 (s, 3H, major), 2.74 (s, 3H, minor), 2.70 (d, *J* = 15.3 Hz, 3H), 1.79–1.45 (m, 11H), 1.39 (s, 14H), 0.84 (m, 30H). ^13^C NMR (151 MHz, DMSO-*d*_6_) δ 172.8, 172.6, 172.1, 171.3, 136.4, 128.9, 128.5, 128.2, 79.4, 66.3, 53.95, 50.8, 47.3, 41.6, 37.8, 37.7, 30.9, 28.5, 24.8, 24.7, 24.5, 23.6, 23.5, 23.4, 23.3, 22.1, 22.0, 22.0, 21.6. HRMS (ESI) *m*/*z* calcd. for C_44_H_75_N_5_O_8_ [M + Na]^+^: 824.5508; found: 824.5507.
*N-Boc-Me-D-Leu-D-Leu-L-Leu-Me-D-Leu-D-Leu-OBn* **8g**. The **8g** was synthesized according to the method of **8** from **7d** (10.0 g, 27.9 mmol, 1.0 equiv) and **6g** (14.1 g, 30.7 mmol, 1.1 equiv), HOBT (4.1 g, 30.7 mmol, 1.1 equiv), and EDCI (5.9 g, 30.7 mmol, 1.1 equiv). The crude product was purified by silica gel chromatography (hexane/EtOAc = 70:30, *v*/*v*) to obtain **8g** (18.6 g, 23.2 mmol, 81% yield) as a white solid. [α]^25^_D_ = −154.8 (*c* = 1.0, CHCl_3_). m.p. 138–139 °C. ^1^H NMR (600 MHz, DMSO-*d*_6_) δ 8.23 (d, *J* = 7.7 Hz, 1H), 8.11–7.90 (m, 1H), 7.76 (m, 8.4 Hz, 1H), 7.35 (m, 5H), 5.27–4.95 (m, 3H), 4.73 (m, 1H), 4.68–4.40 (m, 1H), 4.40–4.20 (m, 2H), 2.91 (s, 3H), 2.75–2.58 (m, 3H), 1.71–1.43 (m, 11H), 1.42–1.23 (m, 14H), 0.98–0.67 (m, 30H). ^13^C NMR (151 MHz, DMSO-*d*_6_) δ 172.6, 172.6, 172.0, 171.3, 171.0, 155.4, 136.4, 128.8, 128.5, 128.3, 79.4, 66.4, 55.3, 53.8, 50.8, 47.3, 41.3, 37.4, 30.7, 30.1, 28.4, 24.9, 24.7, 24.6, 24.6, 24.5, 23.6, 23.5, 23.3, 22.2, 22.1, 22.0, 21.6, 21.4. HRMS (ESI) *m*/*z* calcd. for C_44_H_75_N_5_O_8_ [M + Na]^+^: 824.5508; found: 824.5507.
*N-Boc-Me-L-Leu-L-Leu-D-Pro-Me-L-Leu-L-Leu-OBn* **8h**. The **8h** was synthesized according to the method of **8** from **7a** (10.0 g, 27.9 mmol, 1.0 equiv) and **6h** (13.7 g, 30.7 mmol, 1.1 equiv), HOBT (4.1 g, 30.7 mmol, 1.1 equiv), and EDCI (5.9 g, 30.7 mmol, 1.1 equiv). The crude product was purified by silica gel chromatography (hexane/EtOAc = 70:30, *v*/*v*) to obtain **8h** (19.0 g, 24.2 mmol, 88% yield) as a white solid. [α]^25^_D_ = −178.9 (*c* = 1.0, CHCl_3_). m.p. 169–170 °C. ^1^H NMR (600 MHz, DMSO-*d*_6_) δ 8.19 (d, *J* = 7.6 Hz, 1H), 7.90 (m, 1H), 7.39–7.29 (m, 5H), 5.18–5.06 (m, 2H), 5.05–4.81 (m, 1H), 4.77–4.58 (m, 1H), 4.58–4.42 (m, 2H), 4.37–4.20 (m, 1H), 3.71 (d, *J* = 8.3 Hz, 1H), 3.57–3.38 (m, 1H), 2.91 (s, 3H, major), 2.69 (d, *J* = 7.7 Hz, 3H), 2.60 (d, *J* = 5.5 Hz, 1H, minor), 2.19–2.07 (m, 1H), 2.04–1.95 (m, 1H), 1.93–1.82 (m, 1H), 1.75–1.53 (m, 5H), 1.52–1.39 (m, 9H), 1.34–1.21 (m, 10H), 1.02–0.62 (m, 24H). ^13^C NMR (151 MHz, DMSO-*d*_6_) δ 172.6, 172.2, 171.5, 136.4, 128.9, 128.5, 128.3, 66.4, 56.9, 50.8, 47.1, 37.3, 35.0, 34.8, 31.6, 30.3, 28.5, 24.8, 24.7, 24.6, 23.7, 23.7, 23.3, 22.0, 21.5. HRMS (ESI) *m*/*z* calcd. for C_43_H_71_N_5_O_8_ [M + Na]^+^: 808.5195; found: 808.5203.
*N-Boc-Me-L-Leu-L-Leu-D-Phe-Me-L-Leu-L-Leu-OBn* **8i**. The **8i** was synthesized according to the method of **8** from **7a** (10.0 g, 27.9 mmol, 1.0 equiv) and **6i** (15.2 g, 30.7 mmol, 1.1 equiv), HOBT (4.1 g, 30.7 mmol, 1.1 equiv), and EDCI (5.9 g, 30.7 mmol, 1.1 equiv). The crude product was purified by silica gel chromatography (hexane/EtOAc = 70:30, *v*/*v*) to obtain **8i** (20.0 g, 23.9 mmol, 86% yield) as a white solid. [α]^25^_D_ = −155.6 (*c* = 1.0, CHCl_3_). m.p. 141–142 °C. ^1^H NMR (600 MHz, DMSO-*d*_6_) δ 8.37–8.22 (m, 1H), 8.19 (d, *J* = 7.6 Hz, 1H), 7.63 (m 1H), 7.35 (m, 5H), 7.27–7.09 (m, 5H), 5.12 (dd, *J* = 15.7, 11.2 Hz, 2H), 5.08 (d, *J* = 7.2 Hz, 1H), 4.89 (q, *J* = 7.3 Hz, 1H), 4.66–4.38 (m, 1H), 4.38–4.17 (m, 2H), 2.98 (dd, *J* = 13.9, 5.9 Hz, 1H), 2.84 (s, 3H, major), 2.83–2.78 (m, 1H), 2.69 (d, *J* = 12.3 Hz, 3H), 2.55(s, 3H, minor), 1.67–1.44 (m, 7H), 1.44–1.17 (m, 14H), 0.96–0.65 (m, 24H). ^13^C NMR (151 MHz, DMSO-*d*_6_) δ 172.6, 172.0, 171.4, 155.4, 137.9, 136.4, 129.9, 129.7, 128.9, 128.8, 128.5, 128.3, 128.2, 126.7, 79.4, 66.4, 60.2, 53.9, 50.9, 41.6, 37.6, 30.8, 30.2, 28.4, 24.9, 24.8, 24.6, 23.7, 23.2, 22.0, 21.7, 21.6, 21.2. HRMS (ESI) *m*/*z* calcd. for C_47_H_73_N_5_O_8_ [M + Na]^+^: 858.5351; found: 858.5359.
*N-Boc-Me-L-Leu-L-Leu-Gly-Me-L-Leu-L-Leu-OBn* **8j**. The **8j** was synthesized according to the method of **8** from **7a** (10.0 g, 27.9 mmol, 1.0 equiv) and **6j** (12.4 g, 30.7 mmol, 1.1 equiv), HOBT (4.1 g, 30.7 mmol, 1.1 equiv), and EDCI (5.9 g, 30.7 mmol, 1.1 equiv). The crude product was purified by silica gel chromatography (hexane/EtOAc = 70:30, *v*/*v*) to obtain **8j** (17.5 g, 23.5 mmol, 84% yield) as a white solid. [α]^25^_D_ = −167.5 (*c* = 1.0, CHCl_3_). m.p. 137–138 °C. ^1^H NMR (600 MHz, DMSO-*d*_6_) δ 8.60 (d, *J* = 7.6 Hz, 1H, minor), 8.23 (d, *J* = 7.6 Hz, 1H, major), 8.03–7.73 (m, 2H), 7.51–7.16 (m, 5H), 5.17–5.09 (m, 2H), 5.07 (t, *J* = 5.4 Hz, 1H), 4.64 (s, 1H, major), 4.50 (d, *J* = 6.4 Hz, 1H, minor), 4.41–4.24 (m, 2H), 3.97 (d, *J* = 5.1 Hz, 2H), 2.82 (s, 3H, major), 2.78 (s, 3H, minor), 2.71 (s, 3H), 1.57 (m, 10H), 1.40 (d, *J* = 9.9 Hz, 11H), 1.02–0.61 (m, 24H). ^13^C NMR (151 MHz, DMSO-*d*_6_) δ 172.6, 172.5, 171.5, 170.50, 169.5, 136.4, 128.9, 128.5, 128.4, 128.3, 66.4, 54.3, 51.0, 41.4, 41.1, 37.5, 30.2, 28.5, 25.0, 24.8, 24.7, 24.7, 23.7, 23.6, 23.2, 23.2, 23.0, 22.6, 21.9, 21.7, 21.6. HRMS (ESI) *m*/*z* calcd. for C_40_H_67_N_5_O_8_ [M + Na]^+^: 768.4882; found: 768.4892.
*N-Boc-Me-L-Leu-L-Leu-D-Ala-Me-L-Leu-L-Leu-OBn* **8k**. The **8k** was synthesized according to the method of **8** from **7a** (10.0 g, 27.9 mmol, 1.0 equiv) and **6k** (12.9 g, 30.7 mmol, 1.1 equiv), HOBT (4.1 g, 30.7 mmol, 1.1 equiv), and EDCI (5.9 g, 30.7 mmol, 1.1 equiv). The crude product was purified by silica gel chromatography (hexane/EtOAc = 70:30, *v*/*v*) to obtain **8k** (18.0 g, 23.7 mmol, 85% yield) as a white solid. [α]^25^_D_ = −187.5 (*c* = 1.0, CHCl_3_). m.p. 128–129 °C. ^1^H NMR (600 MHz, DMSO-d_6_) δ 8.67–8.04 (m, 1H), 7.99 (dd, J = 29.8, 7.5 Hz, 1H), 7.72 (dd, J = 79.4, 8.9 Hz, 1H), 7.48–7.23 (m, J = 6.6 Hz, 5H), 5.16–5.08 (m, 2H), 5.06 (d, J = 7.9 Hz, 1H), 4.77–4.42 (m, 2H), 4.32 (dp, J = 17.5, 6.0, 5.5 Hz, 2H), 2.91 (s, 3H, major), 2.83 (s, 3H, minor), 2.70 (s, 3H), 1.83–1.47 (m, 9H), 1.40 (d, J = 6.9 Hz, 13H), 1.16 (d, J = 6.7 Hz, 3H), 0.98–0.54 (m, 24H). ^13^C NMR (151 MHz, DMSO-d_6_) δ 173.3, 173.1, 172.6, 171.3, 155.9, 136.4, 128.9, 128.5, 128.4, 128.2, 79.5, 66.5, 66.3, 54.4, 51.5, 51.1, 45.7, 44.7, 37.2, 31.1, 30.2, 28.4, 25.0, 24.9, 24.7, 24.7, 23.7, 23.6, 23.5, 23.4, 23.2, 22.2, 21.8, 21.6, 21.6, 21.3. HRMS (ESI) *m*/*z* calcd. for C_41_H_69_N_5_O_8_ [M + Na]^+^: 782.5039; found: 782.5032.
*N-Boc-Me-L-Leu-L-Leu-O-Me-D-Ser-Me-L-Leu-L-Leu-OBn* **8l**. The **8l** was synthesized according to the method of **8** from **7a** (10.0 g, 27.9 mmol, 1.0 equiv) and **6l** (13.8 g, 30.7 mmol, 1.1 equiv), HOBT (4.1 g, 30.7 mmol, 1.1 equiv), and EDCI (5.9 g, 30.7 mmol, 1.1 equiv). The crude product was purified by silica gel chromatography (hexane/EtOAc = 70:30, *v*/*v*) to obtain **8l** (19.1 g, 24.1 mmol, 86% yield) as a white solid. [α]^25^_D_ = −198.6 (*c* = 1.0, CHCl_3_). m.p. 139–140 °C. ^1^H NMR (600 MHz, DMSO-*d*_6_) δ 8.14 (m, 1H), 7.97 (m, 1H), 7.74 (m, 1H), 7.47–7.16 (m, 5H), 5.11 (m, 2H), 5.06 (d, *J* = 9.6 Hz, 1H), 4.97–4.74 (m, 1H), 4.66–4.45 (m, 1H), 4.42–4.18 (m, 2H), 3.45 (d, *J* = 6.3 Hz, 2H), 3.22 (s, 3H, major), 3.17 (s, 3H, minor), 2.94 (s, 3H, major), 2.80 (s, 3H, minor), 2.70 (d, *J* = 5.2 Hz, 3H), 1.82–1.46 (m, 9H), 1.40 (d, *J* = 7.1 Hz, 13H), 1.00–0.63 (m, 24H). ^13^C NMR (151 MHz, DMSO-*d*_6_) δ 172.6, 172.4, 171.3, 171.0, 170.5, 156.0, 136.4, 128.8, 128.4, 128.4, 128.2, 79.4, 72.7, 72.2, 66.5, 66.3, 58.9, 54.6, 51.5, 51.1, 41.4, 38.9, 37.2, 31.3, 28.4, 24.9, 24.7, 24.6, 23.8, 23.5, 23.2, 21.8, 21.6, 21.5. HRMS (ESI) *m*/*z* calcd. for C_42_H_71_N_5_O_9_ [M + Na]^+^: 812.5144; found: 812.5152.
*N-Boc-Me-L-Leu-L-Leu-O-Me-D-Tyr-Me-L-Leu-L-Leu-OBn* **8m**. The **8m** was synthesized according to the method of **8** from **7a** (10.0 g, 27.9 mmol, 1.0 equiv) and **6m** (16.1 g, 30.7 mmol, 1.1 equiv), HOBT (4.1 g, 30.7 mmol, 1.1 equiv), and EDCI (5.9 g, 30.7 mmol, 1.1 equiv). The crude product was purified by silica gel chromatography (hexane/EtOAc = 70:30, *v*/*v*) to obtain **8m** (23.2 g, 26.6 mmol, 95% yield) as a white solid. [α]^25^_D_ = −156.6 (*c* = 1.0, CHCl_3_). m.p. 141–142 °C. ^1^H NMR (600 MHz, DMSO-*d*_6_) δ 8.60–8.19 (m, 1H), 8.16 (d, *J* = 7.5 Hz, 1H), 7.65 (mm, 1H), 7.35 (m, 5H), 7.12 (d, *J* = 8.1 Hz, 2H), 6.76 (d, *J* = 8.0 Hz, 2H), 5.11 (q, *J* = 12.9 Hz, 3H), 4.83 (t, *J* = 7.4 Hz, 1H), 4.65–4.42 (m, 1H), 4.32 (m, 2H), 3.70 (d, *J* = 8.9 Hz, 3H), 2.91 (dd, *J* = 14.1, 5.8 Hz, 1H), 2.85 (s, 3H, major), 2.79–2.73 (m, 1H), 2.69 (d, *J* = 13.1 Hz, 3H), 2.56 (s, 3H, minor), 1.72–1.13 (m, 21H), 1.03–0.57 (m, 24H). ^13^C NMR (151 MHz, DMSO-*d*_6_) δ 172.6, 172.1, 171.4, 158.3, 136.4, 130.7, 129.7, 128.9, 128.5, 128.3, 128.2, 113.9, 79.4, 66.4, 55.3, 53.9, 50.9, 37.6, 30.9, 30.16, 28.43, 24.92, 24.78, 24.54, 23.67, 23.22, 21.96, 21.64. HRMS (ESI) *m*/*z* calcd. for C_48_H_75_N_5_O_9_ [M + H]^+^: 888.5457; found: 888.5456.
*N-Boc-Me-L-Leu-L-Leu-Methylester-D-Asp-Me-L-Leu-L-Leu-OBn* **8n**. The **8n** was synthesized according to the method of **8** from **7a** (10.0 g, 27.9 mmol, 1.0 equiv) and **6n** (14.6 g, 30.7 mmol, 1.1 equiv), HOBT (4.1 g, 30.7 mmol, 1.1 equiv), and EDCI (5.9 g, 30.7 mmol, 1.1 equiv). The crude product was purified by silica gel chromatography (hexane/EtOAc = 70:30, *v*/*v*) to obtain **8n** (20.0 g, 24.5 mmol, 88% yield) as a white solid. [α]^25^_D_ = −139.8 (*c* = 1.0, CHCl_3_). m.p. 139–140 °C. ^1^H NMR (600 MHz, DMSO-*d*_6_) δ 8.70–8.27 (m, 1H), 7.80 (d, *J* = 8.2 Hz, 2H), 7.49–7.18 (m, 5H), 5.10 (d, *J* = 6.0 Hz, 2H), 5.07 (d, *J* = 9.1 Hz, 2H), 4.69–4.42 (m, 1H), 4.39–4.23 (m, 2H), 3.54 (s, 3H), 2.95 (d, *J* = 8.8 Hz, 3H), 2.91–2.85 (m, 1H), 2.75–2.63 (m, 3H), 2.61–2.52 (m, 1H), 1.73–1.17 (m, 21H), 1.05–0.55 (m, 24H). ^13^C NMR (151 MHz, DMSO-*d*_6_) δ 172.5, 171.8, 171.1, 136.4, 128.9, 128.5, 128.2, 66.4, 54.2, 51.9, 51.0, 41.5, 37.0, 36.6, 30.8, 28.4, 24.9, 24.7, 24.6, 24.4, 23.8, 23.6, 23.2, 21.9, 21.7, 21.5. HRMS (ESI) *m*/*z* calcd. for C_43_H_71_N_5_O_10_ [M + H]^+^: 840.5093; found: 840.5101.
*N-Boc-Me-L-Leu-L-Leu-3-Flu-D-Phe-Me-L-Leu-L-Leu-OBn* **8o**. The **8o** was synthesized according to the method of **8** from **7a** (10.0 g, 27.9 mmol, 1.0 equiv) and 6o (15.7 g, 30.7 mmol, 1.1 equiv), HOBT (4.1 g, 30.7 mmol, 1.1 equiv), and EDCI (5.9 g, 30.7 mmol, 1.1 equiv). The crude product was purified by silica gel chromatography (hexane/EtOAc = 70:30, *v*/*v*) to obtain **8o** (21.2 g, 24.6 mmol, 88% yield) as a white solid. [α]^25^_D_ = −145.6 (*c* = 1.0, CHCl_3_). m.p. 140–141 °C. ^1^H NMR (600 MHz, DMSO-*d*_6_) δ 8.47 (d, *J* = 6.8 Hz, 1H, major), 8.38 (d, *J* = 7.6 Hz, 1H, minor), 7.96 (d, *J* = 7.5 Hz, 1H, major), 7.86 (d, *J* = 7.5 Hz, 1H, minor), 7.63 (d, *J* = 8.7 Hz, 1H, major), 7.46 (d, *J* = 8.9 Hz, 1H, minor), 7.43–7.27 (m, 5H), 7.07 (m, 3H), 5.20–4.98 (m, 3H), 4.99–4.71 (m, 1H), 4.67–4.39 (m, 1H), 4.31 (m, 2H), 2.96 (dd, *J* = 13.4, 7.1 Hz, 1H), 2.88 (t, *J* = 15.5 Hz, 3H), 2.82–2.70 (m, 1H), 2.73–2.58 (m, 3H), 1.89–1.00 (m, 21H), 1.00–0.53 (m, 24H). ^13^C NMR (151 MHz, DMSO-*d*_6_) δ 172.6, 172.1, 171.2, 163.3, 161.7, 136.4, 136.3, 128.8, 128.8, 128.6, 128.4, 128.2, 126.0, 116.8, 116.6, 113.8, 113.7, 79.4, 66.6, 66.3, 60.2, 51.3, 51.1, 41.7, 37.7, 37.2, 36.9, 31.2, 30.2, 28.4, 24.9, 24.7, 24.6, 24.5, 23.7, 23.6, 23.4, 23.4, 23.2, 23.0, 21.9, 21.7, 21.6, 21.5. HRMS (ESI) *m*/*z* calcd. for C_47_H_72_FN_5_O_8_ [M + Na]^+^: 876.5257; found: 876.5254.
*N-Boc-Me-L-Leu-L-Leu-2-Me-D-Phe-Me-L-Leu-L-Leu-OBn* **8q**. The **8q** was synthesized according to the method of **8** from **7a** (10.0 g, 27.9 mmol, 1.0 equiv) and **6q** (15.6 g, 30.7 mmol, 1.1 equiv), HOBT (4.1 g, 30.7 mmol, 1.1 equiv), and EDCI (5.9 g, 30.7 mmol, 1.1 equiv). The crude product was purified by silica gel chromatography (hexane/EtOAc = 70:30, *v*/*v*) to obtain **8q** (21.1 g, 24.6 mmol, 88% yield) as a white solid. [α]^25^_D_ = −156.6 (*c* = 1.0, CHCl_3_). m.p. 161–162 °C. ^1^H NMR (600 MHz, DMSO-*d*_6_) δ 8.59–8.15 (m, 1H), 7.98–7.63 (m, 1H), 7.57–7.25 (m, 5H), 7.22 (d, *J* = 7.5 Hz, 1H), 7.08 (m, 3H), 5.17–5.03 (m, 2H), 5.01 (dt, *J* = 11.2, 5.6 Hz, 1H), 4.92 (d, *J* = 7.5 Hz, 1H), 4.68–4.42 (m, 1H), 4.36 (dt, *J* = 14.1, 6.6 Hz, 1H), 4.32–4.19 (m, 1H), 3.02 (d, *J* = 11.0 Hz, 1H), 2.80 (dd, *J* = 15.1, 8.7 Hz, 1H), 2.75 (s, 1H), 2.68 (d, *J* = 10.4 Hz, 4H), 2.31 (s, 3H, major), 2.23 (s, 3H, minor), 1.80–1.44 (m, 6H), 1.45–1.20 (m, 13H), 0.96–0.59 (m, 24H). ^13^C NMR (151 MHz, DMSO-*d*_6_) δ 172.6, 172.5, 172.5, 171.2, 156.0, 136.4, 135.9, 134.0, 129.7, 129.1, 128.8, 128.8, 128.4, 128.4, 128.1, 79.4, 66.5, 66.2, 51.3, 51.1, 41.8, 41.5, 37.2, 36.9, 31.1, 30.1, 30.0, 28.4, 24.9, 24.7, 24.6, 23.7, 23.6, 23.4, 23.2, 23.1, 21.7, 21.5, 21.1. HRMS (ESI) *m*/*z* calcd. for C_48_H_75_N_5_O_8_ [M + Na]^+^: 872.5508; found: 872.5514.
*N-Boc-Me-L-Leu-L-Leu-3-Chl-D-Phe-Me-L-Leu-L-Leu-OBn* **8p**. The **8p** was synthesized according to the method of **8** from **7a** (10.0 g, 27.9 mmol, 1.0 equiv) and **6p** (16.2 g, 30.7 mmol, 1.1 equiv), HOBT (4.1 g, 30.7 mmol, 1.1 equiv), and EDCI (5.9 g, 30.7 mmol, 1.1 equiv). The crude product was purified by silica gel chromatography (hexane/EtOAc = 70:30, *v*/*v*) to obtain **8p** (22.2 g, 25.3 mmol, 90% yield) as a white solid. [α]^25^_D_ = −116.6 (*c* = 1.0, CHCl_3_). m.p. 161–162 °C. ^1^H NMR (600 MHz, DMSO-*d*_6_) δ 8.45 (d, *J* = 6.7 Hz, 1H, major), 8.35 (d, *J* = 7.5 Hz, 1H, minor), 7.94 (d, *J* = 7.5 Hz, 1H, major), 7.85 (d, *J* = 7.5 Hz, 1H, minor), 7.64 (d, *J* = 8.5 Hz, 1H, major), 7.48 (d, *J* = 9.0 Hz, 1H, minor), 7.41–7.11 (m, 8H), 5.10 (m, 2H), 4.99 (m, 1H), 4.91 (d, *J* = 7.4 Hz, 1H), 4.67–4.40 (m, 1H), 4.41–4.19 (m, 2H), 3.00–2.69 (m, 5H), 2.68 (d, *J* = 9.9 Hz, 3H), 1.86–1.02 (m, 20H), 1.02–0.17 (m, 24H). ^13^C NMR (151 MHz, DMSO-*d*_6_) δ 172.6, 172.5, 172.2, 170.6, 155.3, 136.4, 131.7, 128.9, 128.8, 128.5, 128.4, 128.2, 79.5, 79.4, 66.6, 66.3, 51.3, 51.1, 50.8, 41.7, 37.7, 37.2, 37.0, 31.2, 30.2, 28.4, 24.9, 24.7, 24.5, 24.3, 23.7, 23.5, 23.4, 23.2, 23.1, 22.0, 22.0, 21.7, 21.5, 21.1. HRMS (ESI) *m*/*z* calcd. for C_47_H_72_ClN_5_O_8_ [M + Na]^+^: 892.4962; found: 892.4966.
*N-Boc-Me-L-Leu-L-Leu-3-Bro-D-Phe-Me-L-Leu-L-Leu-OBn* **8r**. The **8r** was synthesized according to the method of **8** from **7a** (10.0 g, 27.9 mmol, 1.0 equiv) and **6r** (17.6 g, 30.7 mmol, 1.1 equiv), HOBT (4.1 g, 30.7 mmol, 1.1 equiv), and EDCI (5.9 g, 30.7 mmol, 1.1 equiv). The crude product was purified by silica gel chromatography (hexane/EtOAc = 70:30, *v*/*v*) to obtain **8r** (24.0 g, 26.3 mmol, 94% yield) as a white solid. [α]^25^_D_ = −175.6 (*c* = 1.0, CHCl_3_). m.p. 171–172 °C. ^1^H NMR (600 MHz, DMSO-*d*_6_) δ 8.44 (d, *J* = 6.8 Hz, 1H, major), 8.34 (d, *J* = 7.6 Hz, 1H, minor), 7.95 (d, *J* = 7.5 Hz, 1H, major), 7.86 (d, *J* = 7.4 Hz, 1H, minor), 7.64 (d, *J* = 8.6 Hz, 1H, major), 7.48 (d, *J* = 8.8 Hz, 1H, minor), 7.45 (m, 2H), 7.35 (m, 5H), 7.19 (m, 2H), 5.24–5.01 (m, 2H), 5.00–4.68 (m, 2H), 4.64–4.38 (m, 1H), 4.38–4.07 (m, 2H), 2.86 (m, 4H), 2.77 (d, *J* = 13.3 Hz, 1H), 2.67 (d, *J* = 8.7 Hz, 3H), 1.82–1.00 (m, 20H), 1.01–0.49 (m, 24H). ^13^C NMR (151 MHz, DMSO-*d*_6_) δ 173.0, 172.6, 171.1, 170.6, 156.0, 136.4, 132.1, 131.4, 131.0, 128.8, 128.8, 128.5, 128.4, 128.4, 128.1, 120.3, 79.4, 66.6, 66.2, 60.2, 51.4, 51.1, 50.8, 41.8, 41.5, 37.2, 36.9, 31.2, 28.4, 24.9, 24.7, 24.6, 23.7, 23.5, 23.4, 23.2, 23.1, 22.1, 22.0, 21.7, 21.7, 21.5, 21.1. HRMS (ESI) *m*/*z* calcd. for C_47_H_72_BrN_5_O_8_ [M + H]^+^: 936.4456; found: 936.4454.
*N-Boc-Me-L-Leu-L-Leu-D-Cyclohexyl-Gly-Me-L-Leu-L-Leu-OBn* **8s**. The **8s** was synthesized according to the method of **8** from **7a** (10.0 g, 27.9 mmol, 1.0 equiv) and **6s** (14.9 g, 30.7 mmol, 1.1 equiv), HOBT (4.1 g, 30.7 mmol, 1.1 equiv), and EDCI (5.9 g, 30.7 mmol, 1.1 equiv). The crude product was purified by silica gel chromatography (hexane/EtOAc = 70:30, *v*/*v*) to obtain **8s** (21.1 g, 25.4 mmol, 91% yield) as a white solid. [α]^25^_D_ = −145.6 (*c* = 1.0, CHCl_3_). m.p. 140–141 °C. ^1^H NMR (600 MHz, DMSO-*d*_6_) δ 8.09 (d, *J* = 7.0 Hz, 1H, major), 7.98 (d, *J* = 7.5 Hz, 1H, minor), 7.90 (d, *J* = 8.8 Hz, 1H, major), 7.84 (dd, *J* = 14.0, 8.4 Hz, 1H, minor), 7.75–7.65 (m, 1H), 7.35 (m, 5H), 5.18–5.08 (m, 2H), 5.06 (d, *J* = 12.9 Hz, 1H), 4.62 (d, *J* = 8.1 Hz, 1H), 4.52–4.20 (m, 3H), 2.97 (s, 3H, major), 2.86 (s, 3H, minor), 2.69 (d, *J* = 7.4 Hz, 3H), 1.89–0.94 (m, 32H), 0.95–0.58 (m, 24H). ^13^C NMR (151 MHz, DMSO-*d*_6_) δ 172.7, 172.6, 172.5, 172.1, 170.7, 155.9, 136.4, 136.3, 128.9, 128.9, 128.6, 128.5, 128.2, 79.4, 66.5, 66.2, 54.5, 51.1, 41.4, 38.0, 37.1, 31.5, 29.2, 28.4, 26.2, 26.0, 26.0, 25.0, 24.9, 24.8, 24.6, 23.9, 23.5, 23.3, 23.3, 22.3, 21.6, 21.5, 21.4, 21.3. HRMS (ESI) *m*/*z* calcd. for C_46_H_77_N_5_O_8_ [M + Na]^+^: 850.5670; found:850.5675.
*N-Boc-Me-L-Leu-L-Leu-D-Phenyl-Gly-Me-L-Leu-L-Leu-OBn* **8t**. The **8t** was synthesized according to the method of **8** from **7a** (10.0 g, 27.9 mmol, 1.0 equiv) and **6t** (14.8 g, 30.7 mmol, 1.1 equiv), HOBT (4.1 g, 30.7 mmol, 1.1 equiv), and EDCI (5.9 g, 30.7 mmol, 1.1 equiv). The crude product was purified by silica gel chromatography (hexane/EtOAc = 70:30, *v*/*v*) to obtain **8t** (20.0 g, 24.3 mmol, 87% yield) as a white solid. [α]^25^_D_ = −167.5 (*c* = 1.0, CHCl_3_). m.p. 140–141 °C. ^1^H NMR (600 MHz, DMSO-*d*_6_) δ 8.41 (d, *J* = 7.3 Hz, 1H), 8.19 (d, *J* = 7.5 Hz, 1H), 8.09–7.76 (m, 1H), 7.46–7.14 (m, 9H), 5.77 (q, *J* = 7.4, 6.8 Hz, 1H), 5.13 (d, *J* = 12.2 Hz, 2H), 5.08 (d, *J* = 12.7 Hz, 1H), 4.75–4.43 (m, 1H), 4.43–4.13 (m, 2H), 2.88 (s, 3H, minor), 2.75 (s, 3H, major), 2.69 (s, 3H), 1.84–1.07 (m, 20H), 1.09–0.24 (m, 24H). ^13^C NMR (151 MHz, DMSO-*d*_6_) δ 172.6, 171.7, 171.4, 171.0, 136.4, 128.9, 128.8, 128.5, 128.4, 128.3, 79.5, 66.4, 54.4, 51.5, 51.0, 40.8, 37.4, 31.1, 30.2, 28.4, 25.0, 24.7, 24.6, 24.4, 23.6, 23.5, 23.2, 22.2, 21.9, 21.6, 21.3. HRMS (ESI) *m*/*z* calcd. for C_46_H_71_N_5_O_8_ [M + Na]^+^: 844.5195; found: 844.5204.
*N-Boc-Me-L-Leu-L-Leu-D-Val-Me-L-Leu-L-Leu-OBn* **8u**. The **8u** was synthesized according to the method of **8** from **7a** (10.0 g, 27.9 mmol, 1.0 equiv) and **6u** (13.7 g, 30.7 mmol, 1.1 equiv), HOBT (4.1 g, 30.7 mmol, 1.1 equiv), and EDCI (5.9 g, 30.7 mmol, 1.1 equiv). The crude product was purified by silica gel chromatography (hexane/EtOAc = 70:30, *v*/*v*) to obtain **8u** (19.1 g, 24.1 mmol, 86% yield) as a white solid. [α]^25^_D_ = −175.6 (*c* = 1.0, CHCl_3_). m.p. 128–129 °C. ^1^H NMR (600 MHz, DMSO-*d*_6_) δ 8.26 (d, *J* = 7.8 Hz, 1H), 7.99 (m, 1H), 7.85–7.63 (m, 1H), 7.49–7.26 (m, 5H), 5.14 (d, *J* = 6.1 Hz, 1H), 5.13–5.05 (m, 2H), 4.49 (t, *J* = 8.7 Hz, 2H), 4.31 (m, 2H), 2.96 (s, 3H), 2.70 (d, *J* = 4.2 Hz, 3H), 1.97 (q, *J* = 7.2 Hz, 1H), 1.68–1.43 (m, 9H), 1.43–1.36 (m, 10H), 1.35–1.22 (m, 3H), 0.85 (m, 30H). ^13^C NMR (151 MHz, DMSO-*d*_6_) δ 172.5, 172.4, 172.2, 171.3, 136.4, 128.8, 128.5, 128.3, 79.4, 66.4, 54.3, 53.6, 50.8, 41.3, 37.5, 31.0, 30.2, 28.5, 24.9, 24.7, 24.6, 23.5, 23.3, 22.1, 21.9, 21.4, 19.4. HRMS (ESI) *m*/*z* calcd. for C_43_H_73_N_5_O_8_ [M + Na]^+^: 810.5351; found: 810.5359.
*N-Boc-Me-L-Leu-L-Leu-D-Met-Me-L-Leu-L-Leu-OBn* **8v**. The **8v** was synthesized according to the method of **8** from **7a** (10.0 g, 27.9 mmol, 1.0 equiv) and **6v** (14.7 g, 30.7 mmol, 1.1 equiv), HOBT (4.1 g, 30.7 mmol, 1.1 equiv), and EDCI (5.9 g, 30.7 mmol, 1.1 equiv). The crude product was purified by silica gel chromatography (hexane/EtOAc = 70:30, *v*/*v*) to obtain **8v** (22.0 g, 26.9 mmol, 96% yield) as a white solid. [α]^25^_D_ = −139.8 (*c* = 1.0, CHCl_3_). m.p. 118–119 °C. ^1^H NMR (600 MHz, DMSO-*d*_6_) δ 8.21 (d, *J* = 7.2 Hz, 1H, major), 8.17 (d, *J* = 7.4 Hz, 1H, minor), 7.99 (d, *J* = 7.3 Hz, 1H, major), 7.93 (d, *J* = 7.6 Hz, 1H, minor), 7.75 (d, *J* = 8.5 Hz, 1H, major), 7.63 (d, *J* = 8.7 Hz, 1H, minor), 7.48–6.87 (m, 5H), 5.17–5.07 (m, 2H), 5.07–5.00 (m, 1H), 4.79 (m, 1H), 4.66–4.39 (m, 1H), 4.39–4.17 (m, 2H), 2.96 (s, 3H, major), 2.81 (s, 3H, minor), 2.70 (d, *J* = 4.6 Hz, 3H), 2.42 (q, *J* = 6.2, 4.6 Hz, 2H), 2.04 (s, 3H, major), 1.98 (s, 3H, minor), 1.81–1.14 (m, 22H), 1.10–0.56 (m, 24H). ^13^C NMR (151 MHz, DMSO-*d*_6_) δ 172.6, 172.4, 171.3, 170.8, 136.4, 128.9, 128.5, 128.4, 128.2, 79.5, 66.5, 66.3, 54.5, 51.7, 51.1, 41.5, 37.9, 37.2, 31.2, 30.1, 29.7, 28.4, 24.9, 24.7, 23.7, 23.5, 23.4, 23.2, 22.2, 21.8, 21.6, 21.6, 21.5, 15.1. HRMS (ESI) *m*/*z* calcd. for C_43_H_73_N_5_O_8_S [M + Na]^+^: 842.5072; found: 842.5082.
*N-Boc-Me-L-Leu-L-Leu-3-Me-D-Phe-Me-L-Leu-L-Leu-OBn* **8w**. The **8w** was synthesized according to the method of **8** from **7a** (10.0 g, 27.9 mmol, 1.0 equiv) and **6w** (15.6 g, 30.7 mmol, 1.1 equiv), HOBT (4.1 g, 30.7 mmol, 1.1 equiv), and EDCI (5.9 g, 30.7 mmol, 1.1 equiv). The crude product was purified by silica gel chromatography (hexane/EtOAc = 70:30, *v*/*v*) to obtain **8w** (20.2 g, 23.6 mmol, 85% yield) as a white solid. [α]^25^_D_ = −178.6 (*c* = 1.0, CHCl_3_). m.p. 122–123 °C. ^1^H NMR (600 MHz, DMSO-*d*_6_) δ 8.47 (d, *J* = 6.6 Hz, 1H, major), 8.36 (d, *J* = 7.3 Hz, 1H, minor), 7.95 (d, *J* = 7.4 Hz, 1H, major), 7.86 (d, *J* = 7.5 Hz, 1H, minor), 7.65 (d, *J* = 8.7 Hz, 1H, major), 7.47 (d, *J* = 9.2 Hz, 1H, minor), 7.35 (m, 5H), 7.24–6.88 (m, 4H), 5.23–5.04 (m, 2H), 5.01 (dd, *J* = 11.3, 4.8 Hz, 1H), 4.95–4.68 (m, 1H), 4.68–4.41 (m, 1H), 4.41–4.12 (m, 2H), 3.02–2.56 (m, 8H), 2.25 (d, *J* = 8.3 Hz, 3H), 1.81–1.56 (m, 3H), 1.45 (m, 17H), 1.19–0.95 (m, 1H), 0.96–0.45 (m, 24H). ^13^C NMR (151 MHz, DMSO-*d*_6_) δ 172.9, 172.6, 172.4, 171.9, 170.6, 156.0, 137.6, 137.1, 136.4, 130.4, 128.9, 128.9, 128.5, 128.2, 127.6, 126.9, 79.4, 66.6, 66.2, 57.0, 55.6, 54.4, 51.1, 41.8, 41.6, 37.7, 36.9, 31.1, 30.2, 30.1, 28.4, 24.9, 24.7, 24.6, 24.2, 23.7, 23.6, 23.5, 23.2, 23.1, 22.0, 21.9, 21.7, 21.7, 21.5, 21.5, 21.2. HRMS (ESI) *m*/*z* calcd. for C_48_H_75_N_5_O_8_ [M + Na]^+^: 872.5508; found: 872.5517.
*N-Boc-Me-L-Leu-L-Leu-4-Me-D-Phe-Me-L-Leu-L-Leu-OBn* **8x**. The **8x** was synthesized according to the method of **8** from **7a** (10.0 g, 27.9 mmol, 1.0 equiv) and **6x** (15.6 g, 30.7 mmol, 1.1 equiv), HOBT (4.1 g, 30.7 mmol, 1.1 equiv), and EDCI (5.9 g, 30.7 mmol, 1.1 equiv). The crude product was purified by silica gel chromatography (hexane/EtOAc = 70:30, *v*/*v*) to obtain **8x** (21.0 g, 24.6 mmol, 88% yield) as a white solid. [α]^25^_D_ = −156.7 (*c* = 1.0, CHCl_3_). m.p. 142–143 °C. ^1^H NMR (600 MHz, DMSO-*d*_6_) δ8.47 (d, *J* = 6.6 Hz, 1H, major), 8.36 (d, *J* = 7.3 Hz, 1H, minor), 7.95 (d, *J* = 7.4 Hz, 1H, major), 7.86 (d, *J* = 7.5 Hz, 1H, minor), 7.65 (d, *J* = 8.7 Hz, 1H, major), 7.47 (d, *J* = 9.2 Hz, 1H, minor), 7.35 (m, 5H), 7.24–6.88 (m, 4H), 5.23–5.04 (m, 2H), 5.01 (dd, *J* = 11.3, 4.8 Hz, 1H), 4.95–4.68 (m, 1H), 4.68–4.41 (m, 1H), 4.41–4.12 (m, 2H), 3.02–2.56 (m, 8H), 2.25 (d, *J* = 8.3 Hz, 3H), 1.81–1.56 (m, 3H), 1.45 (m, 17H), 1.19–0.95 (m, 1H), 0.96–0.45 (m, 24H). ^13^C NMR (151 MHz, DMSO-*d*_6_) δ 172.9, 172.6, 172.4, 171.9, 170.6, 156.0, 137.6, 137.1, 136.4, 130.38, 128.9, 128.9, 128.5, 128.2, 127.6, 126.9, 79.4, 66.6, 66.3, 57.0, 55.6, 54.4, 51.1, 41.8, 41.6, 37.7, 36.9, 31.1, 30.2, 30.1, 28.4, 24.9, 24.7, 24.6, 24.2, 23.7, 23.6, 23.5, 23.3, 23.1, 22.0, 21.9, 21.7, 21.7, 21.5, 21.5, 21.2. HRMS (ESI) *m*/*z* calcd. for C_48_H_75_N_5_O_8_ [M + Na]^+^: 872.5508; found: 872.5517.

#### 3.1.8. Synthesis of **9**

The product **8** (33.4 mmol, 1.0 equiv) was dissolved in DCM (0.5 M), followed by the addition of TFA (0.12 M). The reaction mixture was stirred at room temperature for 1 h. After that, excess TFA and DCM was removed in vacuo to produce **9** as a colorless oil.

#### 3.1.9. Synthesis of **10**

Catalytic hydrogenation of **9** (33.4 mmol, 1.0 equiv) over 10% Pd/C (4.7 mmol, 0.1 equiv) was carried out at atmospheric pressure in methanol for 2 h. The mixture was filtered and concentrated in vacuo to obtain **10**.

#### 3.1.10. Synthesis of **Gala**

Route A using HATU conditions: After dissolving the resultant white solid **10** (2.5 mmol, 1 equiv) in THF (0.5 M), the solution was added to a suspension of 2-(7-Azabenzotriazol-1-yl)-*N*,*N*,*N*’,*N*’-tetramethyluronium hexafluorophosphate HATU (37.2 mmol, 15 equiv) and DIPEA (74.4 mmol, 30 equiv) in THF (1 mM) using a micro syringe pump (1.0 mL/h). The result was agitated for 24 h at room temperature. Lower pressure was used to concentrate the filtrate. Saturated NaHCO_3_, brine, 1% HCl, and ethyl acetate were used to wash the residue. Under lower pressure, the organic phase was dried (Na_2_SO_4_) and concentrated. By using column chromatography on silica gel (hexane/EtOAc = 60:40, *v*/*v*) to purify the crude product, **Gala** was obtained as an off-white solid [21].

Route B using T_3_P^®^ conditions: A solution of **10** (0.348 mmol, 1 equiv) and pyridine (0.348 mmol, 1 equiv) in ethyl acetate (0.5 M) was stirred at 0 °C. *N*-Propanephosphonic acid anhydride (T_3_P^®^, 50% solution in ethyl acetate, 0.871 mmol, 2.5 equiv) was added dropwise. The reaction mixture was allowed to warm to room temperature and was stirred for 16 h. The reaction mixture was concentrated under reduced pressure and diluted with water, and the product was extracted with DCM. The organic solution was dried over Na_2_SO_4_ and concentrated. Chromatography on silica by using hexane/EtOAc = 60:40 *v*/*v* as the eluant obtained **Gala** [22].

Route C using DPPA conditions: To a 0 °C stirred solution of **10** (1 mmol, 1 equiv) in dry DMF (1 mM) was added *N,N*-diisopropylethylamine (6 mmol, 6 equiv) dropwise over 1 min followed by diphenyl phosphorazidate (DPPA) (4 mmol, 4 equiv); then, it was slowly brought to room temperature and stirred until the complete consumption of starting material; then, DMF was removed under reduced pressure. The resulting viscous solution was diluted with water and thoroughly extracted with EtOAc. The combined organic extracts were washed with 1N HCl, saturated with aqueous NaHCO_3_, dried over Na_2_SO_4_, and concentrated to obtain a residue, which was purified by silica gel flash column chromatograph [23].

Route D using FDPP conditions: The crude deprotected product **10** (0.221 mmol, 1 equiv) was suspended in anhydrous CH_3_CN (1 mM) and cooled to 0 °C, and pentafluorophenyldiphenylphosphinate (FDPP) reagent (0.221 mmol, 1 equiv) was added in one portion and stirred for 20 min at the same temperature. Then, DIPEA (0.442 mmol, 2 equiv) was slowly added over a period of 30 min. After the addition was over, the mixture was allowed to warm to room temperature and stirred for 26 h. The mixture was concentrated in vacuo, and the residue was purified by silica gel (100–200 mesh) flash column chromatograph to obtain **Gala** [24].

Route E using PyBOP conditions: **10** (0.053 mmol, 2 equiv) was dissolved in MeCN (4.3 mM) and was added dropwise over a 10 h period to a stirred solution of PyBOP (0.053 mmol, 2 equiv) and DMAP (0.079 mmol, 3 equiv) in MeCN (1 mM). The mixture was stirred for an additional 3 h. The solvent was removed under reduced pressure, and the residue was suspended in H_2_O and extracted with EtOAc. The organic layers were combined, washed with saturated Na_2_CO_3_ solution, brined, dried over Na_2_SO_4_, and concentrated under reduced pressure. The residue was purified by column chromatography (hexane/EtOAc = 60:40, *v*/*v*) [25].

*Cyclo(Me-D-Leu-D-Leu-D-Leu-Me-D-Leu-D-Leu)* **Gala01**. The product **10a** (134 mg, 0.22 mmol, 1 equiv) was dissolved in MeCN (4.3 mM) and reacted according to route E for macrolactamization. PyBOP (229 mg, 0.44 mmol, 2 equiv), DMAP (80 mg, 0.66 mmol, 3 equiv). The crude residue was purified by column chromatography (hexane/EtOAc = 60:40, *v*/*v*), obtaining compound **Gala01** (62 mg, 0.10 mmol, 48% yield) as a white solid. [α]^25^_D_ = −5.6 (*c* = 1.0, CHCl_3_). m.p. 200–201 °C. ^1^H NMR (600 MHz, DMSO-*d*_6_) δ 7.62 (d, *J* = 8.0 Hz, 1H), 7.38 (d, *J* = 8.2 Hz, 1H), 7.19 (d, *J* = 8.8 Hz, 1H), 5.10 (t, *J* = 7.6 Hz, 1H), 4.74 (dq, *J* = 35.7, 7.5 Hz, 2H), 4.48 (dd, *J* = 10.1, 5.2 Hz, 1H), 4.15 (dt, *J* = 13.2, 6.6 Hz, 1H), 2.98 (s, 3H), 2.72 (s, 3H), 1.69–1.14 (m, 15H), 0.87 (m, 30H). ^13^C NMR (151 MHz, DMSO-*d*_6_) δ 174.5, 172.2, 171.3, 170.8, 170.5, 57.9, 53.6, 51.3, 48.3, 47.7, 41.34, 41.7, 38.7, 37.2, 35.1, 31.3, 29.9, 25.2, 24.9, 24.8, 24.8, 24.7, 23.5, 23.4, 23.1, 23.0, 23.0, 22.0, 22.0, 21.9, 21.7. HRMS (ESI) *m*/*z* calcd. for C_32_H_59_N_5_O_5_ [M + Na]^+^: 616.4408; found: 616.4415.
*Cyclo(Me-L-Leu-L-Leu-D-Leu-Me-L-Leu-L-Leu)* **Gala02**. The product **10b** (134 mg, 0.22 mmol, 1 equiv) was dissolved in MeCN (4.3 mM) and reacted according to route E for macrolactamization. PyBOP (229 mg, 0.44 mmol, 2 equiv), DMAP (80 mg, 0.66 mmol, 3 equiv). The crude residue was purified by column chromatography (hexane/EtOAc = 60:40, *v*/*v*), obtaining compound **Gala02** (60 mg, 0.10 mmol, 45% yield) as a white solid. [α]^25^_D_ = −13.6 (*c* = 1.0, CHCl_3_). m.p. 200–201 °C. ^1^H NMR (600 MHz, DMSO-*d*_6_) δ 7.63 (d, *J* = 8.1 Hz, 1H), 7.39 (d, *J* = 8.2 Hz, 1H), 7.20 (d, *J* = 8.8 Hz, 1H), 5.11 (dd, *J* = 9.5, 6.2 Hz, 1H), 4.77 (td, *J* = 8.3, 5.6 Hz, 1H), 4.71 (q, *J* = 7.6 Hz, 1H), 4.49 (dd, *J* = 10.4, 5.2 Hz, 1H), 4.15 (ddd, *J* = 10.4, 8.1, 5.2 Hz, 1H), 2.98 (s, 3H), 2.73 (s, 3H), 1.75–1.32 (m, 15H), 1.02–0.61 (m, 30H). ^13^C NMR (151 MHz, DMSO-*d*_6_) δ 174.5, 172.3, 171.4, 170.8, 170.6, 57.9, 53.7, 51.4, 48.3, 47.7, 41.4, 41.1, 38.7, 37.2, 35.2, 31.3, 29.9, 25.3, 24.9, 24.9, 24.8, 24.8, 23.5, 23.4, 23.2, 23.0, 23.0, 22.1, 22.0, 21.9, 21.7. HRMS (ESI) *m*/*z* calcd. for C_32_H_59_N_5_O_5_ [M + Na]^+^: 616.4408; found: 616.4415.
*Cyclo(Me-D-Leu-L-Leu-L-Leu-Me-D-Leu-L-Leu)* **Gala03**. The product **10c** (134 mg, 0.22 mmol, 1 equiv) was dissolved in MeCN (4.3 mM) and reacted according to route E for macrolactamization. PyBOP (229 mg, 0.44 mmol, 2 equiv), DMAP (80 mg, 0.66 mmol, 3 equiv). The crude residue was purified by column chromatography (hexane/EtOAc = 60:40, *v*/*v*), obtaining compound **Gala03** (63 mg, 0.11 mmol, 48% yield) as a white solid. [α]^25^_D_ = −75.6 (*c* = 1.0, CHCl_3_). m.p. 200–201 °C. ^1^H NMR (600 MHz, DMSO-*d*_6_) δ 8.29 (d, *J* = 8.7 Hz, 1H), 7.45 (d, *J* = 8.9 Hz, 1H), 7.01 (d, *J* = 9.4 Hz, 1H), 5.06 (t, *J* = 7.8 Hz, 1H), 4.80 (td, *J* = 9.1, 5.5 Hz, 1H), 4.73–4.50 (m, 2H), 4.16 (td, *J* = 9.5, 5.1 Hz, 1H), 2.95 (s, 3H), 2.54 (s, 3H), 1.75–1.31 (m, 15H), 1.09–0.55 (m, 30H). ^13^C NMR (151 MHz, DMSO-*d*_6_) δ 173.7, 171.7, 171.5, 171.2, 169.7, 55.9, 53.5, 52.3, 47.6, 47.5, 41.6, 41.3, 40.9, 37.1, 34.9, 30.9, 29.3, 25.0, 25.0, 24.8, 24.8, 24.7, 23.4, 23.3, 23.2, 23.0, 22.9, 22.7, 22.3, 22.1, 21.4. HRMS (ESI) *m*/*z* calcd. for C_32_H_59_N_5_O_5_ [M + Na]^+^: 616.4408; found: 616.4415.
*Cyclo(Me-D-Leu-L-Leu-D-Leu-Me-D-Leu-L-Leu)* **Gala04**. The product **10d** (134 mg, 0.22 mmol, 1 equiv) was dissolved in MeCN (4.3 mM) and reacted according to route E for macrolactamization. PyBOP (229 mg, 0.44 mmol, 2 equiv), DMAP (80 mg, 0.66 mmol, 3 equiv). The crude residue was purified by column chromatography (hexane/EtOAc = 60:40, *v*/*v*), obtaining compound **Gala04** (59 mg, 0.11 mmol, 0.45% yield) as a white solid. [α]^25^_D_ = −55.6 (*c* = 1.0, CHCl_3_). m.p. 200–201 °C. ^1^H NMR (600 MHz, DMSO-*d*_6_) δ 8.28 (d, *J* = 8.7 Hz, 1H), 7.44 (d, *J* = 8.9 Hz, 1H), 7.00 (d, *J* = 9.4 Hz, 1H), 5.05 (t, *J* = 7.8 Hz, 1H), 4.79 (q, *J* = 3.6 Hz, 1H), 4.73–4.45 (m, 2H), 4.15 (q, *J* = 4.3 Hz, 1H), 2.94 (s, 3H), 2.53 (s, 3H), 1.76–1.26 (m, 15H), 1.02–0.65 (m, 30H). ^13^C NMR (151 MHz, DMSO-*d*_6_) δ 173.7, 171.7, 171.5, 171.2, 169.7, 55.9, 53.5, 52.3, 47.6, 47.5, 41.6, 41.3, 40.9, 37.1, 34.9, 30.9, 29.3, 25.0, 24.9, 24.8, 24.7, 24.6, 23.4, 23.3, 23.2, 23.0, 22.9, 22.7, 22.3, 22.1, 21.4. HRMS (ESI) *m*/*z* calcd. for C_32_H_59_N_5_O_5_ [M + Na]^+^: 616.4408; found: 616.4415.
*Cyclo(Me-L-Leu-D-Leu-L-Leu-Me-L-Leu-D-Leu)* **Gala05**. The product **10e** (134 mg, 0.22 mmol, 1 equiv) was dissolved in MeCN (4.3 mM) and reacted according to route E for macrolactamization. PyBOP (229 mg, 0.44 mmol, 2 equiv), DMAP (80 mg, 0.66 mmol, 3 equiv). The crude residue was purified by column chromatography (hexane/EtOAc = 60:40, *v*/*v*), obtaining compound **Gala05** (60 mg, 0.10 mmol, 45% yield) as a white solid. [α]^25^_D_ = −75.6 (*c* = 1.0, CHCl_3_). m.p. 200–201 °C. ^1^H NMR (600 MHz, DMSO-*d*_6_) δ 8.30 (d, *J* = 8.7 Hz, 1H), 7.46 (d, *J* = 8.8 Hz, 1H), 7.01 (d, *J* = 9.4 Hz, 1H), 5.06 (t, *J* = 7.7 Hz, 1H), 4.79 (dd, *J* = 9.2, 5.8 Hz, 1H), 4.64 (t, *J* = 8.4 Hz, 2H), 4.15 (dt, *J* = 9.5, 4.7 Hz, 1H), 2.95 (s, 3H), 2.54 (s, 3H), 1.88–1.15 (m, 15H), 1.15–0.41 (m, 30H). ^13^C NMR (151 MHz, DMSO-*d*_6_) δ 173.7, 171.7, 171.5, 171.1, 169.7, 55.9, 53.5, 52.3, 47.6, 47.51, 41.7, 41.2, 40.9, 37.1, 34.9, 30.9, 29.3, 25.0, 24.9, 24.8, 24.7, 24.7, 23.4, 23.4, 23.2, 23.0, 22.9, 22.7, 22.3, 22.1, 21.4. HRMS (ESI) *m*/*z* calcd. for C_32_H_59_N_5_O_5_ [M + Na]^+^: 616.4408; found: 616.4415.
*Cyclo(Me-L-Leu-D-Leu-D-Leu-Me-L-Leu-D-Leu)* **Gala06**. The product **10f** (134 mg, 0.22 mmol, 1 equiv) was dissolved in MeCN (4.3 mM) and reacted according to route E for macrolactamization. PyBOP (229 mg, 0.44 mmol, 2 equiv), DMAP (80 mg, 0.66 mmol, 3 equiv). The crude residue was purified by column chromatography (hexane/EtOAc = 60:40, *v*/*v*), obtaining compound **Gala06** (69 mg, 0.12 mmol, 54% yield) as a white solid. [α]^25^_D_ = −85.6 (*c* = 1.0, CHCl_3_). m.p. 200–201 °C. ^1^H NMR (600 MHz, DMSO-*d*_6_) δ 8.29 (d, *J* = 8.7 Hz, 1H), 7.45 (d, *J* = 8.8 Hz, 1H), 7.00 (d, *J* = 9.4 Hz, 1H), 5.05 (t, *J* = 7.7 Hz, 1H), 4.79 (td, *J* = 9.0, 5.5 Hz, 1H), 4.63 (t, *J* = 8.4 Hz, 2H), 4.15 (td, *J* = 9.5, 5.2 Hz, 1H), 2.94 (s, 3H), 2.53 (s, 3H), 1.76–1.14 (m, 16H), 0.97–0.58 (m, 30H). ^13^C NMR (151 MHz, DMSO-*d*_6_) δ 173.7, 171.7, 171.5, 171.2, 169.7, 55.9, 53.5, 52.3, 47.6, 47.5, 41.6, 41.3, 40.9, 37.1, 34.9, 30.9, 29.3, 25.0, 24.9, 24.8, 24.8, 24.7, 23.4, 23.4, 23.2, 23.0, 22.9, 22.8, 22.3, 22.1, 21.4. HRMS (ESI) *m*/*z* calcd. for C_32_H_59_N_5_O_5_ [M + Na]^+^: 616.4408; found: 616.4415.
*Cyclo(Me-D-Leu-D-Leu-L-Leu-Me-D-Leu-D-Leu)* **Gala07**. The product **10g** (134 mg, 0.22 mmol, 1 equiv) was dissolved in MeCN (4.3 mM) and reacted according to route E for macrolactamization. PyBOP (229 mg, 0.44 mmol, 2 equiv), DMAP (80 mg, 0.66 mmol, 3 equiv). The crude residue was purified by column chromatography (hexane/EtOAc = 60:40, *v*/*v*), obtaining compound **Gala07** (65 mg, 0.11 mmol, 50% yield) as a white solid. [α]^25^_D_ = −10.6 (*c* = 1.0, CHCl_3_). m.p. 199–200 °C. ^1^H NMR (600 MHz, DMSO-*d*_6_) δ 7.63 (d, *J* = 8.0 Hz, 1H), 7.39 (d, *J* = 8.2 Hz, 1H), 7.19 (d, *J* = 8.8 Hz, 1H), 5.11 (t, *J* = 7.6 Hz, 1H), 4.77 (d, *J* = 7.4 Hz, 1H), 4.71 (d, *J* = 7.8 Hz, 1H), 4.49 (dd, *J* = 10.1, 5.2 Hz, 1H), 4.27–3.96 (m, 1H), 2.99 (s, 3H), 2.73 (s, 3H), 1.73–1.26 (m, 15H), 0.87 (m, 30H). ^13^C NMR (151 MHz, DMSO-*d*_6_) δ 174.53, 172.27, 171.37, 170.84, 170.57, 57.94, 53.64, 51.37, 48.34, 47.69, 41.40, 41.09, 38.70, 37.23, 35.17, 31.34, 29.88, 25.26, 24.92, 24.87, 24.81, 24.77, 23.50, 23.40, 23.15, 23.02, 22.98, 22.06, 21.97, 21.91, 21.70. HRMS (ESI) *m*/*z* calcd. for C_32_H_59_N_5_O_5_ [M + Na]^+^: 616.4408; found: 616.4415.
*Cyclo(Me-L-Leu-L-Leu-D-Pro-Me-L-Leu-L-Leu)* **Gala08**. The product **10h** (131 mg, 0.22 mmol, 1 equiv) was dissolved in MeCN (4.3 mM) and reacted according to route E for macrolactamization. PyBOP (229 mg, 0.44 mmol, 2 equiv), DMAP (80 mg, 0.66 mmol, 3 equiv). The crude residue was purified by column chromatography (hexane/EtOAc = 60:40, *v*/*v*), obtaining compound **Gala08** (65 mg, 0.11 mmol, 51% yield) as a white solid. [α]^25^_D_ = −75.6 (*c* = 1.0, CHCl_3_). m.p. 230–231 °C. ^1^H NMR (600 MHz, DMSO-*d*_6_) δ 7.37 (d, *J* = 7.3 Hz, 1H), 6.89 (d, *J* = 7.5 Hz, 1H), 4.91 (t, *J* = 7.8 Hz, 1H), 4.75 (td, *J* = 8.5, 8.1, 5.3 Hz, 2H), 4.60 (dd, *J* = 10.5, 5.8 Hz, 1H), 4.33 (ddd, *J* = 11.6, 7.5, 4.2 Hz, 1H), 3.51 (t, *J* = 8.5 Hz, 1H), 3.45 (q, *J* = 5.3, 3.8 Hz, 1H), 3.02 (d, *J* = 11.7 Hz, 6H), 2.17 (q, *J* = 6.4 Hz, 1H), 2.04–1.91 (m, 1H), 1.88 (dd, *J* = 12.5, 6.6 Hz, 1H), 1.81–1.74 (m, 1H), 1.74–1.66 (m, 1H), 1.65–1.29 (m, 11H), 0.99–0.69 (m, 24H). ^13^C NMR (151 MHz, DMSO-*d*_6_) δ 173.9, 173.0, 171.3, 170.2, 169.5, 57.6, 56.0, 53.8, 50.2, 47.9, 46.4, 42.3, 36.8, 36.3, 31.0, 30.8, 28.5, 25.4, 25.2, 25.1, 24.9, 24.7, 23.7, 23.4, 23.3, 23.2, 22.7, 22.4, 22.0, 21.8. HRMS (ESI) *m*/*z* calcd. for C_31_H_55_N_5_O_5_ [M + Na]^+^: 600.4095; found: 600.4103.
*Cyclo(Me-L-Leu-L-Leu-D-Phe-Me-L-Leu-L-Leu)* (**Gala09**). The product **10i** (142 mg, 0.22 mmol, 1 equiv) was dissolved in MeCN (4.3 mM) and reacted according to route E for macrolactamization. PyBOP (229 mg, 0.44 mmol, 2 equiv), DMAP (80 mg, 0.66 mmol, 3 equiv). The crude residue was purified by column chromatography (hexane/EtOAc = 60:40, *v*/*v*), obtaining compound **Gala09** (70 mg, 0.11 mmol, 51% yield) as a white solid. [α]^25^_D_ = −55.6 (*c* = 1.0, CHCl_3_). m.p. 200–201 °C. ^1^H NMR (600 MHz, DMSO-*d*_6_) δ 7.60 (d, *J* = 7.8 Hz, 1H), 7.57 (d, *J* = 8.8 Hz, 1H), 7.46 (d, *J* = 8.1 Hz, 1H), 7.35–7.04 (m, 5H), 5.06 (dd, *J* = 9.8, 6.0 Hz, 1H), 4.87 (q, *J* = 7.9 Hz, 1H), 4.76 (q, *J* = 7.5 Hz, 1H), 4.58 (dd, *J* = 10.1, 5.6 Hz, 1H), 4.12 (td, *J* = 8.8, 5.7 Hz, 1H), 3.05 (dd, *J* = 13.1, 8.4 Hz, 1H), 2.97 (s, 3H), 2.76 (dd, *J* = 13.2, 6.2 Hz, 1H), 2.63 (s, 3H), 1.86–1.45 (m, 7H), 1.40 (q, *J* = 8.2, 7.3 Hz, 4H), 1.24–1.07 (m, 1H), 0.97–0.59 (m, 24H). ^13^C NMR (151 MHz, DMSO-*d*_6_) δ 174.2, 171.8, 171.3, 170.9, 170.4, 138.2, 129.7, 128.4, 126.6, 57.7, 53.7, 51.6, 51.2, 48.2, 41.4, 38.7, 37.8, 37.2, 35.2, 31.2, 29.8, 25.2, 24.8, 24.8, 24.7, 23.5, 23.4, 23.2, 22.2, 22.0, 21.9. HRMS (ESI) *m*/*z* calcd. for C_35_H_57_N_5_O_5_ [M + Na]^+^: 650.4252; found: 650.4259.
*Cyclo(Me-L-Leu-L-Leu-Gly-Me-L-Leu-L-Leu)* **Gala10**. The product **10j** (122 mg, 0.22 mmol, 1 equiv) was dissolved in MeCN (4.3 mM) and reacted according to route E for macrolactamization. PyBOP (229 mg, 0.44 mmol, 2 equiv), DMAP (80 mg, 0.66 mmol, 3 equiv). The crude residue was purified by column chromatography (hexane/EtOAc = 60:40, *v*/*v*), obtaining compound **Gala10** (60 mg, 0.11 mmol, 51% yield) as a white solid. [α]^25^_D_ = −65.5 (*c* = 1.0, CHCl_3_). m.p. 200–201 °C. ^1^H NMR (600 MHz, DMSO-*d*_6_) δ 8.34–7.84 (m, 1H), 7.76–7.38 (m, 1H), 7.22 (d, *J* = 4.3 Hz, 1H), 5.04 (t, *J* = 7.7 Hz, 1H), 4.83 (td, *J* = 9.0, 4.6 Hz, 1H), 4.60 (dd, *J* = 13.8, 9.1 Hz, 1H), 4.45 (dd, *J* = 9.7, 5.3 Hz, 1H), 4.09 (ddd, *J* = 10.8, 8.1, 4.8 Hz, 1H), 3.25 (d, *J* = 13.7 Hz, 1H), 3.01 (s, 3H), 2.64 (s, 3H), 1.78–1.30 (m, 12H), 1.03–0.62 (m, 24H). ^13^C NMR (151 MHz, DMSO-*d*_6_) δ 174.7, 171.7, 171.3, 170.3, 170.2, 58.1, 53.8, 51.8, 48.3, 43.12, 41.5, 38.3, 37.4, 35.1, 31.6, 31.5, 30.4, 25.3, 24.8, 24.8, 24.8, 23.6, 23.5, 23.4, 23.1, 22.3, 22.2, 22.0, 21.4. HRMS (ESI) *m*/*z* calcd. for C_28_H_51_N_5_O_5_ [M + Na]^+^: 560.3782; found: 560.3786.
*Cyclo(Me-L-Leu-L-Leu-D-Ala-Me-L-Leu-L-Leu)* **Gala11**. The product **10k** (125 mg, 0.22 mmol, 1 equiv) was dissolved in MeCN (4.3 mM) and reacted according to route E for macrolactamization. PyBOP (229 mg, 0.44 mmol, 2 equiv), DMAP (80 mg, 0.66 mmol, 3 equiv). The crude residue was purified by column chromatography (hexane/EtOAc = 60:40, *v*/*v*), obtaining compound **Gala11** (59 mg, 0.11 mmol, 49% yield) as a white solid. [α]^25^_D_ = −54.6 (*c* = 1.0, CHCl_3_). m.p. 191–192 °C. ^1^H NMR (600 MHz, DMSO-*d*_6_) δ 7.78 (d, *J* = 8.3 Hz, 1H), 7.21 (d, *J* = 8.4 Hz, 1H), 7.10 (d, *J* = 8.8 Hz, 1H), 5.13 (t, *J* = 7.6 Hz, 1H), 4.83 (td, *J* = 8.7, 5.0 Hz, 1H), 4.74 (dd, *J* = 8.7, 6.1 Hz, 1H), 4.40 (dd, *J* = 10.1, 5.1 Hz, 1H), 4.12 (ddd, *J* = 12.1, 8.2, 4.3 Hz, 1H), 3.01 (s, 3H), 2.63 (s, 3H), 1.80–1.65 (m, 2H), 1.65–1.29 (m, 10H), 1.12 (d, *J* = 6.3 Hz, 3H), 1.04–0.60 (m, 24H). ^13^C NMR (151 MHz, DMSO-*d*_6_) δ 174.88, 172.24, 171.62, 170.87, 170.38, 58.31, 53.56, 51.46, 48.43, 45.25, 41.50, 38.45, 37.38, 35.00, 31.48, 29.57, 25.30, 24.84, 24.81, 23.68, 23.55, 23.31, 23.06, 22.28, 22.07, 21.92, 21.31, 17.85. HRMS (ESI) *m*/*z* calcd. for C_29_H_53_N_5_O_5_ [M + Na]^+^: 574.3939; found: 574.3943.
*Cyclo(Me-L-Leu-L-Leu-O-Me-D-Ser-Me-L-Leu-L-Leu)* **Gala12**. The product **10l** (132 mg, 0.22 mmol, 1 equiv) was dissolved in MeCN (4.3 mM) and reacted according to route E for macrolactamization. PyBOP (229 mg, 0.44 mmol, 2 equiv), DMAP (80 mg, 0.66 mmol, 3 equiv). The crude residue was purified by column chromatography (hexane/EtOAc = 60:40, *v*/*v*), obtaining compound **Gala12** (67 mg, 0.12 mmol, 52% yield) as a white solid. [α]^25^_D_ = −75.6 (*c* = 1.0, CHCl_3_). m.p. 203–204 °C. ^1^H NMR (600 MHz, DMSO-*d*_6_) δ 7.80 (d, *J* = 8.2 Hz, 1H), 7.31 (d, *J* = 8.4 Hz, 1H), 7.09 (d, *J* = 9.0 Hz, 1H), 5.10 (t, *J* = 7.7 Hz, 1H), 4.82 (td, *J* = 8.2, 4.1 Hz, 2H), 4.45 (dd, *J* = 10.1, 4.9 Hz, 1H), 4.13 (ddd, *J* = 10.8, 8.2, 4.6 Hz, 1H), 3.63 (t, *J* = 8.6 Hz, 1H), 3.28 (dd, *J* = 9.4, 5.7 Hz, 1H), 3.23 (s, 3H), 2.99 (s, 3H), 2.67 (s, 3H), 1.78–1.65 (m, 2H), 1.64–1.31 (m, 10H), 1.05–0.57 (m, 24H). ^13^C NMR (151 MHz, DMSO-*d*_6_) δ 174.7, 171.5, 171.3, 170.7, 170.3, 71.7, 58.8, 58.0, 53.6, 51.4, 48.6, 48.4, 41.4, 38.4, 37.3, 35.1, 31.4, 29.7, 25.3, 25.0, 24.8, 23.6, 23.5, 23.3, 23.1, 22.2, 22.0, 22.0, 21.5. HRMS (ESI) *m*/*z* calcd. for C_30_H_55_N_5_O_6_ [M + Na]^+^: 604.4045; found: 604.4051.
*Cyclo(Me-L-Leu-L-Leu-O-Tyr-Me-L-Leu-L-Leu)* **Gala13**. The product **10m** (148 mg, 0.22 mmol, 1 equiv) was dissolved in MeCN (4.3 mM) and reacted according to route E for macrolactamization. PyBOP (229 mg, 0.44 mmol, 2 equiv), DMAP (80 mg, 0.66 mmol, 3 equiv). The crude residue was purified by column chromatography (hexane/EtOAc = 60:40, *v*/*v*), obtaining compound **Gala13** (75 mg, 0.11 mmol, 52% yield) as a white solid. [α]^25^_D_ = −34.6 (*c* = 1.0, CHCl_3_). m.p. 205–206 °C. ^1^H NMR (600 MHz, DMSO-*d*_6_) δ 7.57 (t, *J* = 7.9 Hz, 2H), 7.45 (d, *J* = 8.2 Hz, 1H), 7.25–6.99 (m, 2H), 6.91–6.66 (m, 2H), 5.04 (dd, *J* = 9.9, 6.0 Hz, 1H), 4.78 (dtd, *J* = 39.0, 8.5, 6.1 Hz, 2H), 4.57 (dd, *J* = 10.2, 5.6 Hz, 1H), 4.11 (ddd, *J* = 9.6, 7.9, 5.6 Hz, 1H), 3.70 (s, 3H), 2.96 (s, 3H), 2.94 (s, 1H), 2.69 (dd, *J* = 13.1, 6.4 Hz, 1H), 2.62 (s, 3H), 1.78–1.44 (m, 7H), 1.39 (m, 5H), 0.96–0.69 (m, 24H). ^13^C NMR (151 MHz, DMSO-*d*_6_) δ 174.1, 172.0, 171.3, 170.9, 170.4, 158.3, 130.7, 130.0, 113.9, 57.7, 55.4, 53.6, 51.5, 51.3, 48.2, 41.4, 38.8, 37.2, 36.9, 35.3, 31.2, 29.9, 25.2, 24.9, 24.8, 24.6, 23.5, 23.4, 23.4, 23.2, 22.2, 22.0, 21.9. HRMS (ESI) *m*/*z* calcd. for C_36_H_59_N_5_O_6_ [M + Na]^+^: 680.4358; found: 680.4365.
*Cyclo(Me-L-Leu-L-Leu-Methylester-D-Asp-Me-L-Leu-L-Leu)* **Gala14**. The product **10n** (138 mg, 0.22 mmol, 1 equiv) was dissolved in MeCN (4.3 mM) and reacted according to route E for macrolactamization. PyBOP (229 mg, 0.44 mmol, 2 equiv), DMAP (80 mg, 0.66 mmol, 3 equiv). The crude residue was purified by column chromatography (hexane/EtOAc = 60:40, *v*/*v*), obtaining compound **Gala14** (70 mg, 0.11 mmol, 52% yield) as a white solid. [α]^25^_D_ = −45.9 (*c* = 1.0, CHCl_3_). m.p. 204–205 °C. ^1^H NMR (600 MHz, DMSO-*d*_6_) δ 7.88 (d, *J* = 8.0 Hz, 1H), 7.28 (dd, *J* = 8.7, 6.4 Hz, 2H), 5.09 (dd, *J* = 9.6, 6.0 Hz, 1H), 4.99 (td, *J* = 9.6, 4.3 Hz, 1H), 4.80 (td, *J* = 8.9, 4.8 Hz, 1H), 4.47 (dd, *J* = 9.9, 5.0 Hz, 1H), 4.05 (ddd, *J* = 11.4, 8.0, 4.4 Hz, 1H), 3.55 (s, 3H), 3.00 (s, 3H), 2.78 (dd, *J* = 16.1, 10.1 Hz, 1H), 2.67 (s, 3H), 2.47 (dd, *J* = 16.1, 4.3 Hz, 1H), 1.78–1.28 (m, 13H), 0.88 (m, 24H). ^13^C NMR (151 MHz, DMSO-*d*_6_) δ 174.66, 171.57, 171.15, 171.05, 170.84, 170.33, 57.80, 53.77, 51.81, 51.64, 48.37, 46.57, 41.30, 38.24, 37.35, 36.25, 34.91, 31.40, 29.62, 25.25, 24.80, 24.78, 23.64, 23.49, 23.43, 23.11, 22.07, 22.01, 21.94, 21.45. HRMS (ESI) *m*/*z* calcd. for C_31_H_55_N_5_O_7_ [M + Na]^+^: 632.3994; found: 632.4000.
*Cyclo(Me-L-Leu-L-Leu-3-Flu-D-Phe-Me-L-Leu-L-Leu)* **Gala15**. The product **10o** (145 mg, 0.22 mmol, 1 equiv) was dissolved in MeCN (4.3 mM) and reacted according to route E for macrolactamization. PyBOP (229 mg, 0.44 mmol, 2 equiv), DMAP (80 mg, 0.66 mmol, 3 equiv). The crude residue was purified by column chromatography (hexane/EtOAc = 60:40, *v*/*v*), obtaining compound **Gala15** (75 mg, 0.12 mmol, 53% yield) as a white solid. [α]^25^_D_ = −54.8 (*c* = 1.0, CHCl_3_). m.p. 205–206 °C. ^1^H NMR (600 MHz, DMSO-*d*_6_) δ 7.59 (t, *J* = 9.1 Hz, 2H), 7.45 (d, *J* = 8.1 Hz, 1H), 7.27 (q, *J* = 7.4 Hz, 1H), 7.10–6.83 (m, 3H), 5.07 (dd, *J* = 10.0, 5.9 Hz, 1H), 4.89 (q, *J* = 7.8 Hz, 1H), 4.75 (q, *J* = 7.8 Hz, 1H), 4.58 (dd, *J* = 10.1, 5.6 Hz, 1H), 4.09 (td, *J* = 8.7, 5.6 Hz, 1H), 3.05 (dd, *J* = 13.2, 7.8 Hz, 1H), 2.97 (s, 3H), 2.79 (dd, *J* = 13.2, 6.8 Hz, 1H), 2.66 (s, 3H), 1.71–1.15 (m, 13H), 1.00–0.58 (m, 24H). ^13^C NMR (151 MHz, DMSO-*d*_6_) δ 174.2, 171.7, 171.3, 170.8, 170.4, 163.3, 141.2, 141.2, 130.3, 130.2, 125.9, 116.5, 116.4, 60.2, 57.7, 53.7, 51.6, 50.9, 48.3, 41.3, 38.7, 37.4, 37.2, 35.3, 31.2, 29.9, 25.2, 24.9, 24.8, 24.7, 23.5, 23.4, 23.3, 23.2, 22.2, 22.0, 21.9, 21.8. HRMS (ESI) *m*/*z* calcd. for C_35_H_56_FN_5_O_5_ [M + Na]^+^: 668.4158; found: 668.4164.
*Cyclo(Me-L-Leu-L-Leu-2-Me-D-Phe-Me-L-Leu-L-Leu)* **Gala16**. The product **10p** (145 mg, 0.22 mmol, 1 equiv) was dissolved in MeCN (4.3 mM) and reacted according to route E for macrolactamization. PyBOP (229 mg, 0.44 mmol, 2 equiv), DMAP (80 mg, 0.66 mmol, 3 equiv). The crude residue was purified by column chromatography (hexane/EtOAc = 60:40, *v*/*v*), obtaining compound **Gala16** (75 mg, 0.12 mmol, 53% yield) as a white solid. [α]^25^_D_ = −35.6 (*c* = 1.0, CHCl_3_). m.p. 235–236 °C. ^1^H NMR (600 MHz, DMSO-*d*_6_) δ 7.66 (d, *J* = 8.0 Hz, 1H), 7.50 (d, *J* = 8.6 Hz, 1H), 7.45 (d, *J* = 8.3 Hz, 1H), 7.18–6.85 (m, 4H), 5.04 (dd, *J* = 9.9, 6.1 Hz, 1H), 4.90 (td, *J* = 8.8, 5.6 Hz, 1H), 4.76 (q, *J* = 7.6 Hz, 1H), 4.57 (dd, *J* = 10.3, 5.4 Hz, 1H), 4.12 (td, *J* = 8.8, 5.3 Hz, 1H), 3.08 (dd, *J* = 13.5, 9.0 Hz, 1H), 2.96 (s, 3H), 2.75 (dd, *J* = 13.5, 5.6 Hz, 1H), 2.59 (s, 3H), 2.32 (s, 3H), 1.77–1.29 (m, 11H), 1.18–1.04 (m, 1H), 1.00–0.53 (m, 24H). ^13^C NMR (151 MHz, DMSO-*d*_6_) δ 174.2, 171.7, 171.3, 171.0, 170.3, 136.7, 136.1, 130.4, 130.1, 126.7, 125.9, 57.7, 53.6, 51.4, 49.9, 48.2, 41.4, 38.7, 37.2, 35.1, 34.9, 31.2, 29.8, 25.2, 24.8, 24.8, 24.6, 23.5, 23.4, 23.2, 22.1, 22.0, 21.9, 21.8, 19.5. HRMS (ESI) *m*/*z* calcd. for C_36_H_59_N_5_O_5_ [M + H]^+^: 664.4408; found: 664.4411.
*Cyclo(Me-L-Leu-L-Leu-4-Chl-D-Phe-Me-L-Leu-L-Leu)* **Gala17**. The product **10q** (149 mg, 0.22 mmol, 1 equiv) was dissolved in MeCN (4.3 mM) and reacted according to route E for macrolactamization. PyBOP (229 mg, 0.44 mmol, 2 equiv), DMAP (80 mg, 0.66 mmol, 3 equiv). The crude residue was purified by column chromatography (hexane/EtOAc = 60:40, *v*/*v*), obtaining compound **Gala17** (80 mg, 0.12 mmol, 55% yield) as a white solid. [α]^25^_D_ = −75.6 (*c* = 1.0, CHCl_3_).−67.5 m.p. 235–236 °C. ^1^H NMR (600 MHz, DMSO-*d*_6_) δ7.60 (d, *J* = 7.8 Hz, 1H), 7.55 (d, *J* = 8.6 Hz, 1H), 7.42 (dd, *J* = 20.0, 8.1 Hz, 1H), 7.30–7.16 (m, 4H), 5.04 (dt, *J* = 10.5, 5.2 Hz, 1H), 4.95–4.80 (m, 1H), 4.80–4.68 (m, 1H), 4.57 (dt, *J* = 11.6, 5.8 Hz, 1H), 4.18–3.81 (m, 1H), 3.03 (m, 1H), 2.97 (s, 3H), 2.76 (dd, *J* = 13.3, 6.4 Hz, 1H), 2.64 (d, *J* = 12.2 Hz, 3H), 1.76–1.20 (m, 12H), 1.03–0.53 (m, 24H). ^13^C NMR (151 MHz, DMSO-*d*_6_) δ 174.2, 171.8, 171.3, 170.9, 170.4, 138.2, 131.7, 129.7, 128.5, 128.4, 57.8, 57.7, 53.7, 51.5, 51.2, 48.2, 41.4, 38.7, 37.2, 35.2, 31.2, 29.9, 25.2, 24.8, 24.8, 24.7, 23.5, 23.4, 23.4, 23.2, 22.2, 22.0, 21.9, 21.9. HRMS (ESI) *m*/*z* calcd. for C_35_H_56_ClN_5_O_5_ [M + H]^+^: 684.3862; found: 684.3864.
*Cyclo(Me-L-Leu-L-Leu-4-Bro-D-Phe-Me-L-Leu-L-Leu)* **Gala18**. The product **10r** (159 mg, 0.22 mmol, 1 equiv) was dissolved in MeCN (4.3 mM) and reacted according to route E for macrolactamization. PyBOP (229 mg, 0.44 mmol, 2 equiv), DMAP (80 mg, 0.66 mmol, 3 equiv). The crude residue was purified by column chromatography (hexane/EtOAc = 60:40, *v*/*v*), obtaining compound **Gala18** (85 mg, 0.12 mmol, 55% yield) as a white solid. [α]^25^_D_ = −65.6 (*c* = 1.0, CHCl_3_). m.p. 245–246 °C. ^1^H NMR (600 MHz, DMSO-*d*_6_) δ 7.58 (dd, *J* = 17.6, 8.3 Hz, 2H), 7.45 (d, *J* = 8.1 Hz, 1H), 7.20 (m, 5H), 5.05 (t, *J* = 7.9 Hz, 1H), 4.87 (q, *J* = 8.0 Hz, 1H), 4.75 (q, *J* = 7.6 Hz, 1H), 4.57 (dd, *J* = 10.3, 5.6 Hz, 1H), 4.11 (q, *J* = 7.8 Hz, 1H), 3.04 (dd, *J* = 13.1, 8.5 Hz, 1H), 2.97 (s, 3H), 2.76 (dd, *J* = 13.3, 6.2 Hz, 1H), 2.63 (s, 3H), 1.92–1.08 (m, 13H), 1.04–0.53 (m, 24H). ^13^C NMR (151 MHz, DMSO-*d*_6_) δ 174.2, 171.8, 171.3, 170.9, 170.4, 138.2, 129.7, 128.5, 126.7, 57.7, 53.7, 51.2, 48.2, 41.4, 38.7, 37.8, 37.2, 35.2, 31.2, 29.9, 25.2, 24.8, 24.8, 24.7, 23.5, 23.4, 23.2, 22.2, 22.0, 21.9, 21.9. HRMS (ESI) *m*/*z* calcd. for C_35_H_56_BrN_5_O_5_ [M + H]^+^: 728.3357; found: 728.3360.
*Cyclo(Me-L-Leu-L-Leu-D-cyclohexyl-Gly-Me-L-Leu-L-Leu)* **Gala19**. The product **10s** (140 mg, 0.22 mmol, 1 equiv) was dissolved in MeCN (4.3 mM) and reacted according to route E for macrolactamization. PyBOP (229 mg, 0.44 mmol, 2 equiv), DMAP (80 mg, 0.66 mmol, 3 equiv). The crude residue was purified by column chromatography (hexane/EtOAc = 60:40, *v*/*v*), obtaining compound **Gala19** (70 mg, 0.11 mmol, 51% yield) as a white solid. [α]^25^_D_ = −35.6 (*c* = 1.0, CHCl_3_). m.p. 204–205 °C. ^1^H NMR (600 MHz, DMSO-*d*_6_) δ 7.63 (d, *J* = 7.8 Hz, 1H), 7.45 (d, *J* = 9.1 Hz, 1H), 7.39 (d, *J* = 7.8 Hz, 1H), 5.10 (dd, *J* = 10.2, 6.0 Hz, 1H), 4.71 (t, *J* = 7.4 Hz, 1H), 4.69–4.54 (m, 1H), 4.43 (t, *J* = 9.5 Hz, 1H), 4.24 (q, *J* = 7.6 Hz, 1H), 2.95 (s, 3H), 2.81 (s, 3H), 1.77 (d, *J* = 11.1 Hz, 1H), 1.73–1.56 (m, 8H), 1.57–1.22 (m, 10H), 1.13 (p, *J* = 12.9, 12.4 Hz, 3H), 0.97–0.73 (m, 26H). ^13^C NMR (151 MHz, DMSO-*d*_6_) δ 174.0, 172.5, 171.1, 171.0, 170.7, 57.5, 54.1, 53.7, 51.3, 48.2, 41.2, 37.0, 35.6, 31.1, 30.3, 29.8, 28.6, 26.5, 25.9, 25.2, 25.0, 24.7, 23.5, 23.2, 22.2, 22.1, 21.9, 21.7. HRMS (ESI) *m*/*z* calcd. for C_34_H_61_N_5_O_5_ [M + Na]^+^: 642.4565; found: 642.4571.
*Cyclo(Me-L-Leu-L-Leu-D-phenyl-Gly-Me-L-Leu-L-Leu)* **Gala20**. The product **10t** (140 mg, 0.22 mmol, 1 equiv) was dissolved in MeCN (4.3 mM) and reacted according to route E for macrolactamization. PyBOP (229 mg, 0.44 mmol, 2 equiv), DMAP (80 mg, 0.66 mmol, 3 equiv). The crude residue was purified by column chromatography (hexane/EtOAc = 60:40, *v*/*v*), obtaining compound **Gala20** (70 mg, 0.11 mmol, 51% yield) as a white solid. [α]^25^_D_ = −34.6 (*c* = 1.0, CHCl_3_). m.p. 204–205 °C. ^1^H NMR (600 MHz, DMSO-*d*_6_) δ 7.89 (d, *J* = 8.2 Hz, 1H), 7.77 (d, *J* = 8.8 Hz, 1H), 7.31 (m, 5H), 5.88 (d, *J* = 8.8 Hz, 1H), 5.08 (t, *J* = 7.8 Hz, 1H), 4.86 (td, *J* = 9.4, 4.0 Hz, 1H), 4.47 (dd, *J* = 9.9, 5.4 Hz, 1H), 4.20 (td, *J* = 9.4, 8.8, 4.7 Hz, 1H), 3.03 (s, 3H), 2.80 (s, 3H), 1.84–1.13 (m, 13H), 0.89 (m, 24H). ^13^C NMR (151 MHz, DMSO-*d*_6_) δ 174.8, 171.8, 171.3, 170.2, 138.8, 128.3, 128.1, 127.6, 58.5, 53.9, 53.4, 51.4, 48.5, 41.4, 38.4, 37.5, 35.0, 31.5, 30.1, 25.3, 24.9, 24.8, 24.8, 24.6, 23.6, 23.6, 23.3, 23.0, 22.3, 22.2, 21.9, 21.3. HRMS (ESI) *m*/*z* calcd. for C_34_H_55_N_5_O_5_ [M + Na]^+^: 636.4095; found: 636.4102.
*Cyclo(Me-L-Leu-L-Leu-D-Val-Me-L-Leu-L-Leu)* **Gala21**. The product **10u** (131 mg, 0.22 mmol, 1 equiv) was dissolved in MeCN (4.3 mM) and reacted according to route E for macrolactamization. PyBOP (229 mg, 0.44 mmol, 2 equiv), DMAP (80 mg, 0.66 mmol, 3 equiv). The crude residue was purified by column chromatography (hexane/EtOAc = 60:40, *v*/*v*), obtaining compound **Gala21** (65 mg, 0.11 mmol, 50% yield) as a white solid. [α]^25^_D_ = −38.7 (*c* = 1.0, CHCl_3_). m.p. 192–193 °C. ^1^H NMR (600 MHz, DMSO-*d*_6_) δ 7.62 (d, *J* = 7.8 Hz, 1H), 7.57 (d, *J* = 9.1 Hz, 1H), 7.37 (d, *J* = 7.7 Hz, 1H), 5.12 (dd, *J* = 10.3, 5.7 Hz, 1H), 4.70 (d, *J* = 7.4 Hz, 1H), 4.63 (dd, *J* = 10.4, 5.7 Hz, 1H), 4.38 (t, *J* = 9.4 Hz, 1H), 4.24 (d, *J* = 7.4 Hz, 1H), 2.95 (s, 3H), 2.83 (s, 3H), 2.04 (dt, *J* = 9.7, 6.7 Hz, 1H), 1.76–1.18 (m, 12H), 0.94–0.77 (m, 26H). ^13^C NMR (151 MHz, DMSO-*d*_6_) δ 173.9, 172.7, 171.1, 171.0, 170.7, 57.5, 55.3, 53.7, 51.5, 48.2, 41.2, 39.1, 37.0, 35.7, 31.1, 30.3, 29.9, 25.2, 25.0, 25.0, 24.7, 23.5, 23.5, 23.3, 23.1, 22.3, 22.2, 21.9, 21.7, 19.6, 18.9. HRMS (ESI) *m*/*z* calcd. for C_31_H_57_N_5_O_5_ [M + H]^+^: 602.4252; found: 602.4261.
*Cyclo(Me-L-Leu-L-Leu-D-Met-Me-L-Leu-L-Leu)* **Gala22**. The product **10v** (138 mg, 0.22 mmol, 1 equiv) was dissolved in MeCN (4.3 mM) and reacted according to route E for macrolactamization. PyBOP (229 mg, 0.44 mmol, 2 equiv), DMAP (80 mg, 0.66 mmol, 3 equiv). The crude residue was purified by column chromatography (hexane/EtOAc = 60:40, *v*/*v*), obtaining compound **Gala22** (70 mg, 0.11 mmol, 52% yield) as a white solid. [α]^25^_D_ = −54.7 (*c* = 1.0, CHCl_3_). m.p. 174–175 °C. ^1^H NMR (600 MHz, DMSO-*d*_6_) δ 7.71 (d, *J* = 8.1 Hz, 1H), 7.33 (d, *J* = 8.2 Hz, 1H), 7.26 (d, *J* = 8.9 Hz, 1H), 5.12 (dd, *J* = 9.5, 6.3 Hz, 1H), 4.81 (dq, *J* = 22.6, 7.3 Hz, 2H), 4.47 (dd, *J* = 10.1, 5.0 Hz, 1H), 4.16 (ddd, *J* = 10.7, 8.0, 5.0 Hz, 1H), 2.99 (s, 3H), 2.70 (s, 3H), 2.44–2.18 (m, 2H), 2.03 (s, 4H), 1.70 (m, 3H), 1.66–1.44 (m, 7H), 1.39 (m, 3H), 0.88 (m, 24H). ^13^C NMR (151 MHz, DMSO-*d*_6_) δ 174.6, 171.6, 171.5, 171.1, 170.4, 58.0, 53.6, 51.4, 48.5, 48.4, 41.4, 38.5, 37.3, 35.1, 31.5, 31.4, 30.0, 29.8, 25.3, 24.9, 24.8, 24.8, 23.6, 23.5, 23.4, 23.1, 22.0, 22.0, 21.6, 14.9. HRMS (ESI) *m*/*z* calcd. for C_31_H_57_N_5_O_5_S [M + Na]^+^: 634.3973; found: 634.3981.
*Cyclo(Me-L-Leu-L-Leu-3-Me-D-Phe-Me-L-Leu-L-Leu)* **Gala23**. The product **10w** (145 mg, 0.22 mmol, 1 equiv) was dissolved in MeCN (4.3 mM) and reacted according to route E for macrolactamization. PyBOP (229 mg, 0.44 mmol, 2 equiv), DMAP (80 mg, 0.66 mmol, 3 equiv). The crude residue was purified by column chromatography (hexane/EtOAc = 60:40, *v*/*v*), obtaining compound **Gala23** (75 mg, 0.12 mmol, 53% yield) as a white solid. [α]^25^_D_ = −45.6 (*c* = 1.0, CHCl_3_). m.p. 194–195 °C. ^1^H NMR (600 MHz, DMSO-*d*_6_) δ 7.62 (d, *J* = 7.9 Hz, 1H), 7.55 (d, *J* = 8.8 Hz, 1H), 7.45 (d, *J* = 8.1 Hz, 1H), 7.11 (t, *J* = 7.5 Hz, 1H), 7.05–6.87 (m, 3H), 5.06 (dd, *J* = 9.8, 6.1 Hz, 1H), 4.84 (d, *J* = 7.7 Hz, 1H), 4.76 (t, *J* = 7.5 Hz, 1H), 4.57 (dd, *J* = 10.4, 5.5 Hz, 1H), 4.13 (d, *J* = 7.6 Hz, 1H), 3.01 (dd, *J* = 13.1, 8.5 Hz, 1H), 2.97 (s, 3H), 2.71 (dd, *J* = 13.3, 6.2 Hz, 1H), 2.62 (s, 3H), 2.25 (s, 3H), 1.64–1.31 (m, 10H), 1.31–1.10 (m, 2H), 1.00–0.50 (m, 24H). ^13^C NMR (151 MHz, DMSO-*d*_6_) δ 174.2, 171.9, 171.3, 170.9, 170.4, 138.1, 137.4, 130.2, 128.4, 127.3, 126.7, 57.7, 53.6, 51.5, 51.1, 48.2, 41.4, 38.7, 37.8, 37.2, 35.2, 31.2, 29.8, 26.8, 25.2, 24.8, 24.8, 24.7, 23.5, 23.4, 23.2, 22.2, 22.0, 21.9, 21.4. HRMS (ESI) *m*/*z* calcd. for C_36_H_59_N_5_O_5_ [M + Na]^+^: 664.4408; found: 664.4412.
*Cyclo(Me-L-Leu-L-Leu-3-Me-D-Phe-Me-L-Leu-L-Leu)* **Gala24**. The product **10x** (145 mg, 0.22 mmol, 1 equiv) was dissolved in MeCN (4.3 mM) and reacted according to route E for macrolactamization. PyBOP (229 mg, 0.44 mmol, 2 equiv), DMAP (80 mg, 0.66 mmol, 3 equiv). The crude residue was purified by column chromatography (hexane/EtOAc = 60:40, *v*/*v*), obtaining compound **Gala24** (71 mg, 0.11 mmol, 50% yield) as a white solid. [α]^25^_D_ = −65.6 (*c* = 1.0, CHCl_3_). m.p. 204–205 °C. ^1^H NMR (600 MHz, DMSO-*d*_6_) δ 7.58 (t, *J* = 9.2 Hz, 2H), 7.45 (d, *J* = 8.1 Hz, 1H), 7.31–6.75 (m, 4H), 5.03 (dd, *J* = 9.8, 5.9 Hz, 1H), 4.83 (q, *J* = 7.9 Hz, 1H), 4.74 (q, *J* = 7.6 Hz, 1H), 4.57 (dd, *J* = 10.3, 5.6 Hz, 1H), 4.11 (q, *J* = 7.6 Hz, 1H), 3.02–2.97 (m, 1H), 2.96 (s, 3H), 2.71 (dd, *J* = 13.2, 6.2 Hz, 1H), 2.62 (s, 3H), 2.24 (s, 3H), 1.70–1.12 (m, 12H), 1.00–0.53 (m, 24H). ^13^C NMR (151 MHz, DMSO-*d*_6_) δ 174.1, 171.9, 171.3, 170.9, 170.4, 135.6, 135.0, 129.5, 129.1, 57.67, 53.6, 51.6, 51.2, 48.2, 41.4, 38.8, 37.4, 37.2, 35.3, 31.2, 29.9, 25.2, 24.9, 24.8, 24.6, 23.5, 23.4, 23.4, 23.2, 22.2, 22.0, 21.9, 21.1. HRMS (ESI) *m*/*z* calcd. for C_36_H_59_N_5_O_5_ [M + Na]^+^: 664.4408; found: 664.4412.

### 3.2. Biological Evaluations

#### 3.2.1. Cell Culture

A549, K562, and 293T cells were purchased from the Cell Resource Center of the Institute of Basic Medicine, Chinese Academy of Medical Sciences. Both A549 cells and K562 cells were cultured in RPMI (full name: Roswell Park Memorial Institute) 1640 medium containing 10% FBS (full name: fetal bovine serum, product number: C04001-500, Vivacell, Shanghai, China) and 1% p/s (full name: penicillin-streptomycin, product number: 15140122, Gbico, Waltham, MA, USA) for culture. The 293T cells were all cultured in DMEM (full name: Dulbecco’s Modified Eagle Medium; cat. no.: C3113-0500, Vivacell, ShanghaiChina; containing 10% FBS and 1% p/s) for cultivation. These cells were cultured at 37 °C in an incubator containing 5% CO_2_.

#### 3.2.2. CCK-8 Experiment

Multiple types of cells were seeded into 96-well plates and treated with different concentrations of **Gala01**~**Gala26** for 48 h after they returned to normal. Then, the relative activity of the cells was detected according to the instructions of the CCK-8 kit (item number: MA0218, Meilunbio, Dalian, China) and analyzed and calculated using GraphPad Prism 8.0.1 software.

## 4. Conclusions

In conclusion, using a traditional “3 + 2” synthesis approach, we have optimized the final macrolactamization cyclization step. Galaxamide and 23 analogs were designed and synthesized by substituting *D*-leucine with other *D*- or *L*-proteinogenic amino acid at different positions. In vitro antitumor activities were investigated utilizing the CCK-8 assay against two human tumor cell lines, A549 and K562, and one human normal cell line (293T). With the two human tumor cell lines tested, broad-spectrum antitumor activity was observed for most of galaxamide analogs, in particular compound **Gala04**, which showed an outstanding antitumor activity on K562. Our research will yield a database that may be used to rationally design galaxamide analogs with enhanced anticancer activity, which holds promise for the creation of anticancer medications.

## Figures and Tables

**Table 1:** Optimization of macrolactamization conditions ^a^.

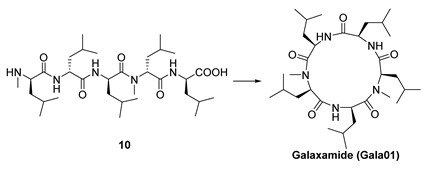				
Entry	Coupling Reagent	Solvent	Base	Yield
1	HATU	DMF	DIPEA	28%
2	T_3_P	DMF	DIPEA	33%
3	DPPA	DMF	DIPEA	37%
4	FDPP	DMF	DIPEA	41%
5	PyBOP	CH_3_CN	DMAP	52%

^a^ Conditions: **10** (0.20 mmol), coupling reagent (0.30 mmol), and base (0.60 mmol) in solvent (0.1 M concentration) at 20 °C.

**Table 2:** Activity of galaxamide and its analogs ^a^.

Compounds	IC_50_		
	A549	K562	293T
(−)-β-elemene	>100.0	>100.0	>100.0
**Gala01**	14.0	>80.0	19.9
**Gala02**	>20.0	8.6	NA
**Gala03**	>20.0	>80.0	NA
**Gala04**	19.0	4.2	16.3
**Gala05**	>20.0	10.2	NA
**Gala06**	>20.0	28.8	NA
**Gala07**	>20.0	>80	NA
**Gala0** **8**	18.5	16.8	16.9
**Gala** **09**	>20.0	14.9	NA
**Gala1** **0**	>20.0	27.2	NA
**Gala1** **1**	19.5	33.8	13.31
**Gala1** **2**	17.0	61.8	14.1
**Gala1** **3**	>20.0	>80.0	NA
**Gala1** **4**	20.0	29.6	22.2
**Gala1** **5**	>20.0	13.0	NA
**Gala1** **6**	>20.0	>80.0	NA
**Gala1** **7**	>20.0	77.7	NA
**Gala** **18**	15.5	24.2	14.1
**Gala** **19**	>20.0	75.2	NA
**Gala2** **0**	>20.0	>80.0	NA
**Gala2** **1**	>20.0	49.3	NA
**Gala2** **2**	19.8	8.5	12.9
**Gala2** **3**	>20.0	6.9	NA
**Gala2** **4**	>20.0	14.4	NA

^a^ NA: not determined due to low inhibitory activity.

## Data Availability

The data supporting this article are available in the Supplementary Materials. Additional data may be obtained from the corresponding author upon request (guoduliu@imu.edu.cn).

## Supplementary Materials

The following supporting information can be downloaded at: https://www.mdpi.com/article/10.3390/molecules30112362/s1, Supplementary Materials include ^1^H and ^13^CNMRspectra.
