# Supplementary material for: Design, Synthesis, and Antitumor Biological Evaluation of Galaxamide and Its Analogs
Source: Molecules. 2025 May 29;30(11):2362. doi: 10.3390/molecules30112362 (PMC12155888; doi:10.3390/molecules30112362)

Supporting Information

Design, Synthesis, and Antitumor Biological Evaluation of Galaxamide and Its Analogs

Yanyan Guo 1, Huixia Fan 2,3, Zhiqiang Luo 2,3,*, Jian Yang 2,3,* and Guodu Liu 1,4,*

1 Inner Mongolia Key Laboratory of Synthesis and Application of Organic Functional Molecules, College of Chemistry and Chemical Engineering, Inner Mongolia University (South Campus), 24 Zhaojun Road, Hohhot 010030, China

2 State Key Laboratory for Quality Ensurance and Sustainable Use of Dao-Di Herbs, National Resource Center for Chinese Materia Medica, China Academy of Chinese Medical Sciences, Beijing 100700, China

3 Research Center for Quality Evaluation of Dao-Di Herbs, Ganjiang New District, Nanchang 330000, China

4 Inner Mongolia Academy of Science and Technology, 2 Shandan Street, Hohhot 010010, China

* Correspondence: luozhiqiang@nrc.ac.cn (Z.L.); yangchem2012@163.com (J.Y.); guoduliu@imu.edu.cn (G.L.)

^{1}H NMR ($CDCl_3$, 600 MHz) and ^{13}C NMR ($CDCl_3$, 151 MHz) for **2a**

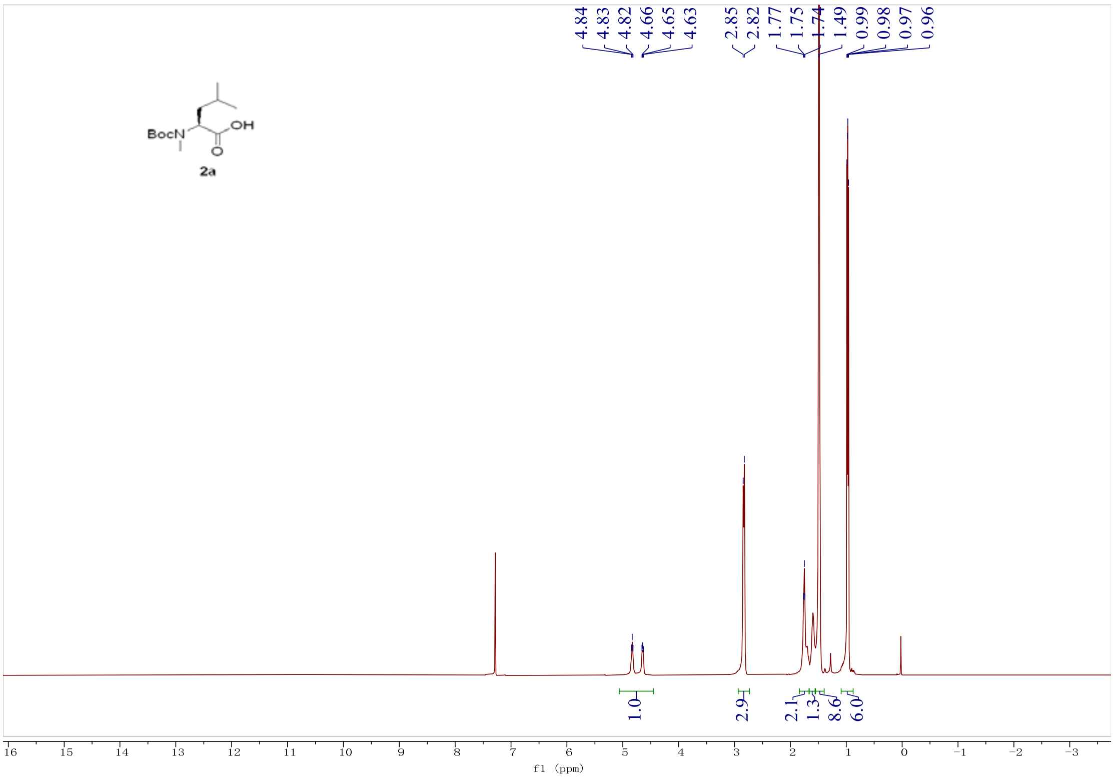

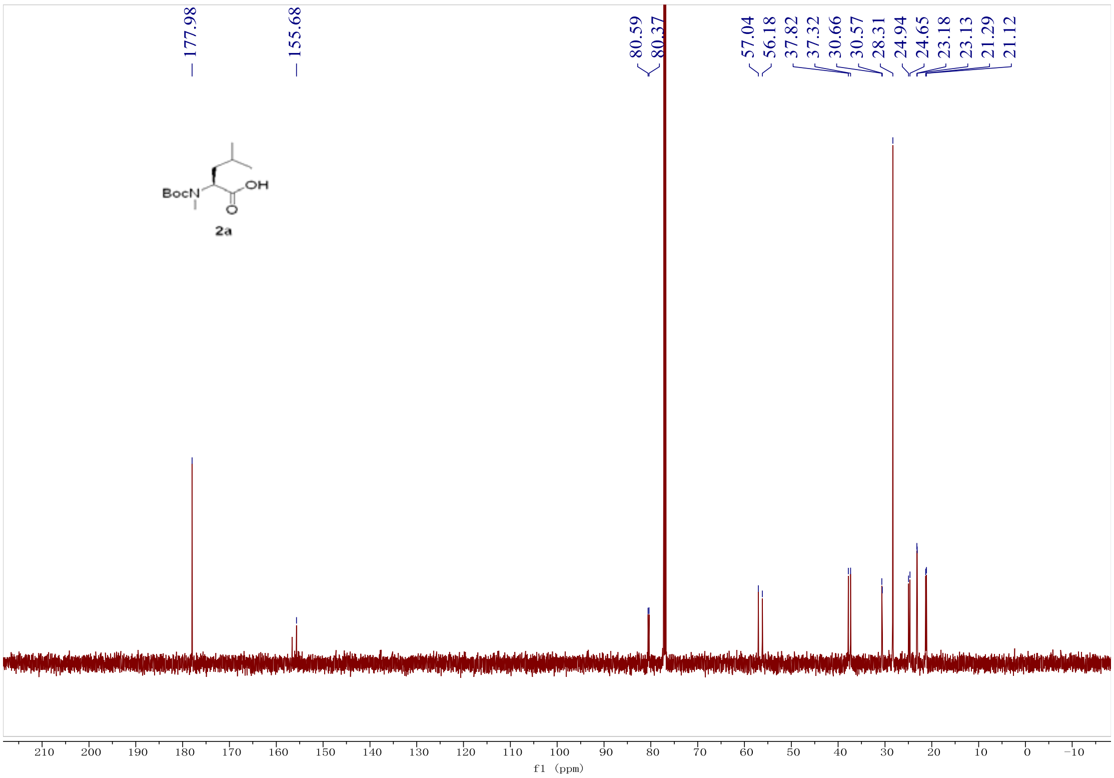

^{1}H NMR ($CDCl_3$, 600 MHz) and ^{13}C NMR ($CDCl_3$, 151 MHz) for **2b**

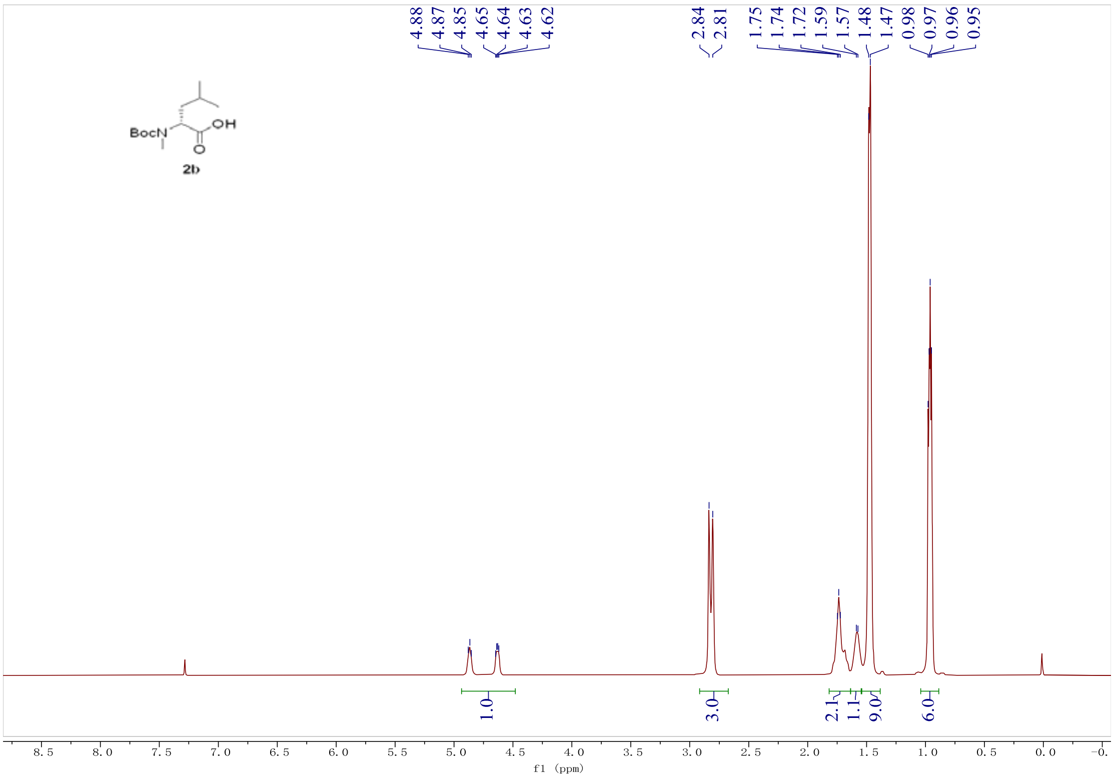

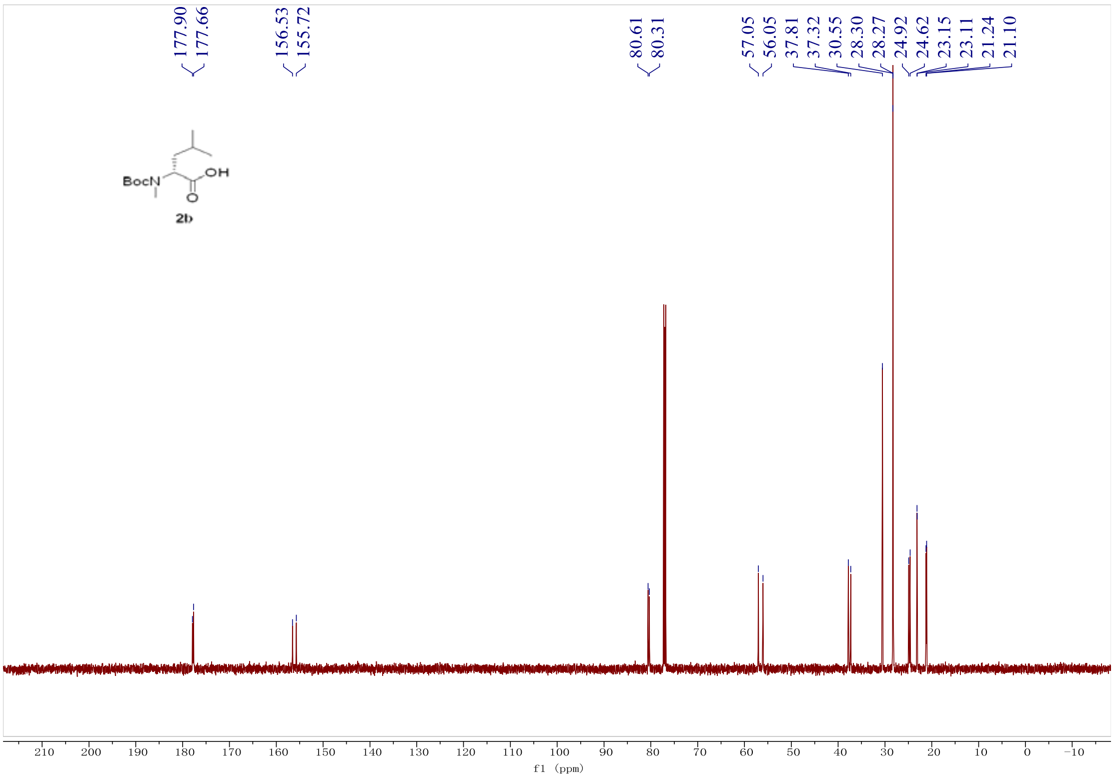

^{1}H NMR (DMSO, 600 MHz) and ^{13}C NMR (DMSO, 151 MHz) for **3a**

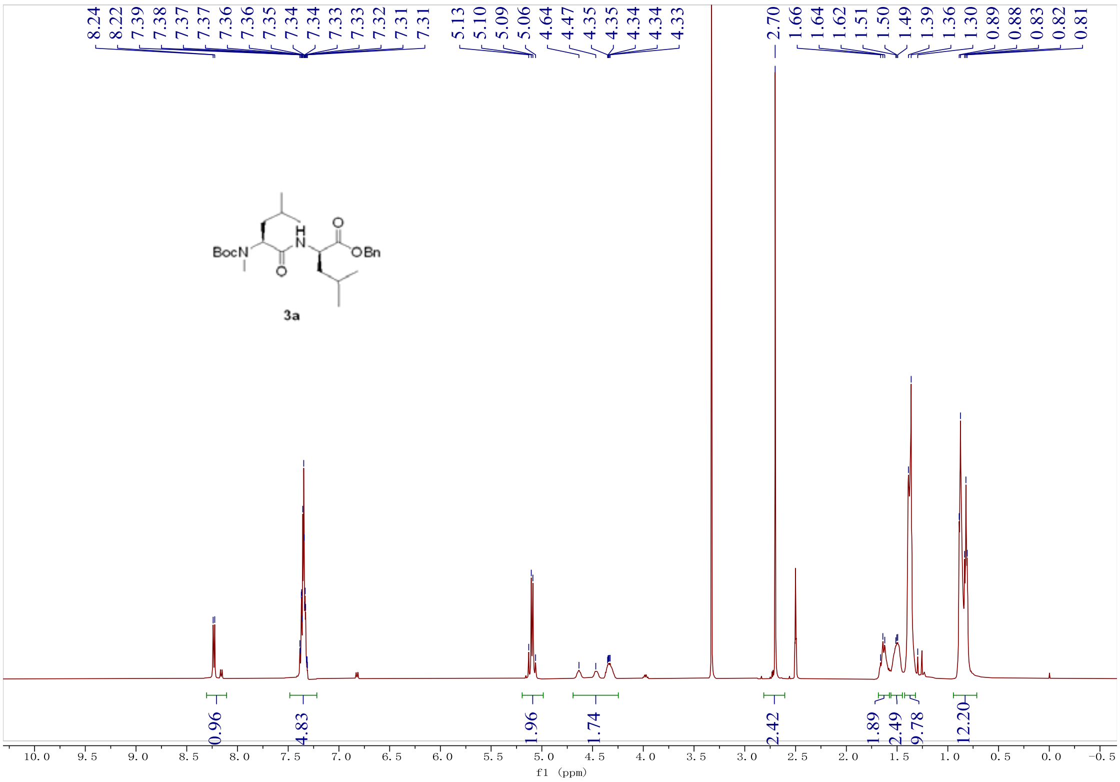

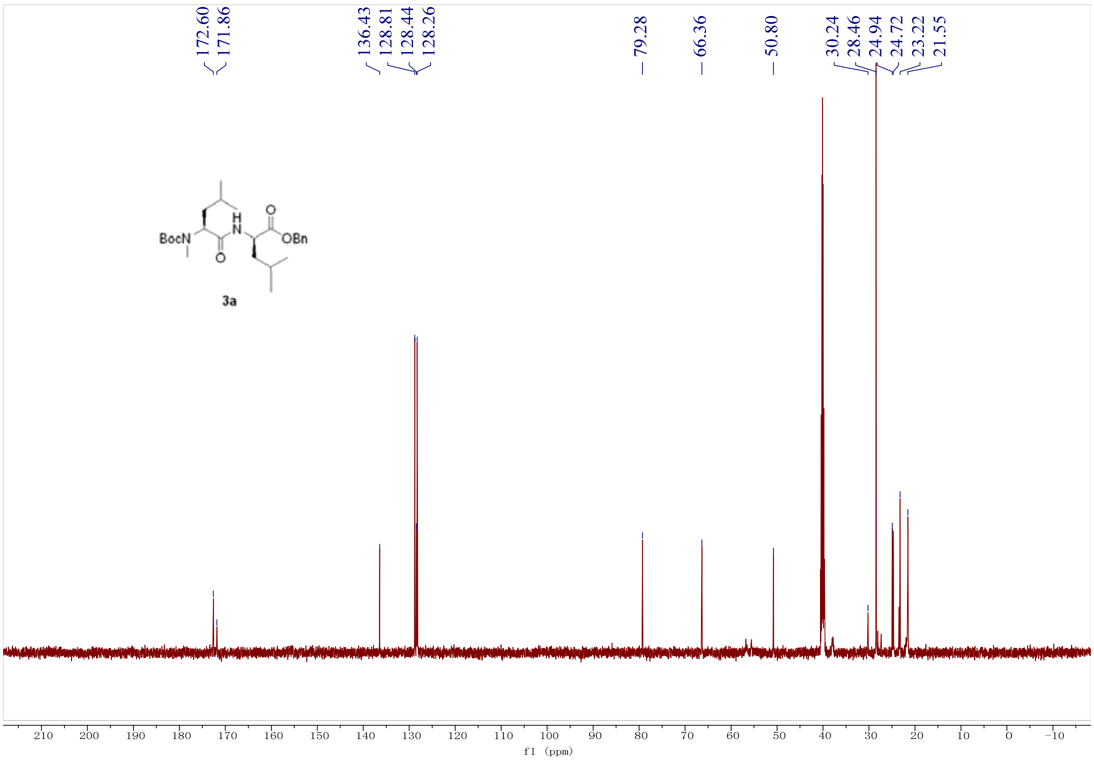

^{1}H NMR (DMSO, 600 MHz) and ^{13}C NMR (DMSO, 151 MHz) for **3b**

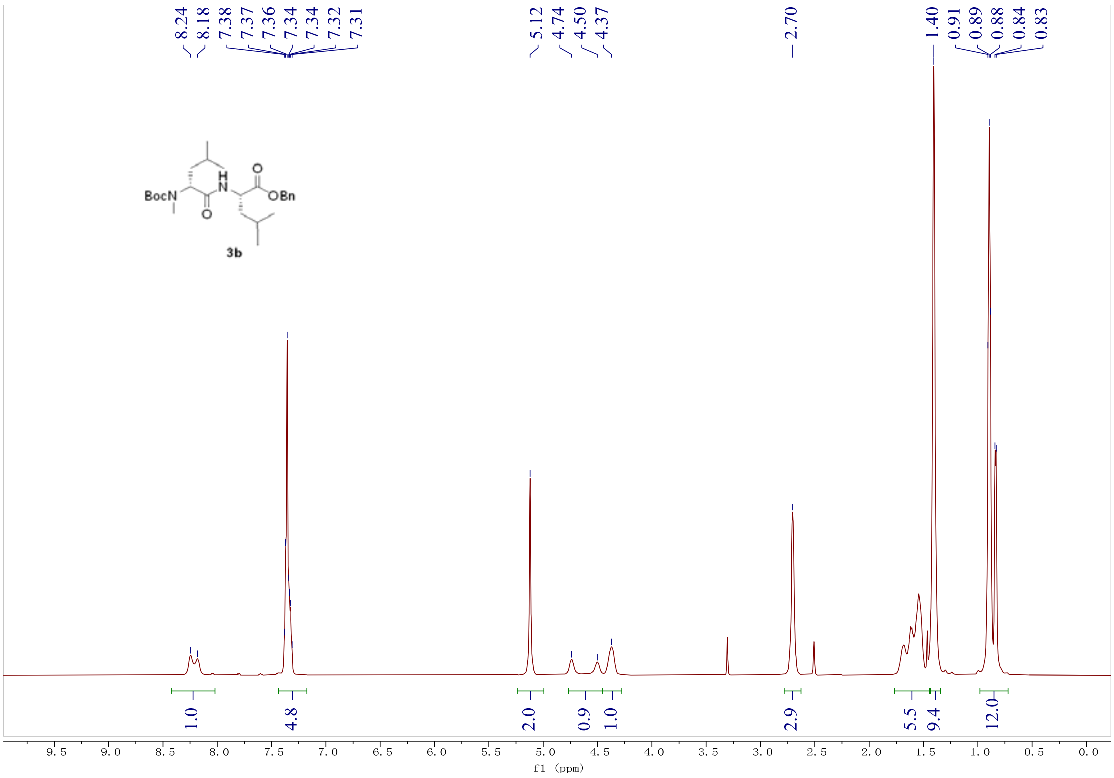

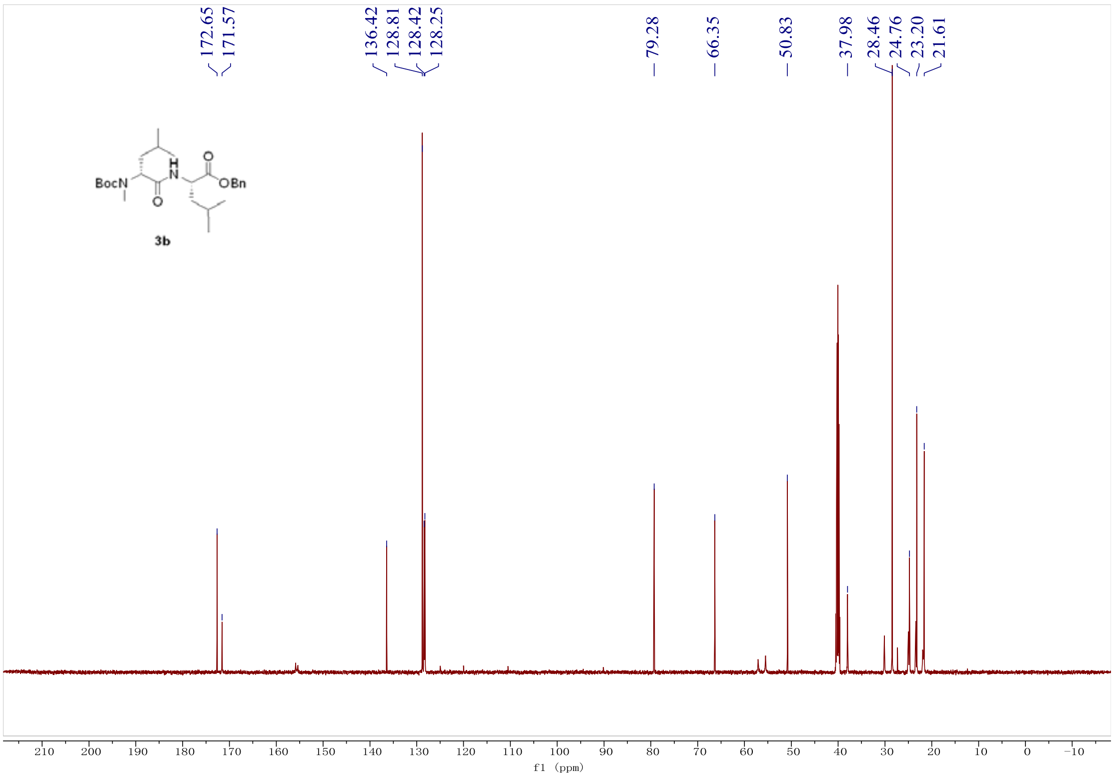

^{1}H NMR (DMSO, 600 MHz) and ^{13}C NMR (DMSO, 151 MHz) for **3c**

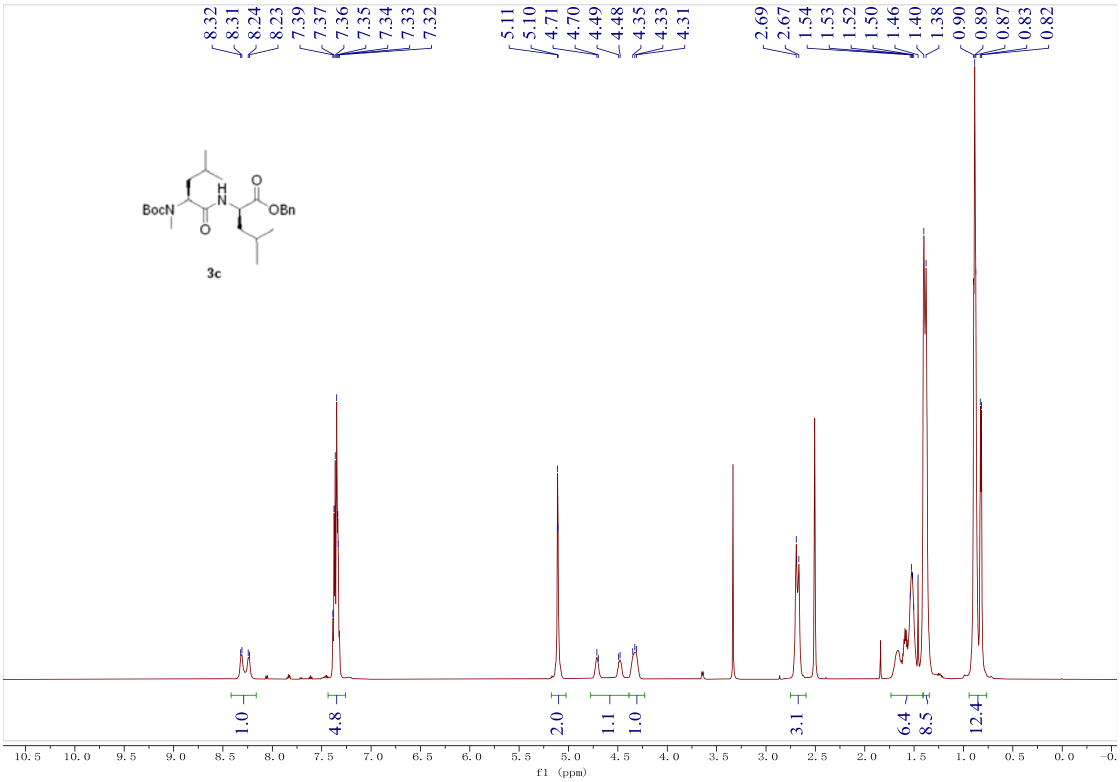

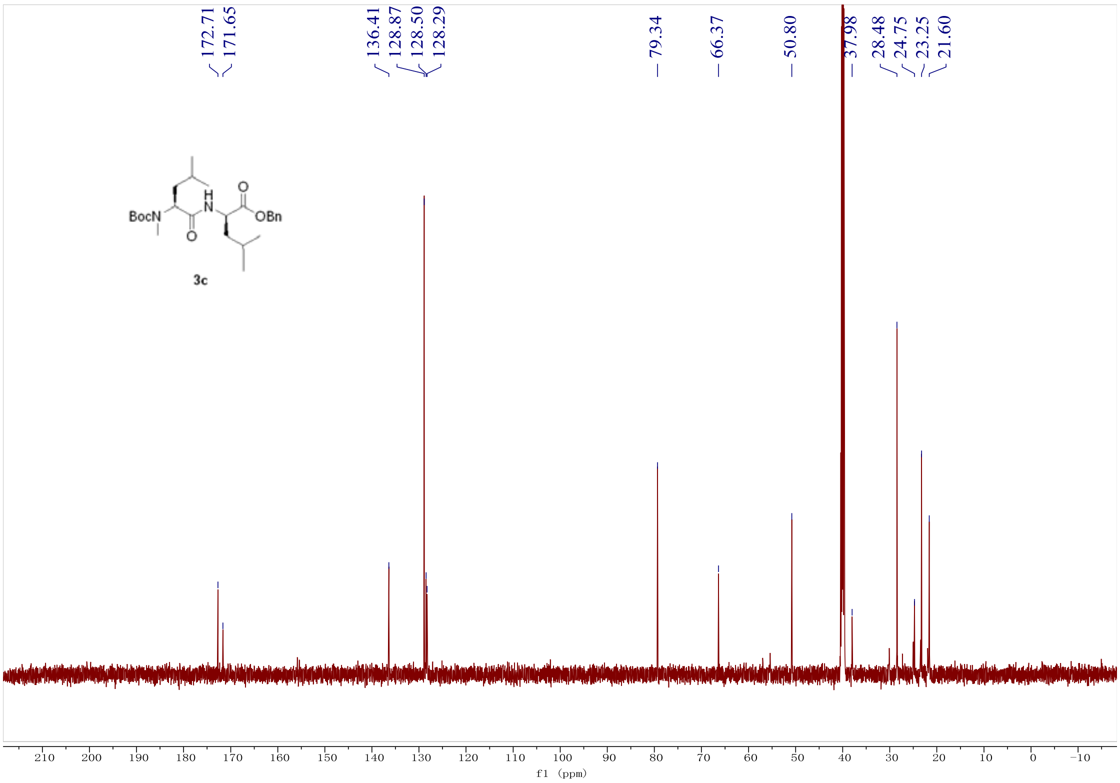

^{1}H NMR (DMSO, 600 MHz) and ^{13}C NMR (DMSO, 151 MHz) for **3d**

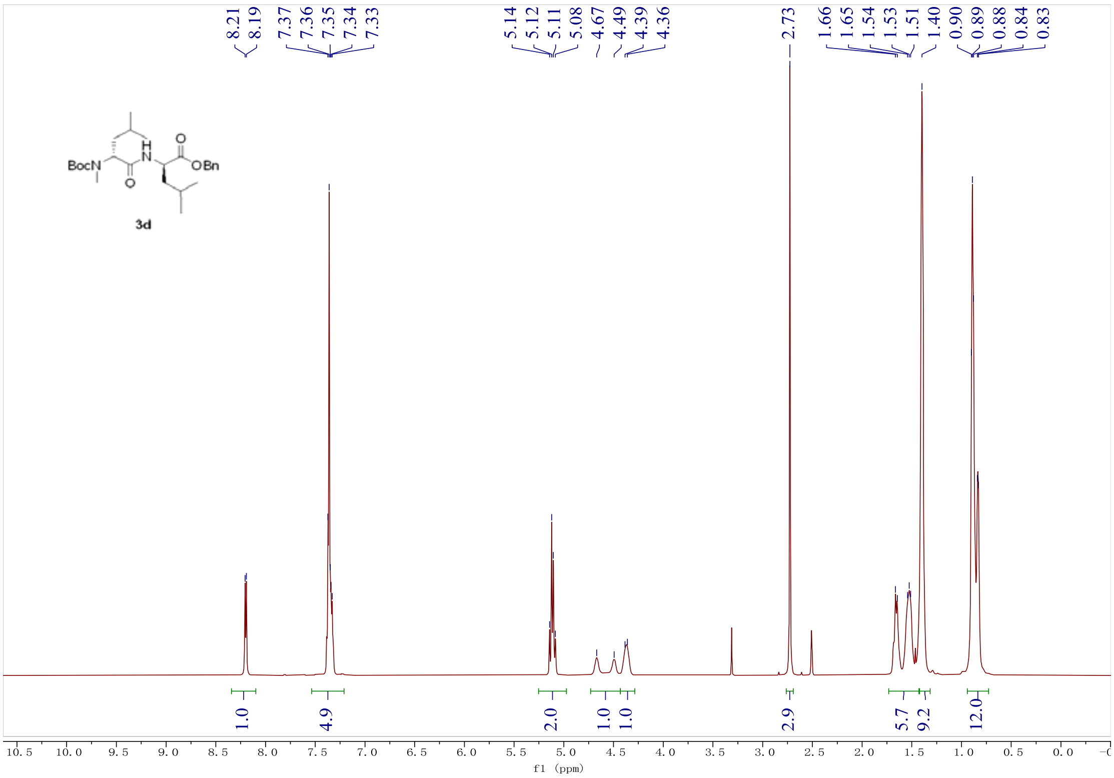

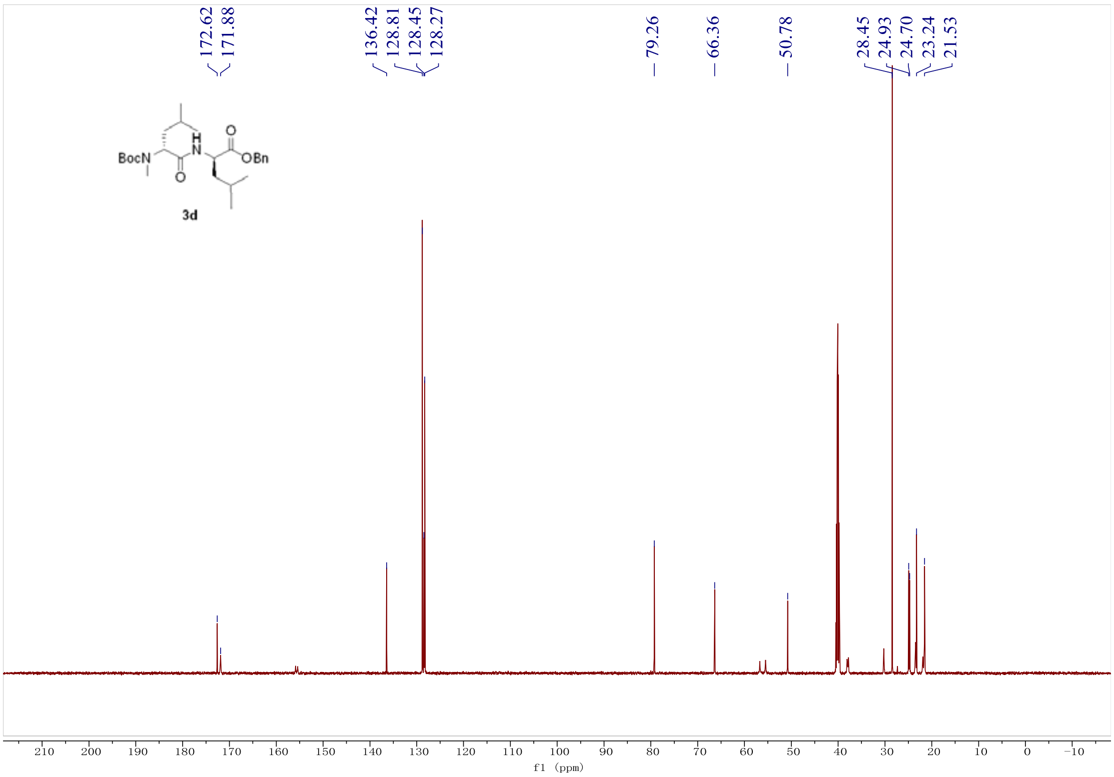

^{1}H NMR (DMSO, 600 MHz) and ^{13}C NMR (DMSO, 151 MHz) for **7a**

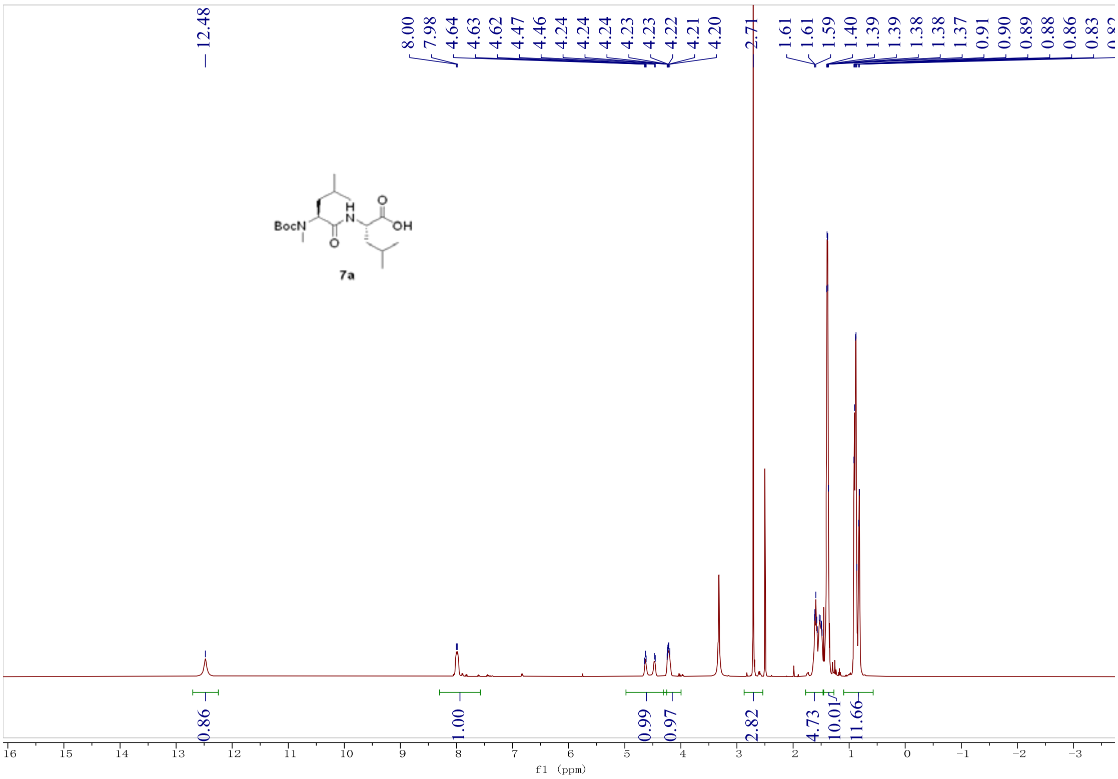

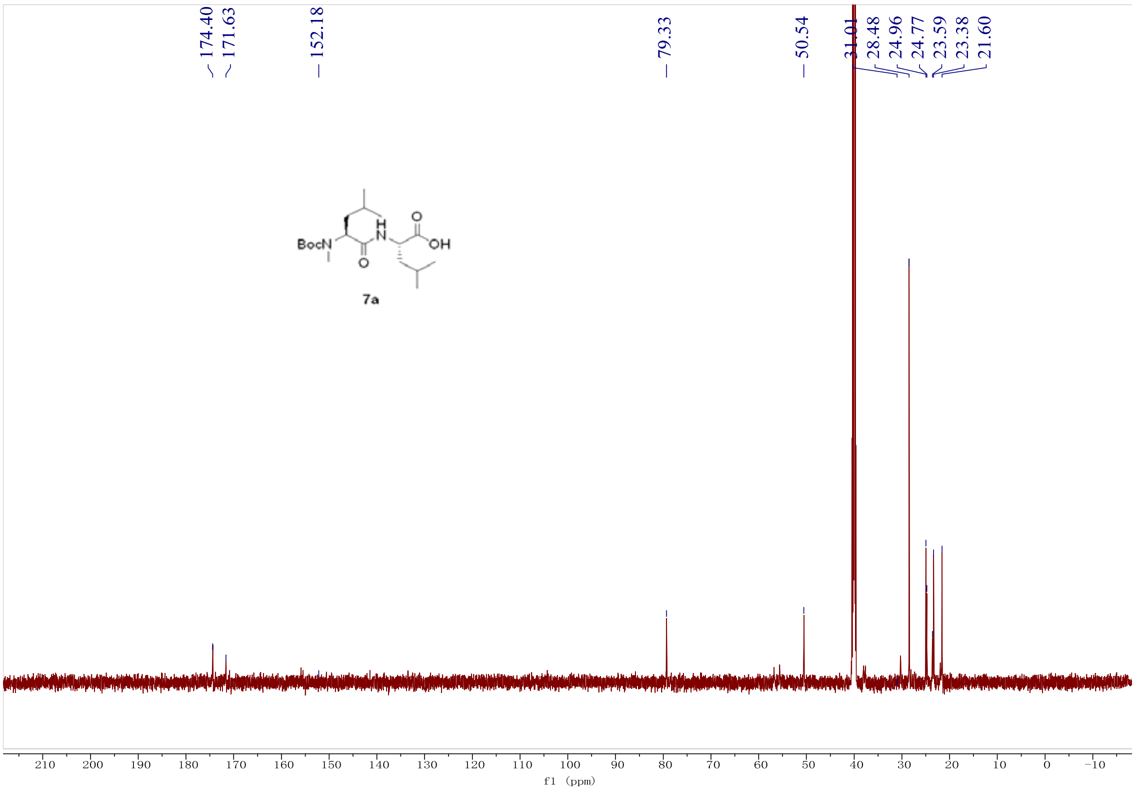

^{1}H NMR (DMSO, 600 MHz) and ^{13}C NMR (DMSO, 151 MHz) for **7b**

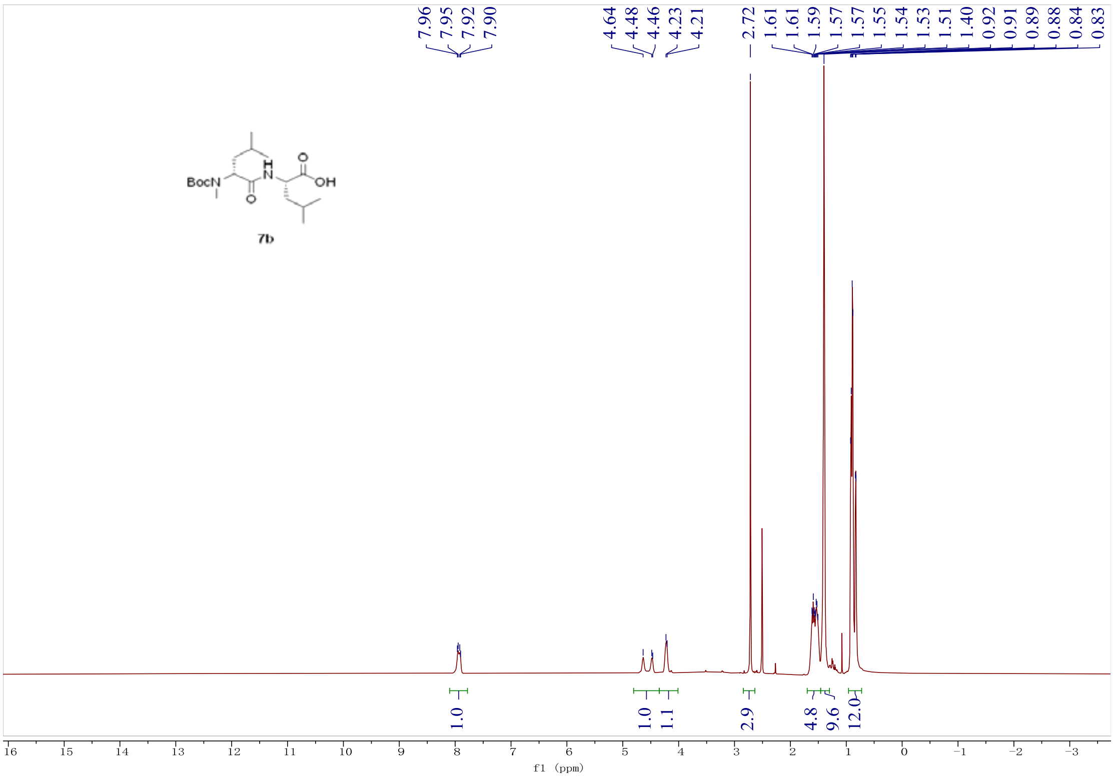

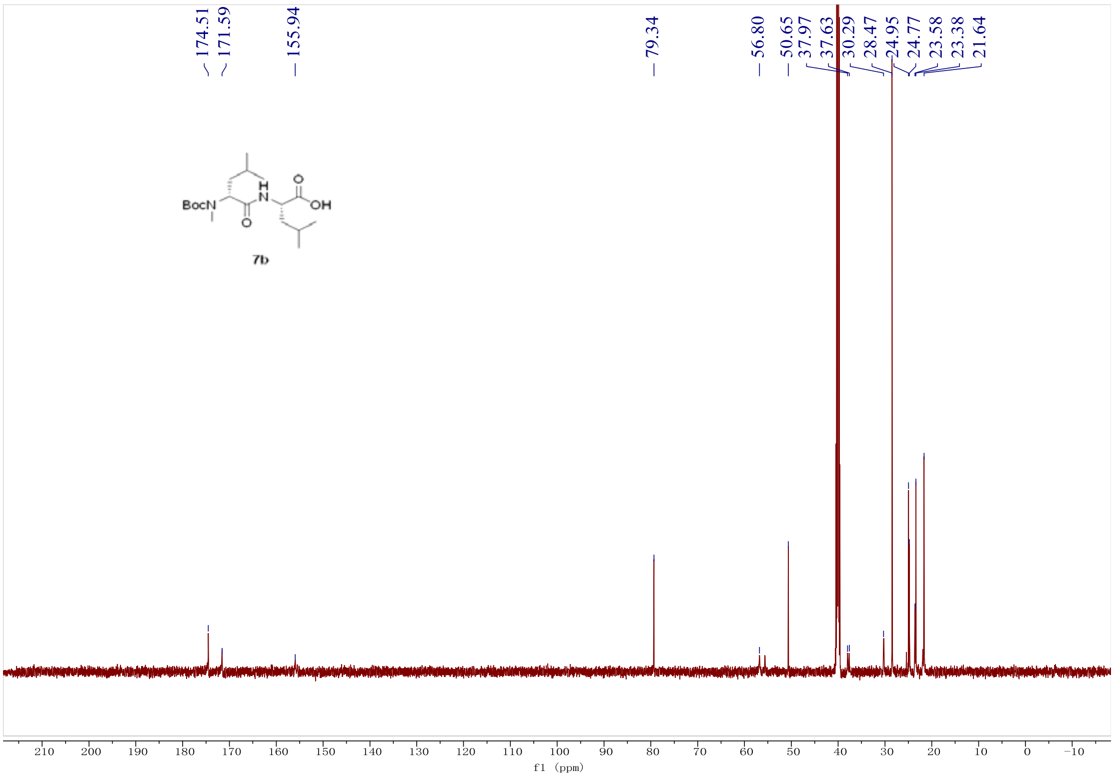

^{1}H NMR (DMSO, 600 MHz) and ^{13}C NMR (DMSO, 151 MHz) for **4c**

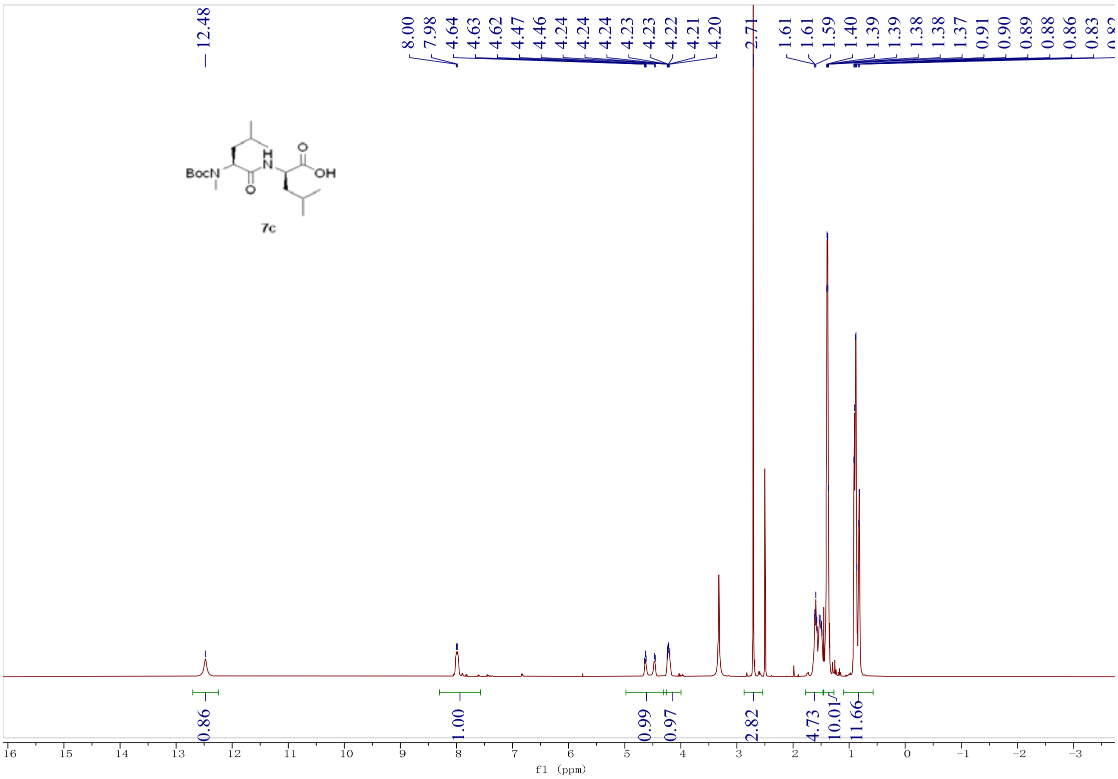

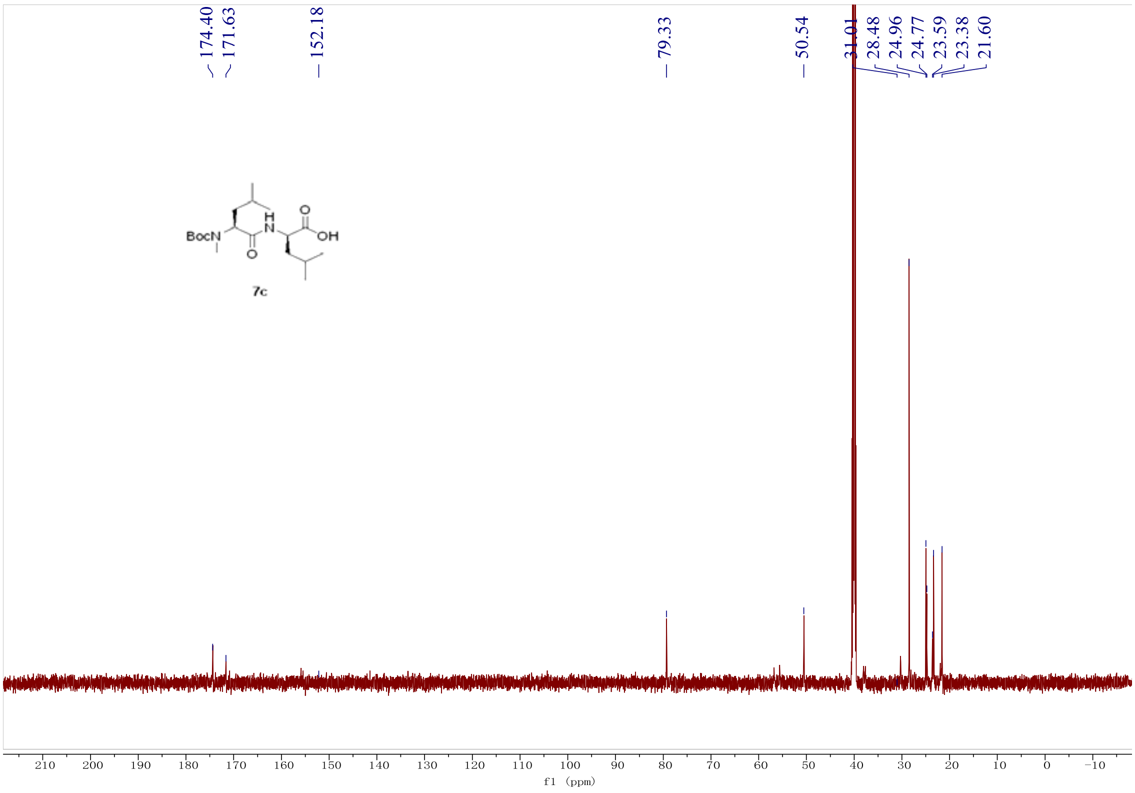

^{1}H NMR (DMSO, 600 MHz) and ^{13}C NMR (DMSO, 151 MHz) for **7d**

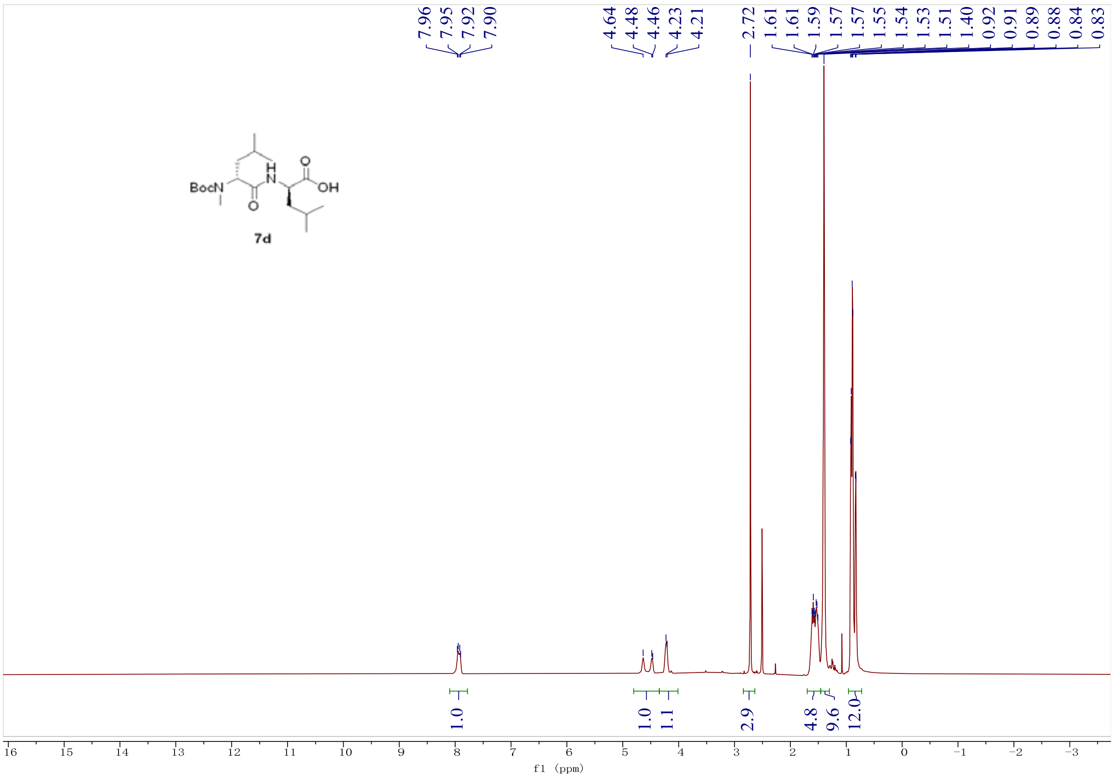

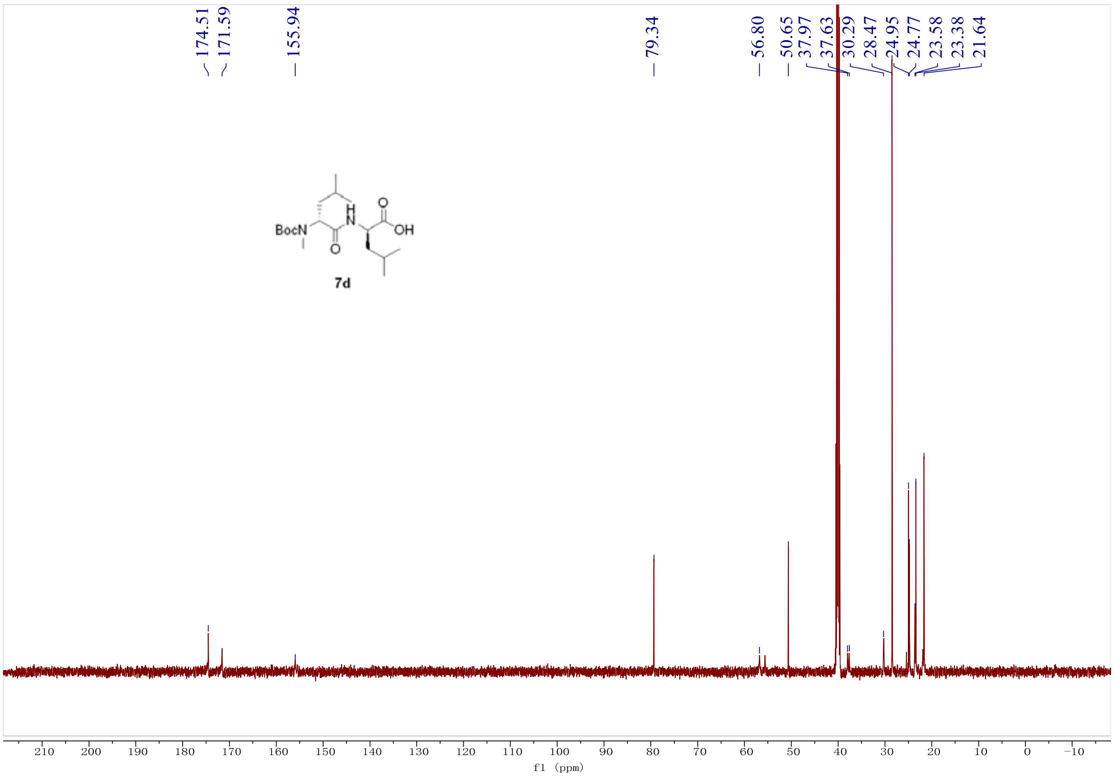

^1H NMR (DMSO, 600 MHz) and ^{13}C NMR (DMSO, 151 MHz) for **4a**

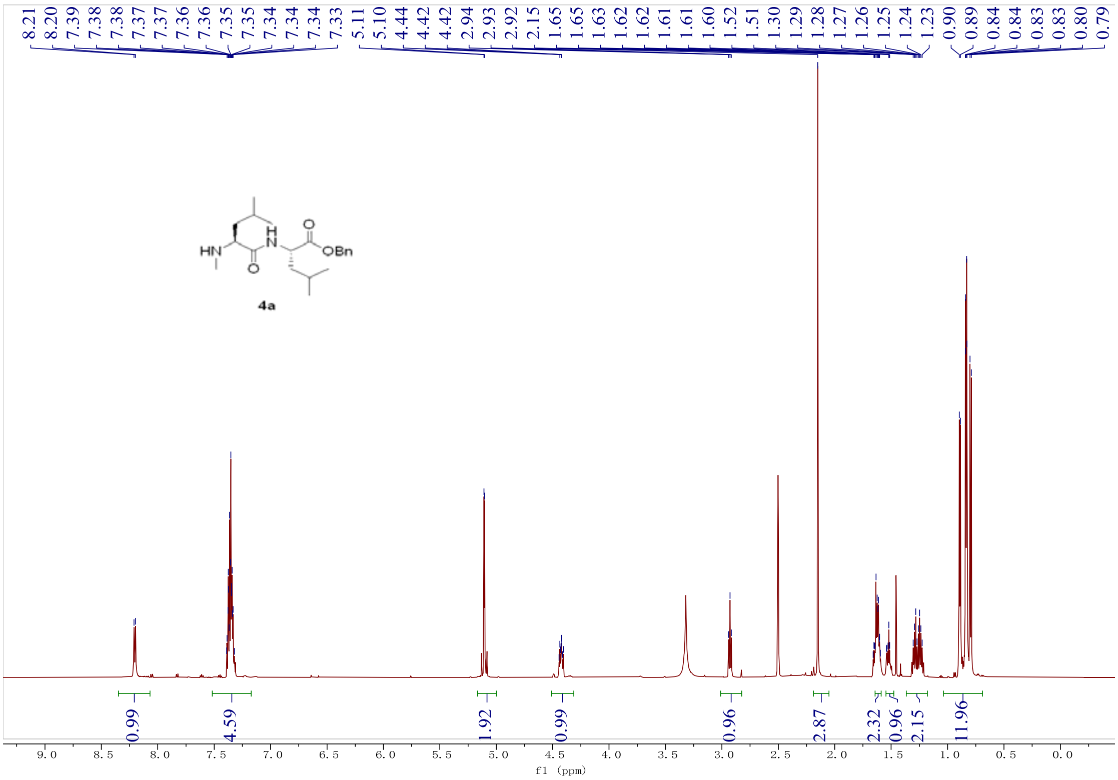

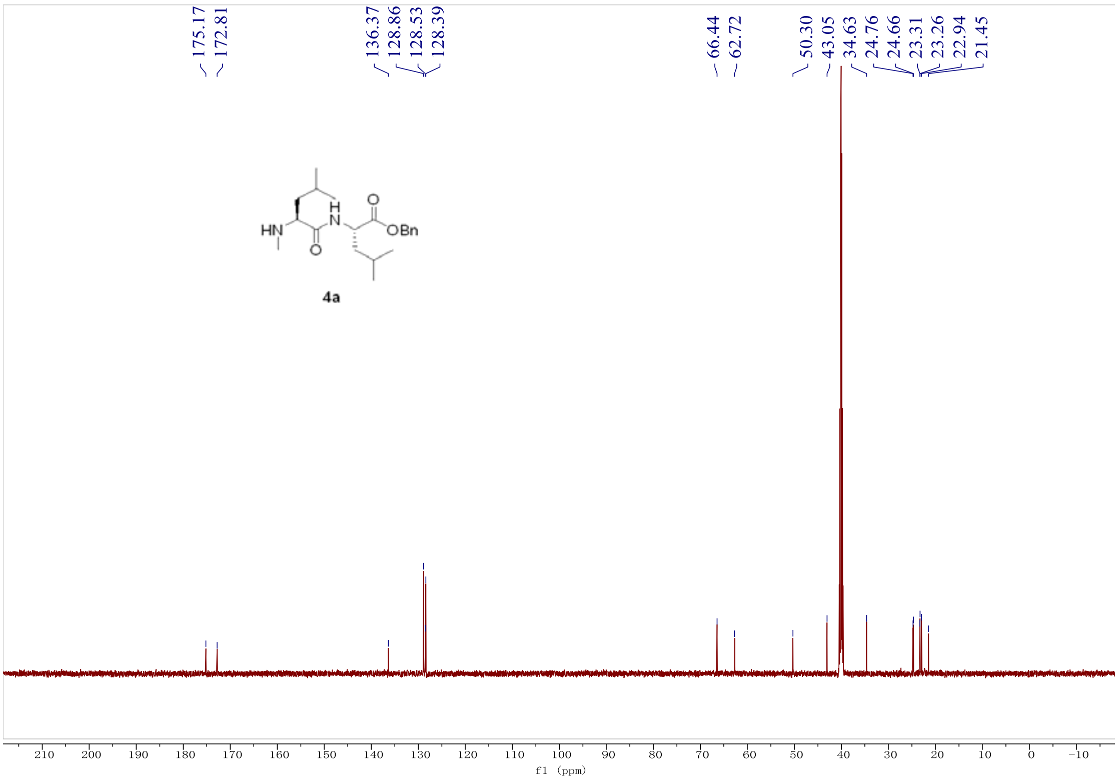

^1H NMR (DMSO, 600 MHz) and ^{13}C NMR (DMSO, 151 MHz) for **4b**

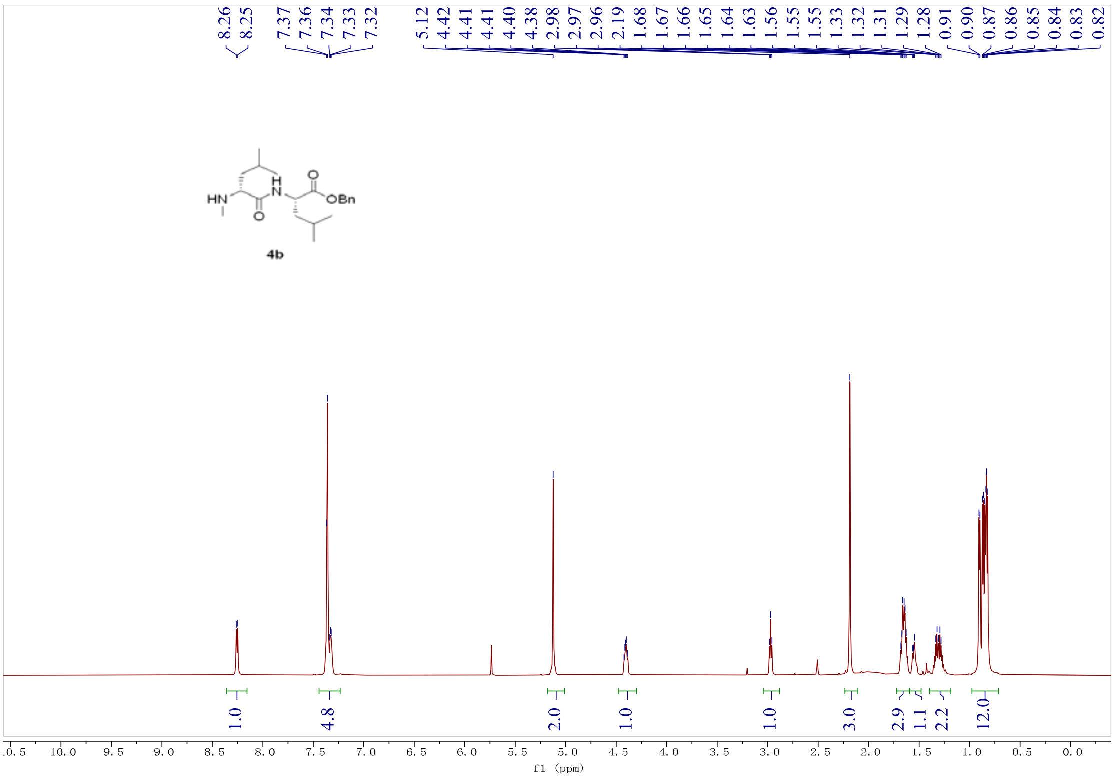

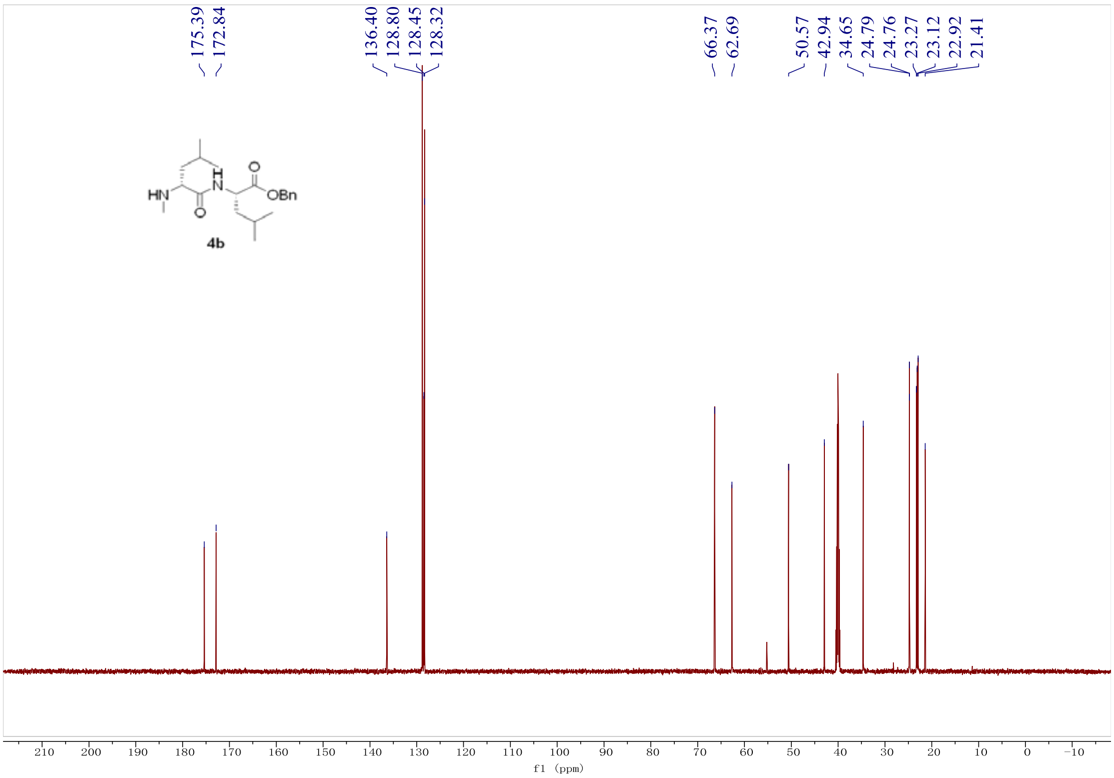

^{1}H NMR (DMSO, 600 MHz) and ^{13}C NMR (DMSO, 151 MHz) for **4c**

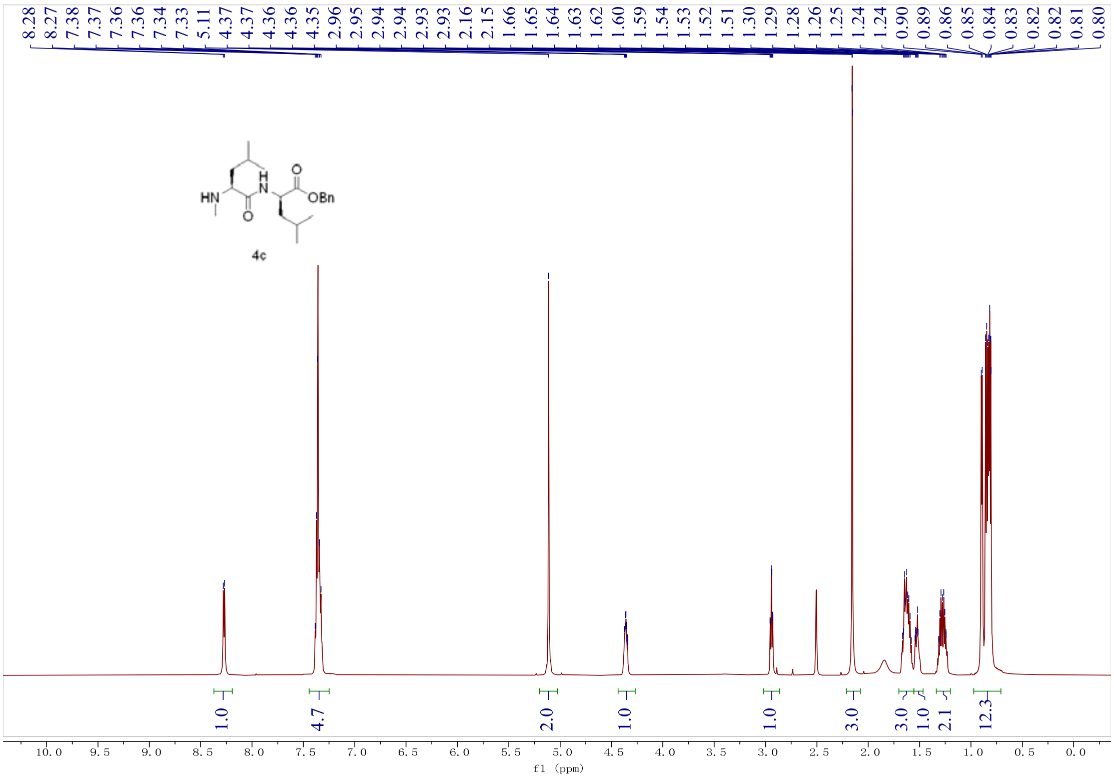

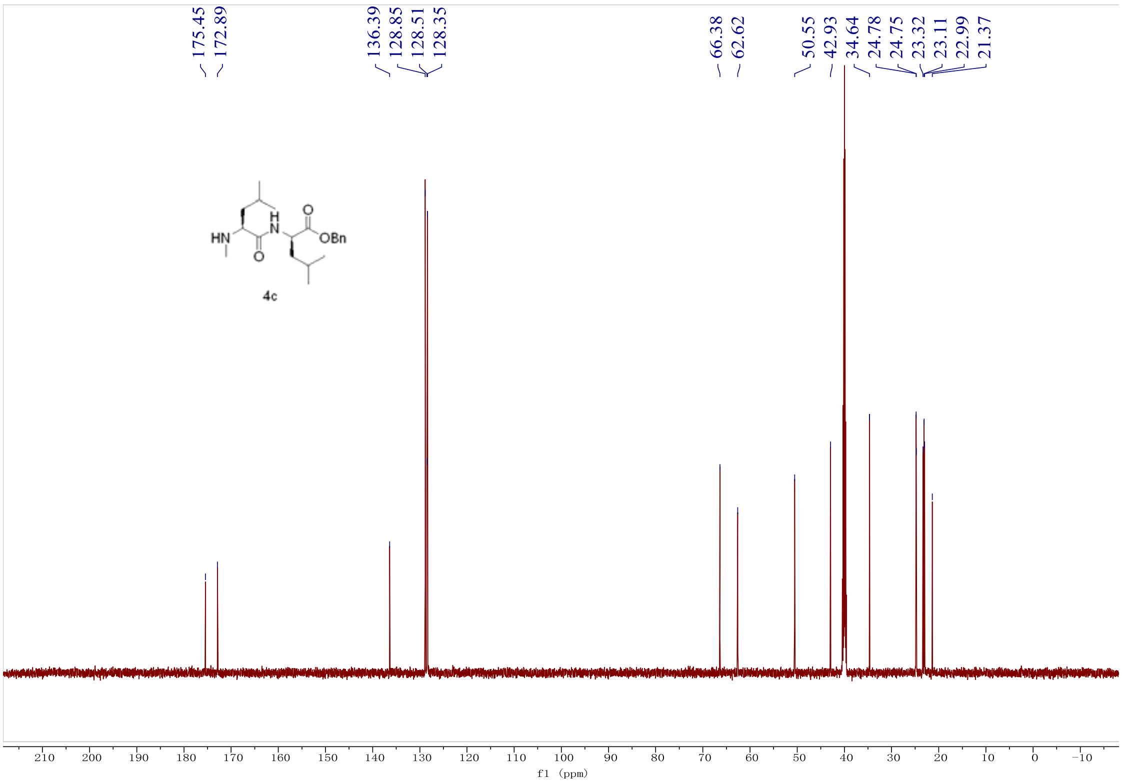

^{1}H NMR (DMSO, 600 MHz) and ^{13}C NMR (DMSO, 151 MHz) for **4d**

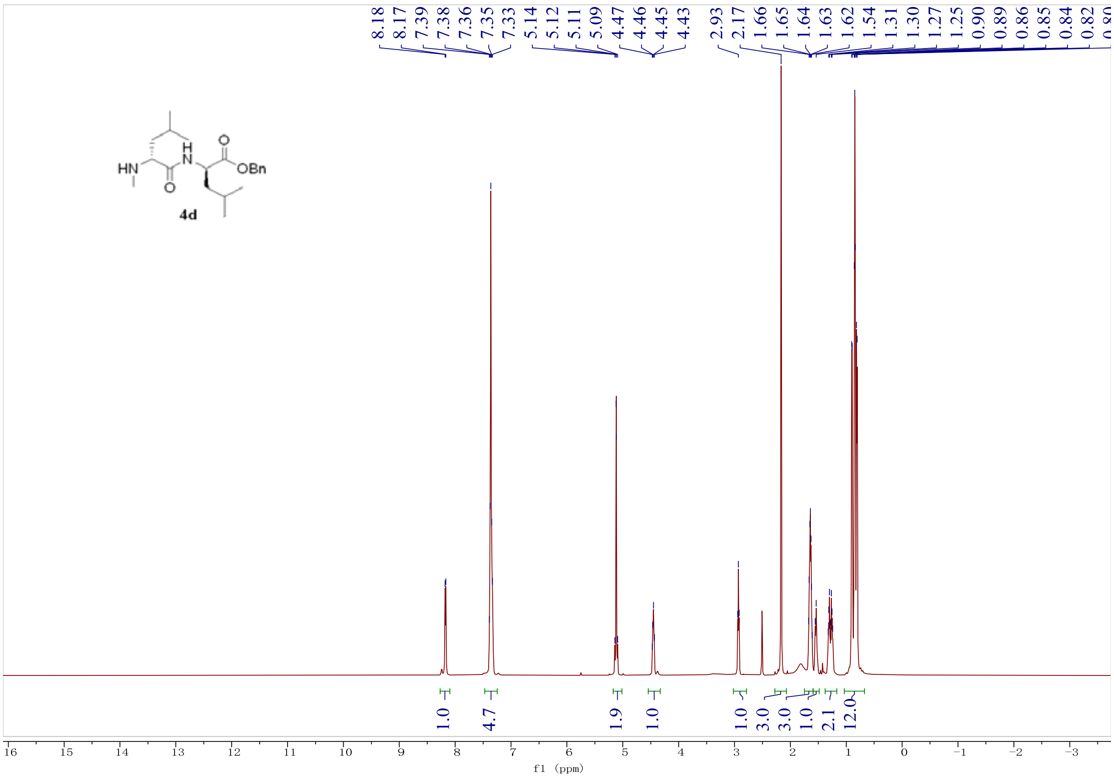

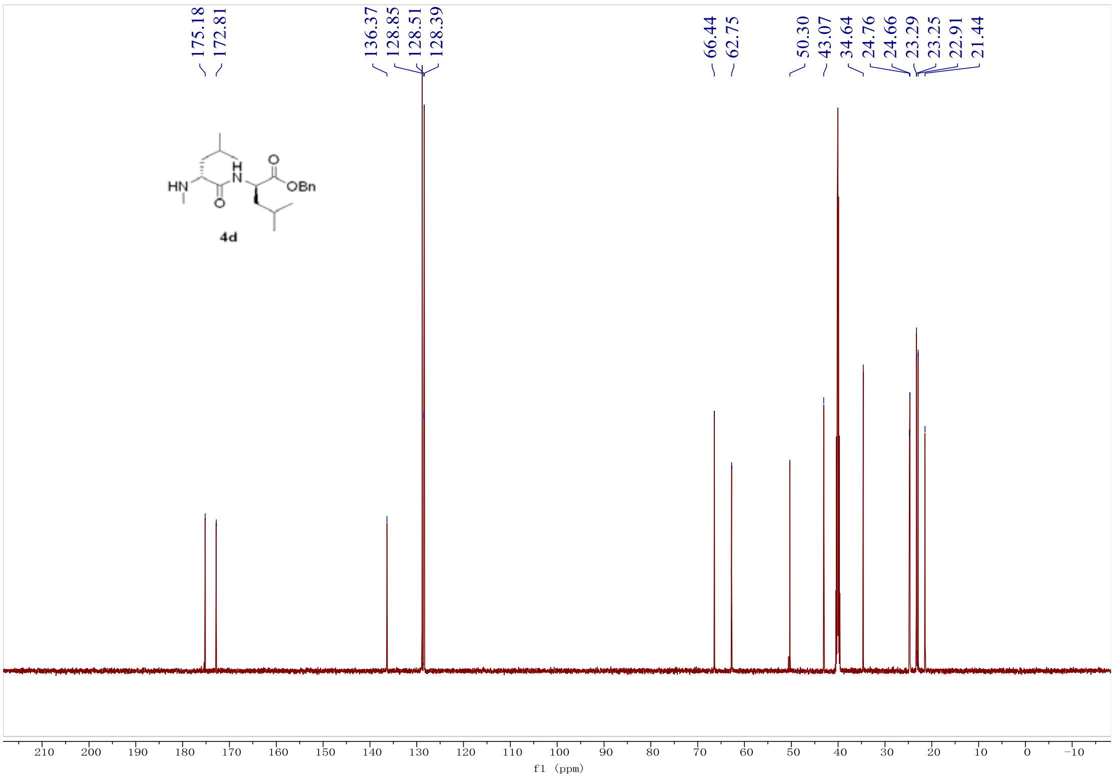

^{1}H NMR ($CDCl_3$, 600 MHz) and ^{13}C NMR ($CDCl_3$, 151 MHz) for **5a**

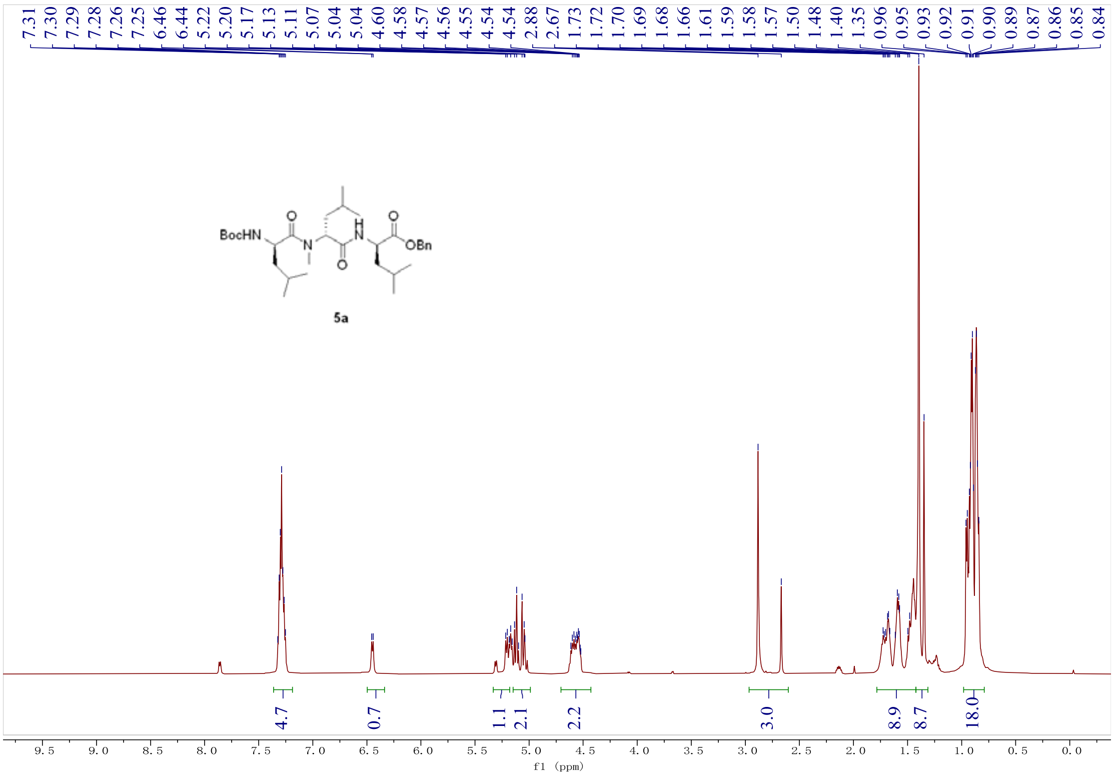

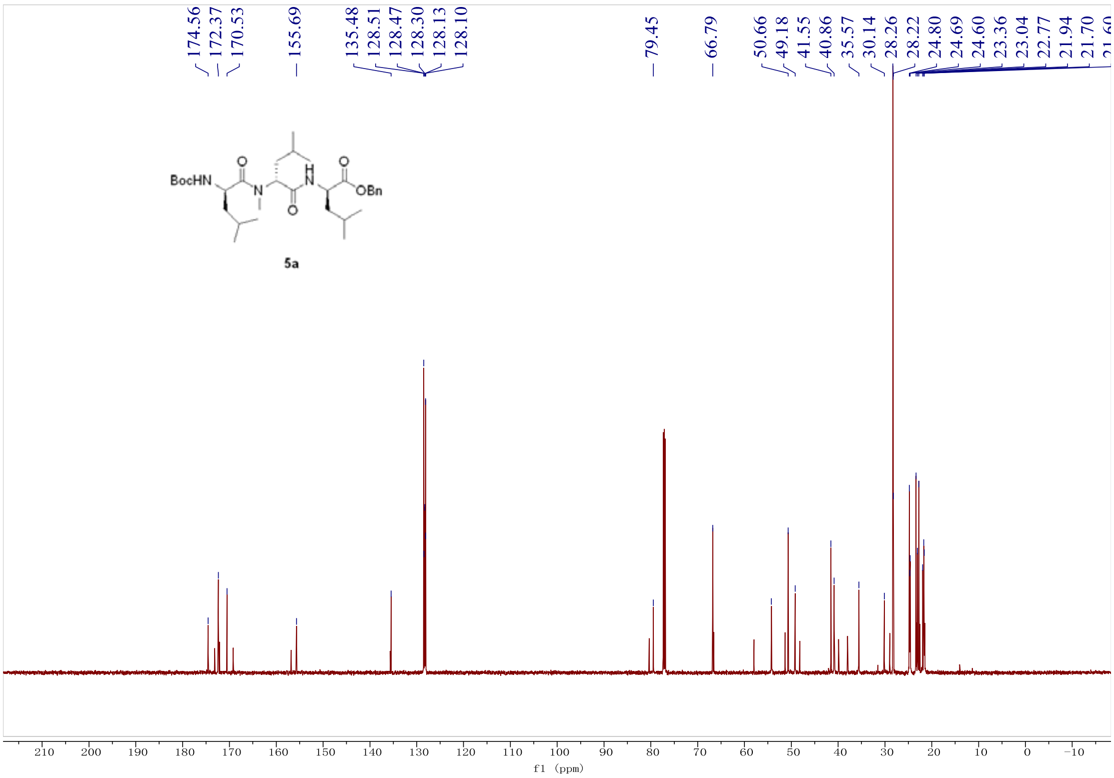

^{1}H NMR (DMSO, 600 MHz) and ^{13}C NMR (DMSO, 151 MHz) for **5b**

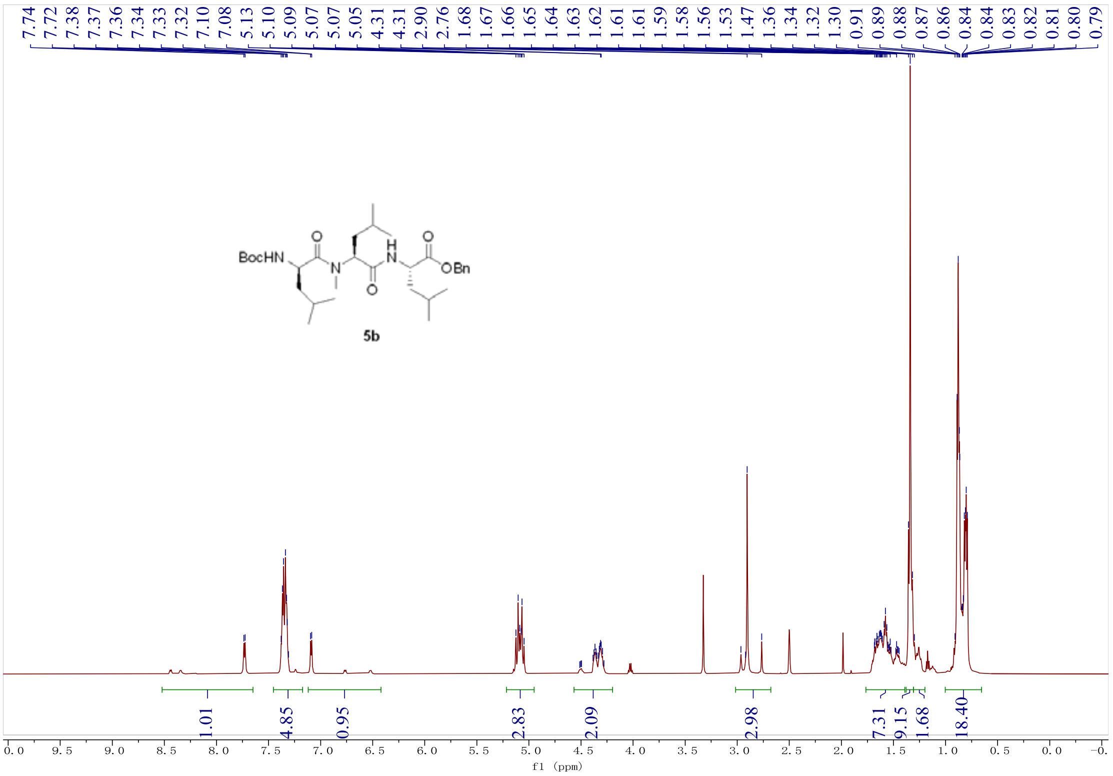

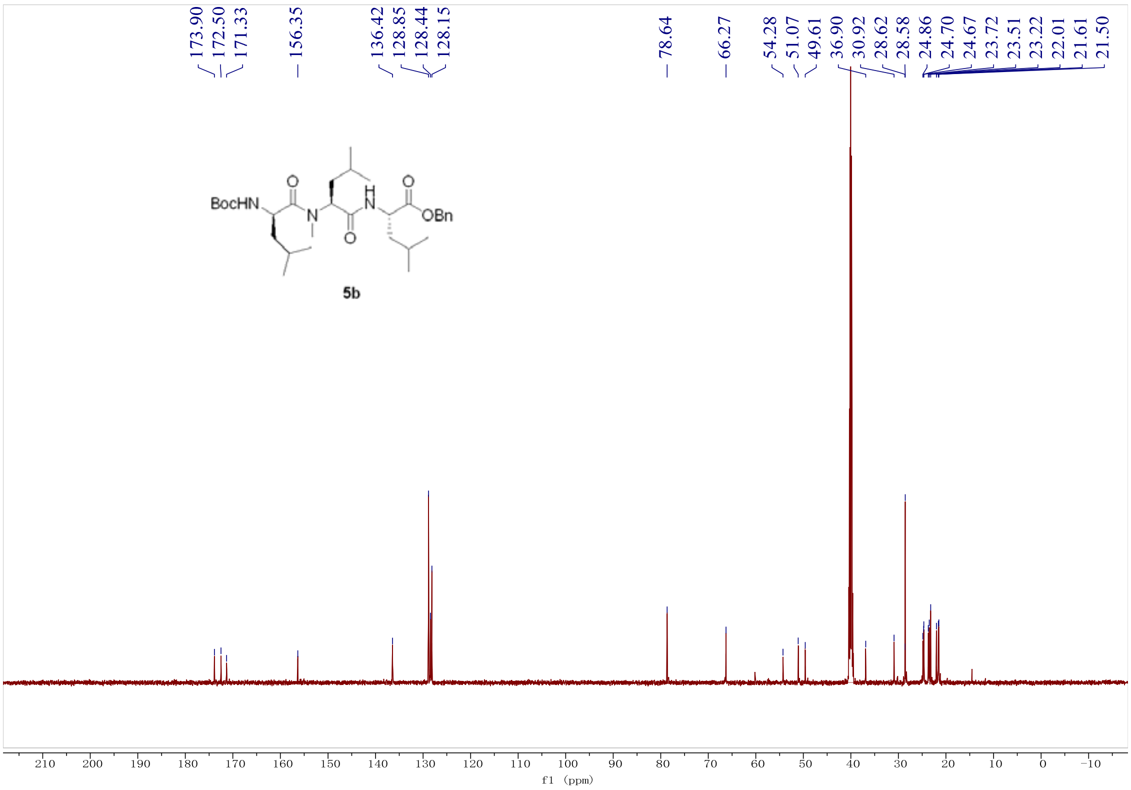

^{1}H NMR (DMSO, 600 MHz) and ^{13}C NMR (DMSO, 151 MHz) for **5c**

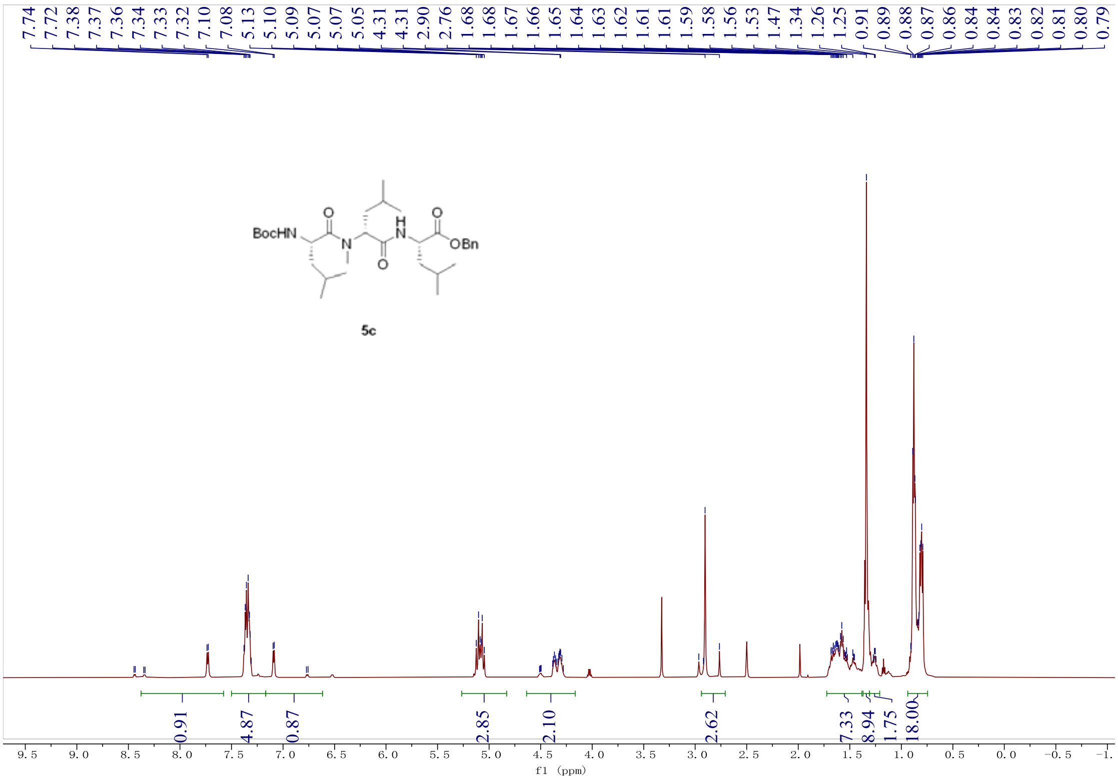

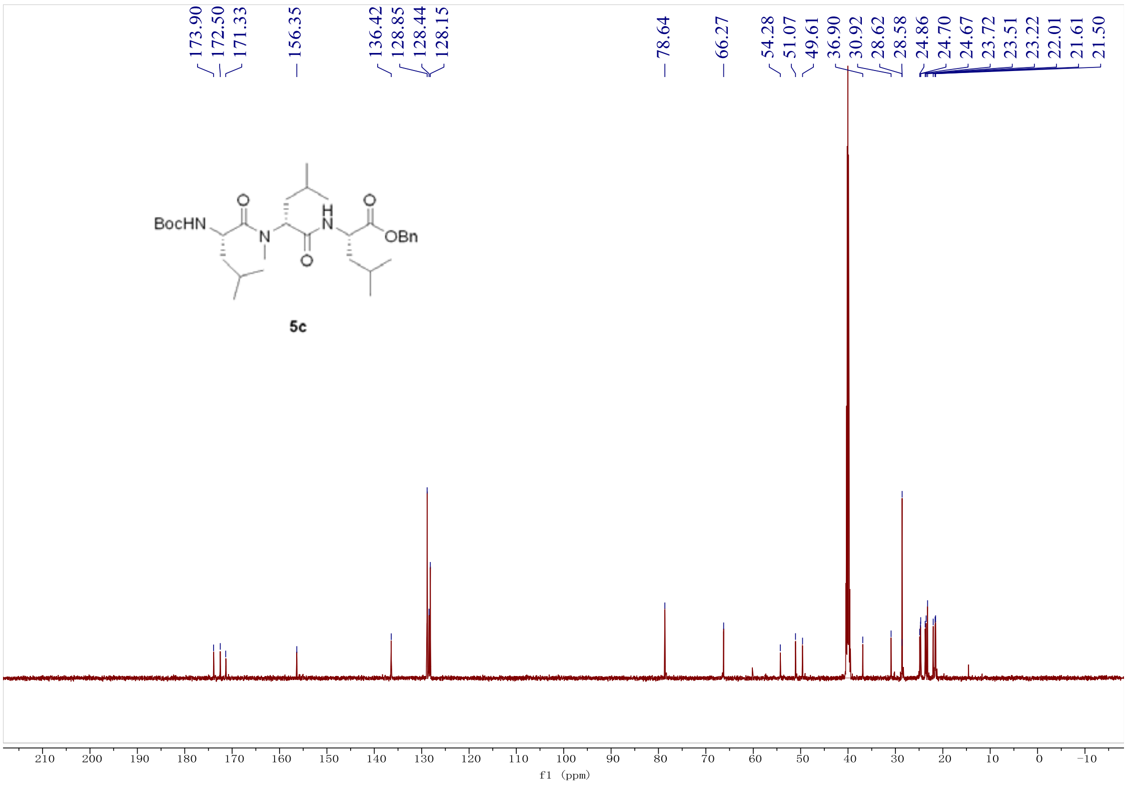

^{1}H NMR (DMSO, 600 MHz) and ^{13}C NMR (DMSO, 151 MHz) for **5d**

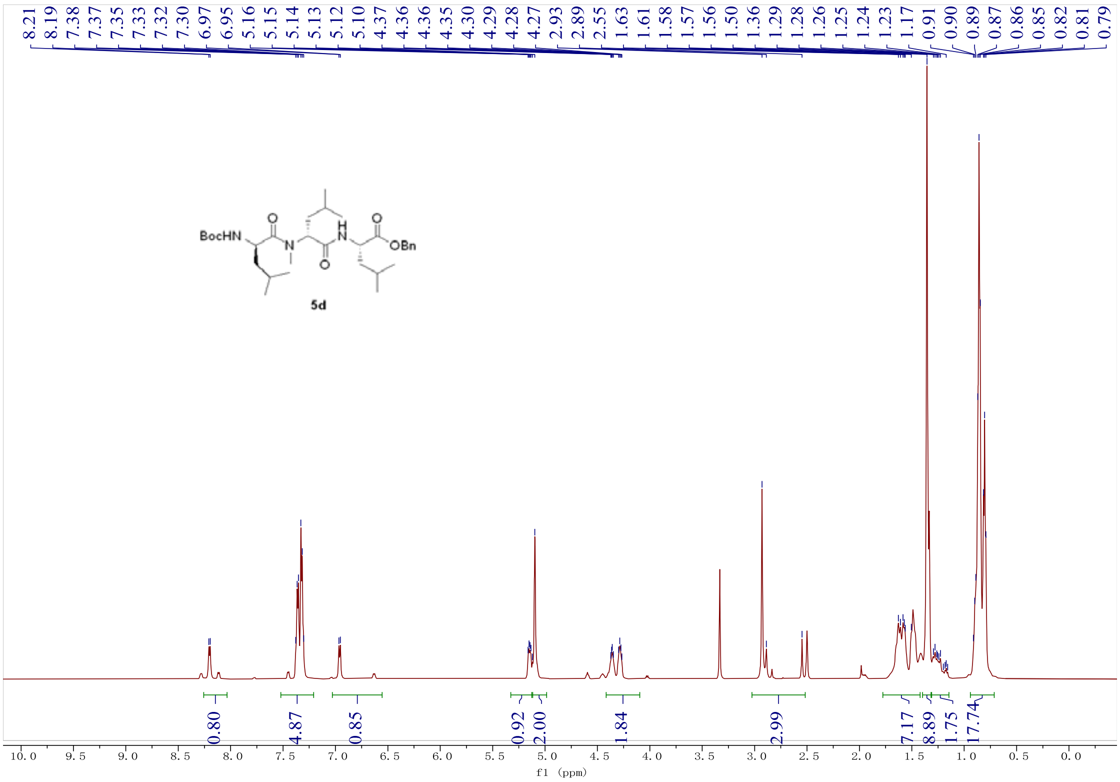

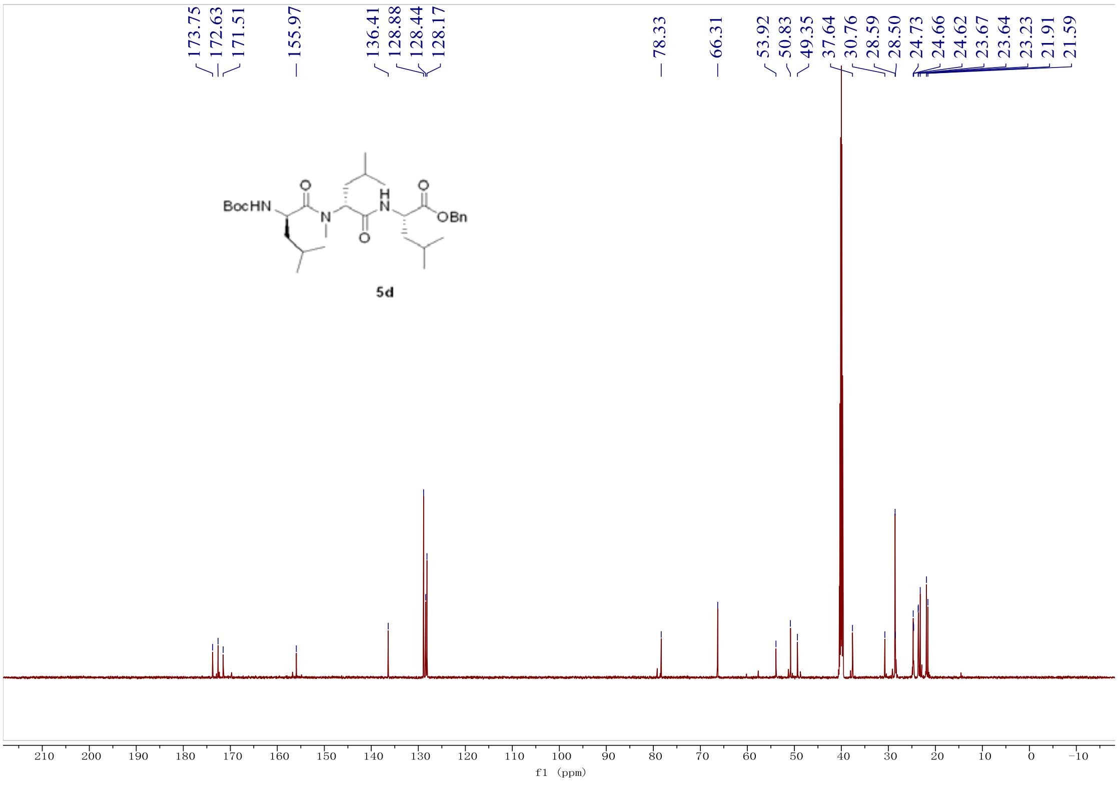

^{1}H NMR (DMSO, 600 MHz) and ^{13}C NMR (DMSO, 151 MHz) for **5e**

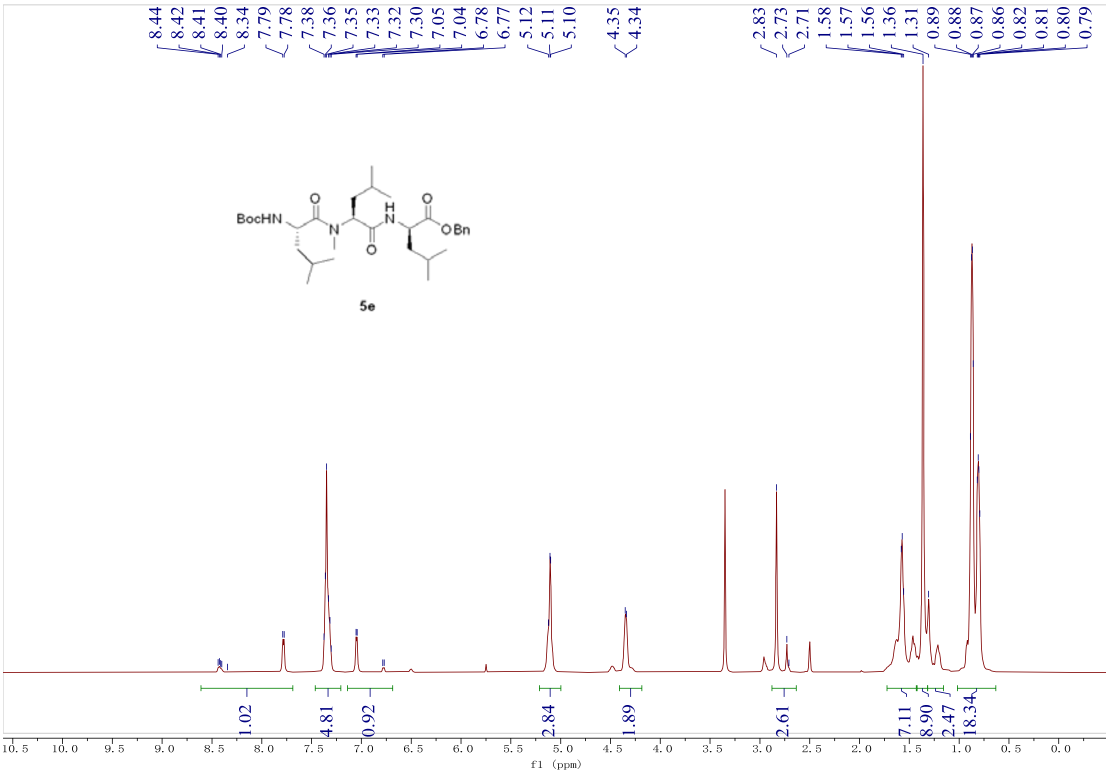

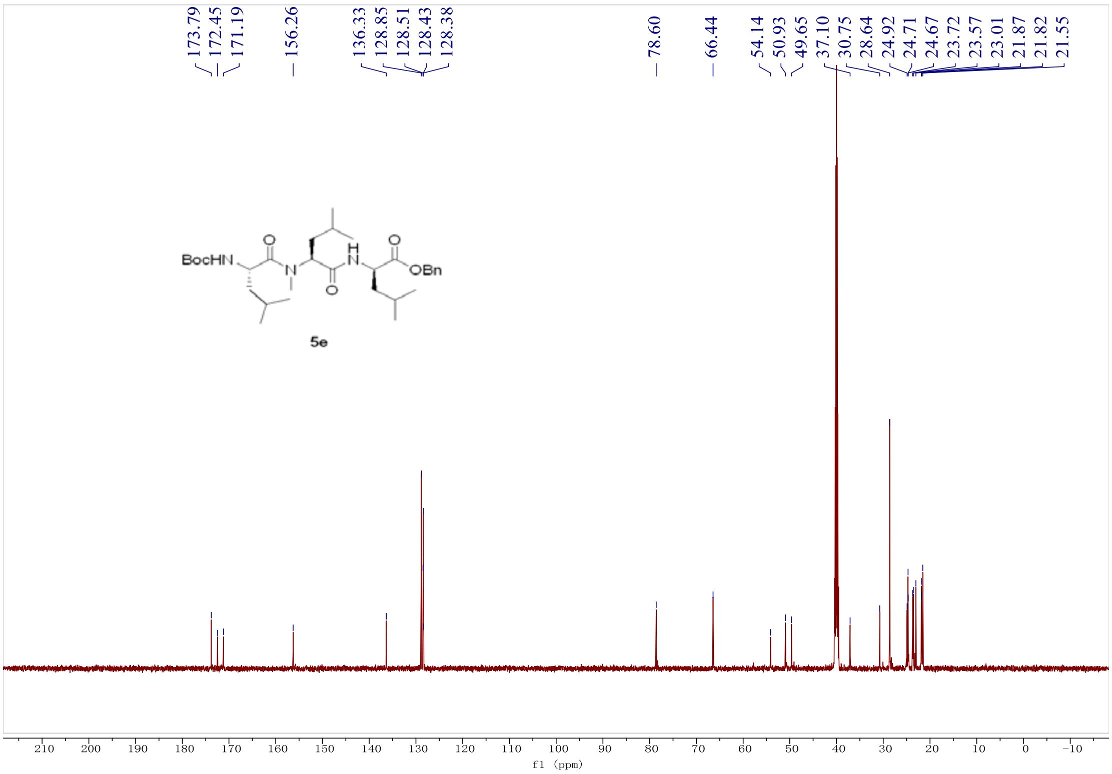

^{1}H NMR (DMSO, 600 MHz) and ^{13}C NMR (DMSO, 151 MHz) for **5f**

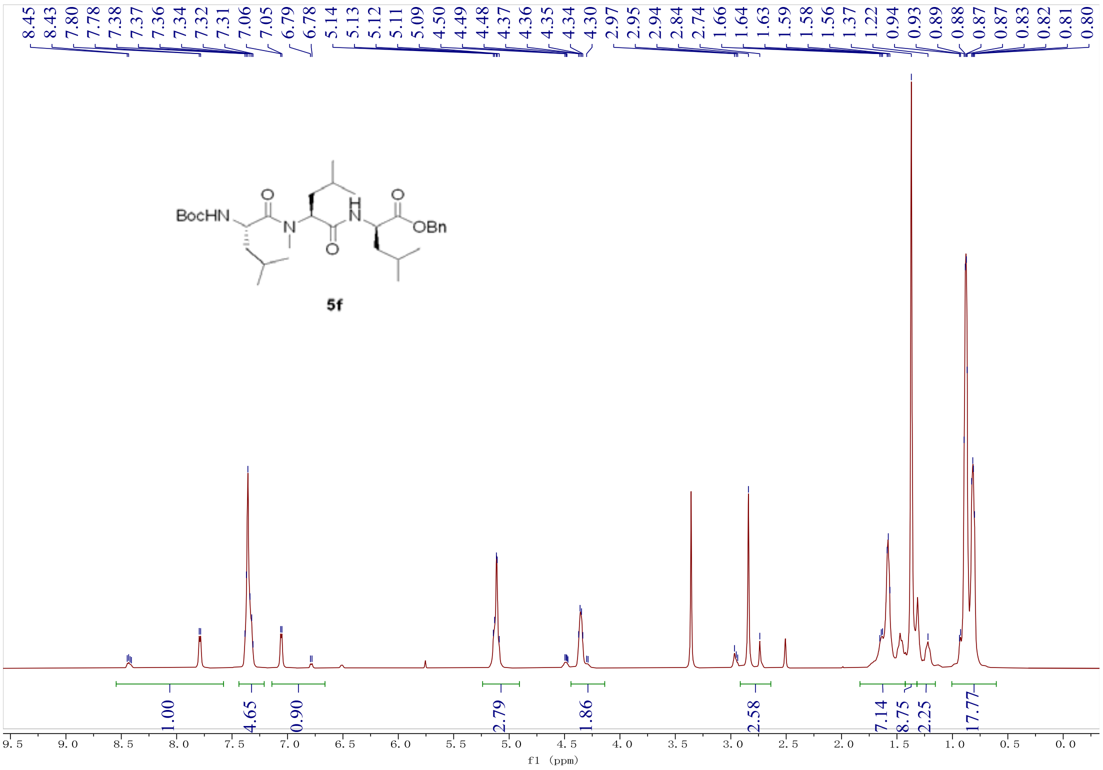

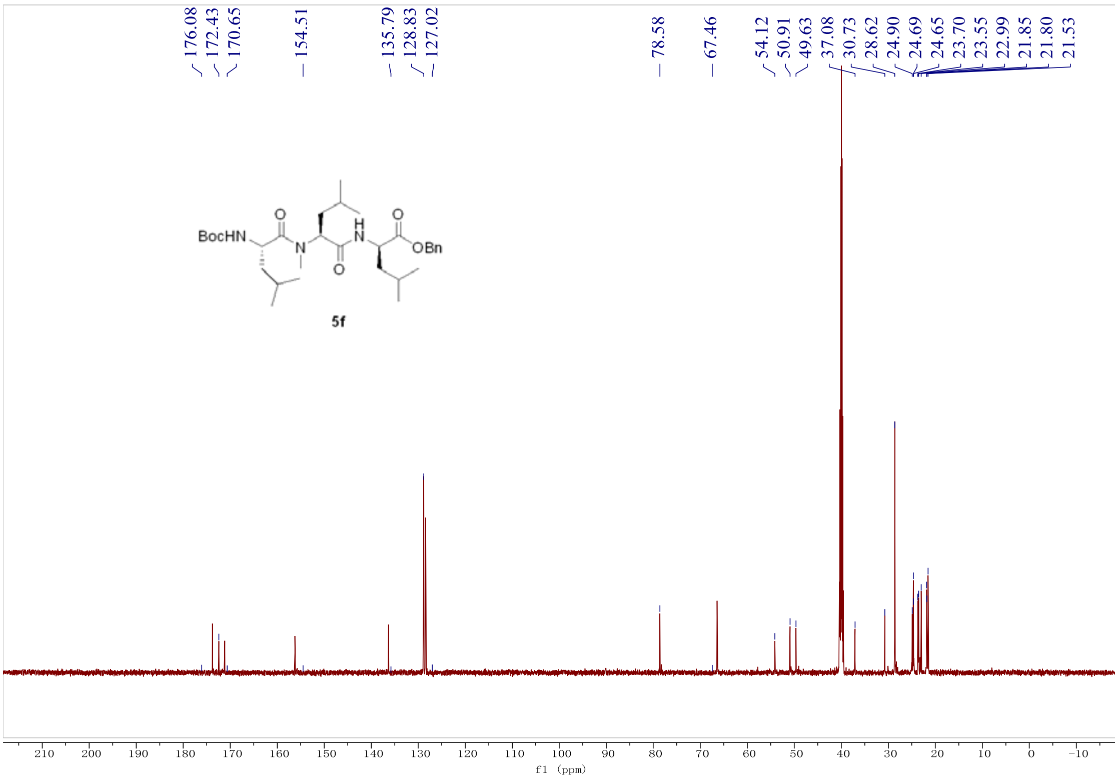

^{1}H NMR ($CDCl_3$, 600 MHz) and ^{13}C NMR ($CDCl_3$, 151 MHz) for **5g**

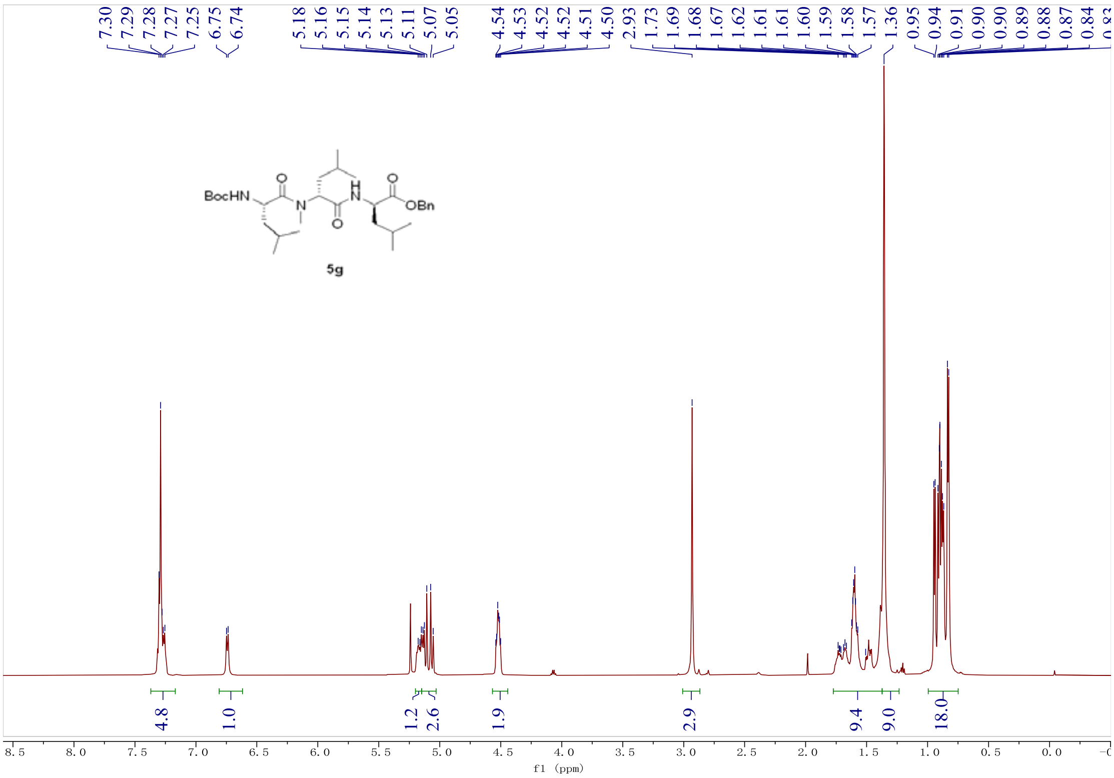

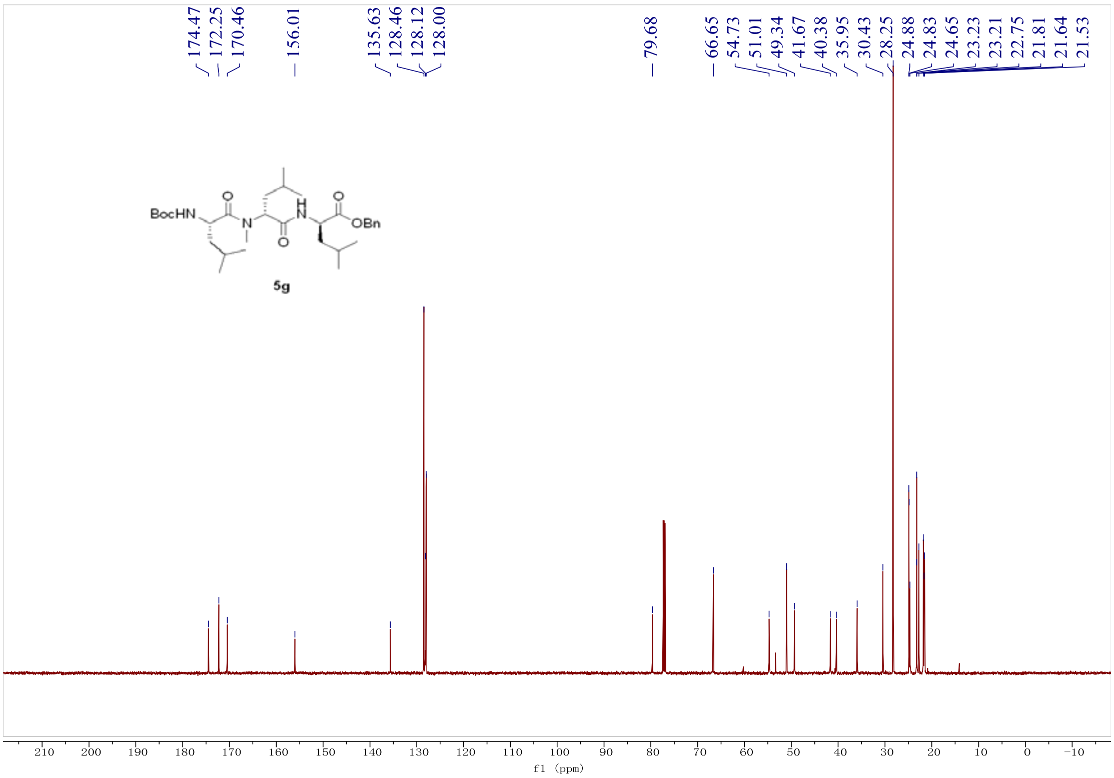

^{1}H NMR ($CDCl_3$, 600 MHz) and ^{13}C NMR ($CDCl_3$, 151 MHz) for **5h**

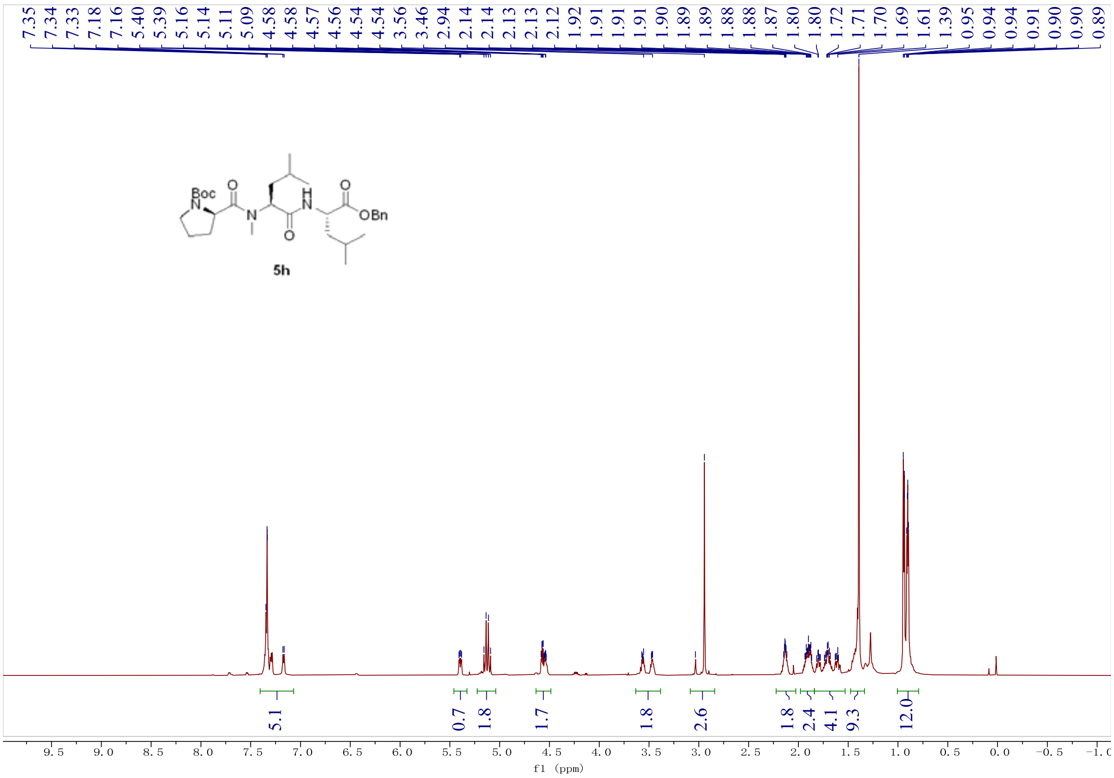

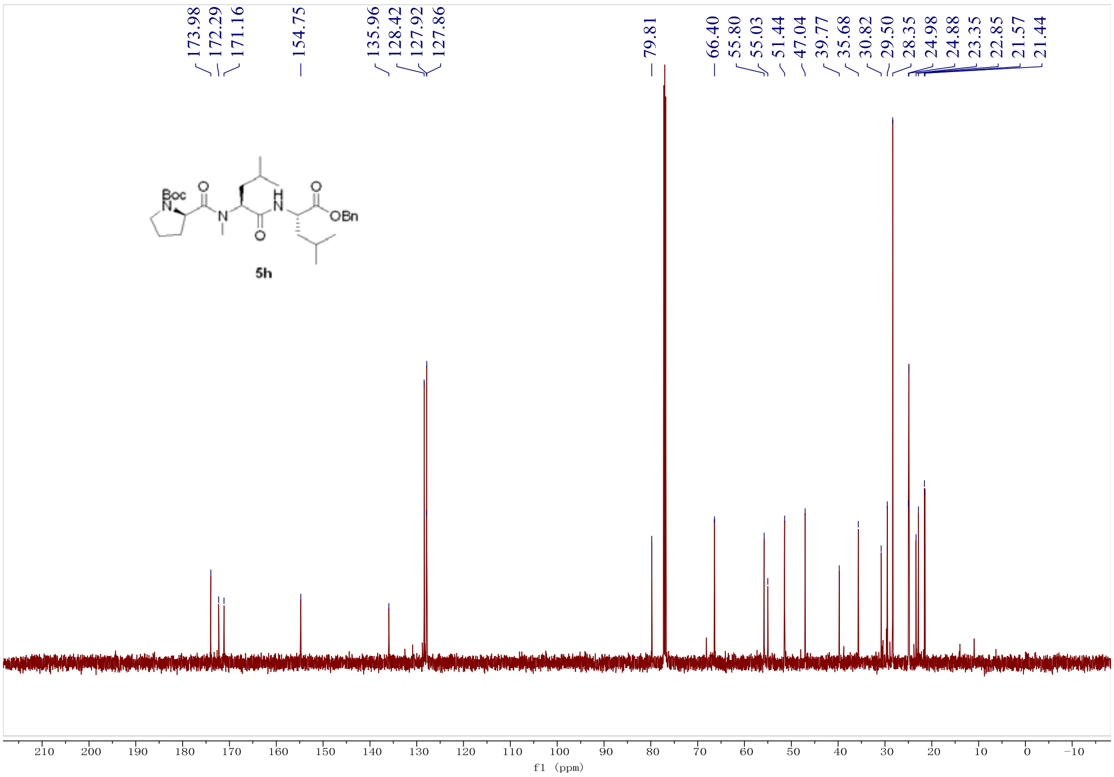

^{1}H NMR ($CDCl_3$, 600 MHz) and ^{13}C NMR ($CDCl_3$, 151 MHz) for **5i**

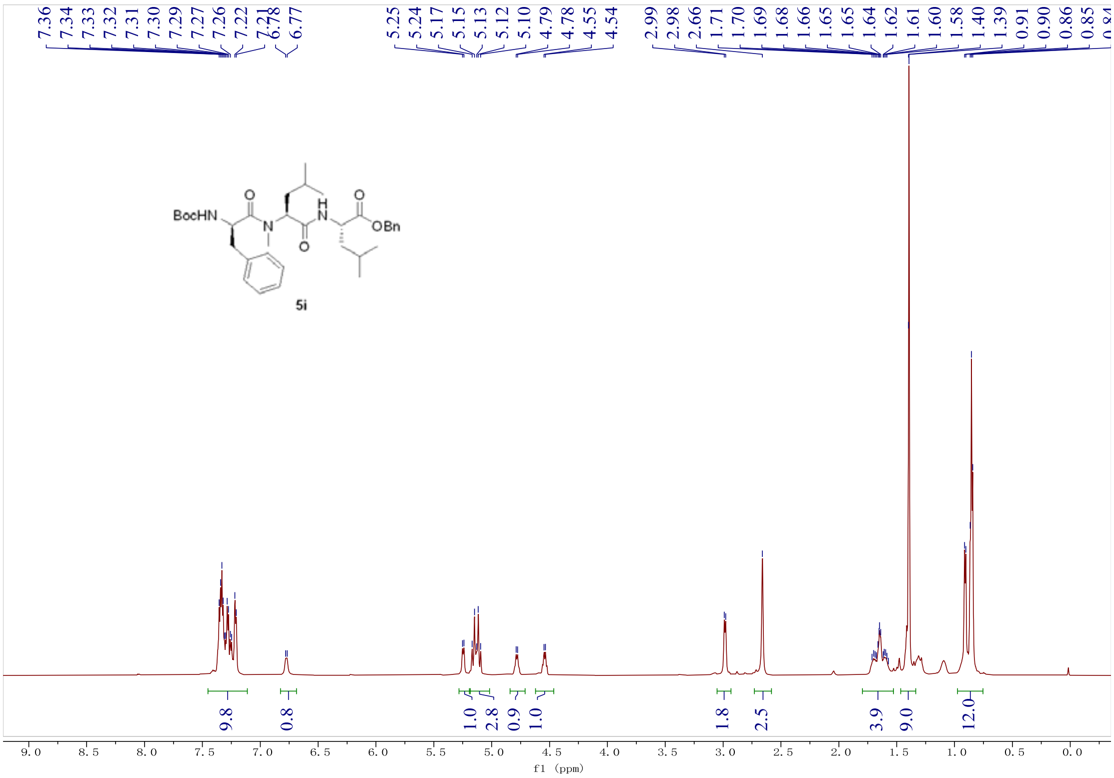

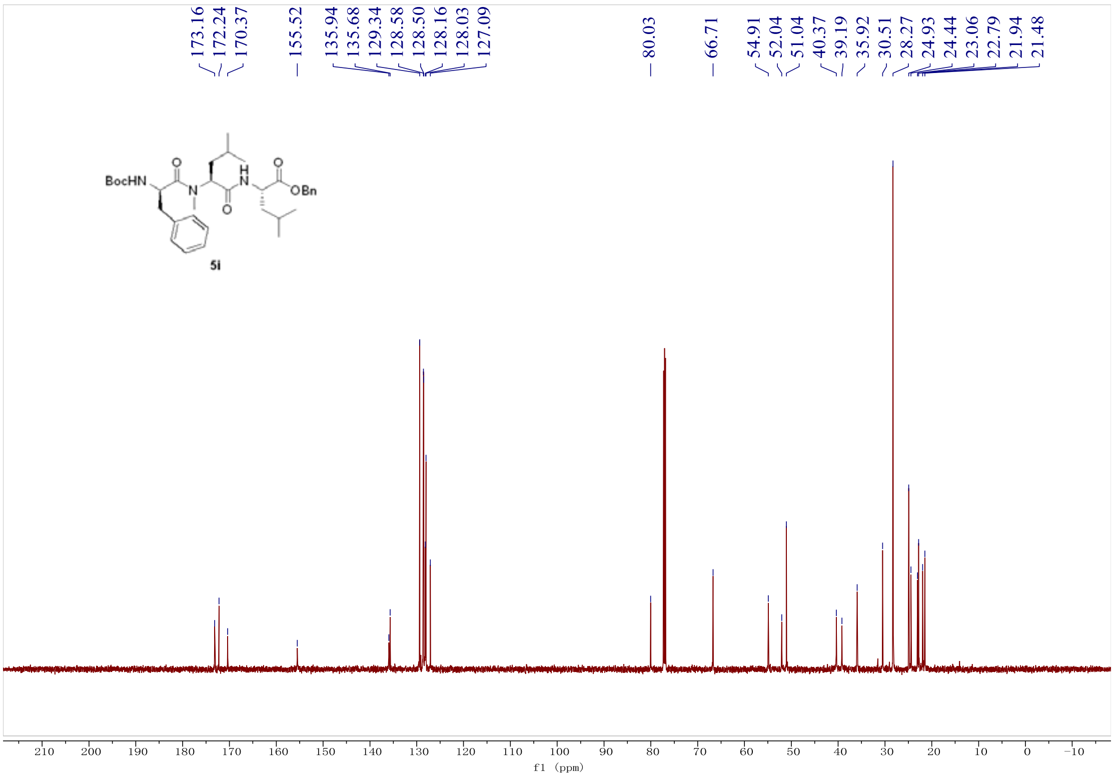

^{1}H NMR (DMSO, 600 MHz) and ^{13}C NMR (DMSO, 151 MHz) for **5j**

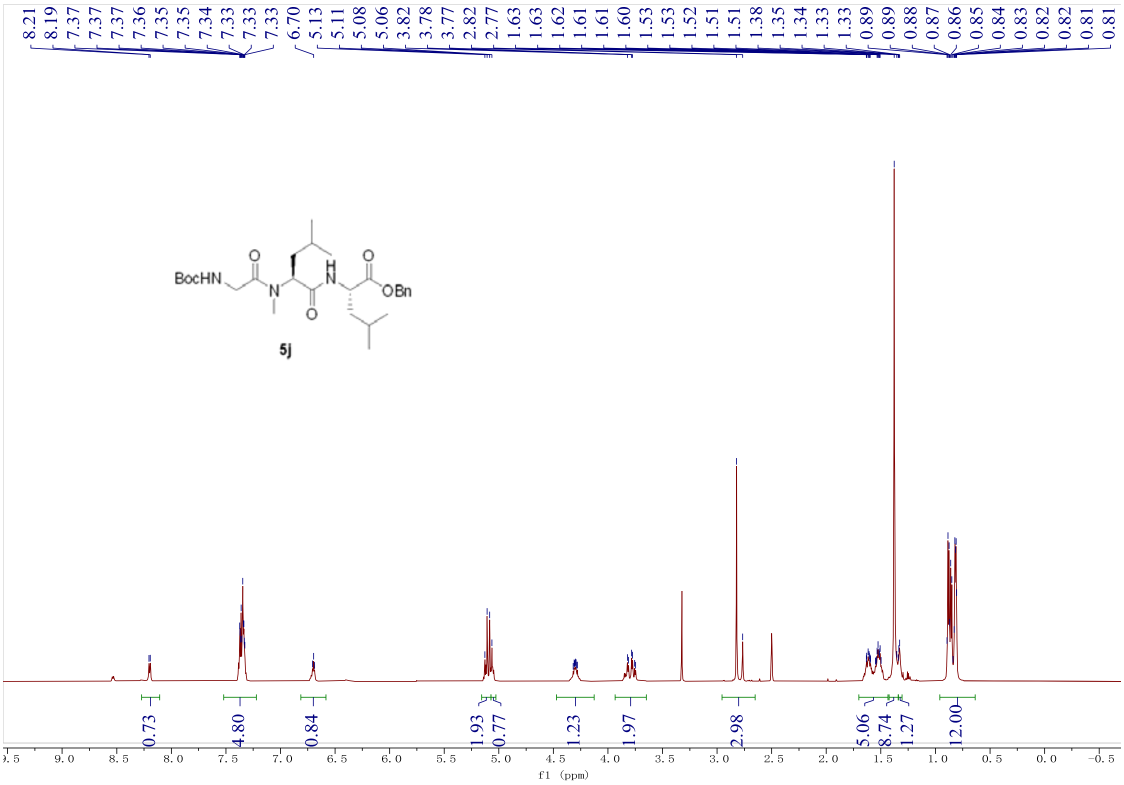

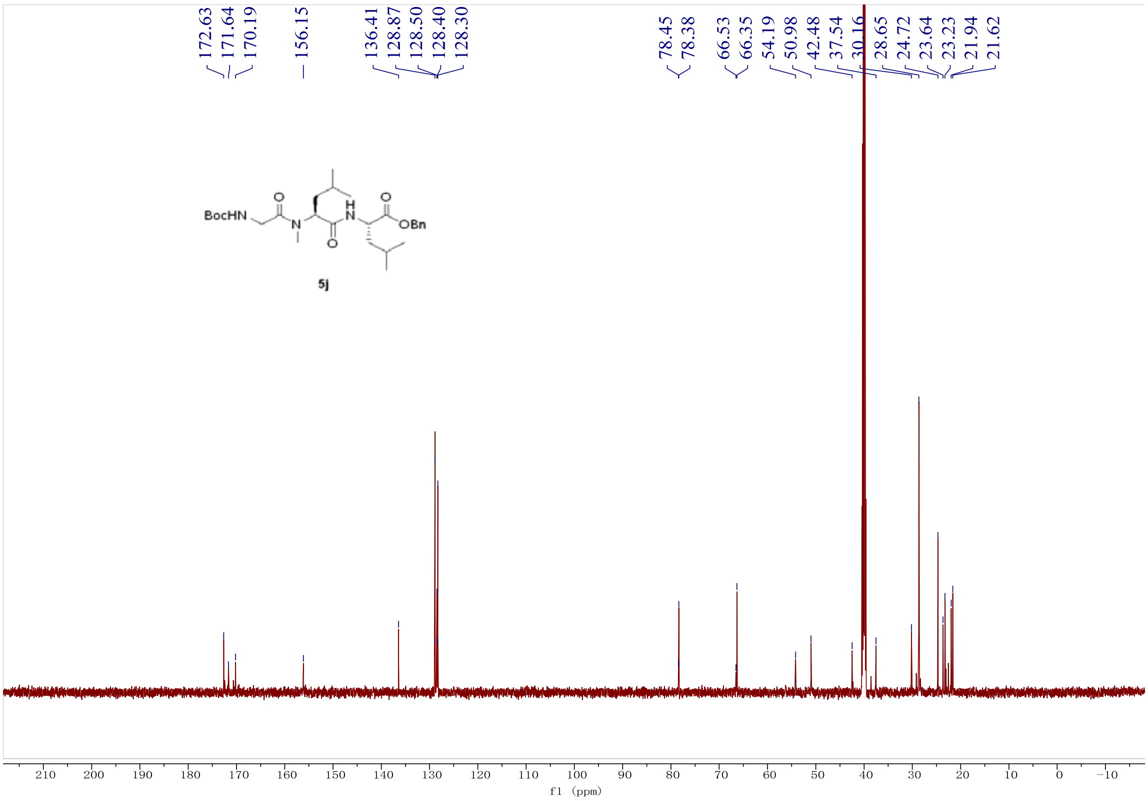

^{1}H NMR ($CDCl_3$, 600 MHz) and ^{13}C NMR ($CDCl_3$, 151 MHz) for **5k**

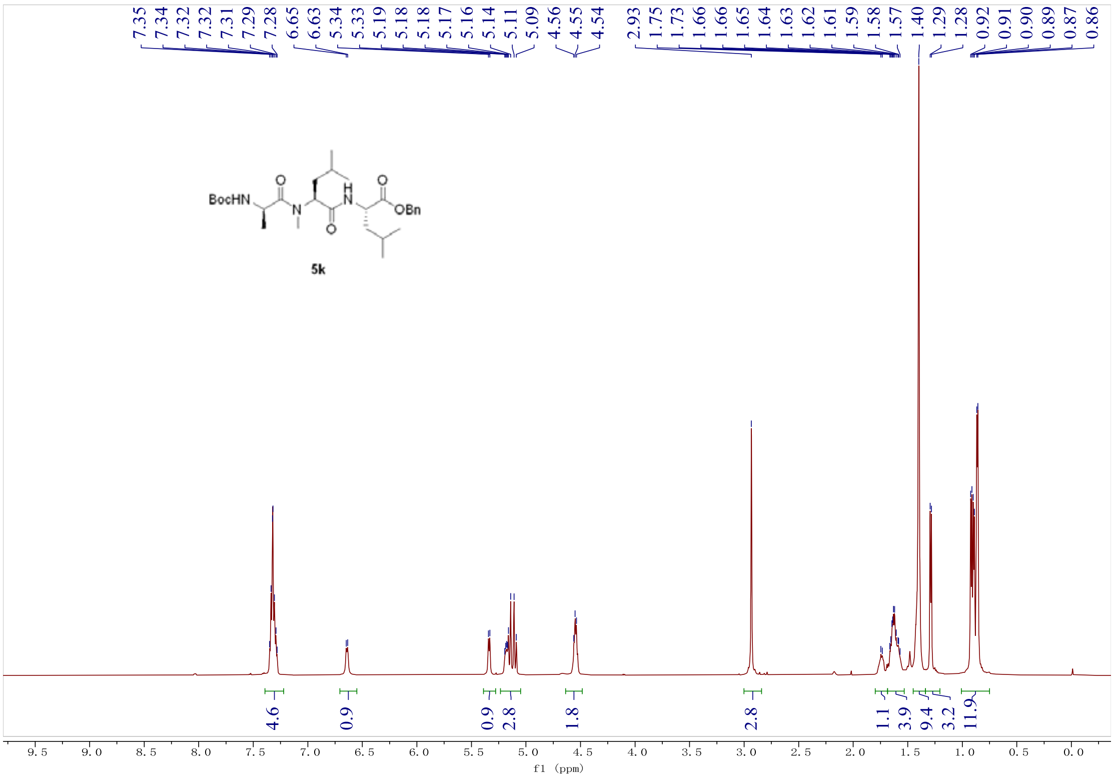

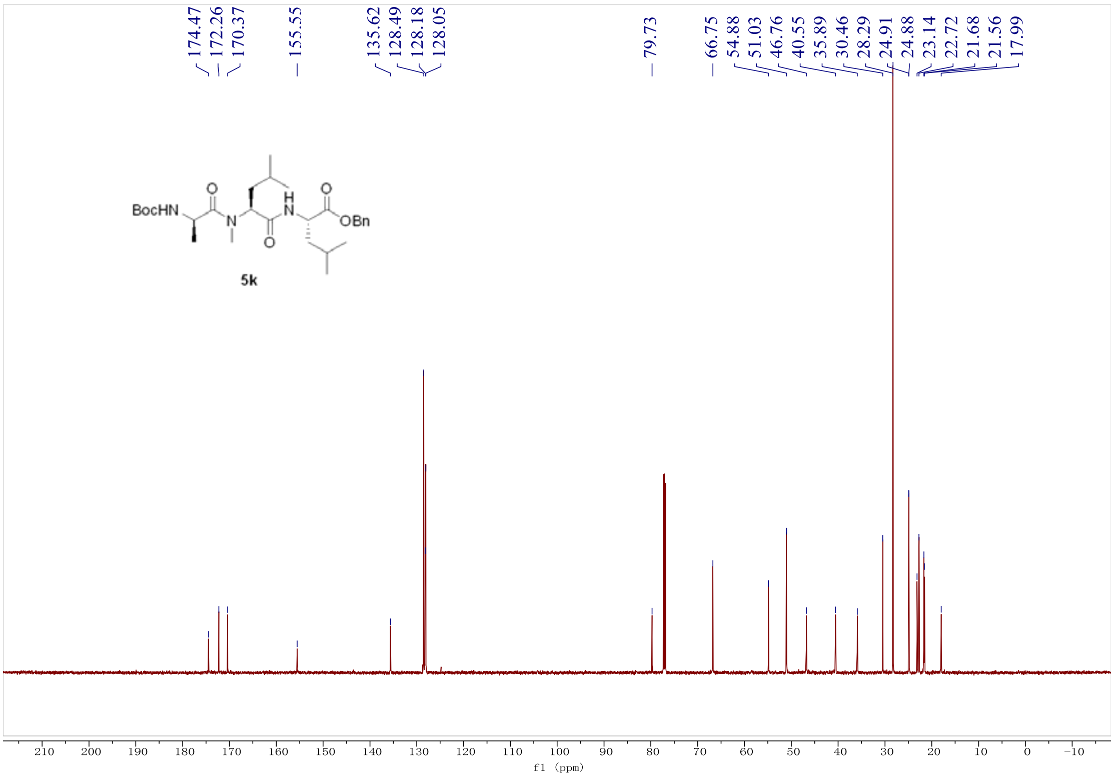

^{1}H NMR (DMSO, 600 MHz) and ^{13}C NMR (DMSO, 151 MHz) for **5l**

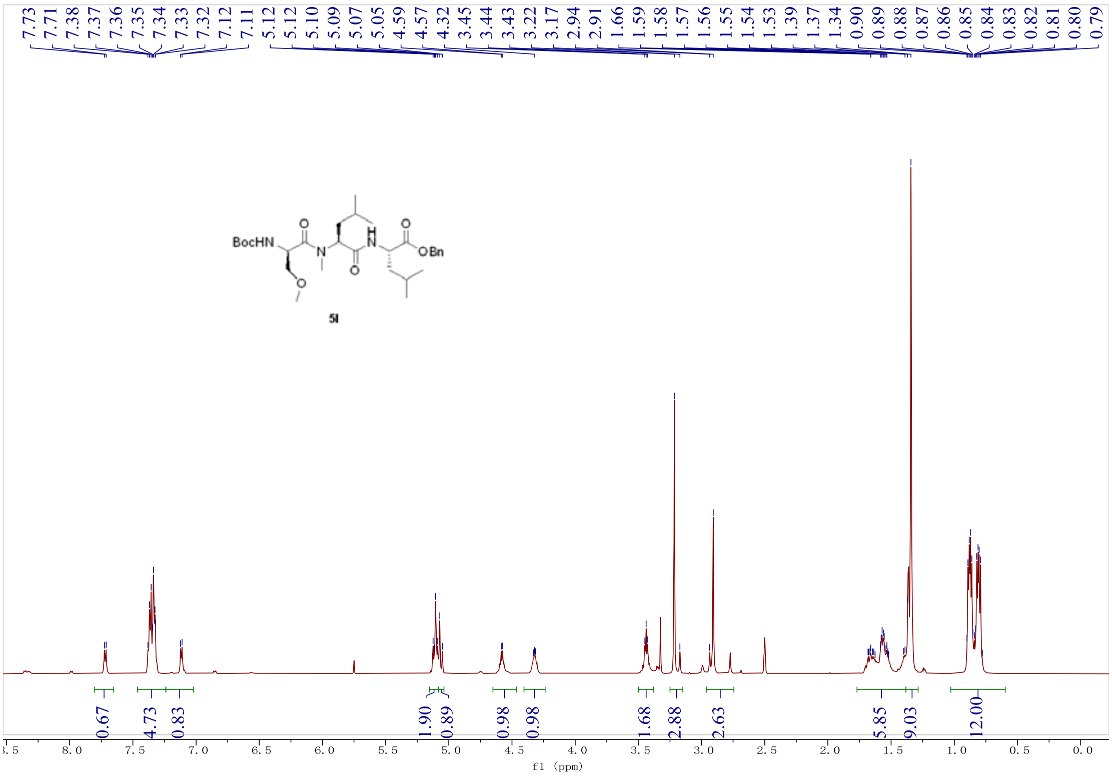

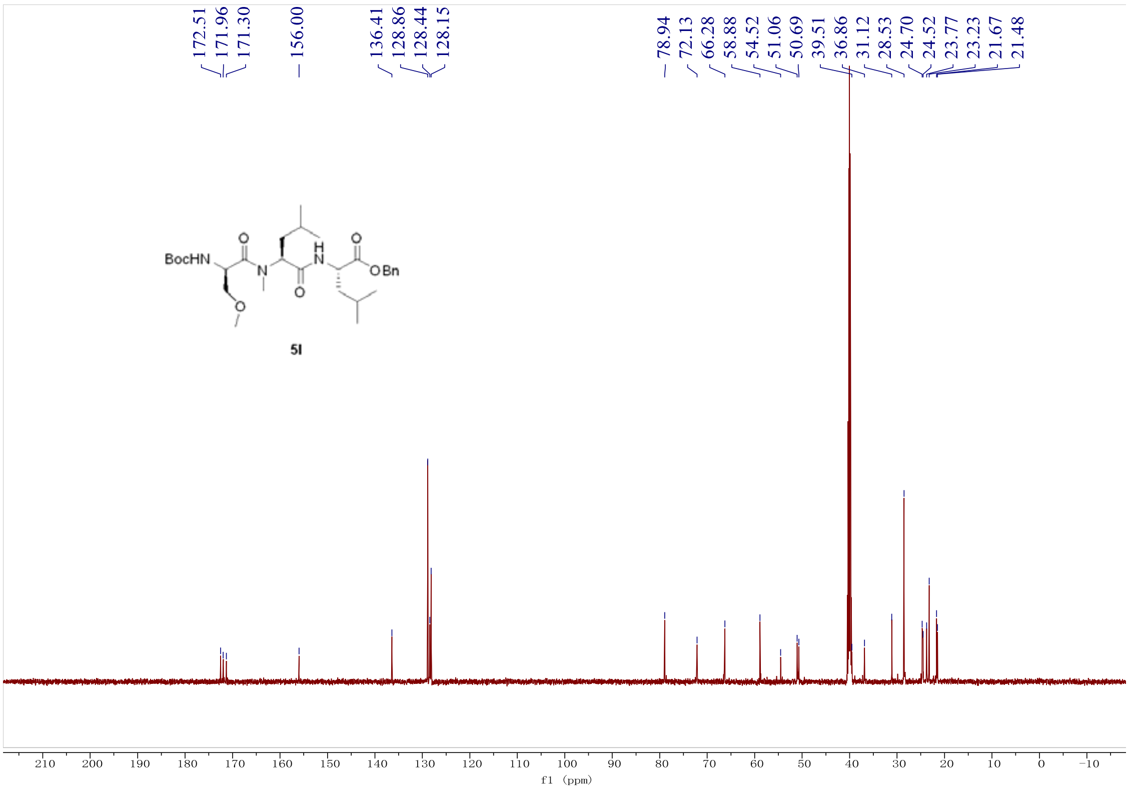

^{1}H NMR ($CDCl_3$, 600 MHz) and ^{13}C NMR ($CDCl_3$, 151 MHz) for **5m**

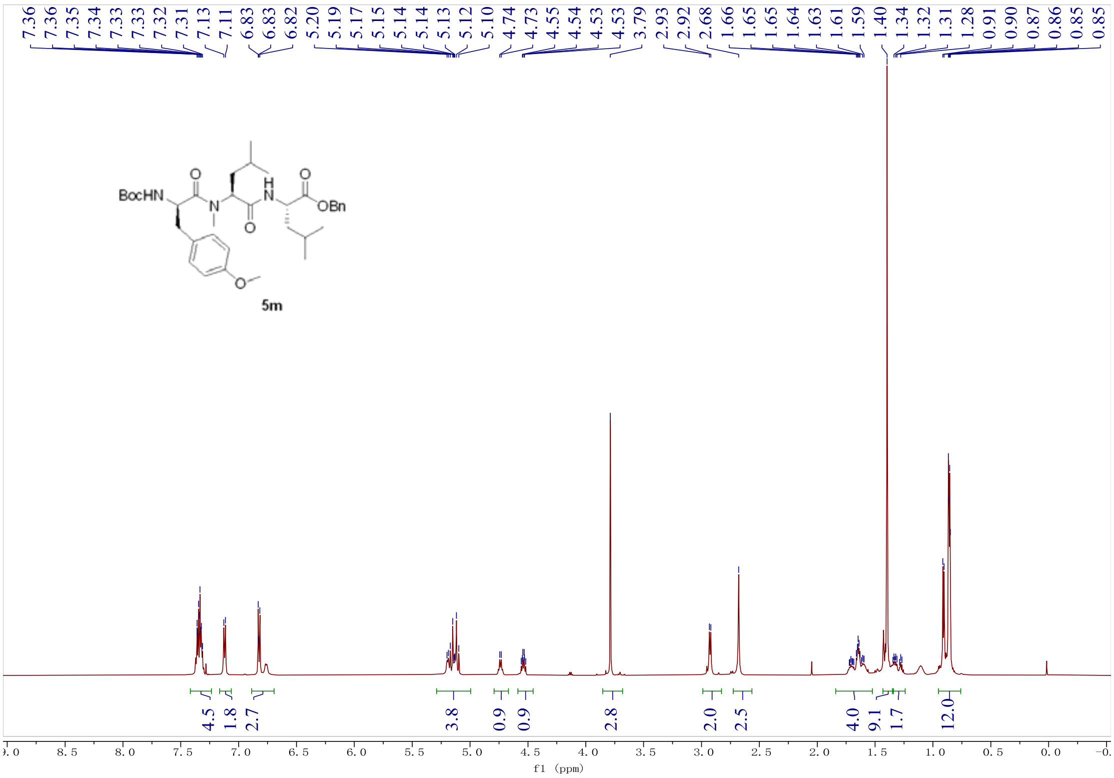

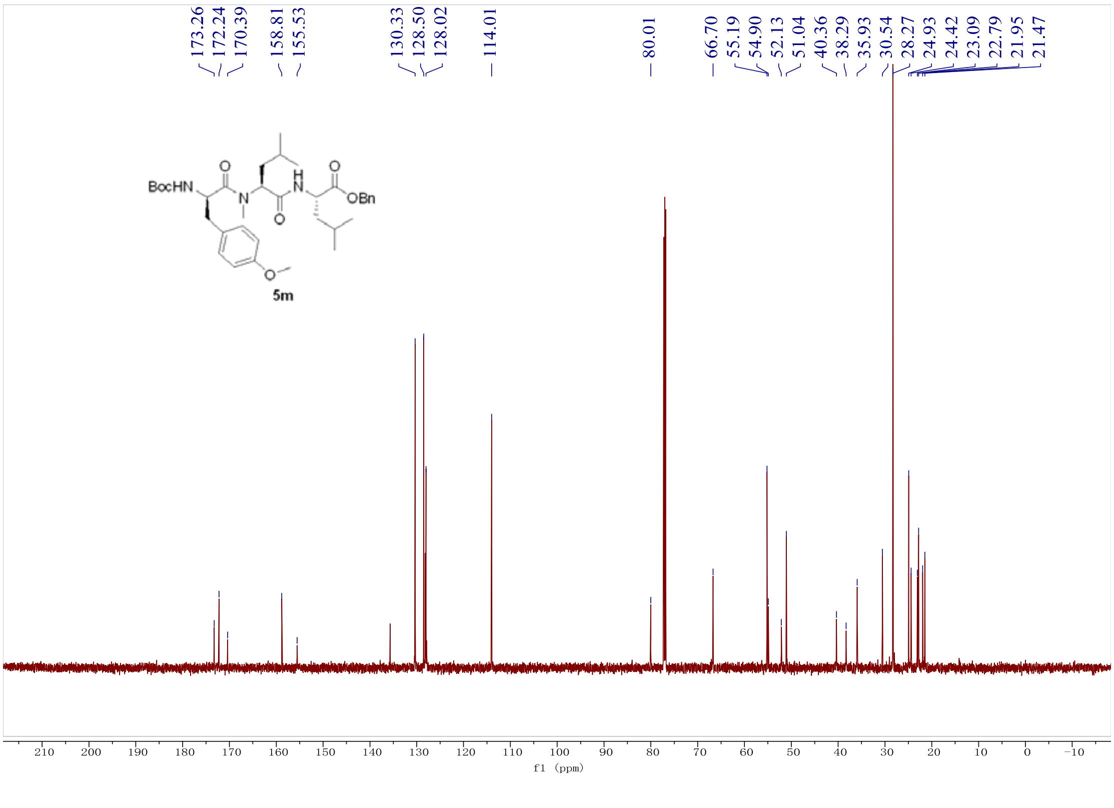

^{1}H NMR (DMSO, 600 MHz) and ^{13}C NMR (DMSO, 151 MHz) for **5n**

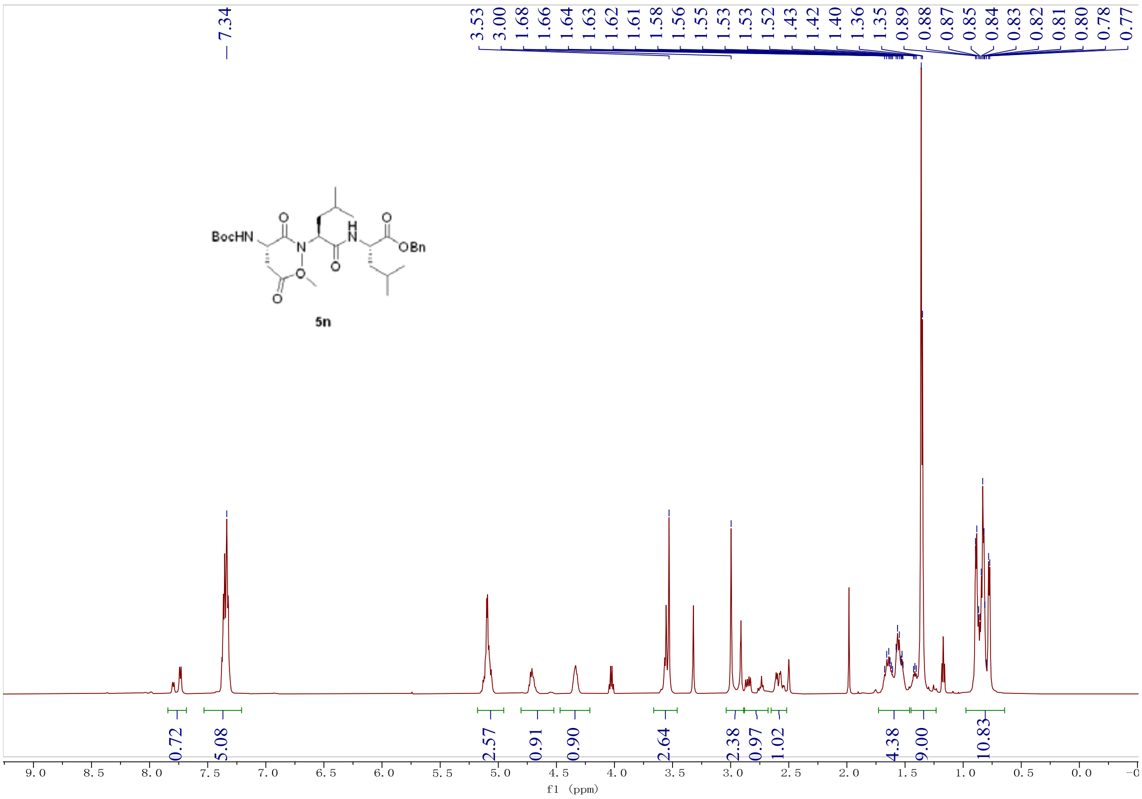

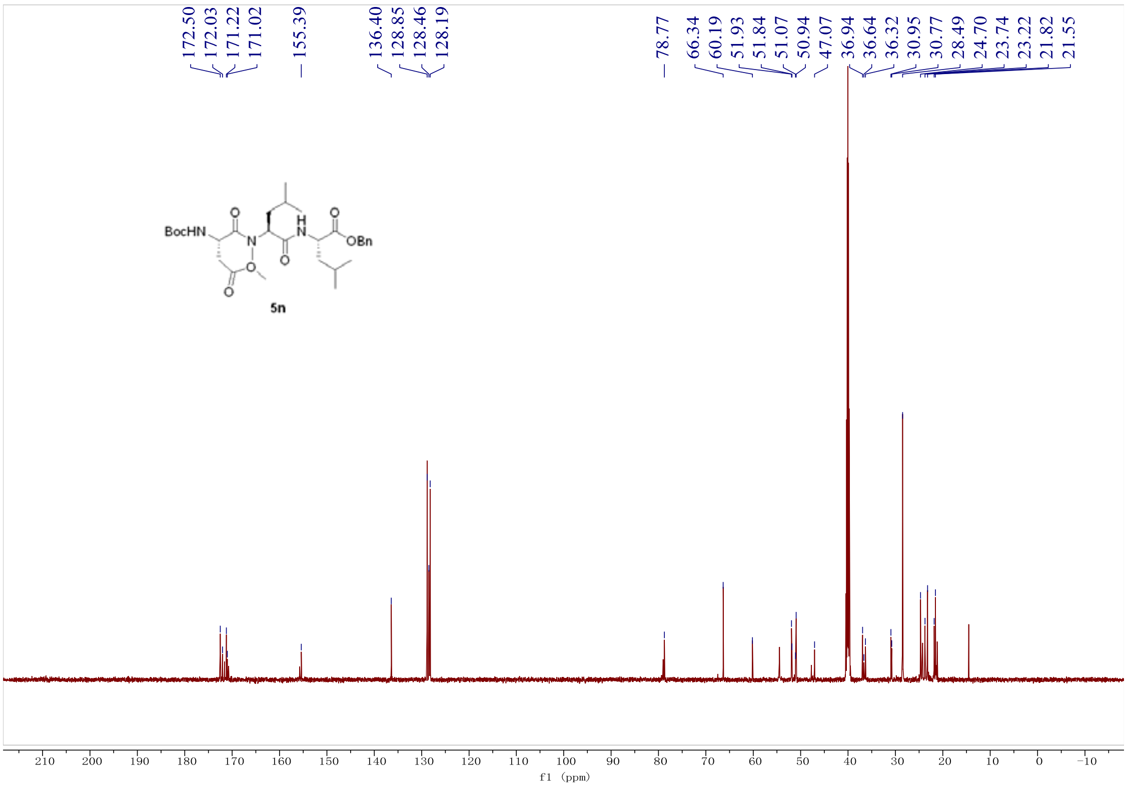

^{1}H NMR (DMSO, 600 MHz) and ^{13}C NMR (DMSO, 151 MHz) for **5o**

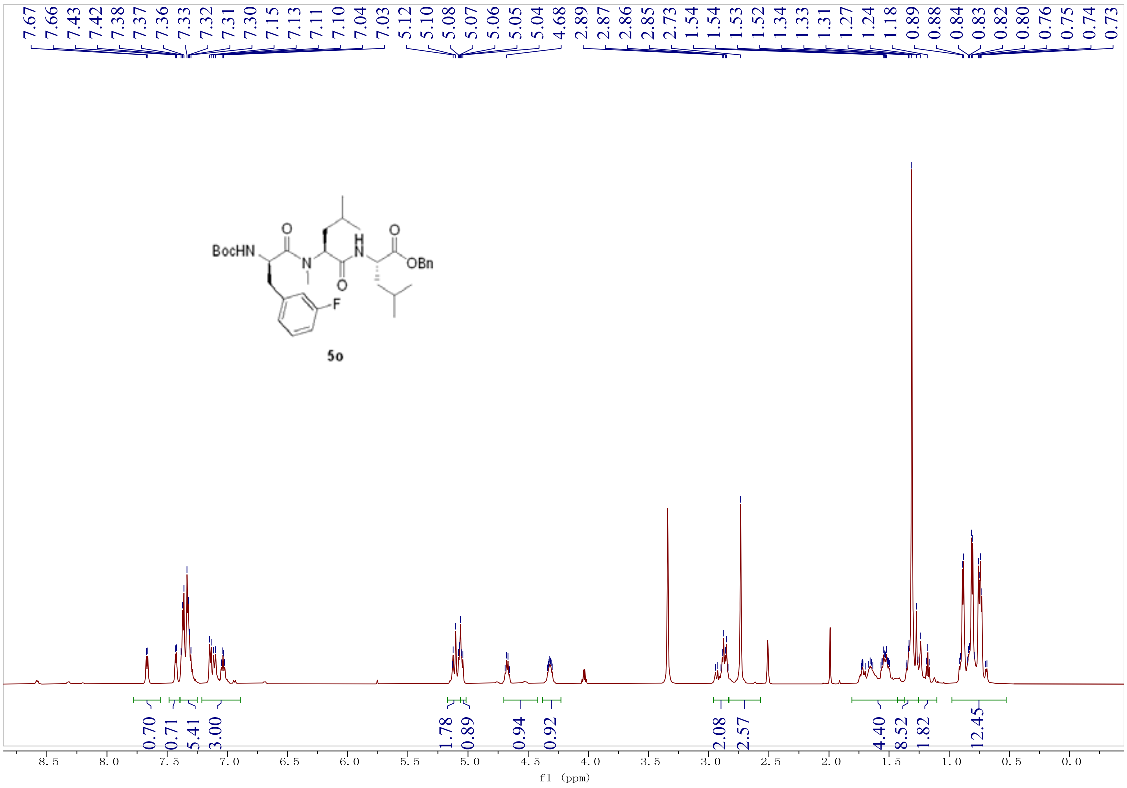

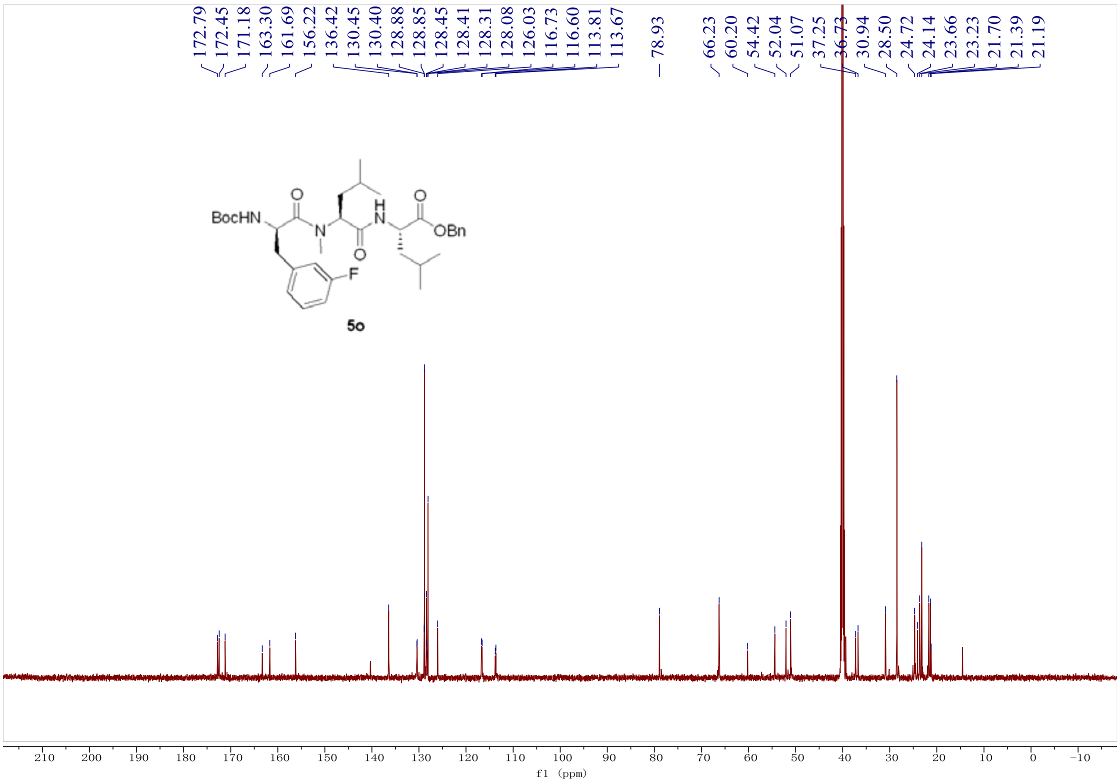

^{1}H NMR ($CDCl_3$, 600 MHz) and ^{13}C NMR ($CDCl_3$, 151 MHz) for **5p**

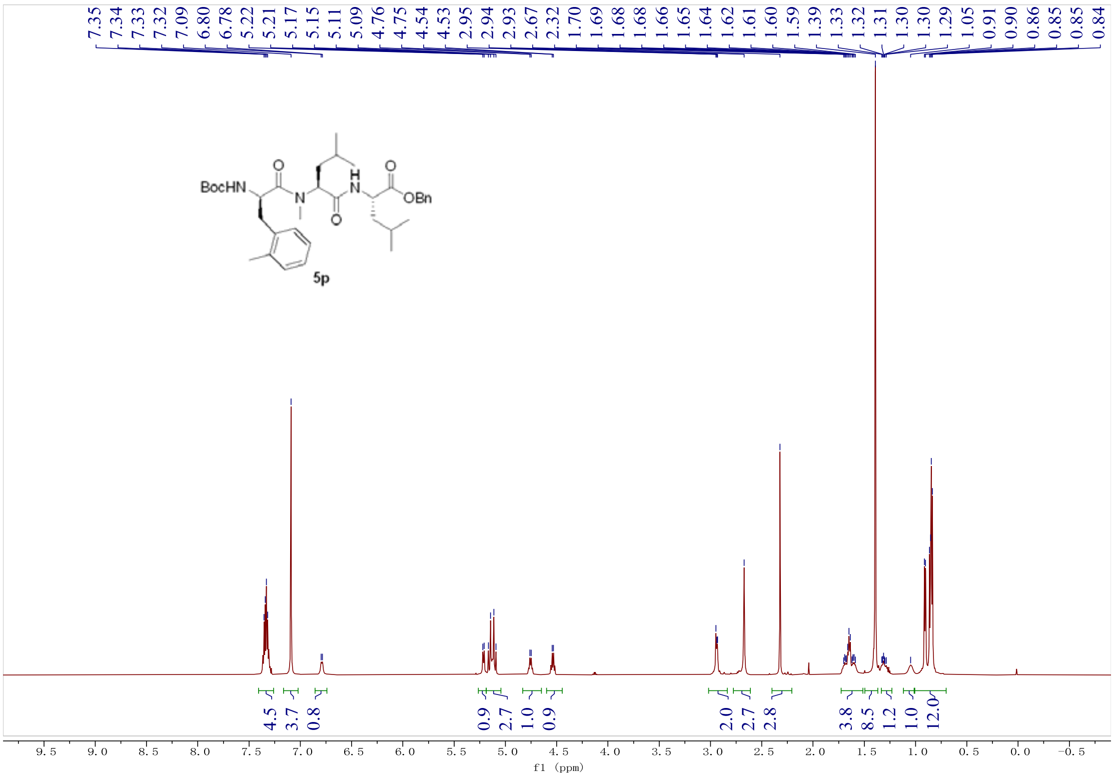

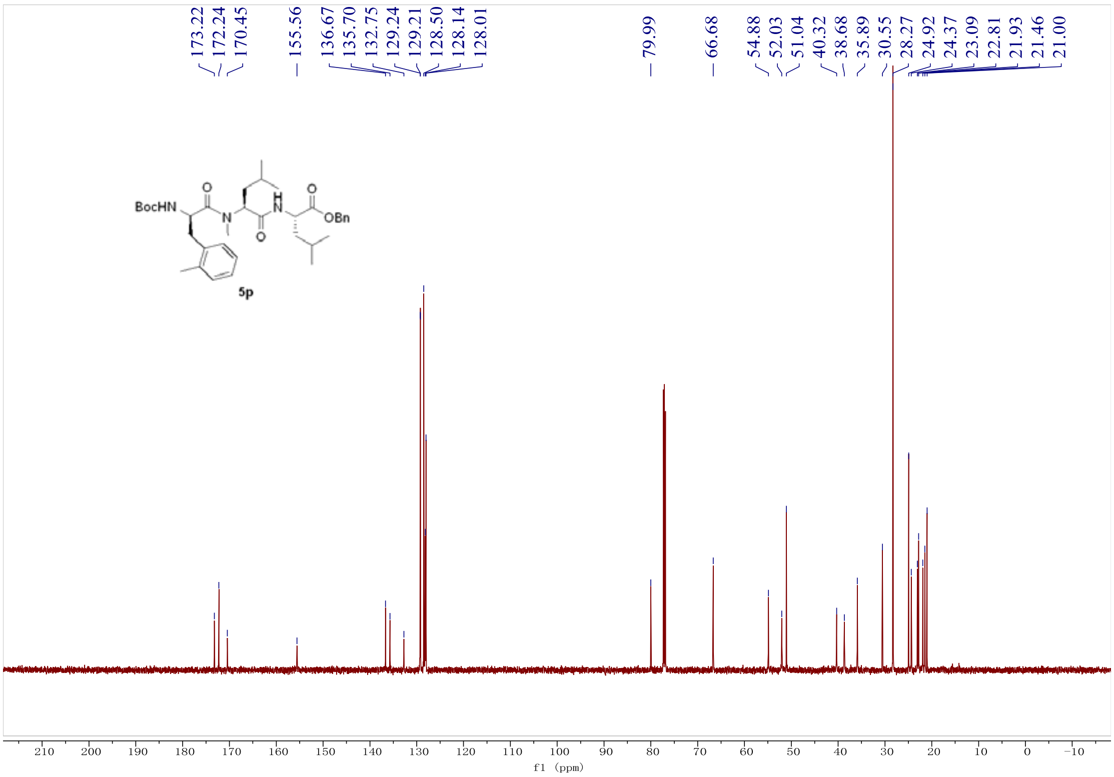

^{1}H NMR (DMSO, 600 MHz) and ^{13}C NMR (DMSO, 151 MHz) for **5q**

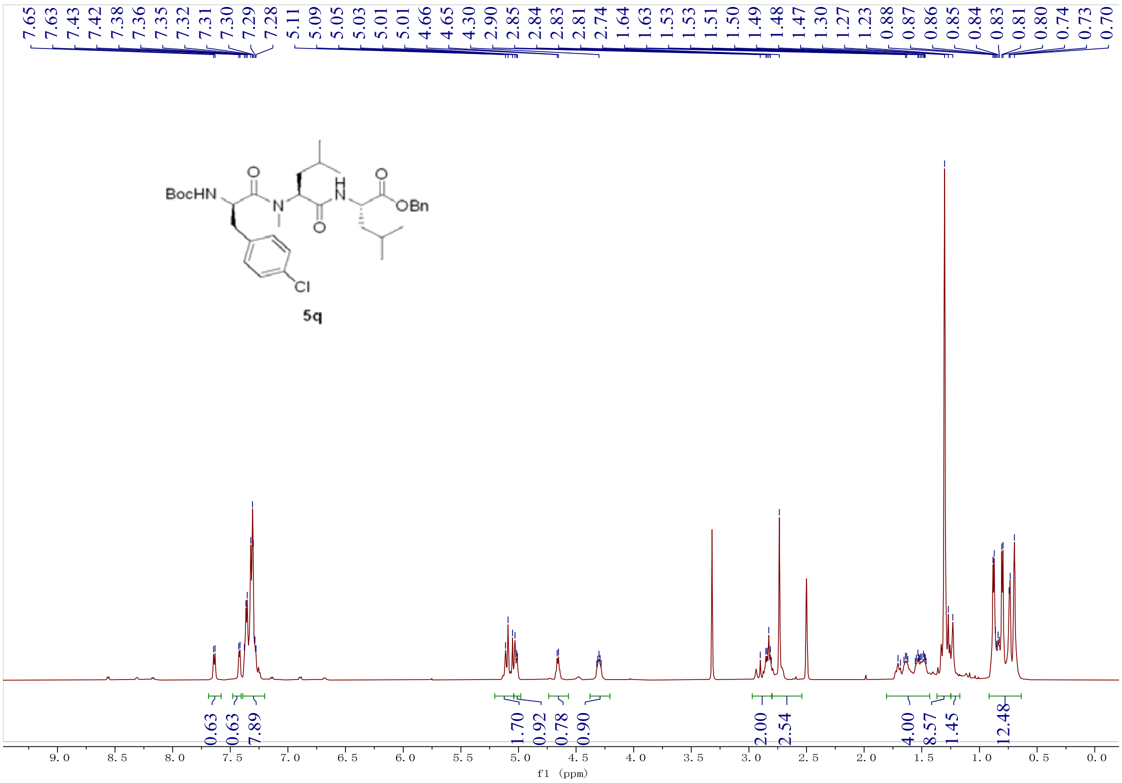

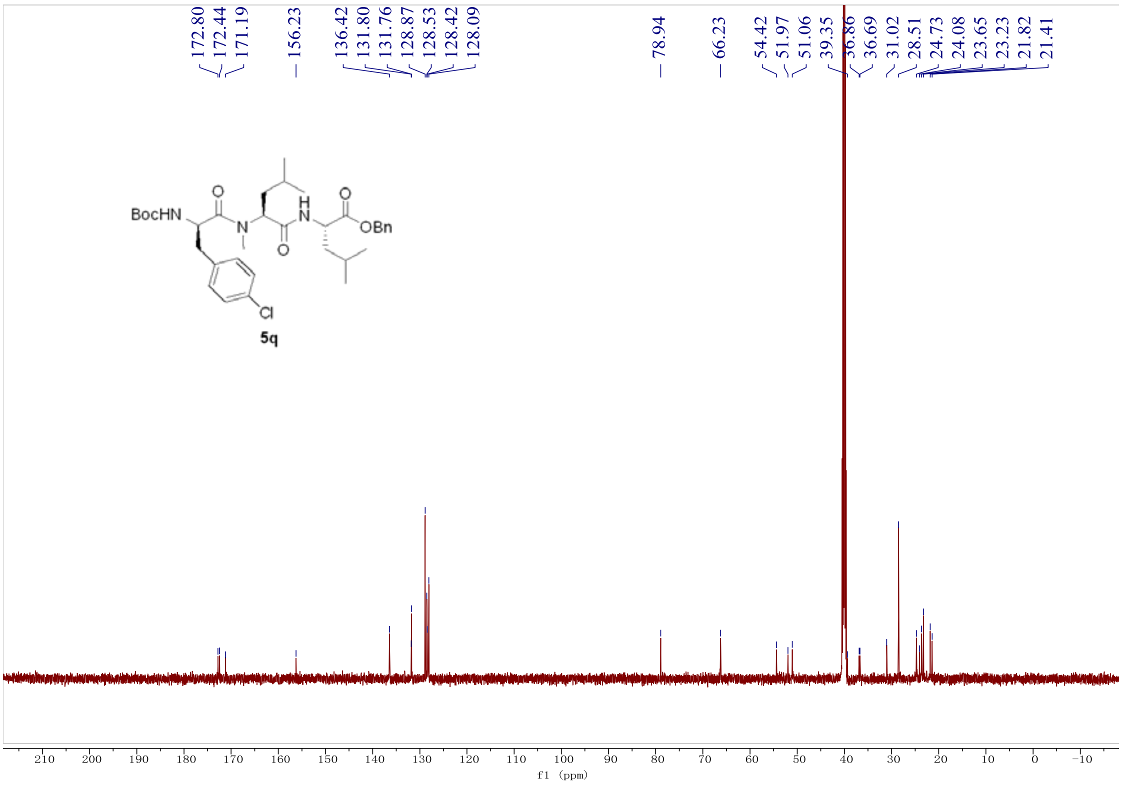

^{1}H NMR ($CDCl_3$, 600 MHz) and ^{13}C NMR ($CDCl_3$, 151 MHz) for **5r**

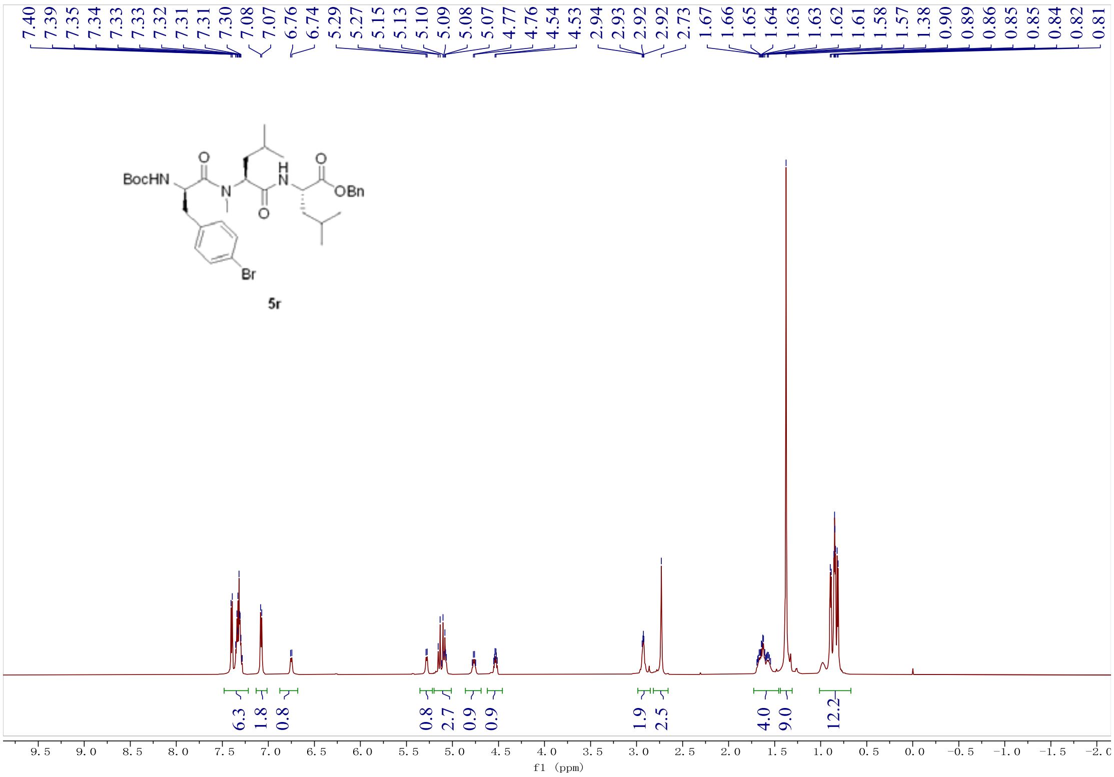

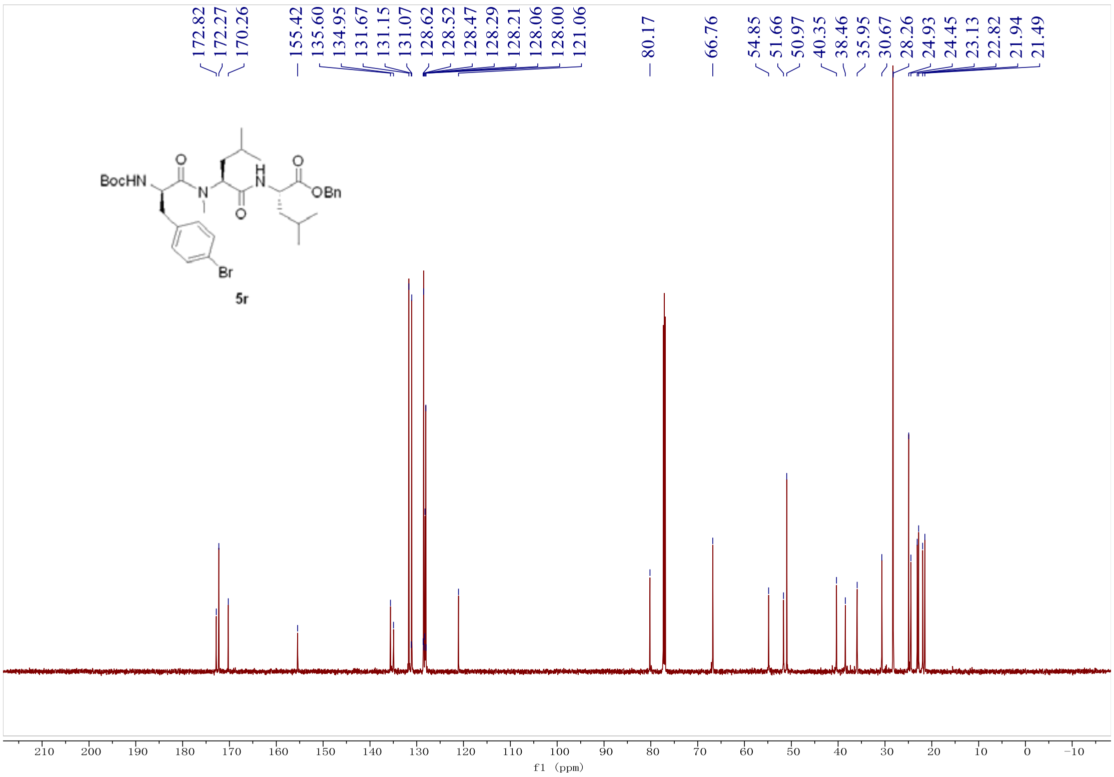

^{1}H NMR (DMSO, 600 MHz) and ^{13}C NMR (DMSO, 151 MHz) for **5s**

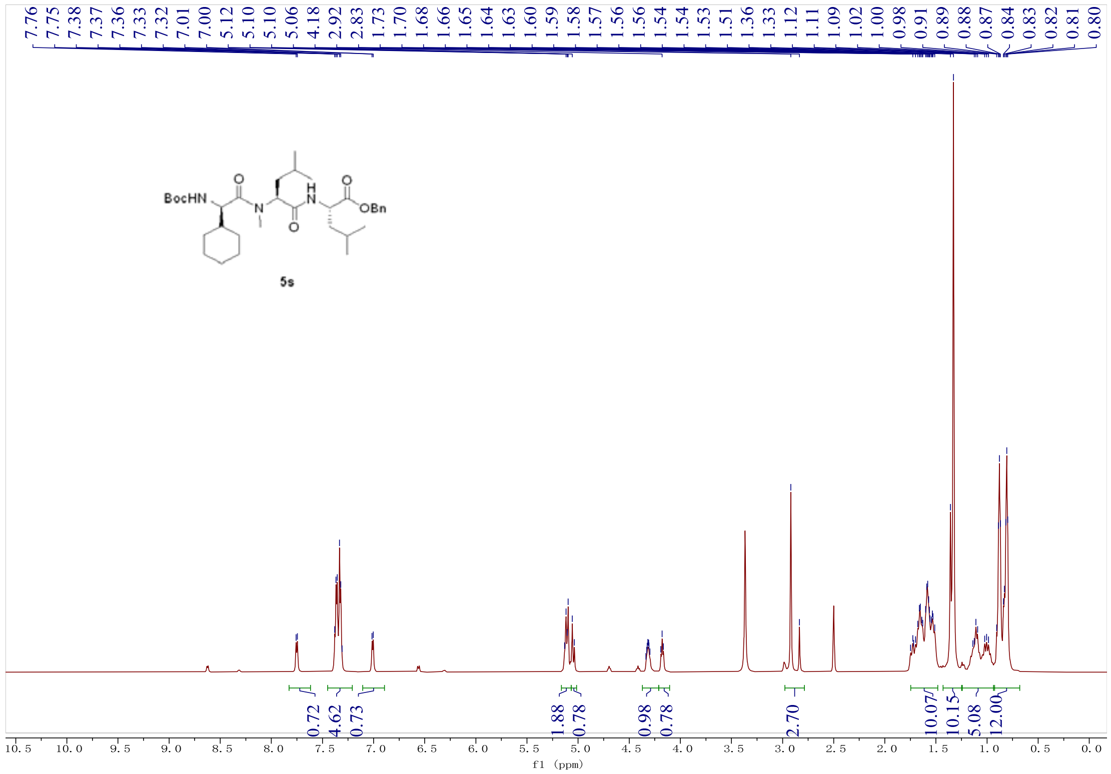

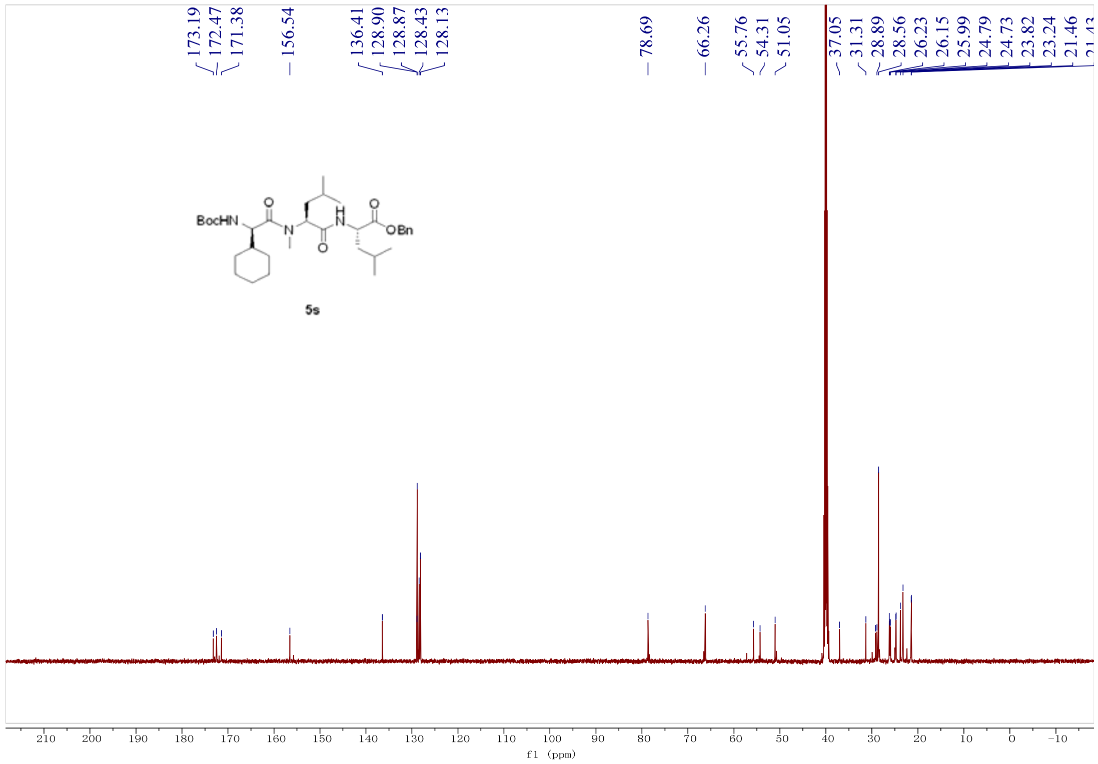

^{1}H NMR (DMSO, 600 MHz) and ^{13}C NMR (DMSO, 151 MHz) for **5t**

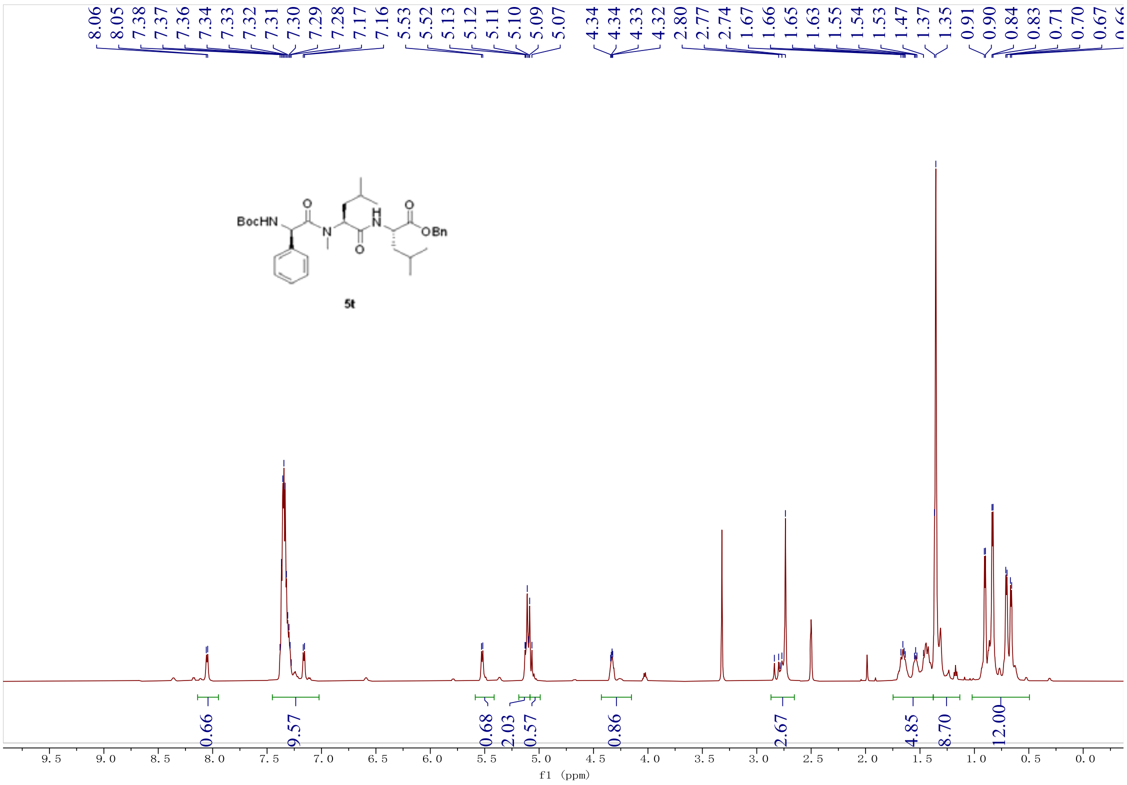

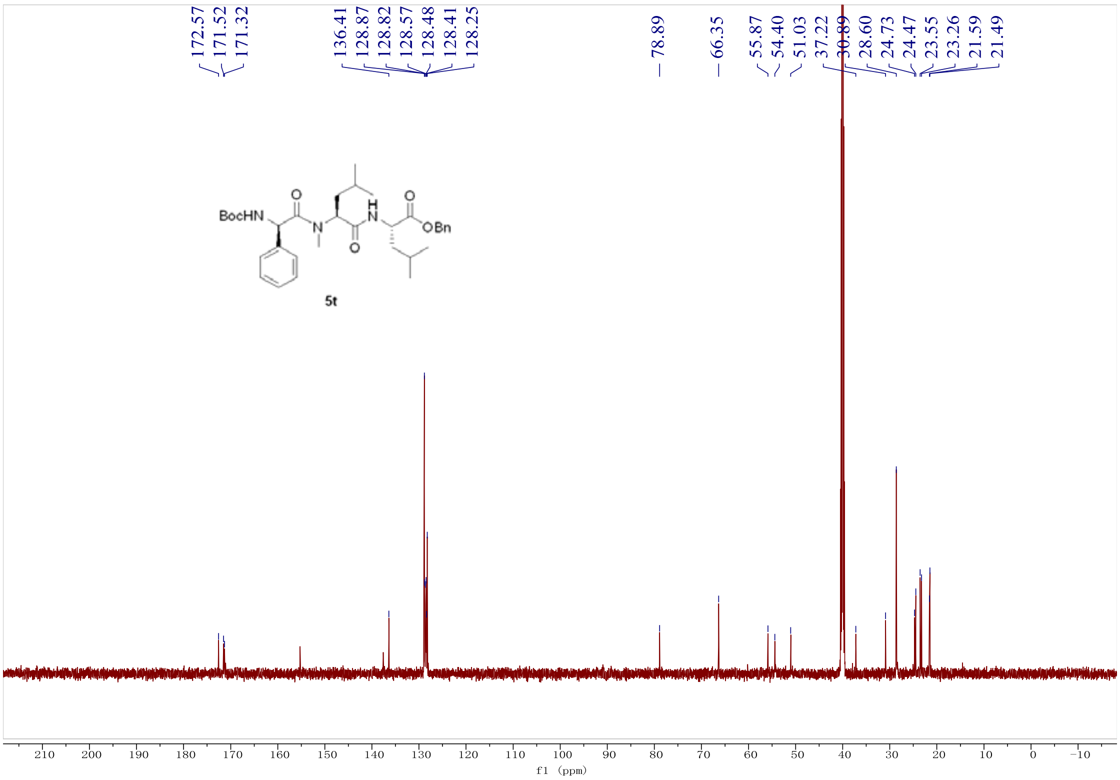

^{1}H NMR (DMSO, 600 MHz) and ^{13}C NMR (DMSO, 151 MHz) for **5u**

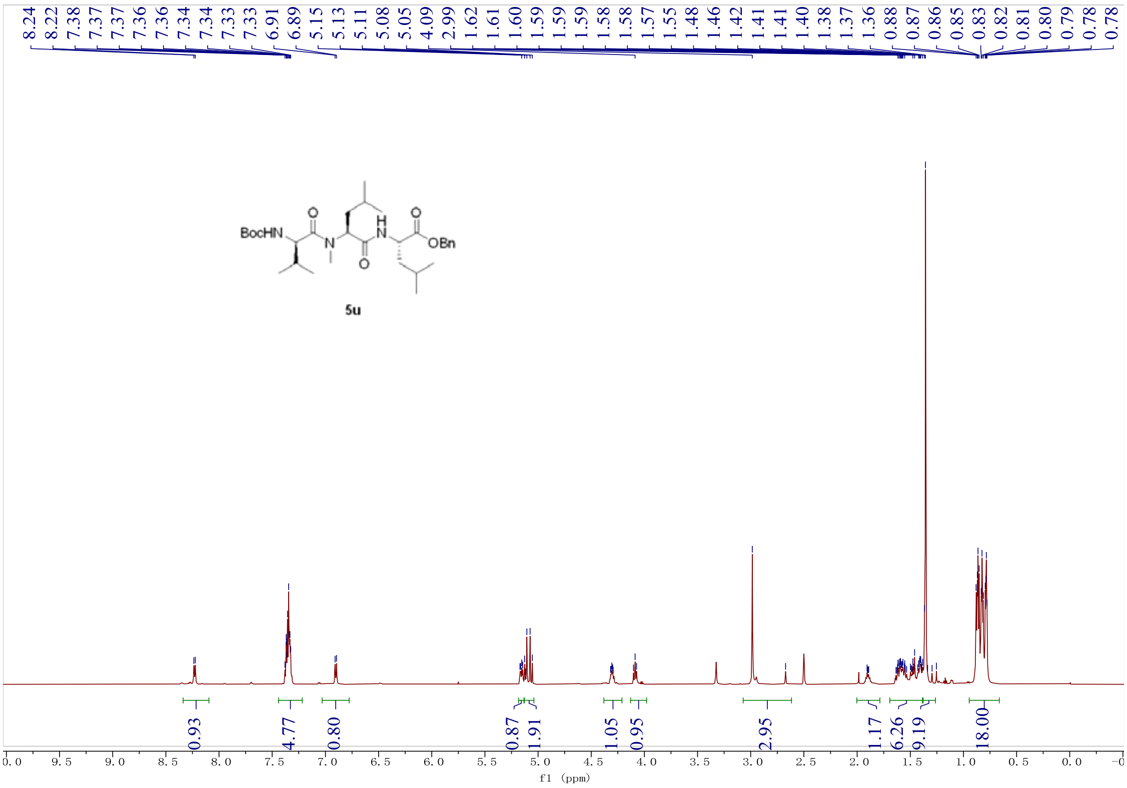

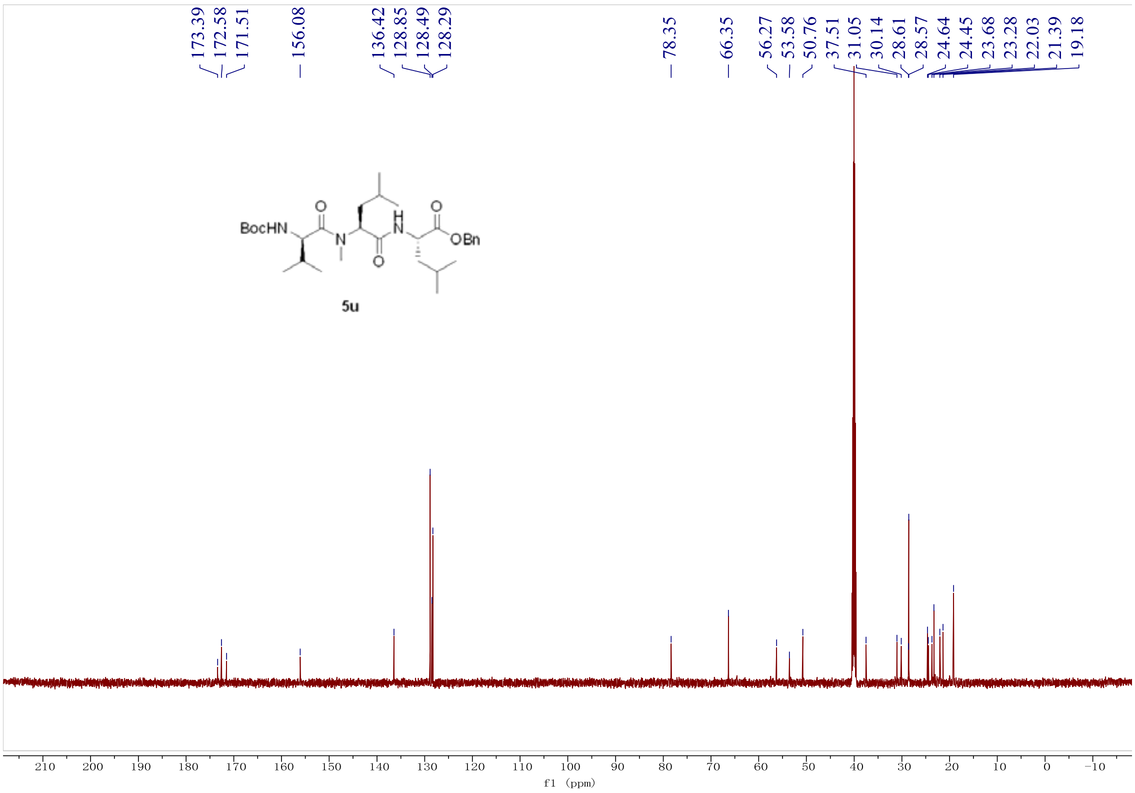

^{1}H NMR (DMSO, 600 MHz) and ^{13}C NMR (DMSO, 151 MHz) for **5v**

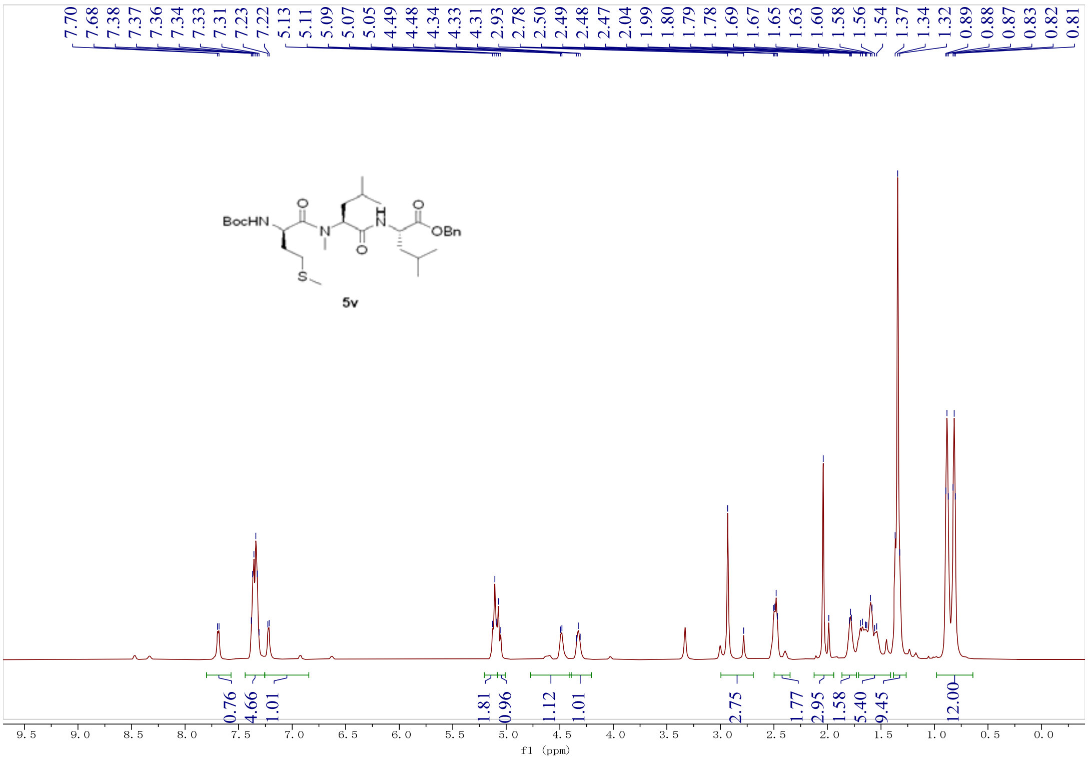

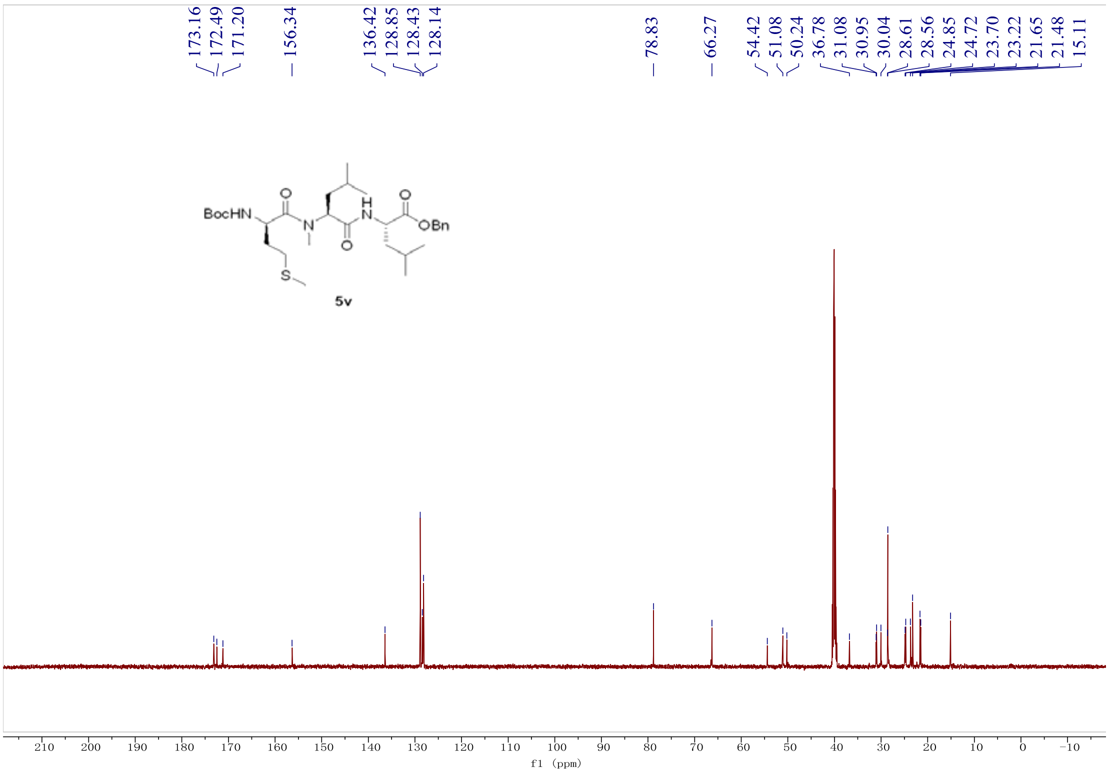

^{1}H NMR (DMSO, 600 MHz) and ^{13}C NMR (DMSO, 151 MHz) for **5w**

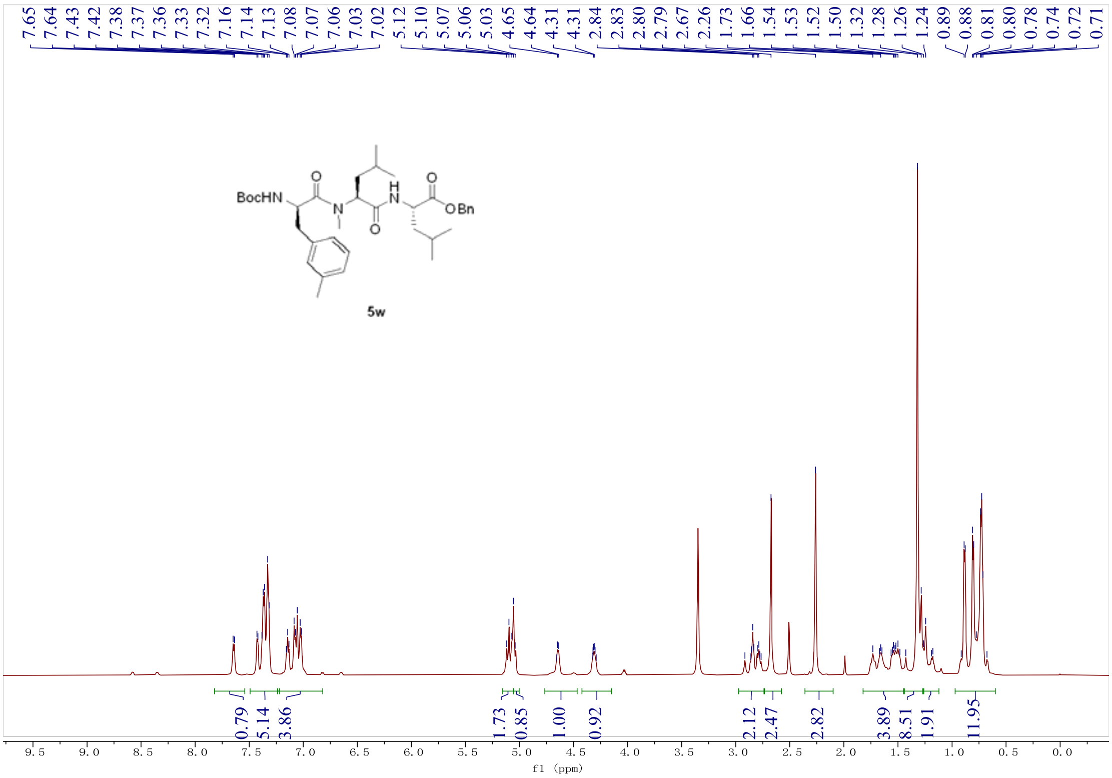

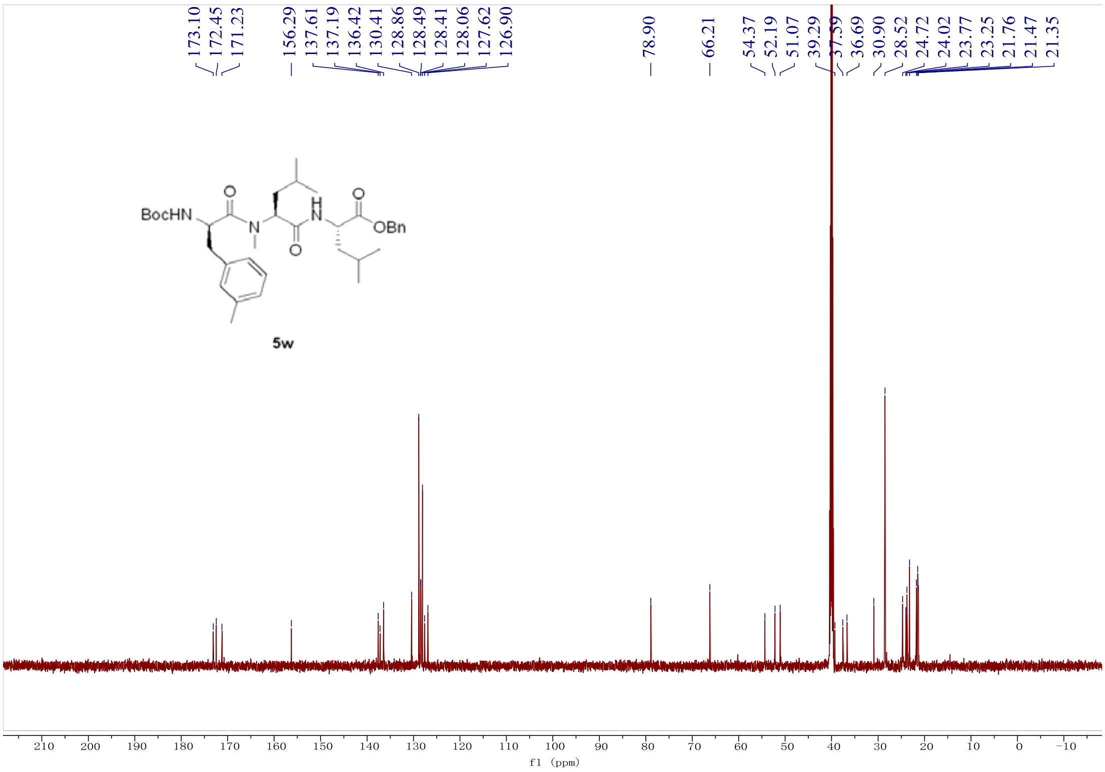

^{1}H NMR (DMSO, 600 MHz) and ^{13}C NMR (DMSO, 151 MHz) for **5x**

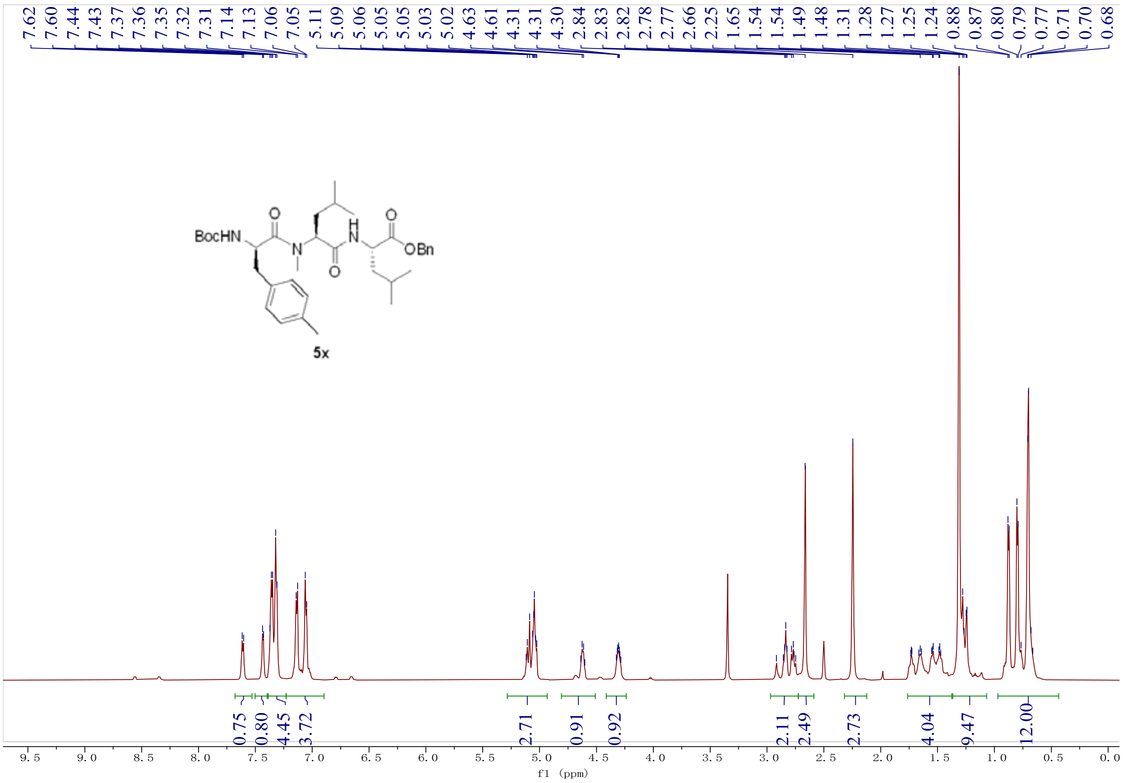

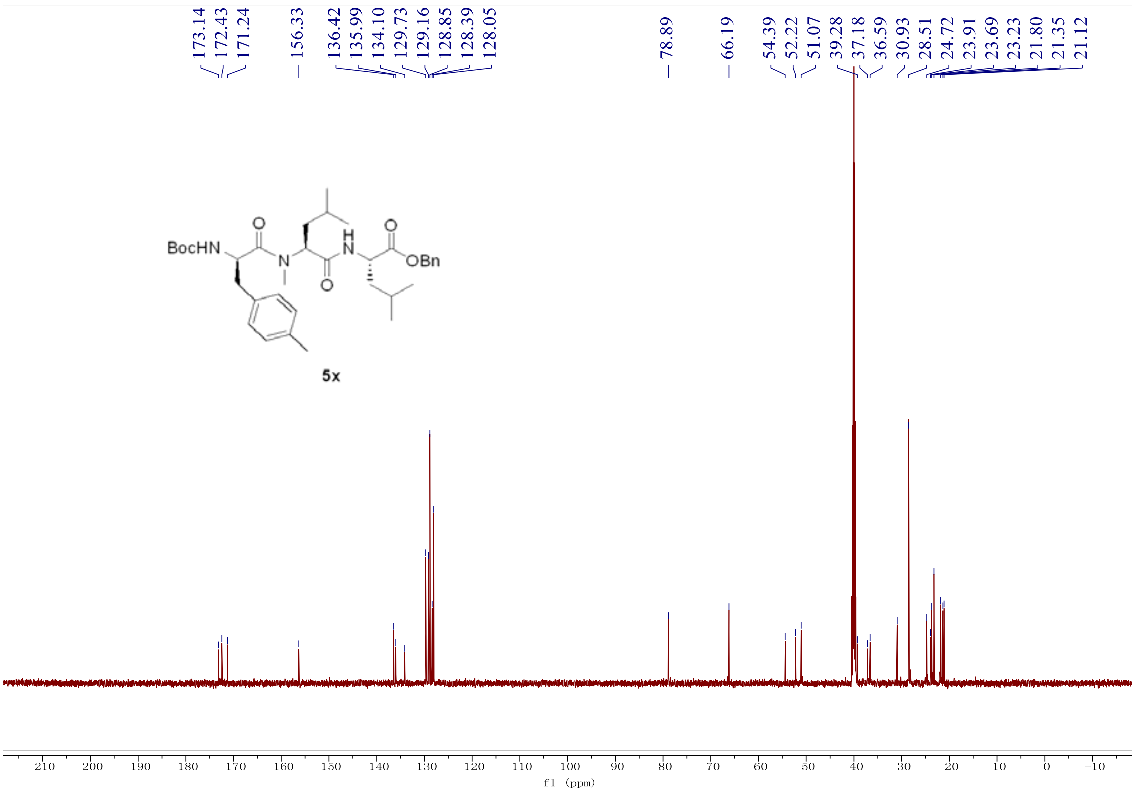

^{1}H NMR (DMSO, 600 MHz) and ^{13}C NMR (DMSO, 151 MHz) for **8a**

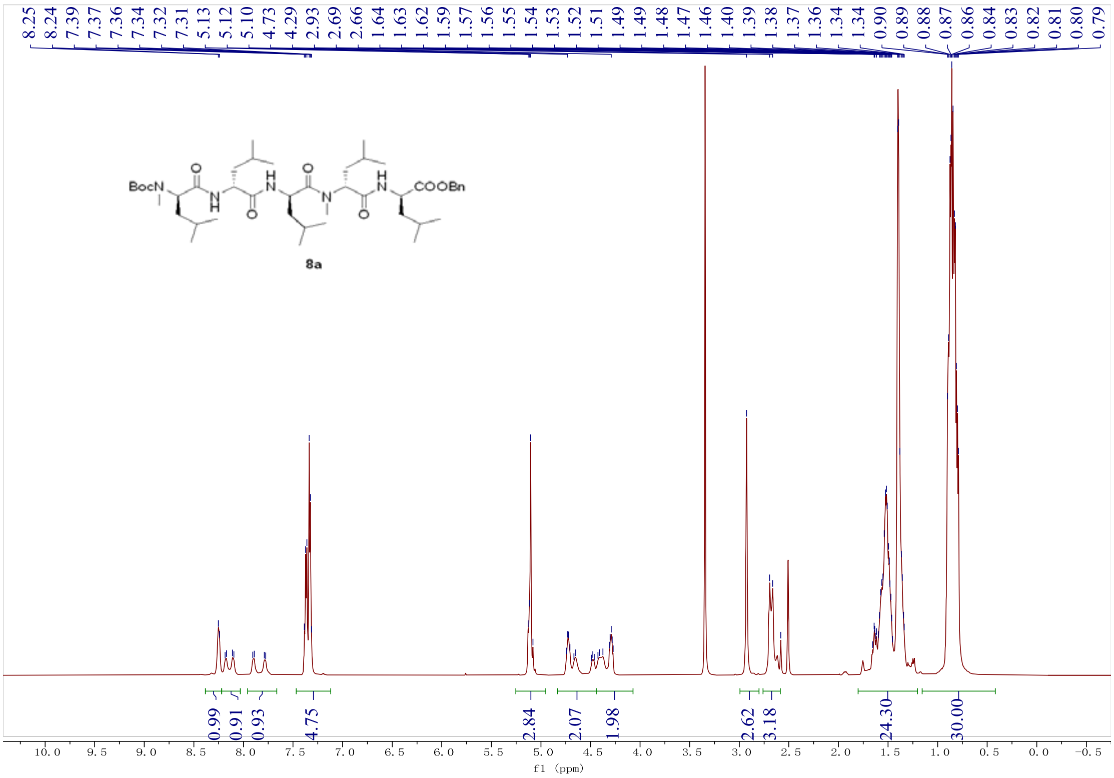

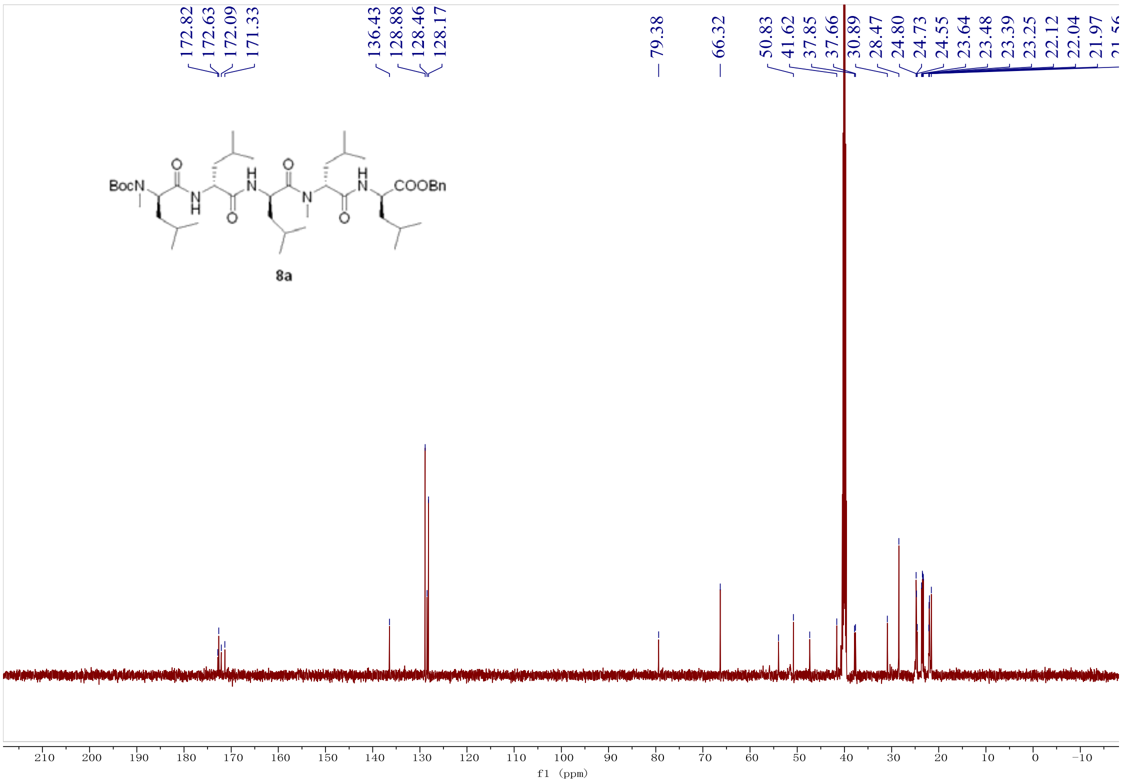

^{1}H NMR (DMSO, 600 MHz) and ^{13}C NMR (DMSO, 151 MHz) for **8b**

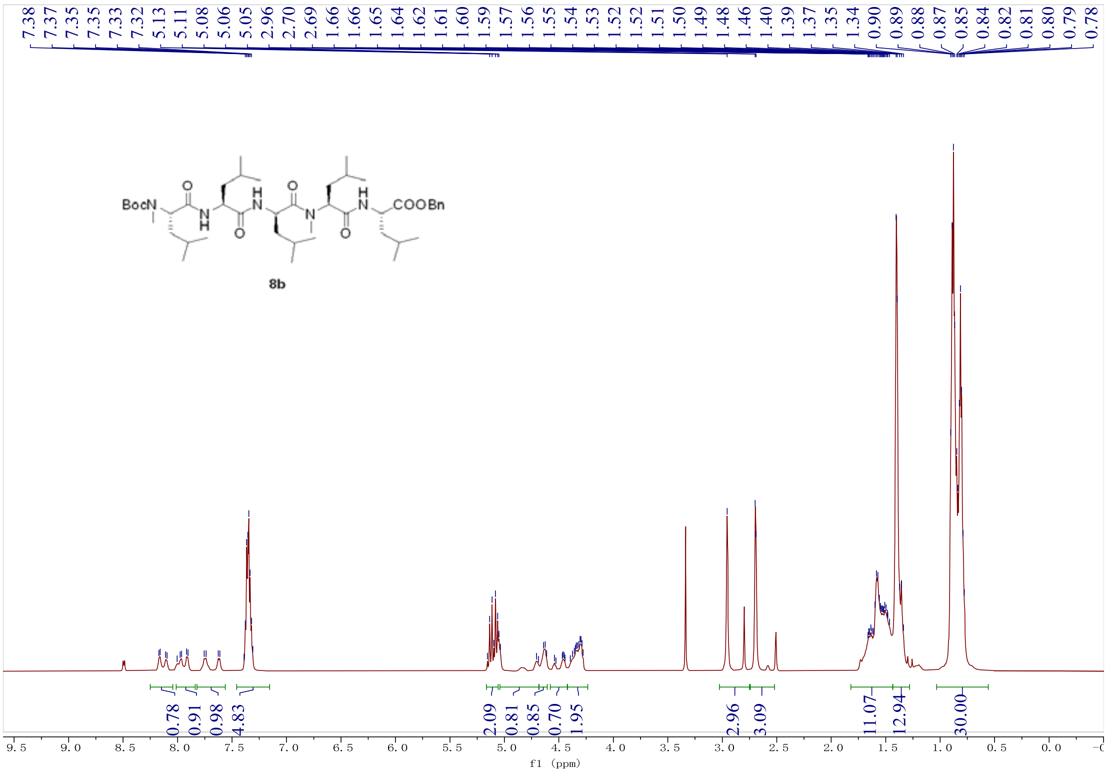

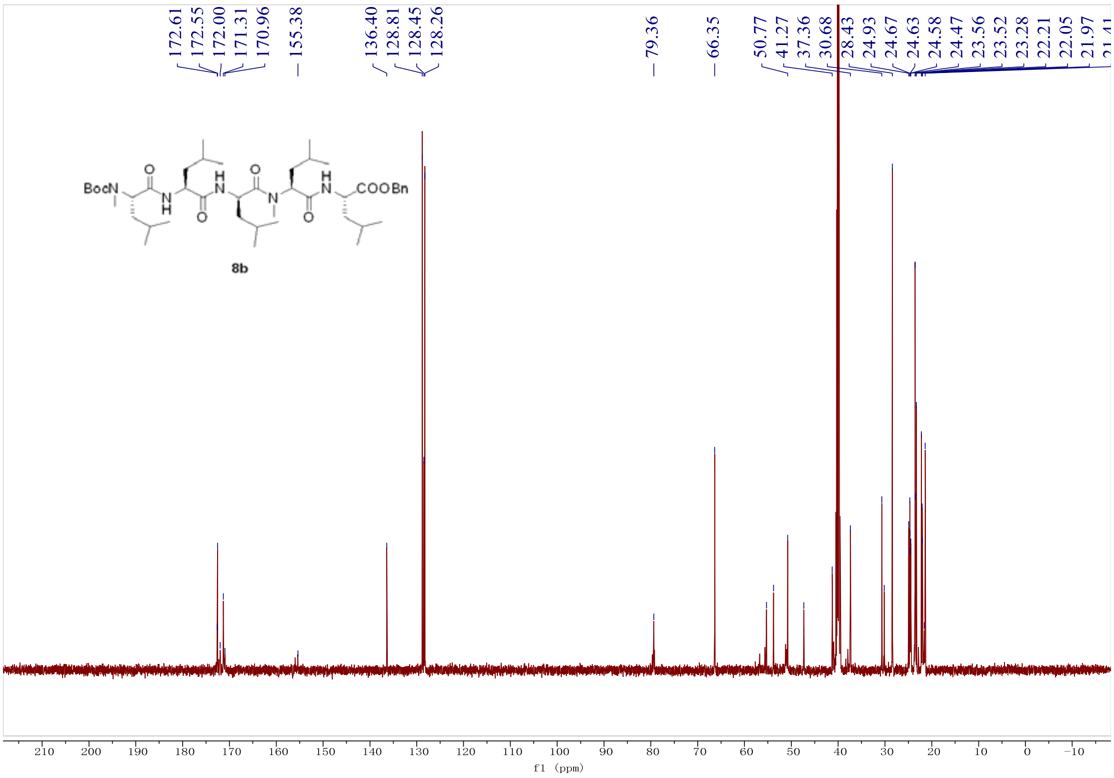

^{1}H NMR (DMSO, 600 MHz) and ^{13}C NMR (DMSO, 151 MHz) for **8c**

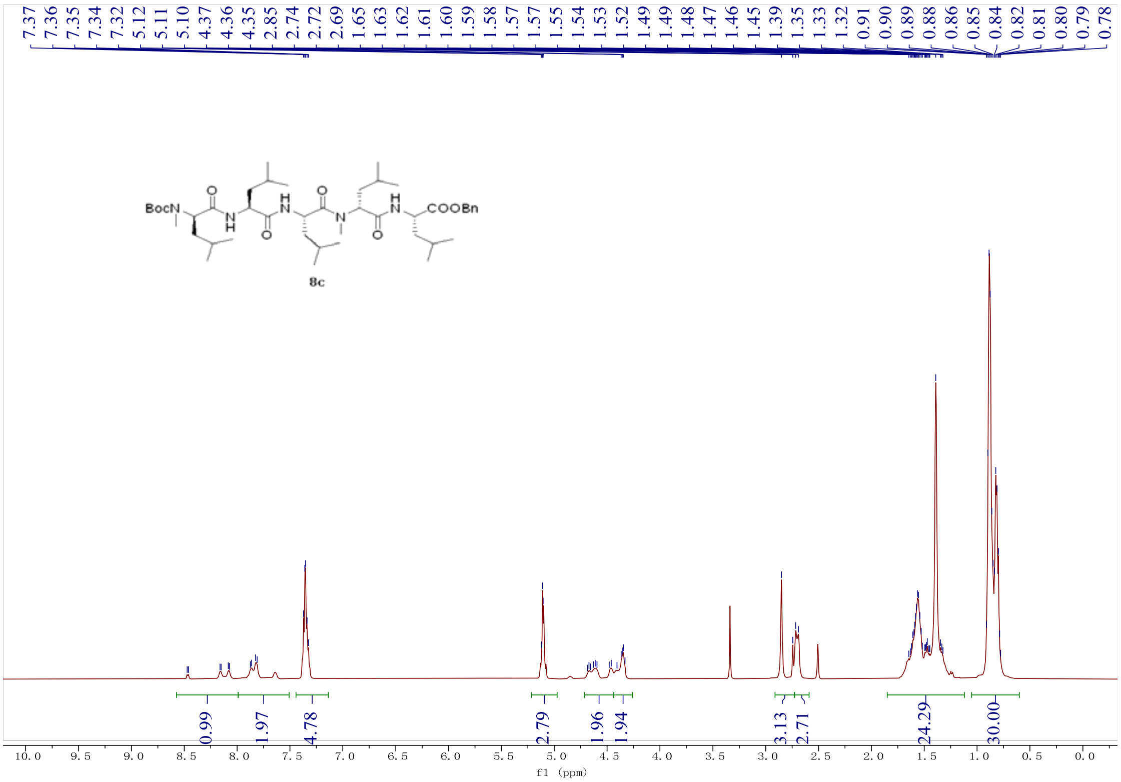

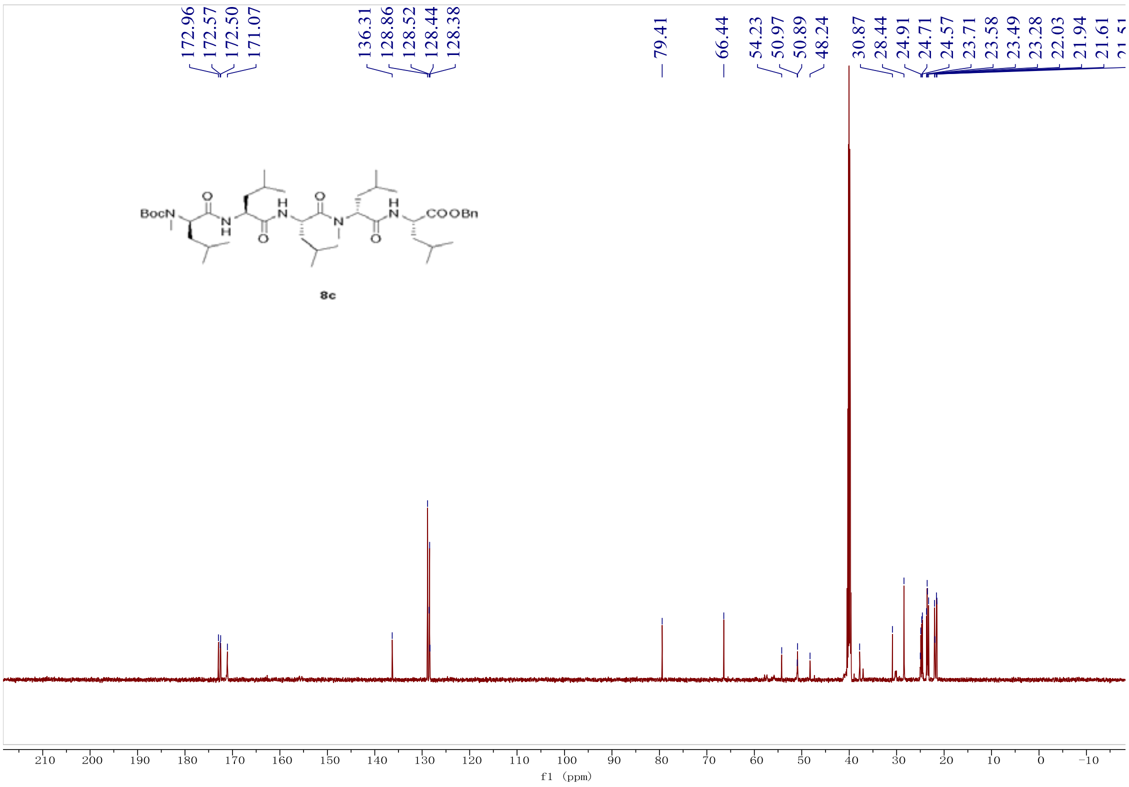

^{1}H NMR (DMSO, 600 MHz) and ^{13}C NMR (DMSO, 151 MHz) for **8d**

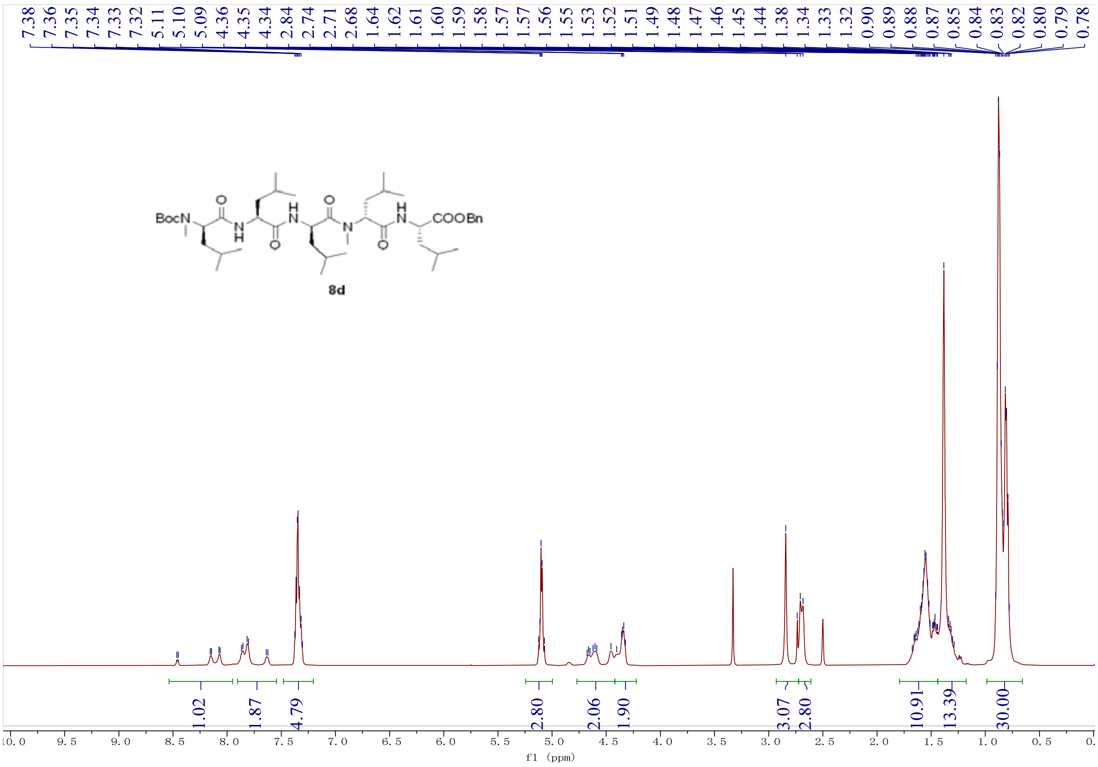

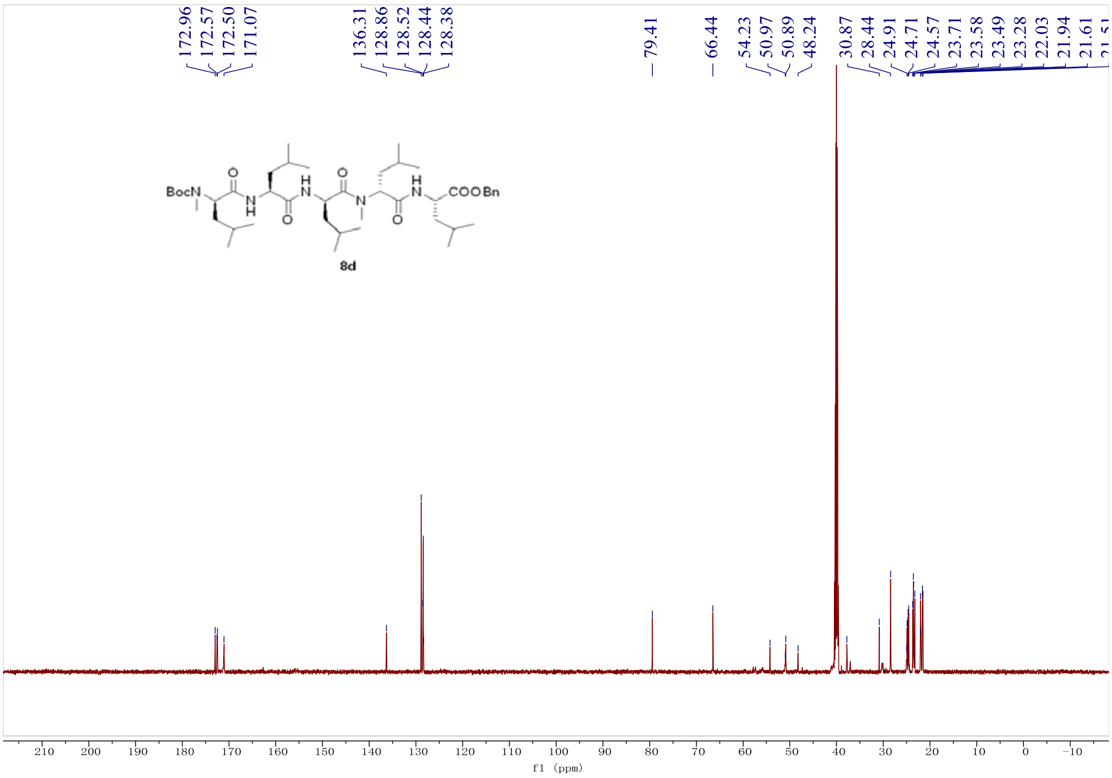

^{1}H NMR (DMSO, 600 MHz) and ^{13}C NMR (DMSO, 151 MHz) for **8e**

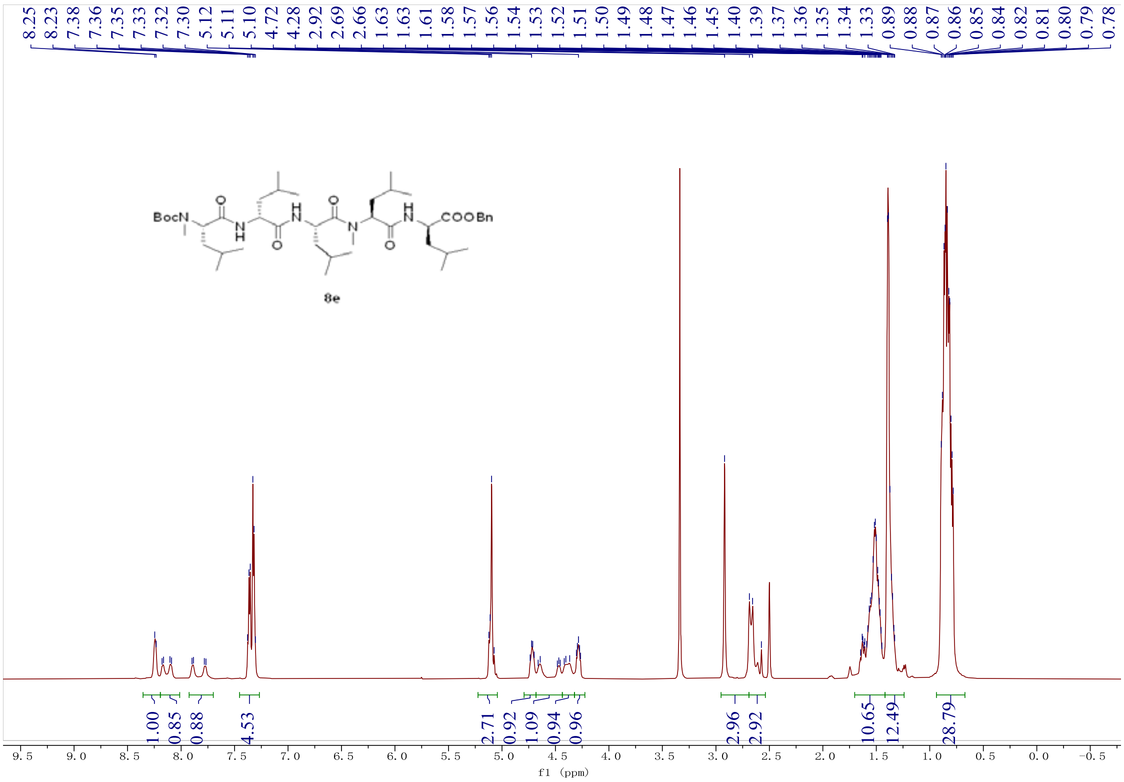

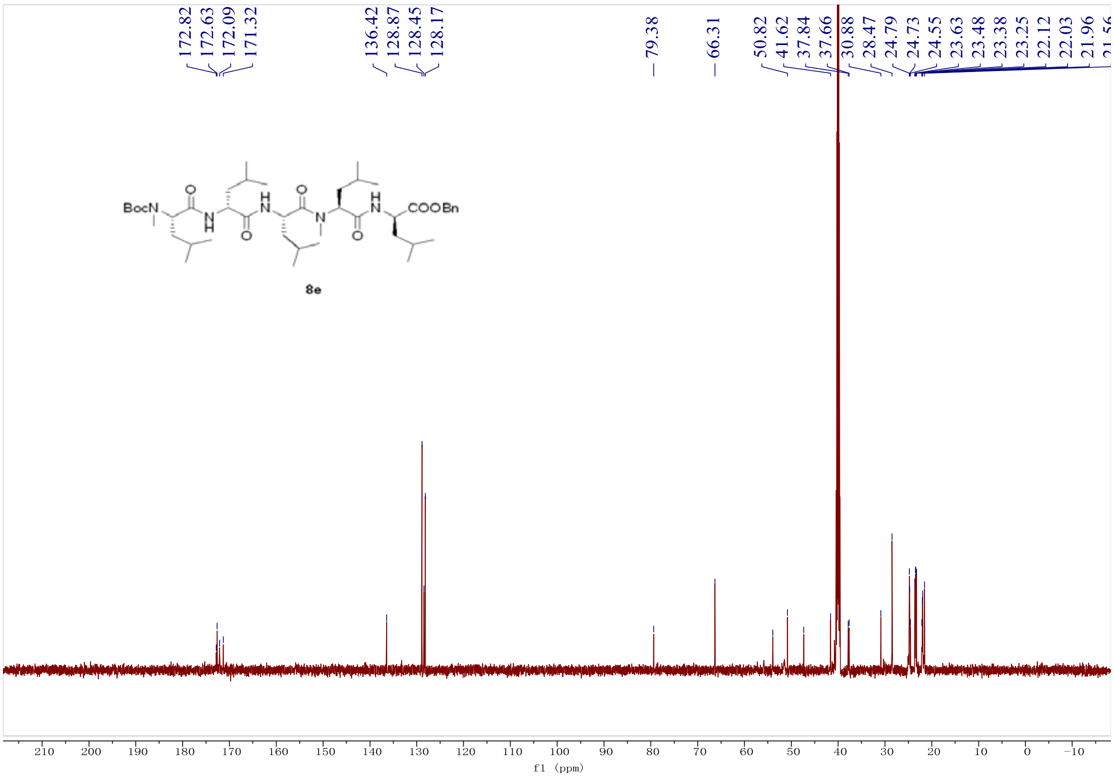

^{1}H NMR (DMSO, 600 MHz) and ^{13}C NMR (DMSO, 151 MHz) for **8f**

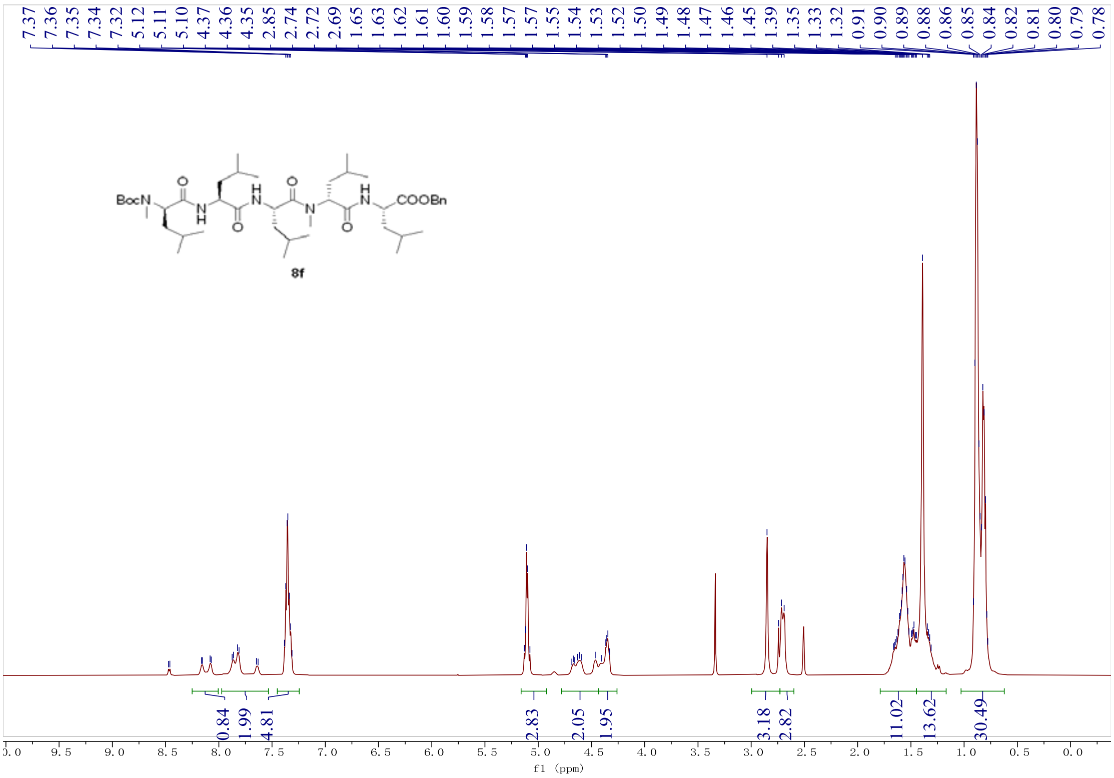

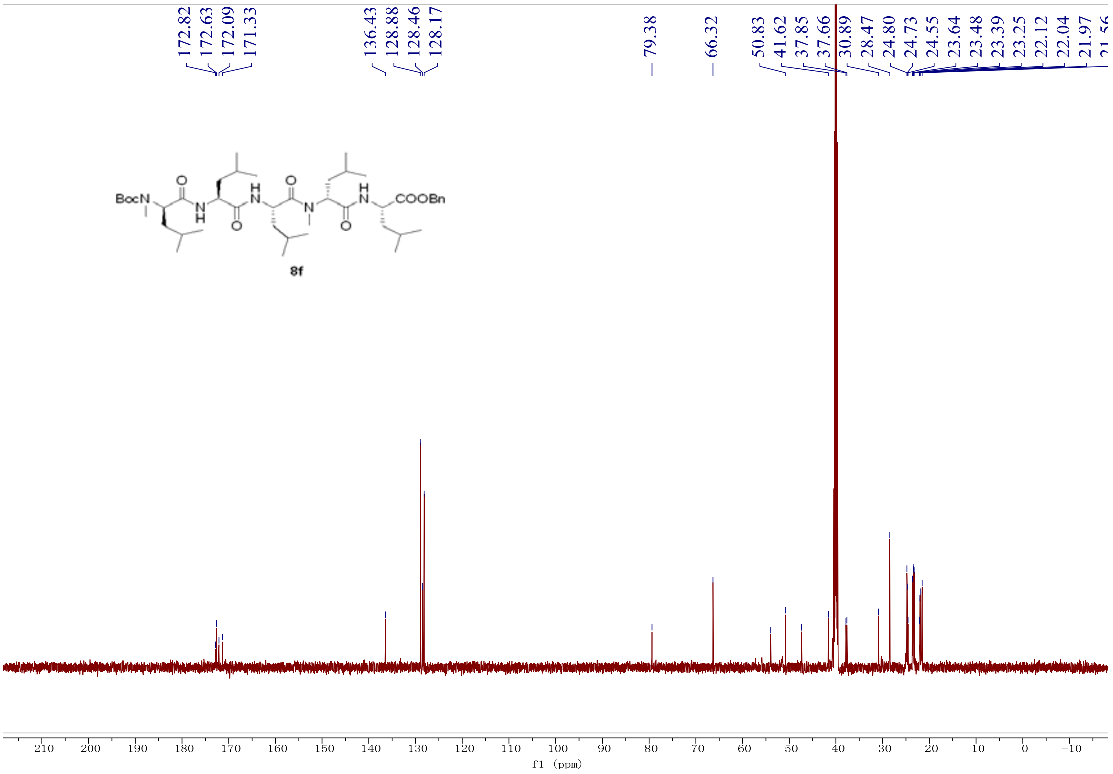

^{1}H NMR (DMSO, 600 MHz) and ^{13}C NMR (DMSO, 151 MHz) for **8g**

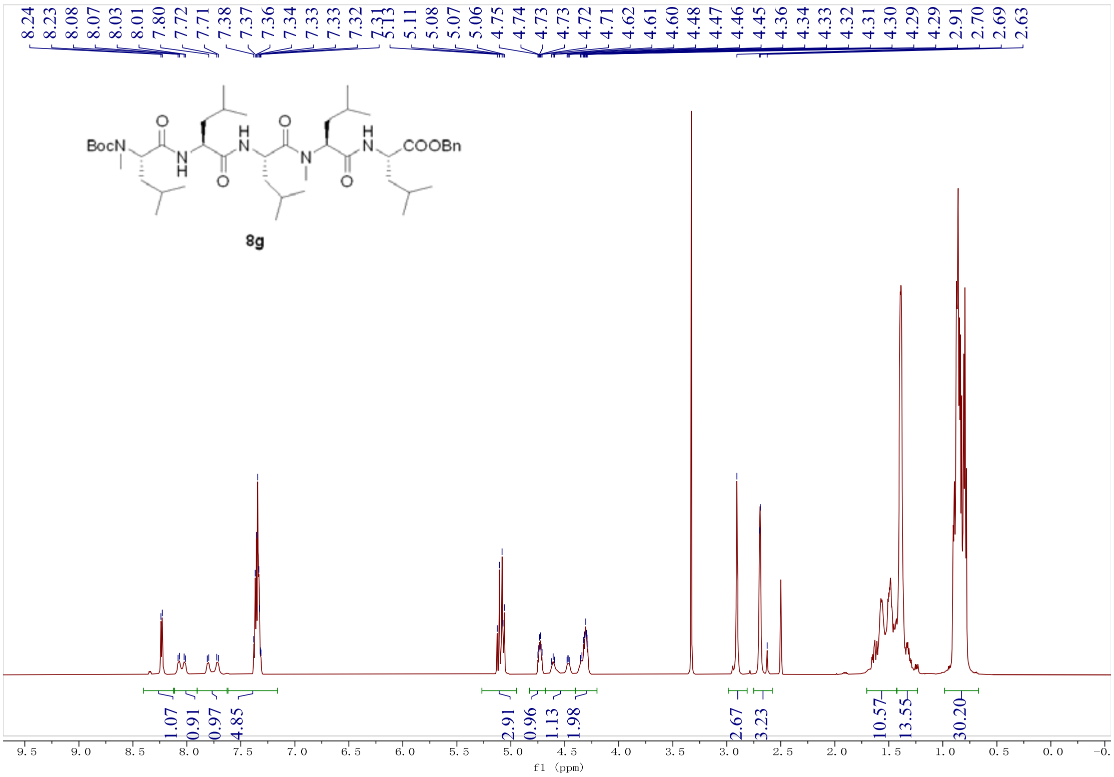

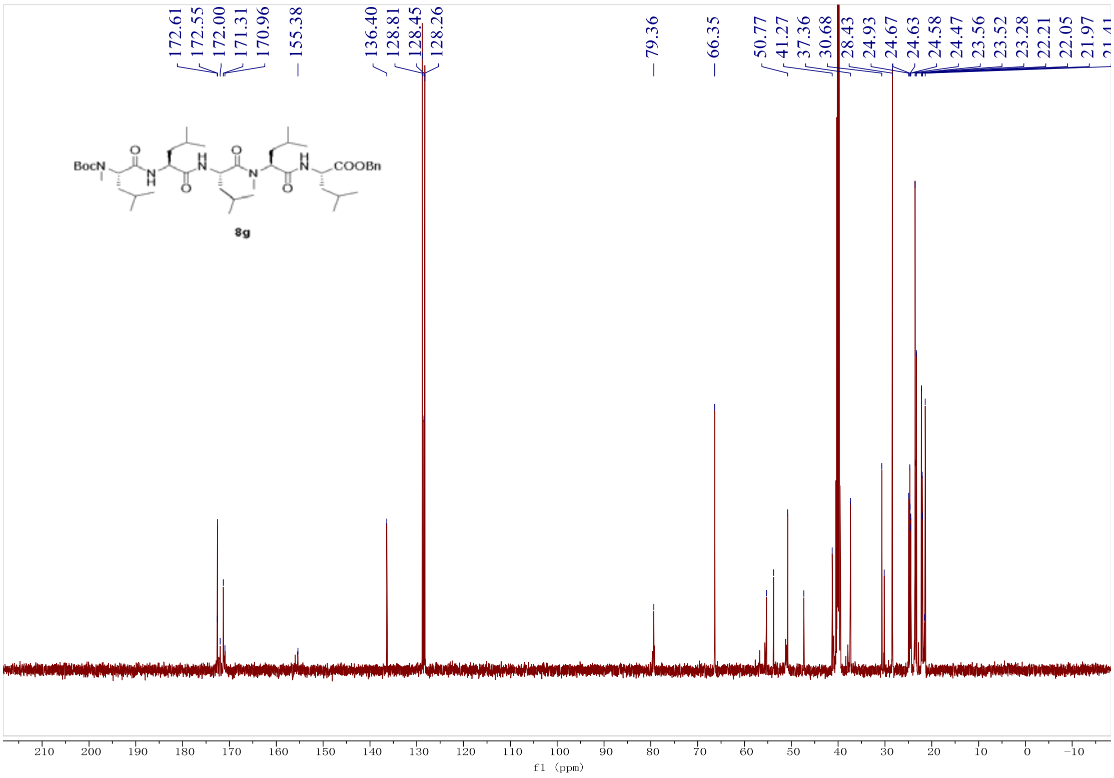

^{1}H NMR (DMSO, 600 MHz) and ^{13}C NMR (DMSO, 151 MHz) for **8h**

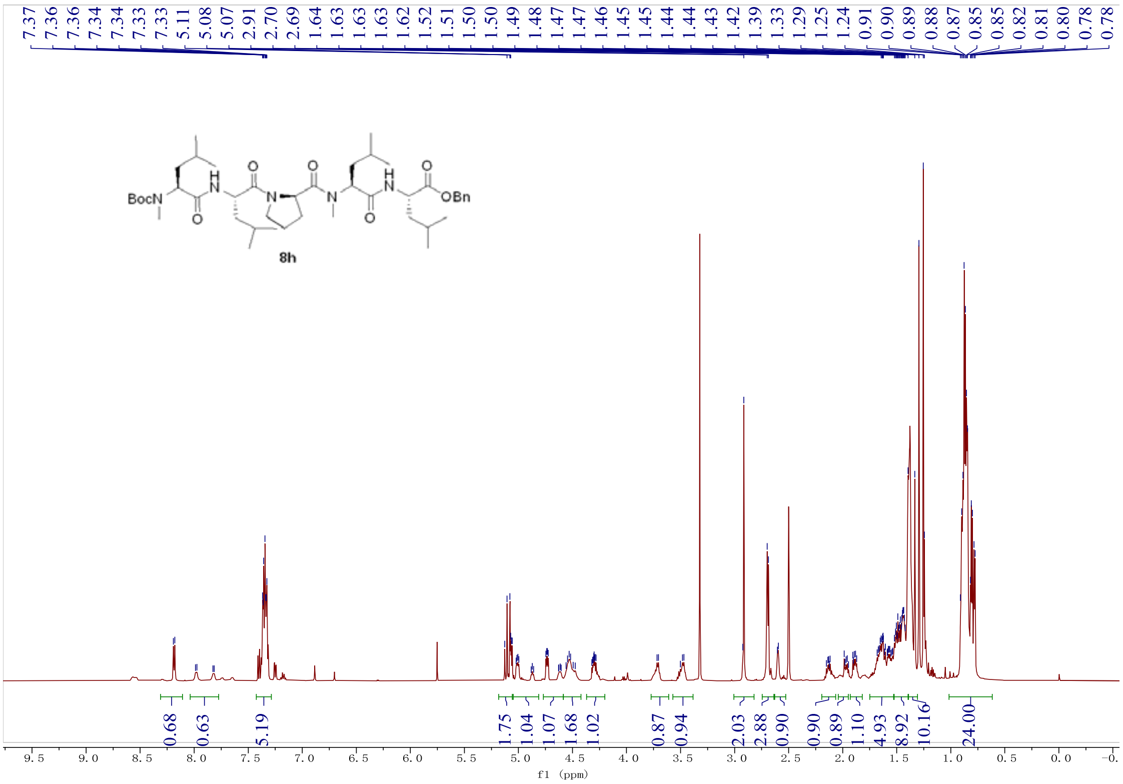

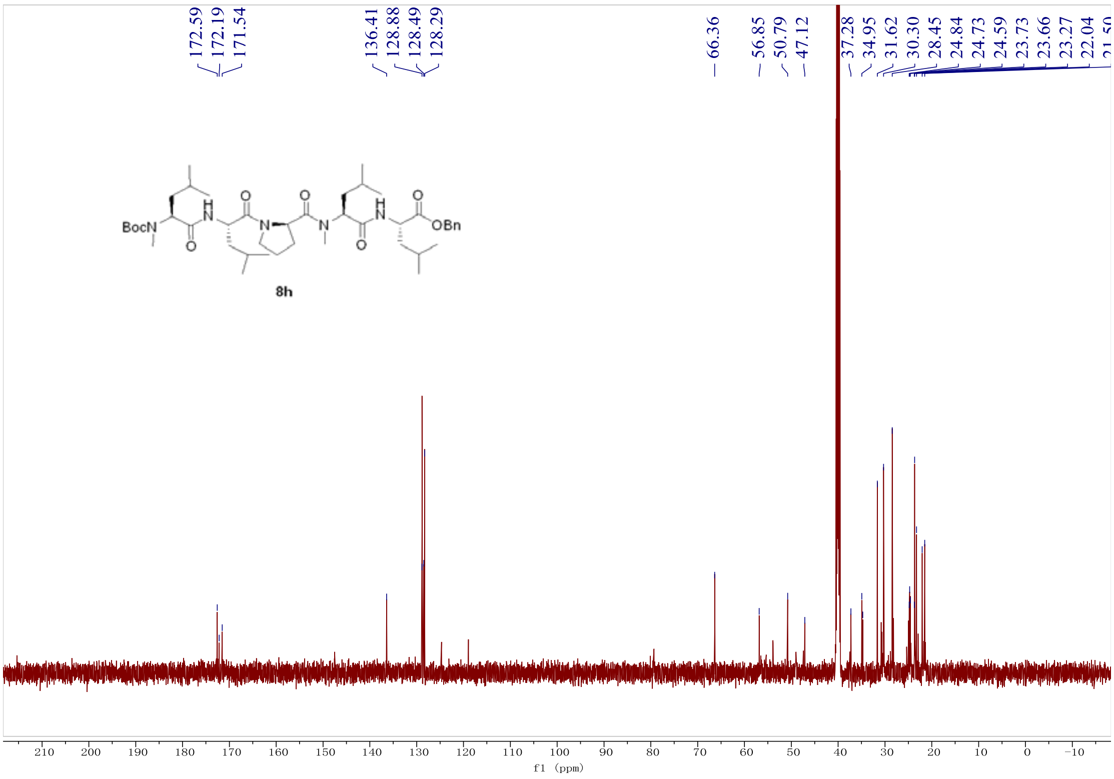

^{1}H NMR (DMSO, 600 MHz) and ^{13}C NMR (DMSO, 151 MHz) for **8i**

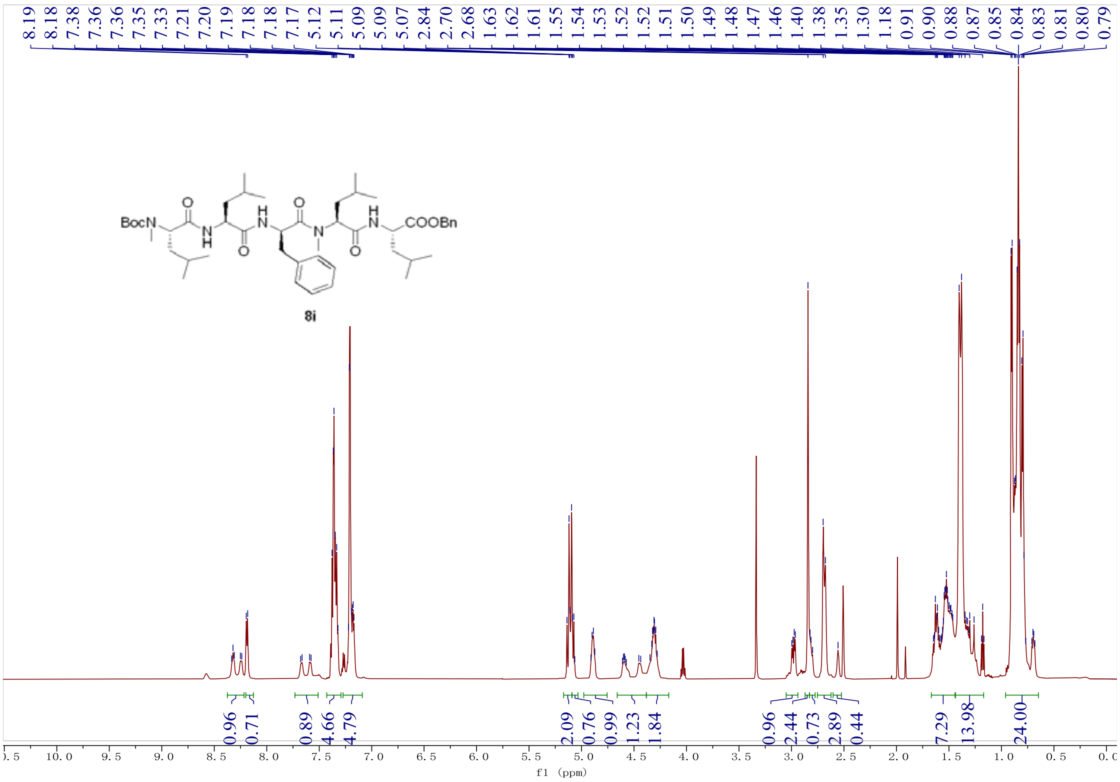

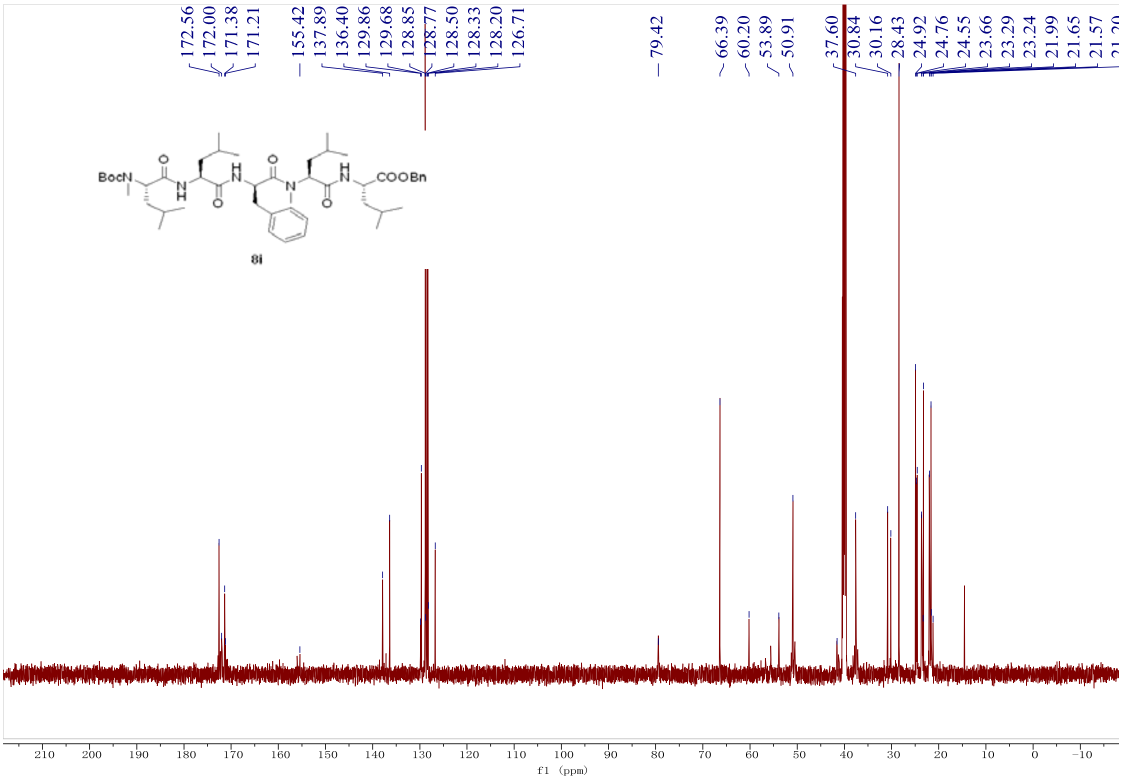

^{1}H NMR (DMSO, 600 MHz) and ^{13}C NMR (DMSO, 151 MHz) for **8j**

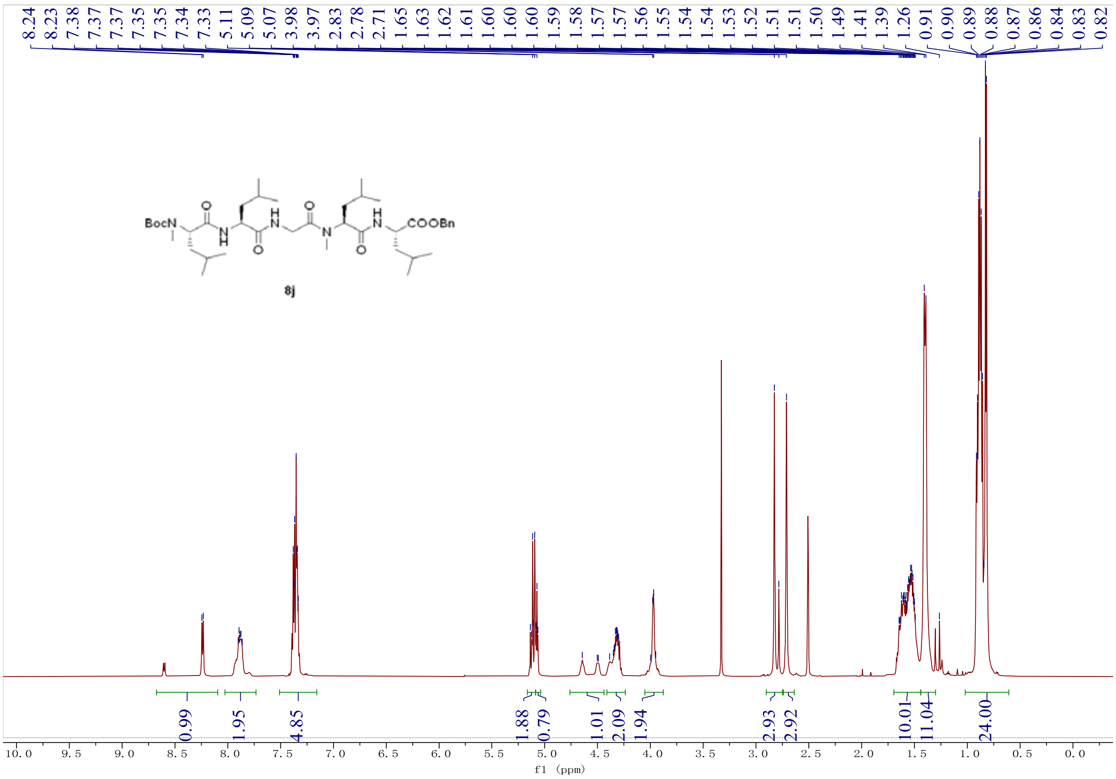

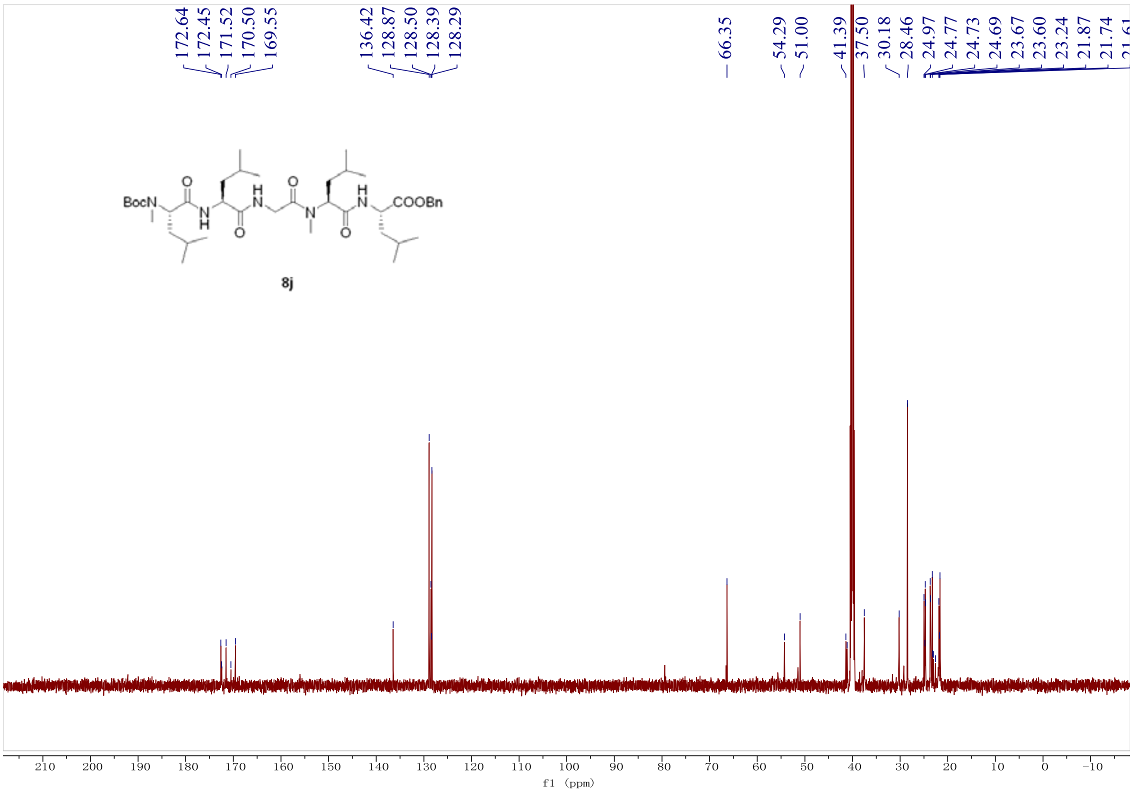

^{1}H NMR (DMSO, 600 MHz) and ^{13}C NMR (DMSO, 151 MHz) for **8k**

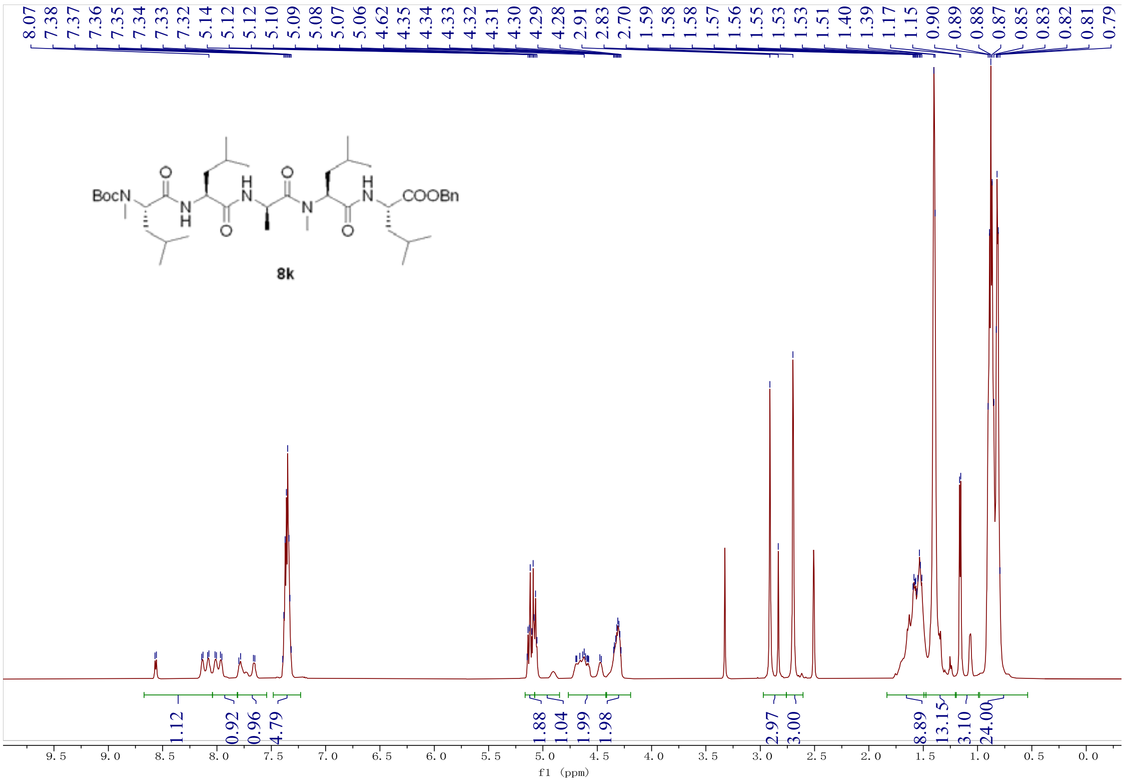

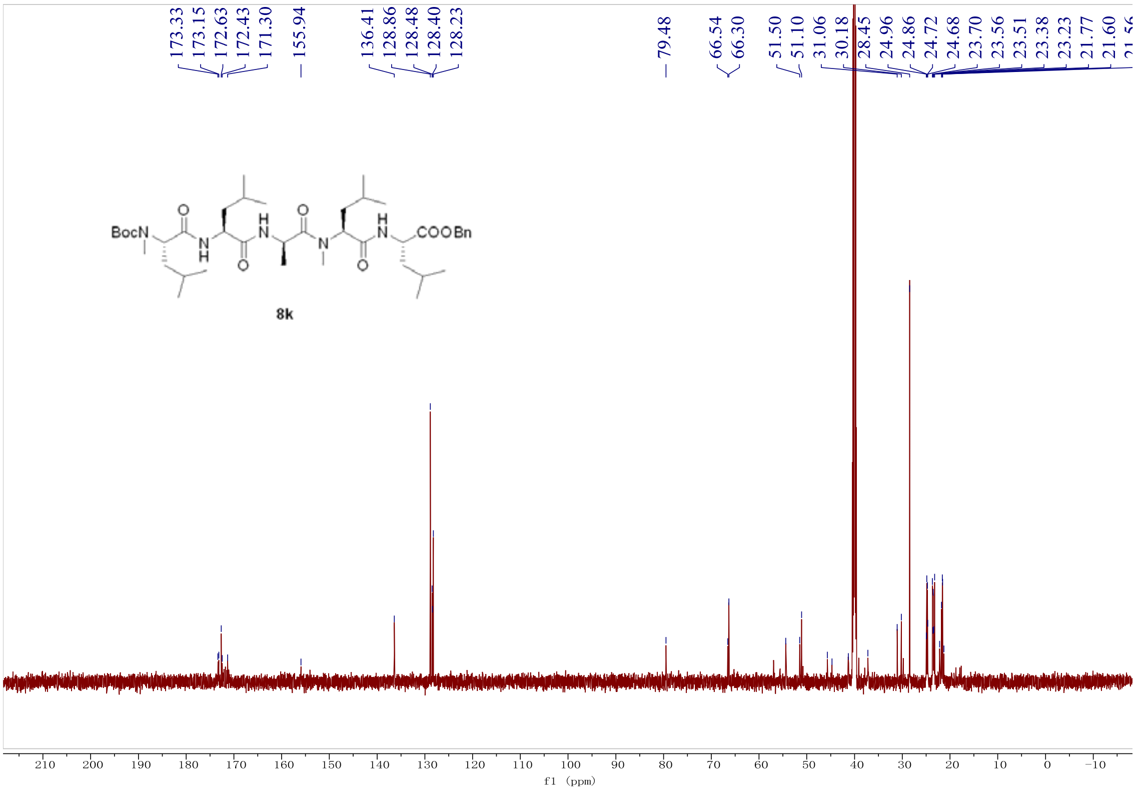

^{1}H NMR (DMSO, 600 MHz) and ^{13}C NMR (DMSO, 151 MHz) for **8l**

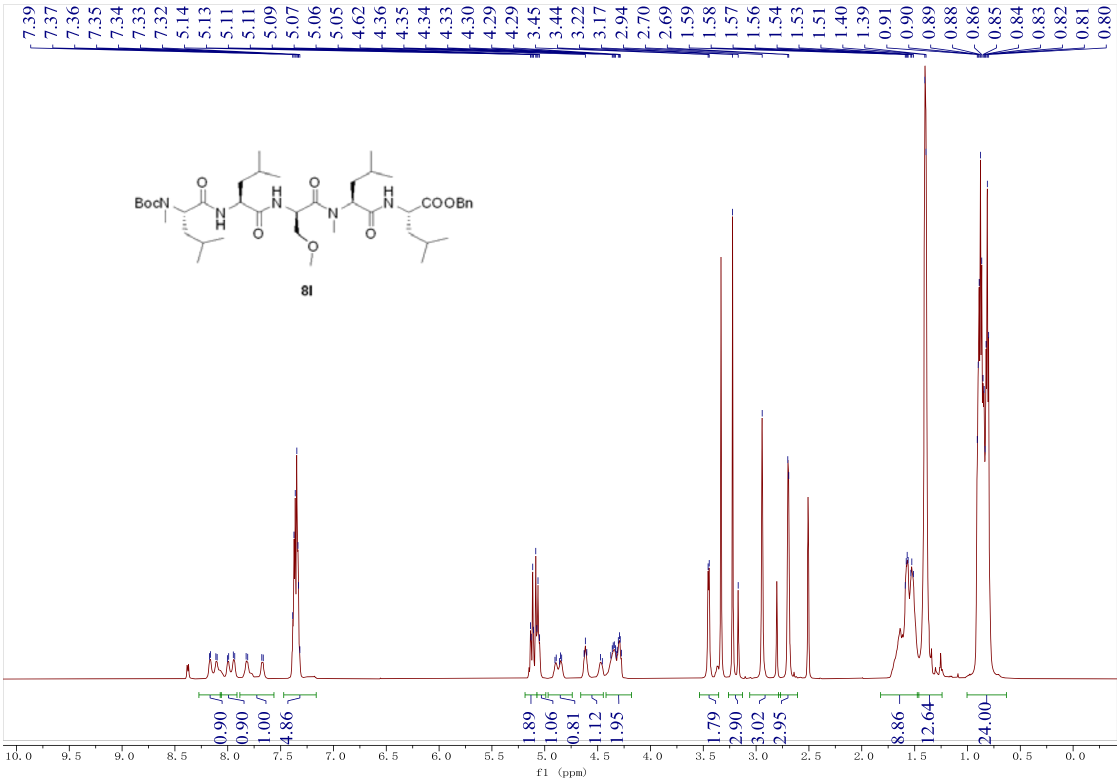

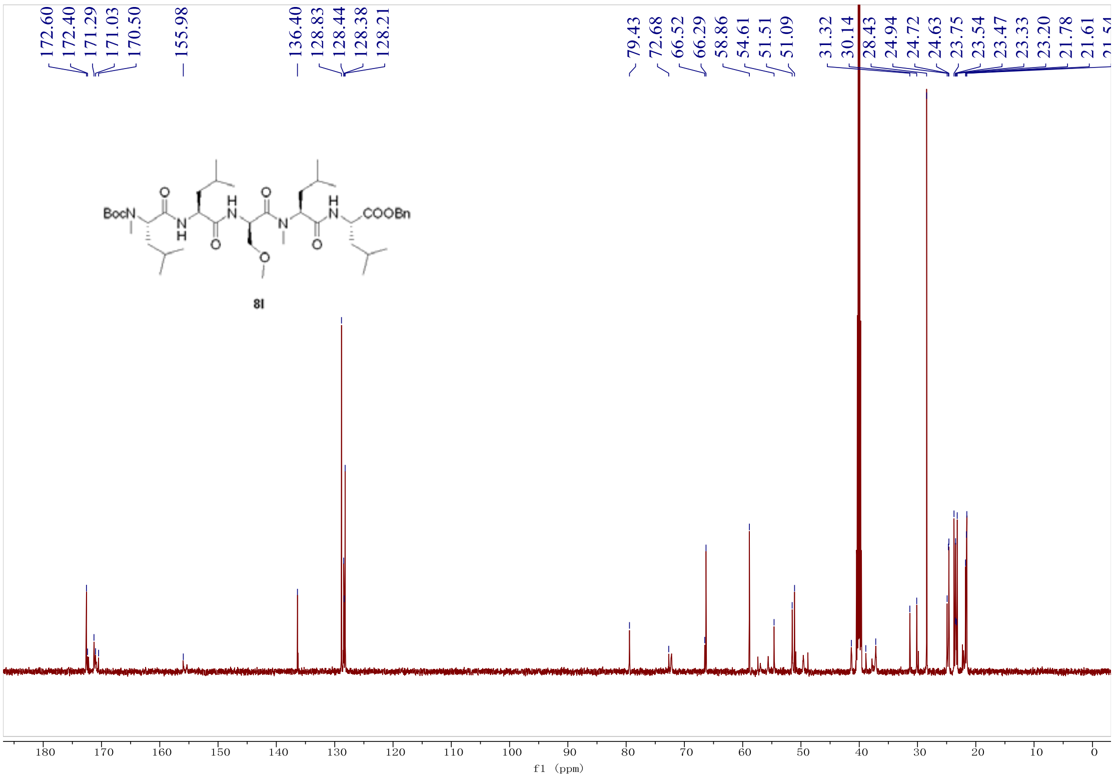

^{1}H NMR (DMSO, 600 MHz) and ^{13}C NMR (DMSO, 151 MHz) for **8m**

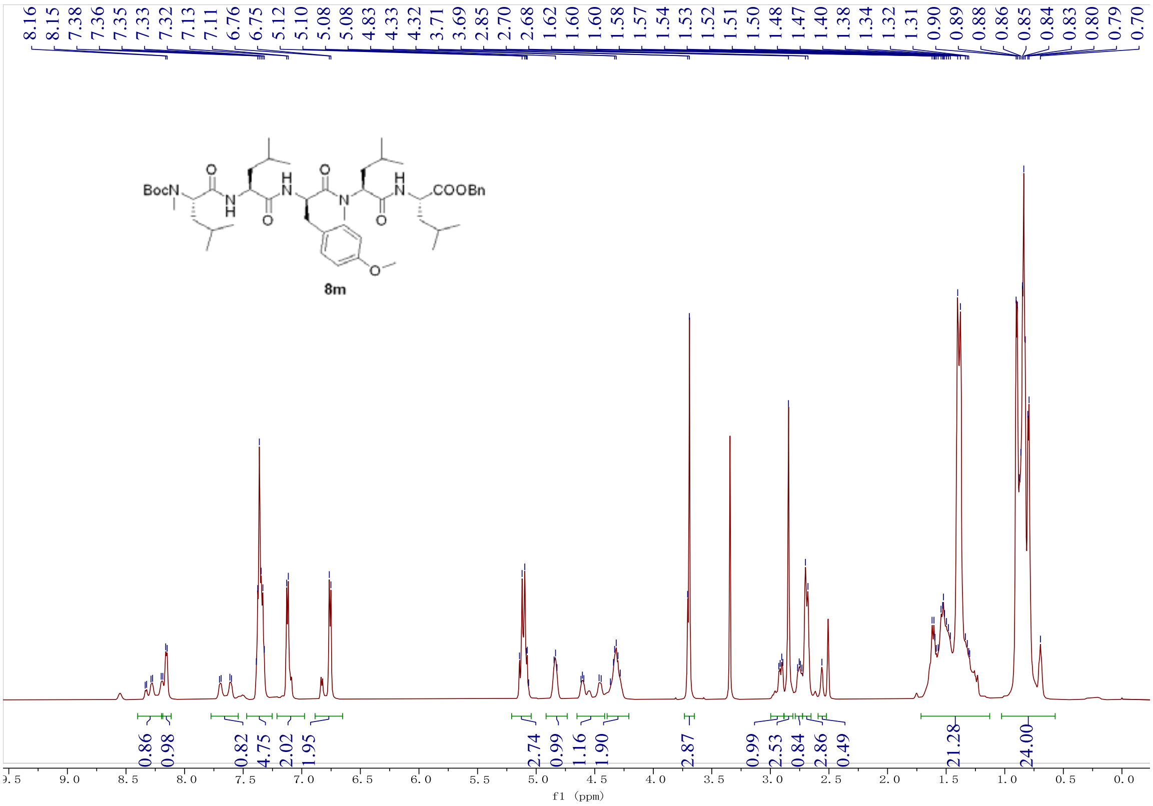

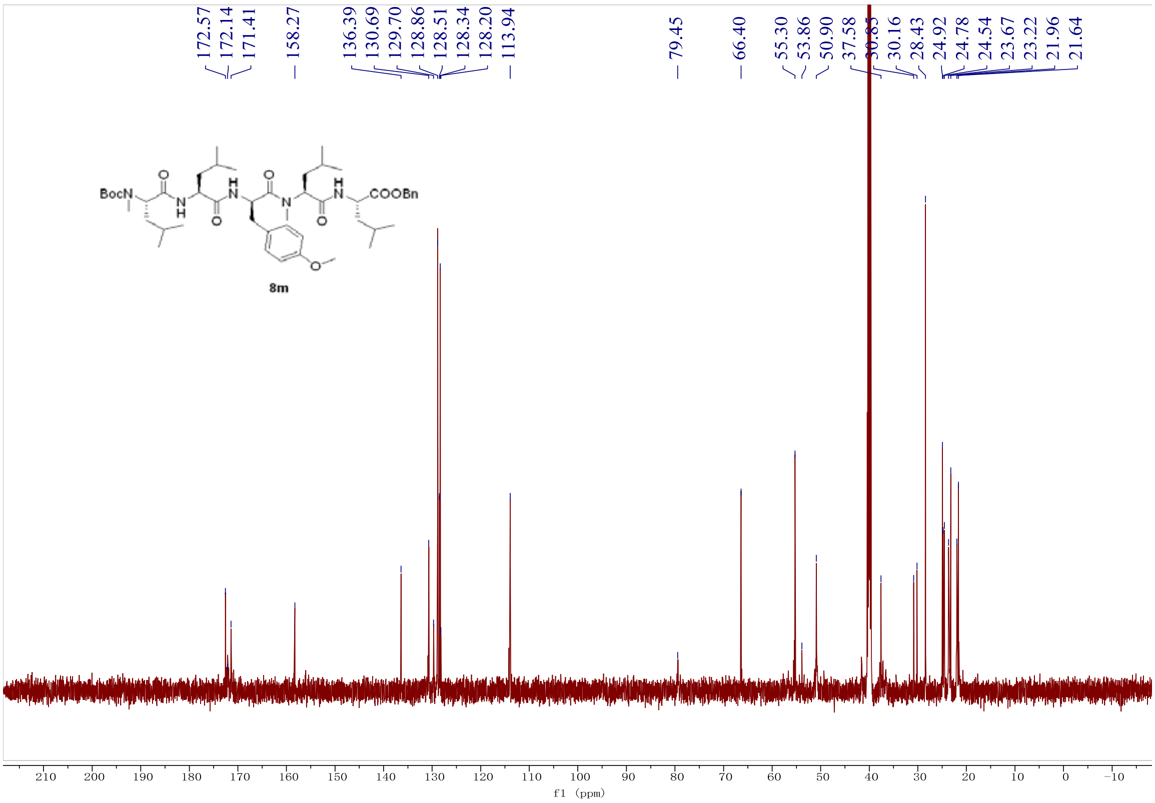

^{1}H NMR (DMSO, 600 MHz) and ^{13}C NMR (DMSO, 151 MHz) for **8n**

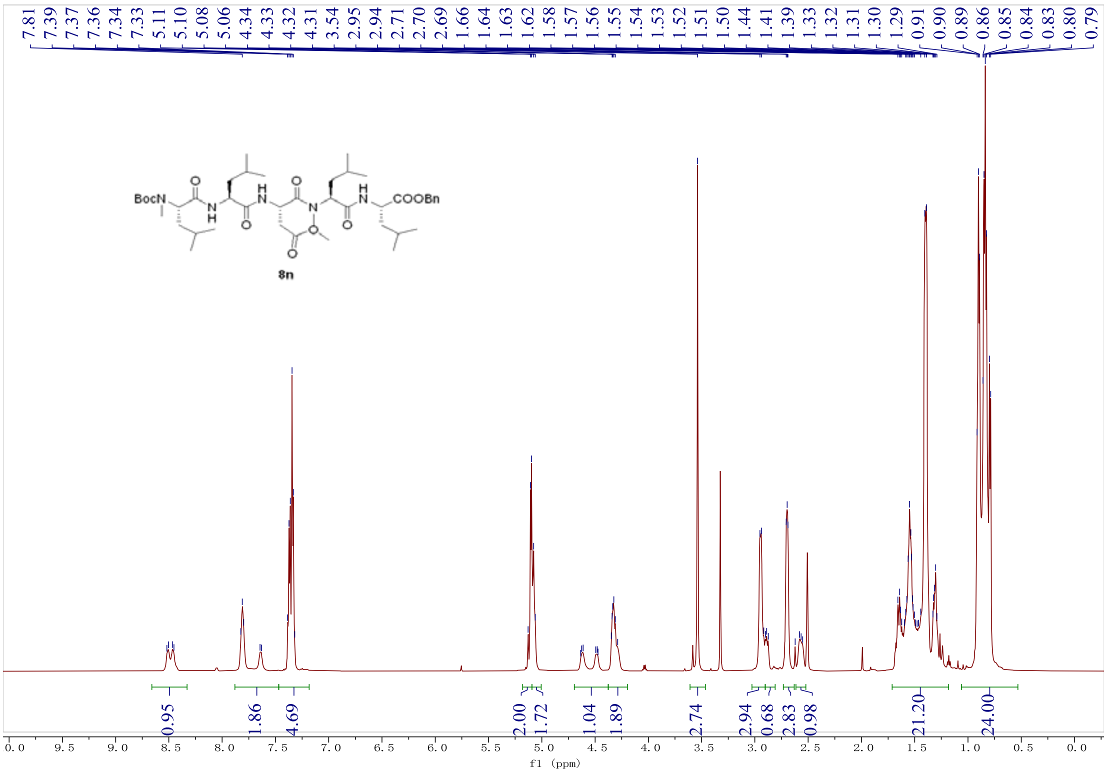

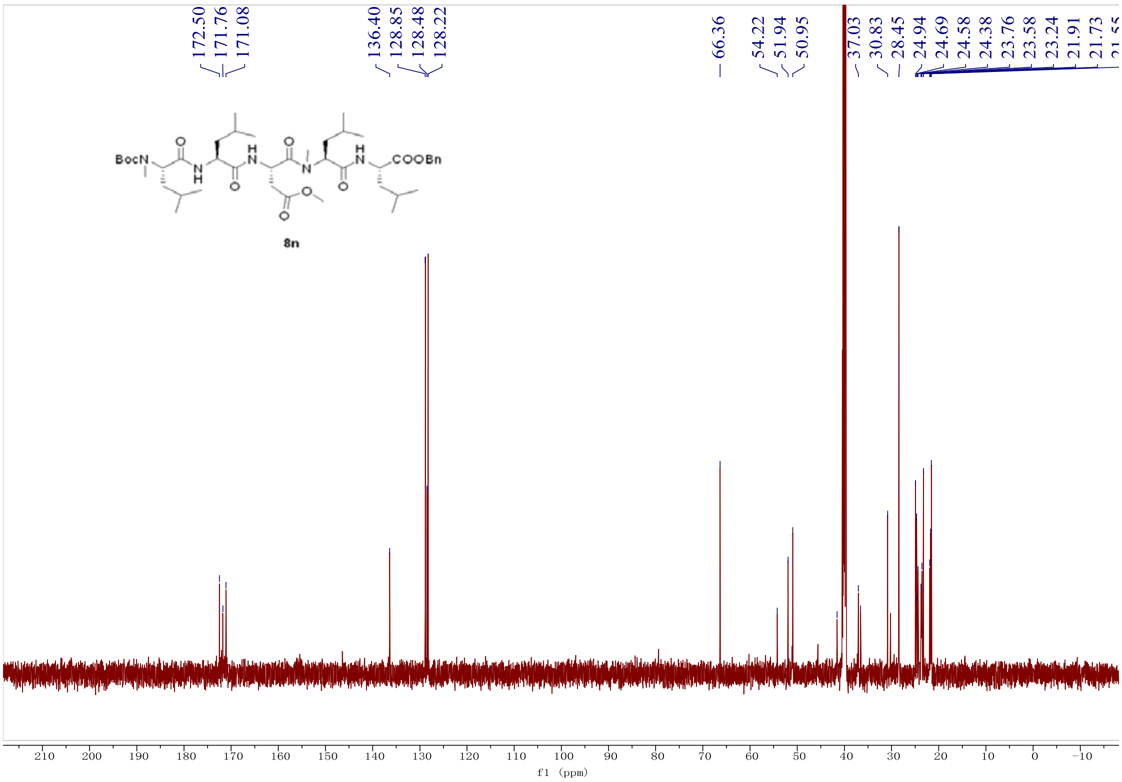

^{1}H NMR (DMSO, 600 MHz) and ^{13}C NMR (DMSO, 151 MHz) for **8o**

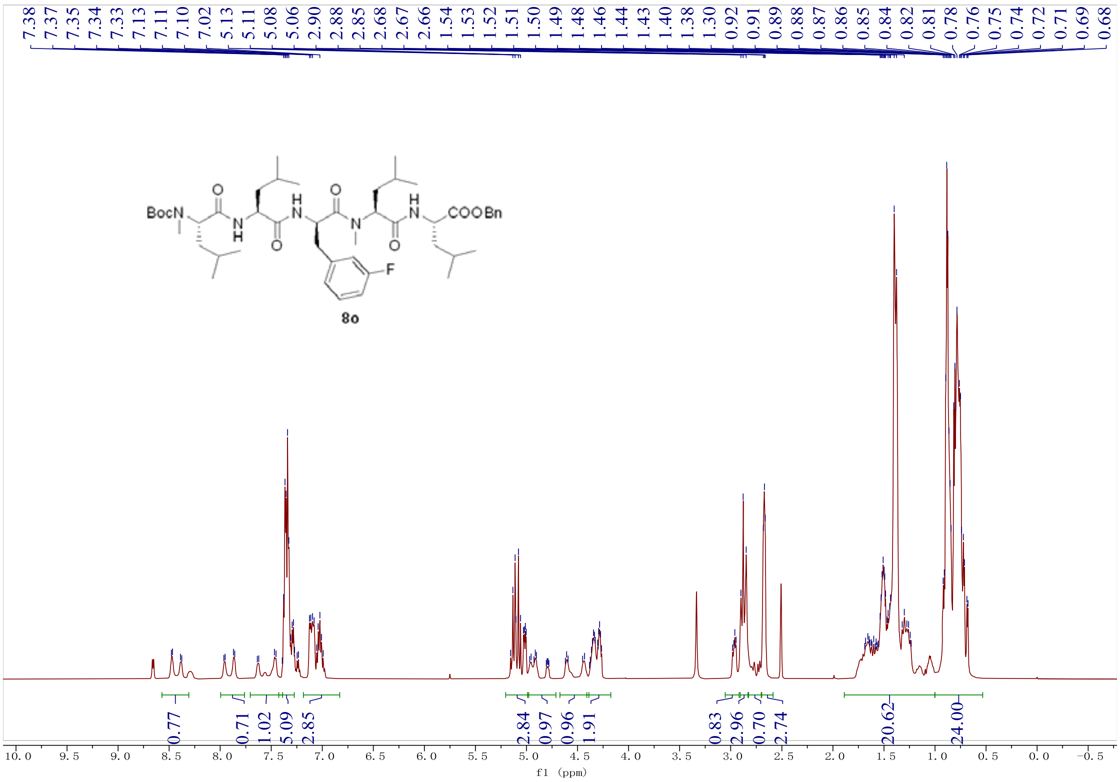

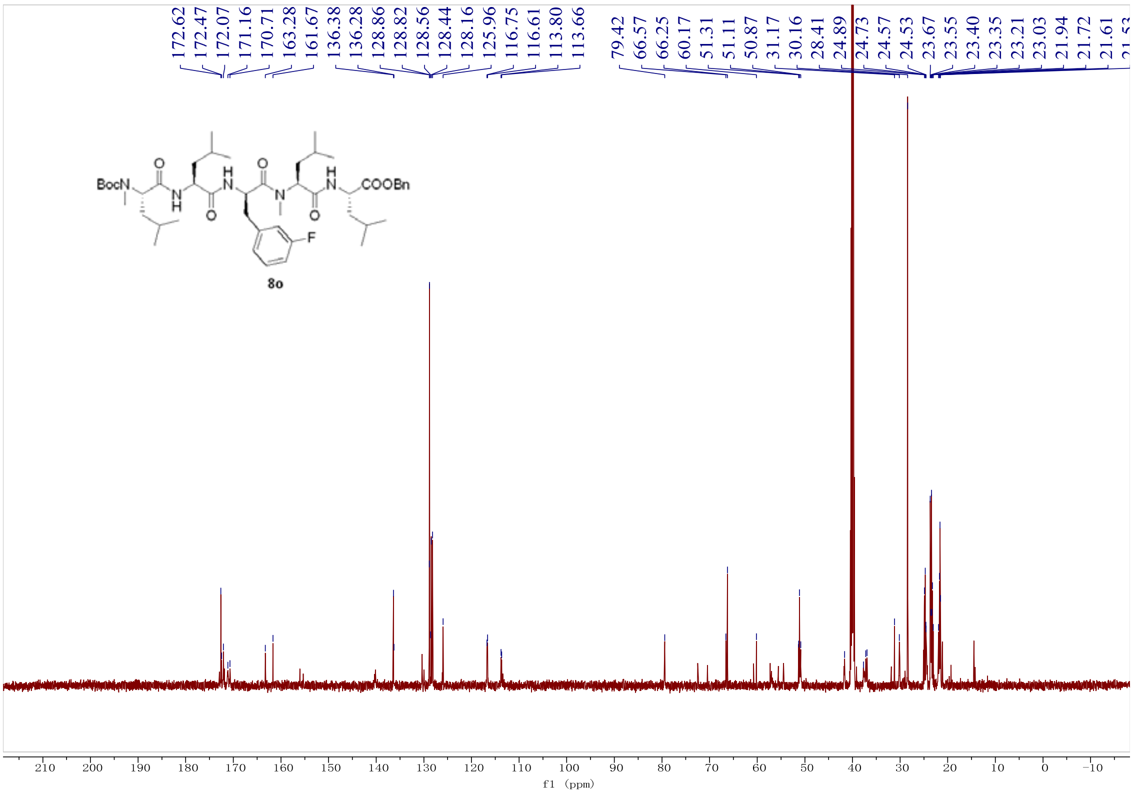

^{1}H NMR (DMSO, 600 MHz) and ^{13}C NMR (DMSO, 151 MHz) for **8p**

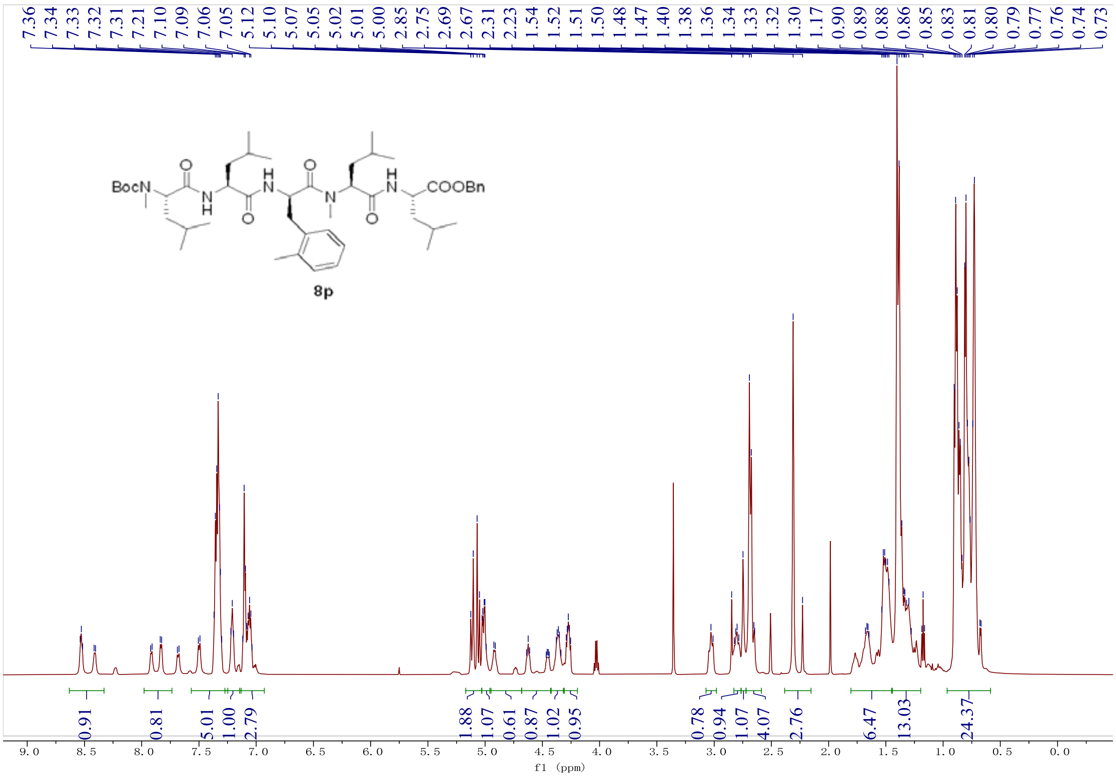

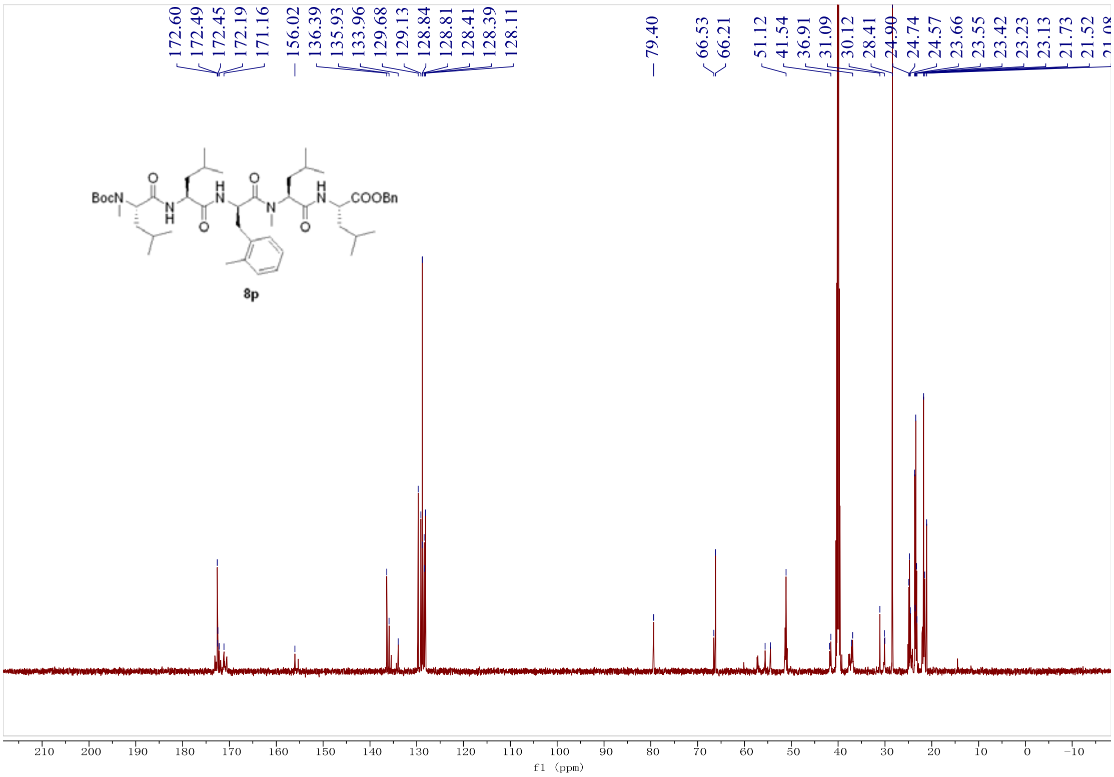

^{1}H NMR (DMSO, 600 MHz) and ^{13}C NMR (DMSO, 151 MHz) for **8q**

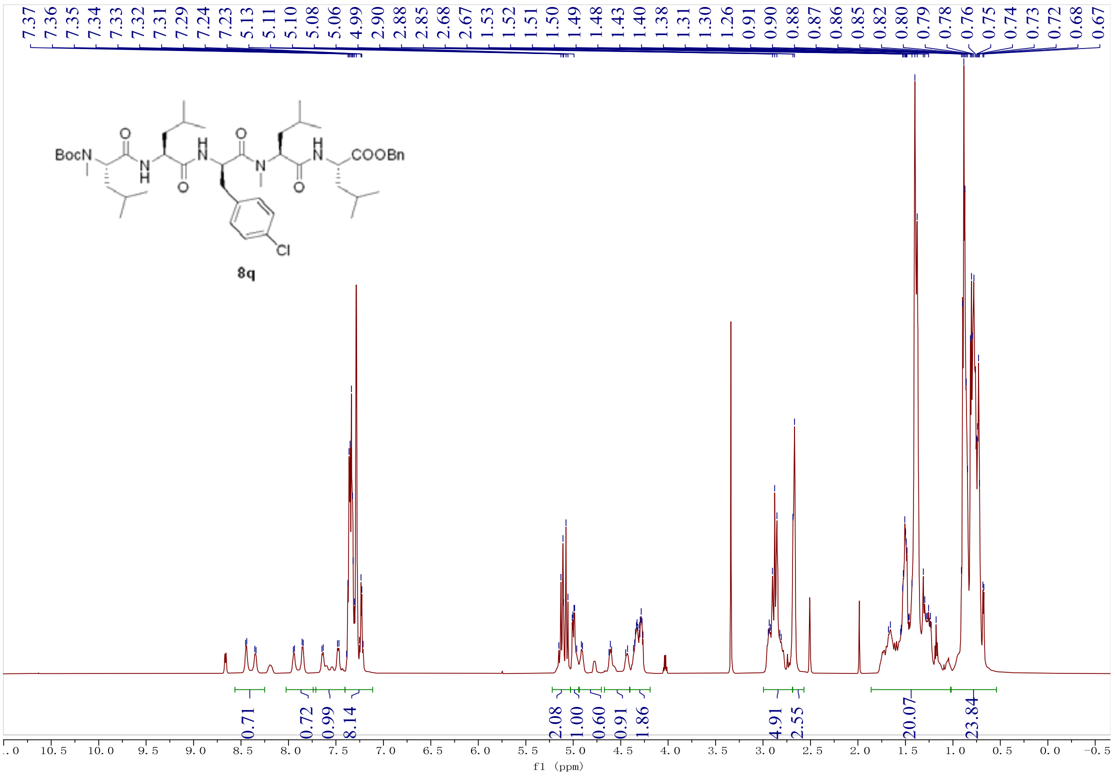

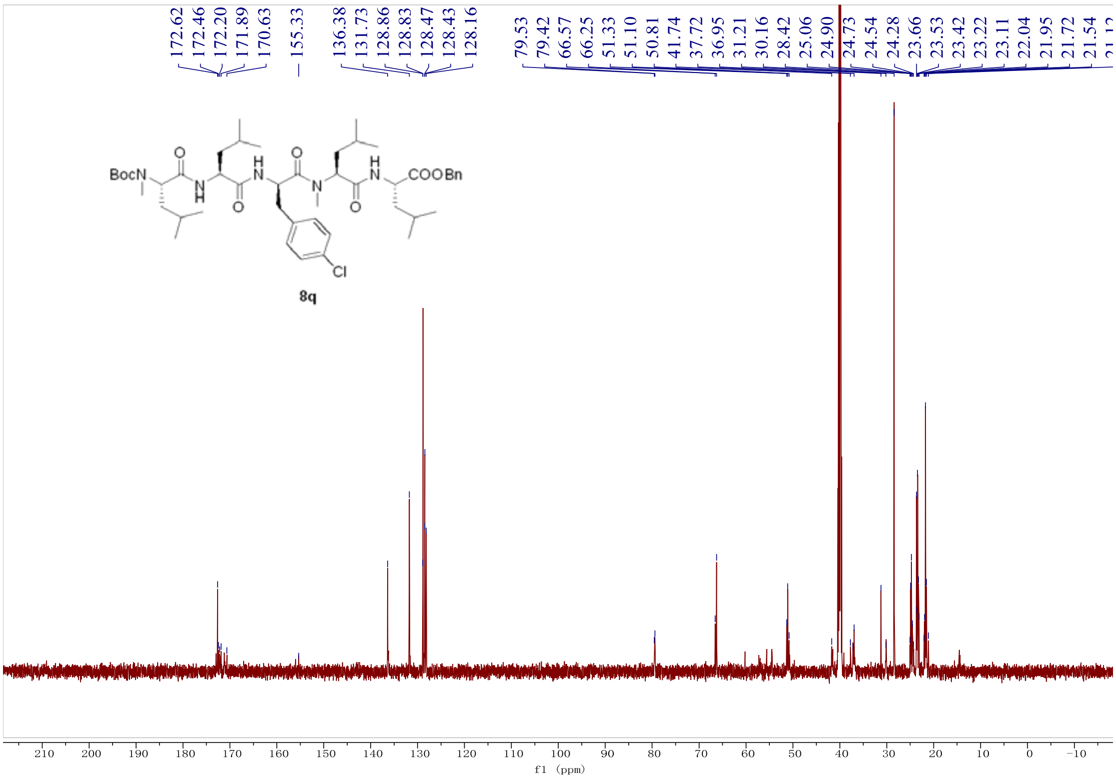

^{1}H NMR (DMSO, 600 MHz) and ^{13}C NMR (DMSO, 151 MHz) for **8r**

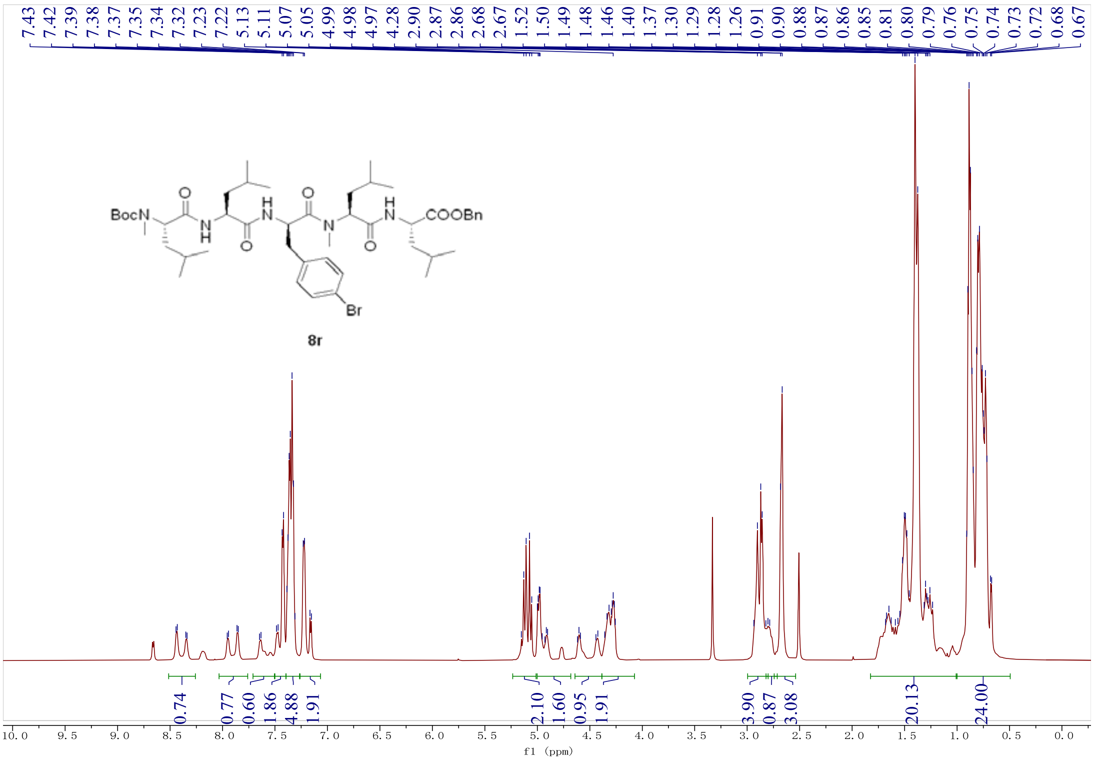

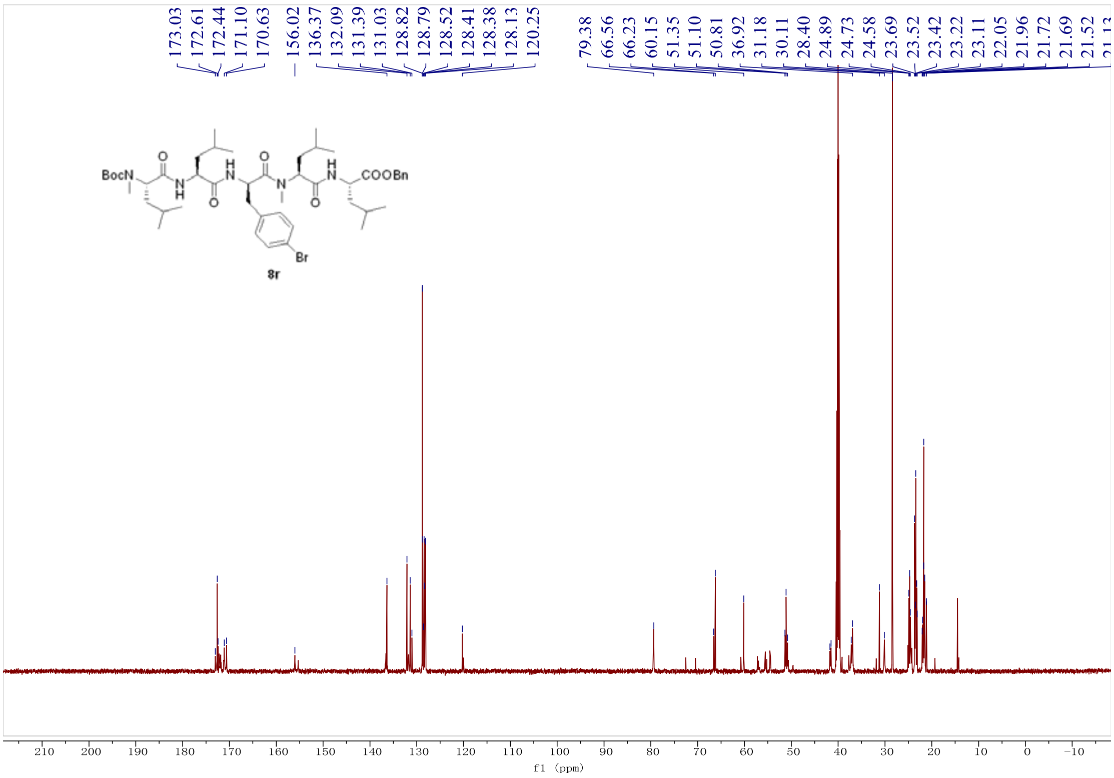

^{1}H NMR (DMSO, 600 MHz) and ^{13}C NMR (DMSO, 151 MHz) for **8s**

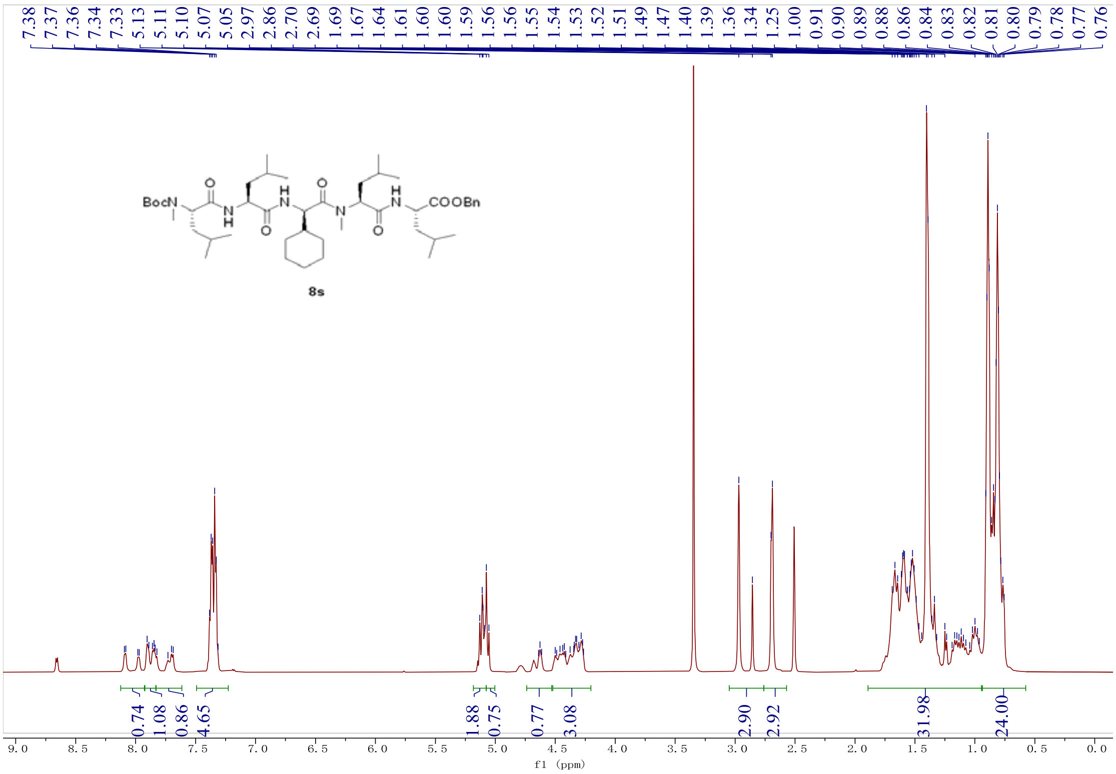

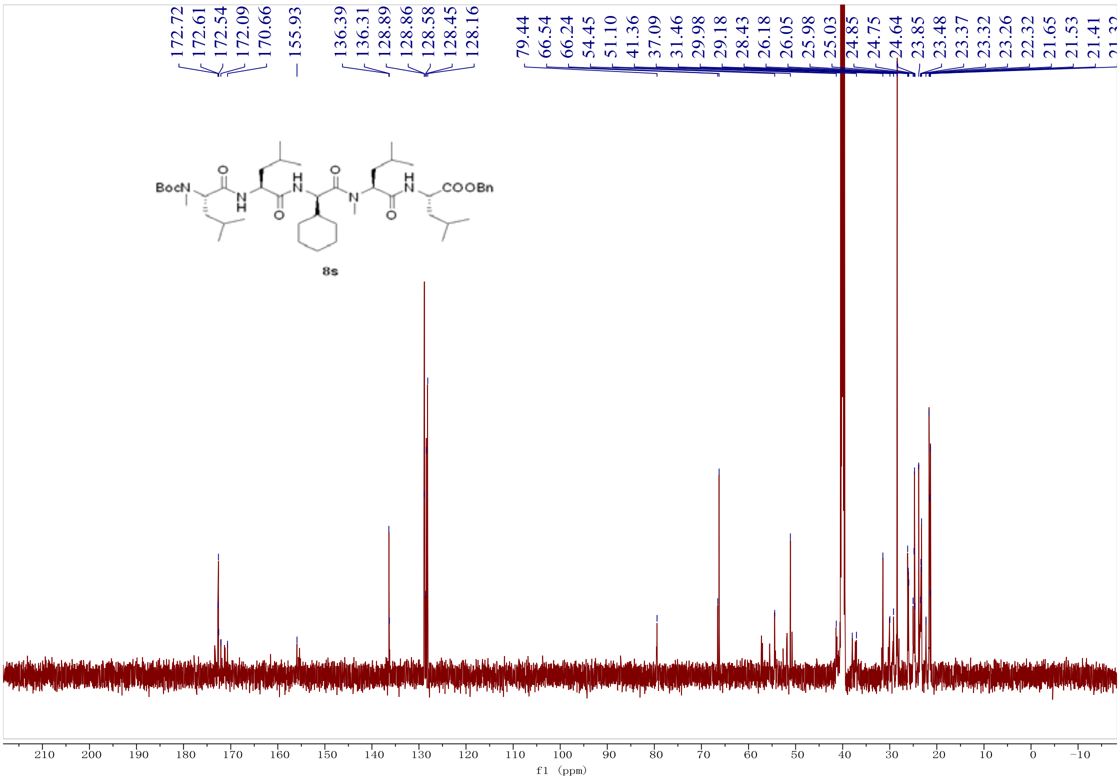

^{1}H NMR (DMSO, 600 MHz) and ^{13}C NMR (DMSO, 151 MHz) for **8t**

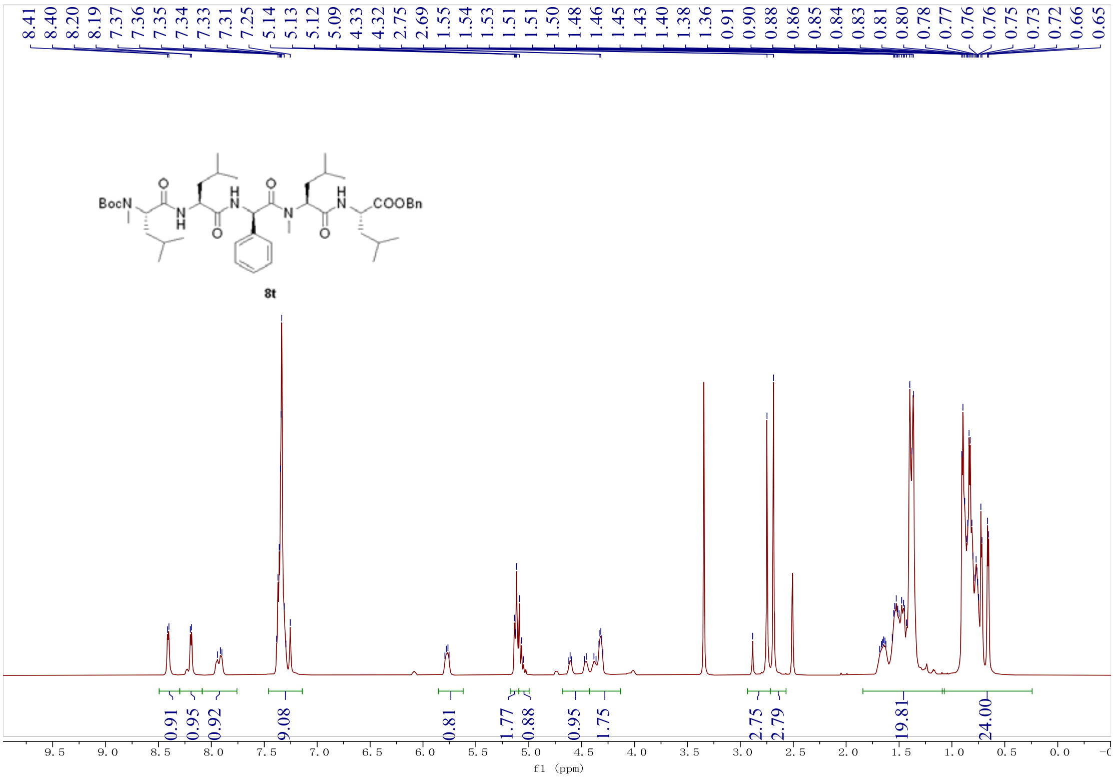

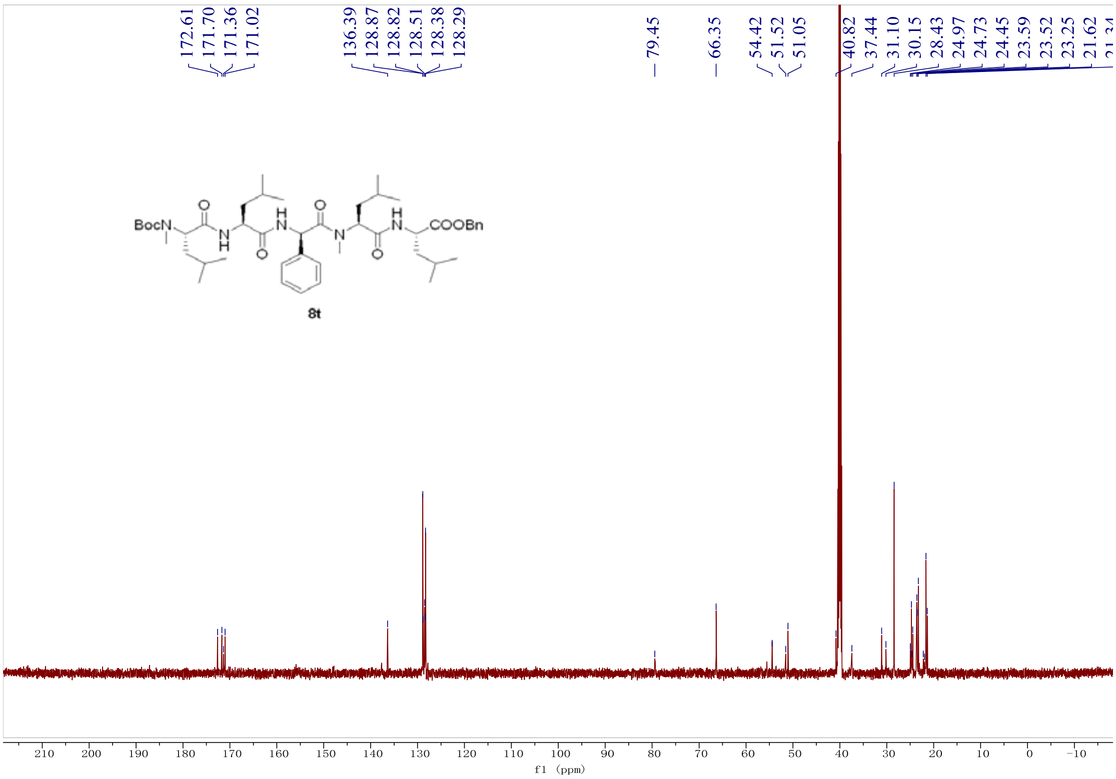

^{1}H NMR (DMSO, 600 MHz) and ^{13}C NMR (DMSO, 151 MHz) for **8u**

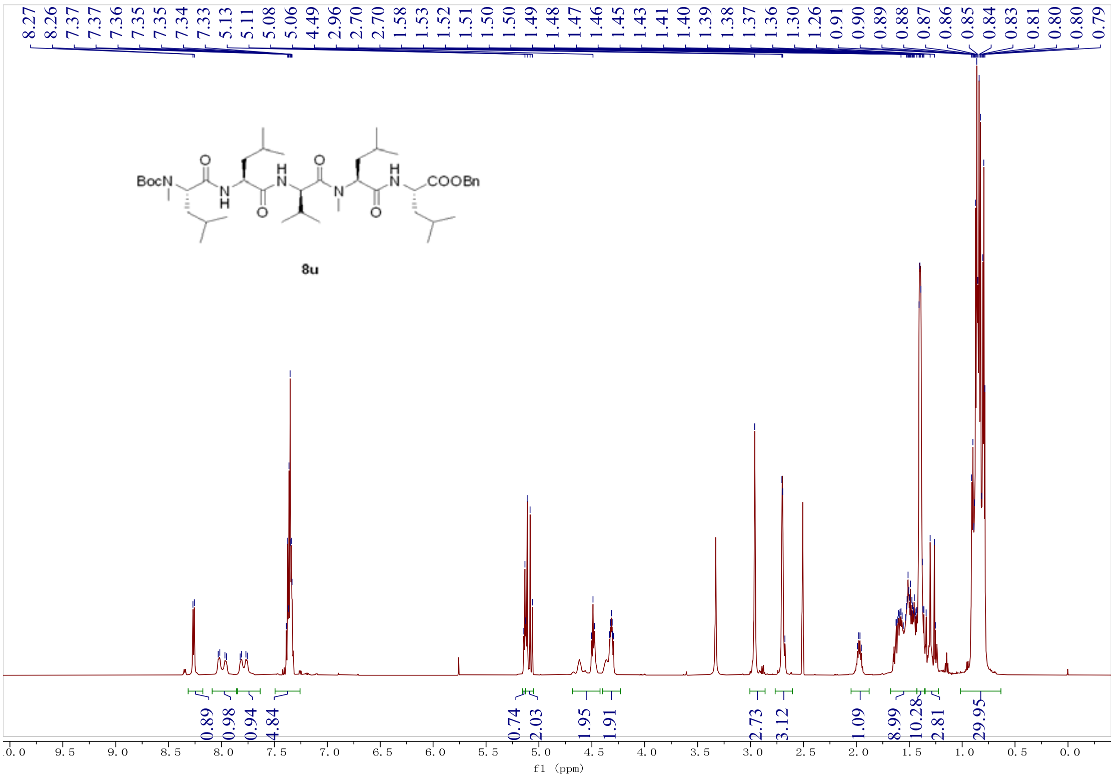

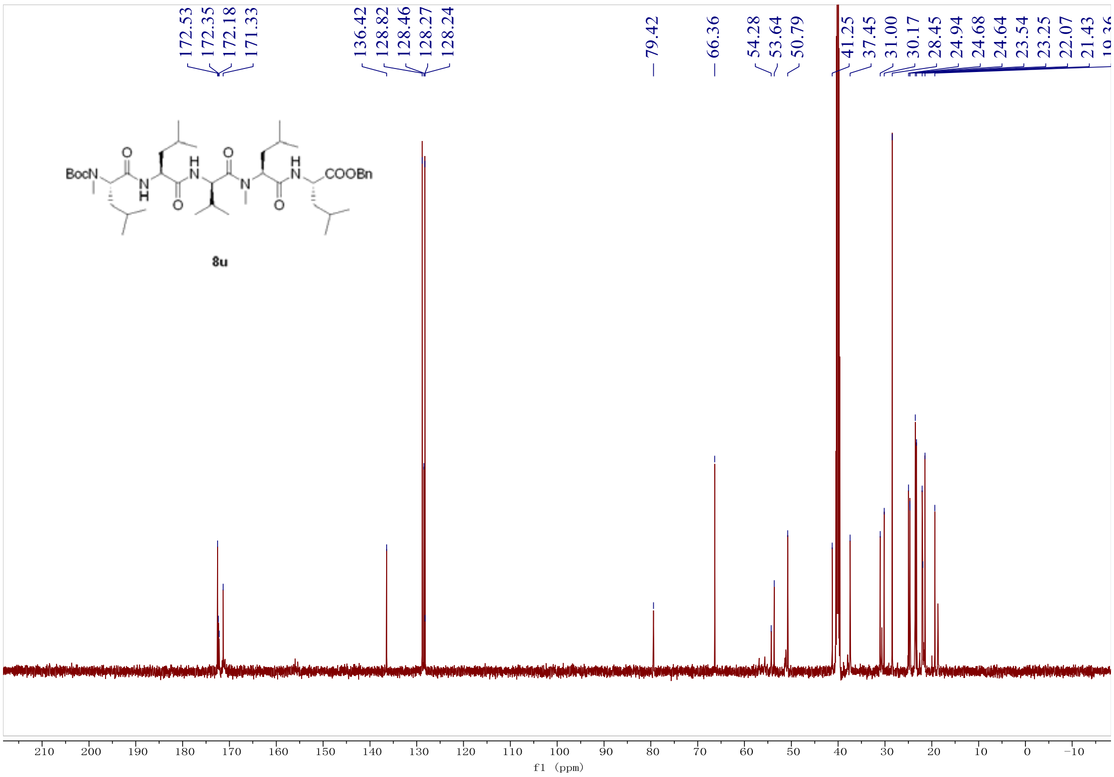

^{1}H NMR (DMSO, 600 MHz) and ^{13}C NMR (DMSO, 151 MHz) for **8v**

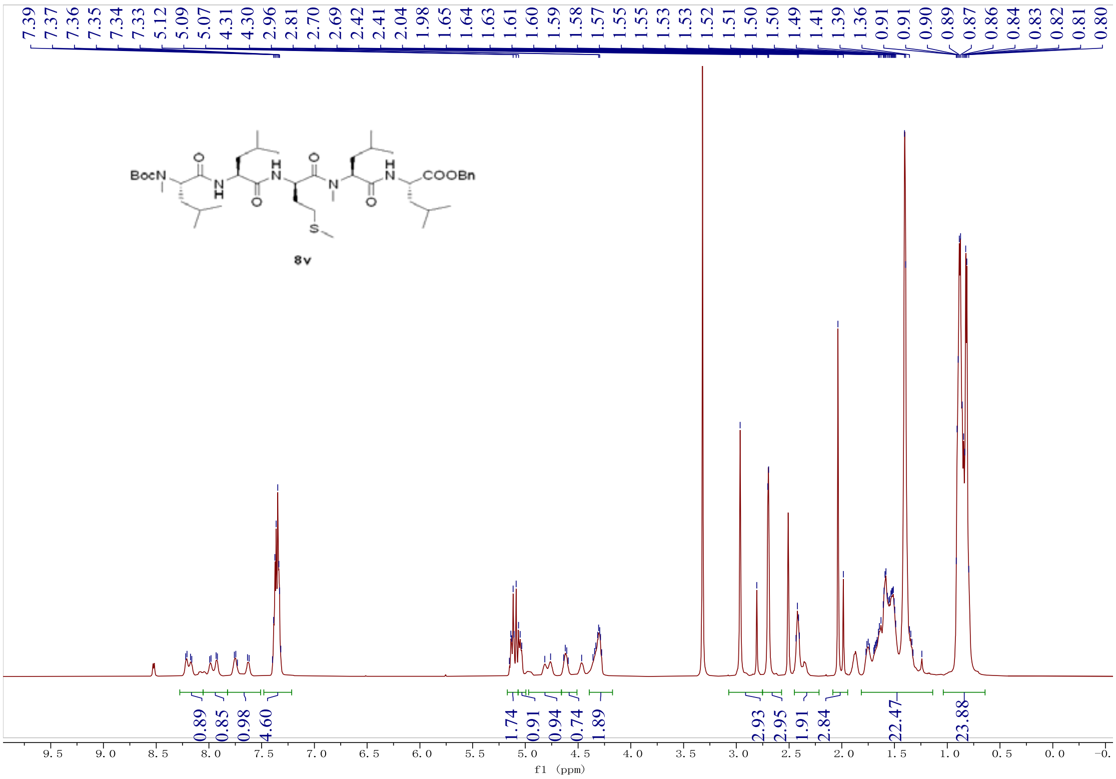

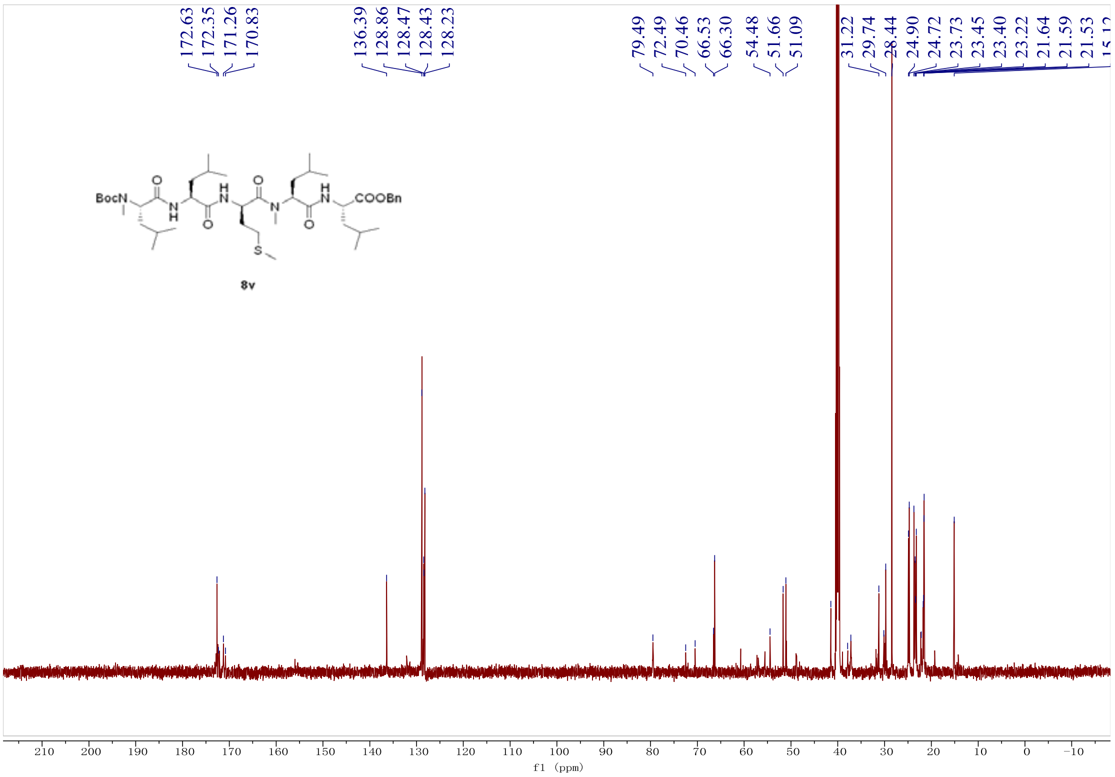

^{1}H NMR (DMSO, 600 MHz) and ^{13}C NMR (DMSO, 151 MHz) for **8w**

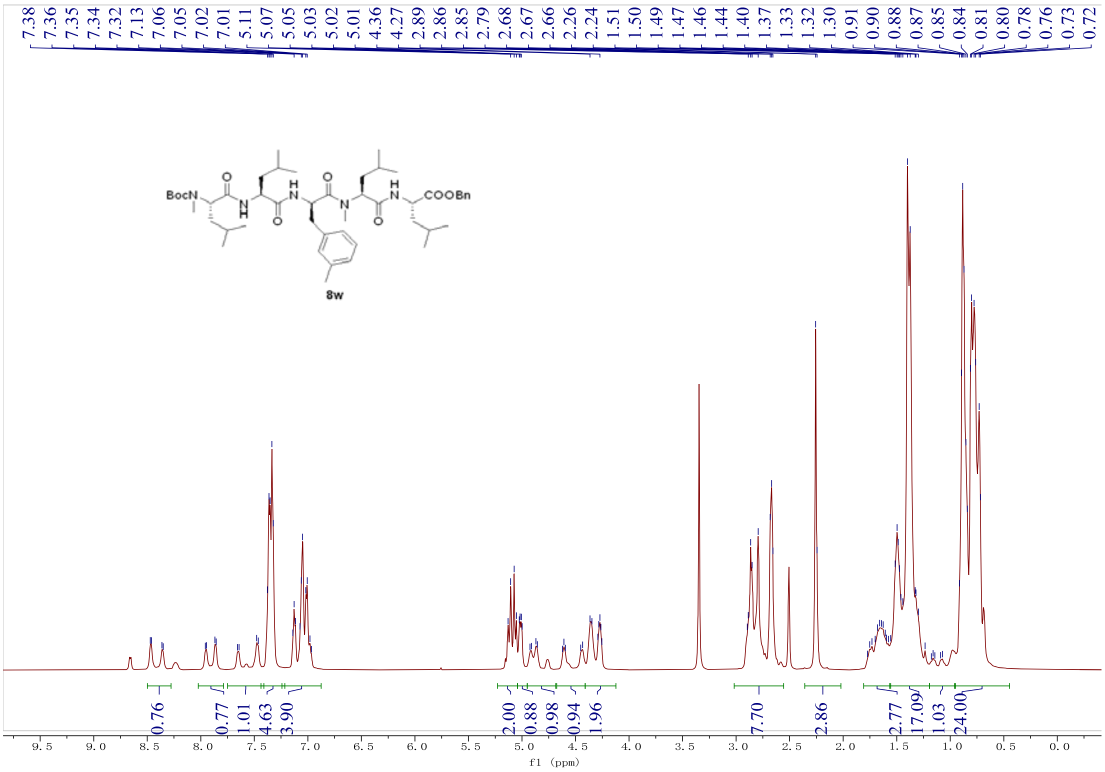

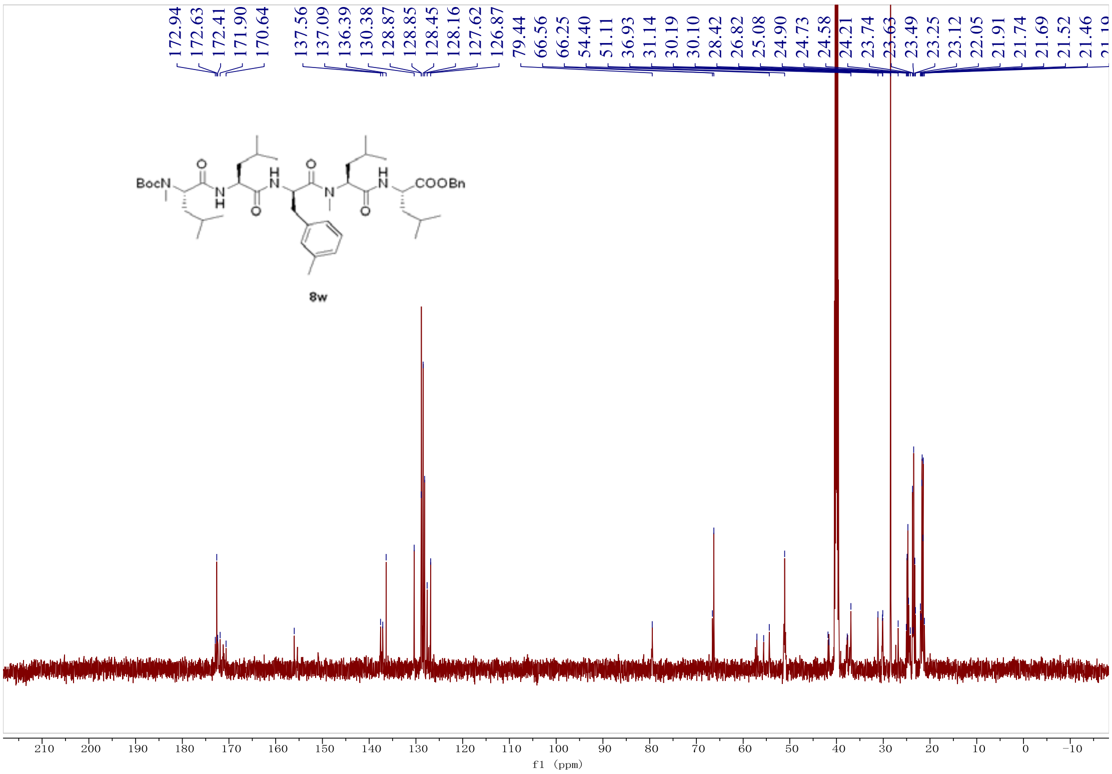

^{1}H NMR (DMSO, 600 MHz) and ^{13}C NMR (DMSO, 151 MHz) for **8x**

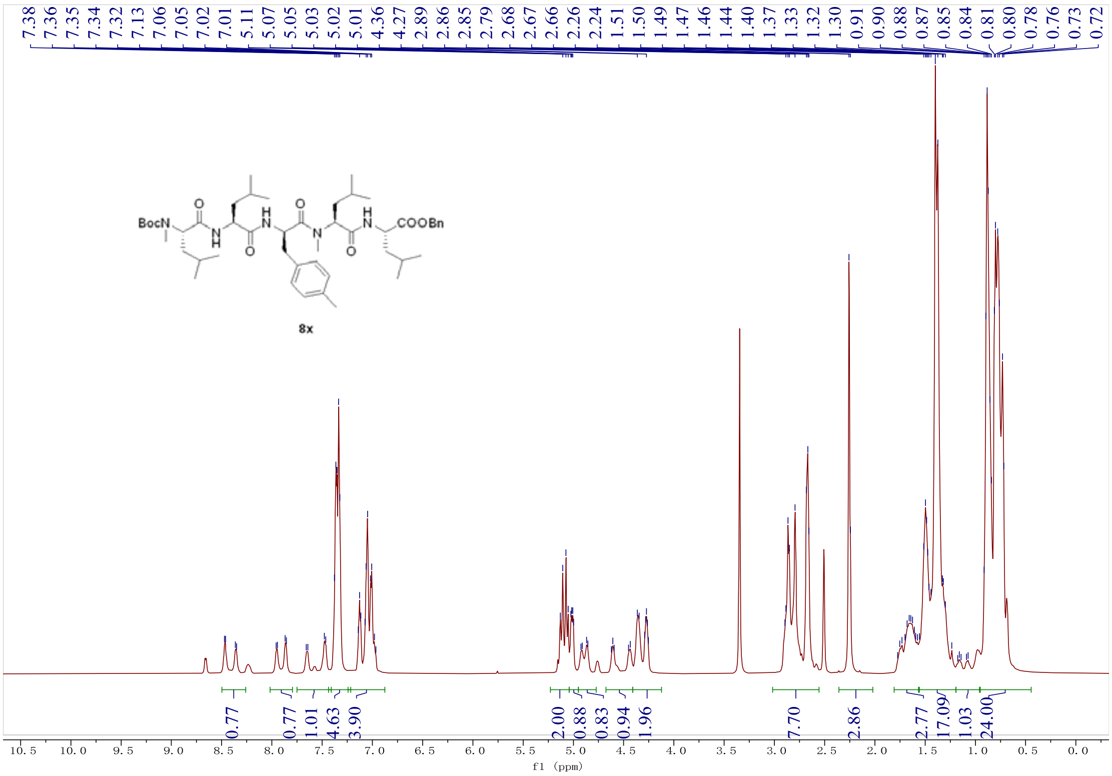

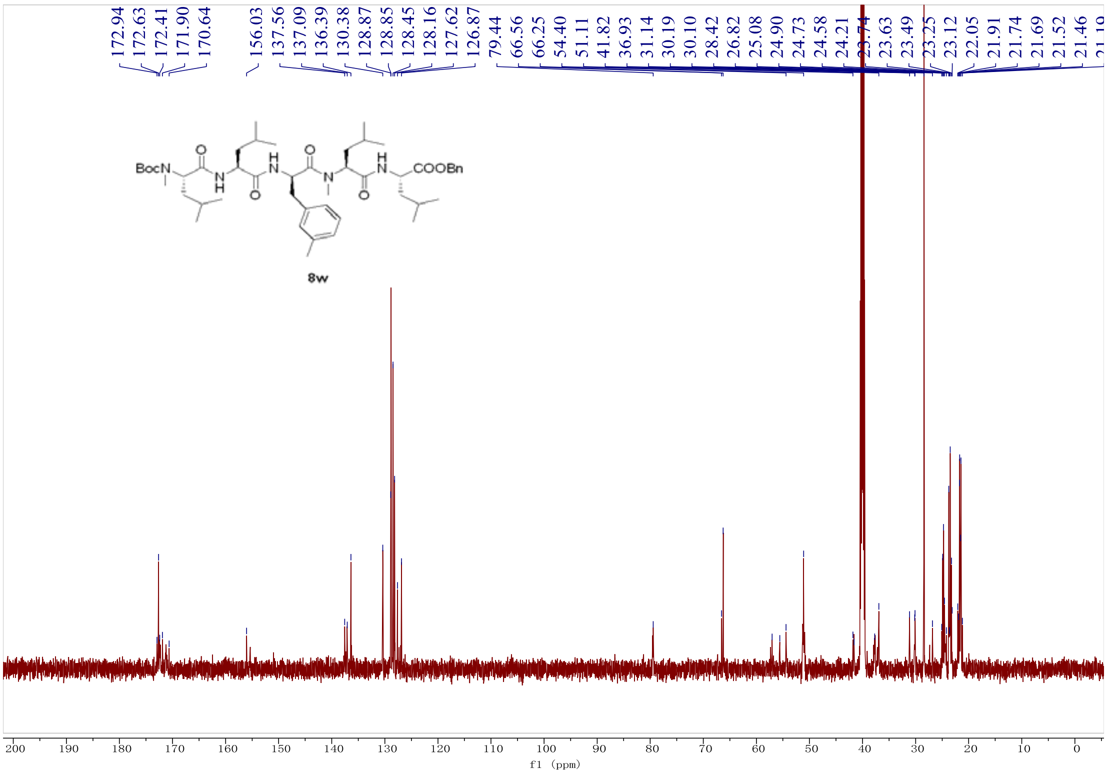

^{1}H NMR (DMSO, 600 MHz) and ^{13}C NMR (DMSO, 151 MHz) for **Gala01**

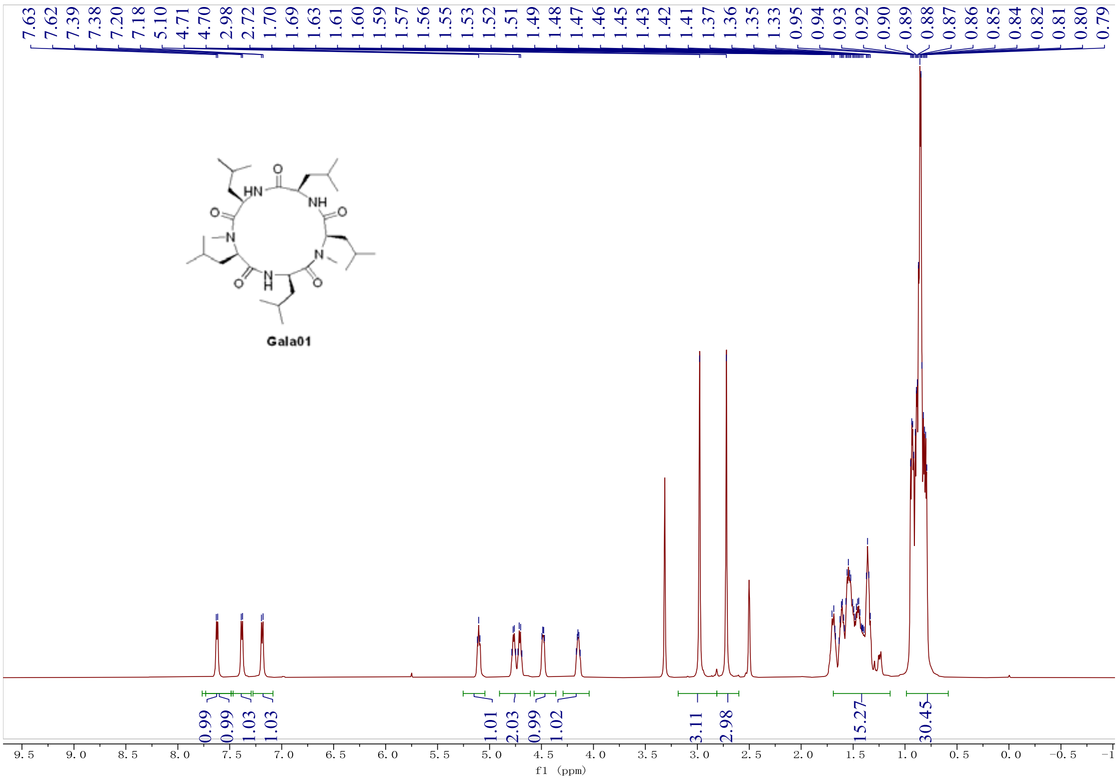

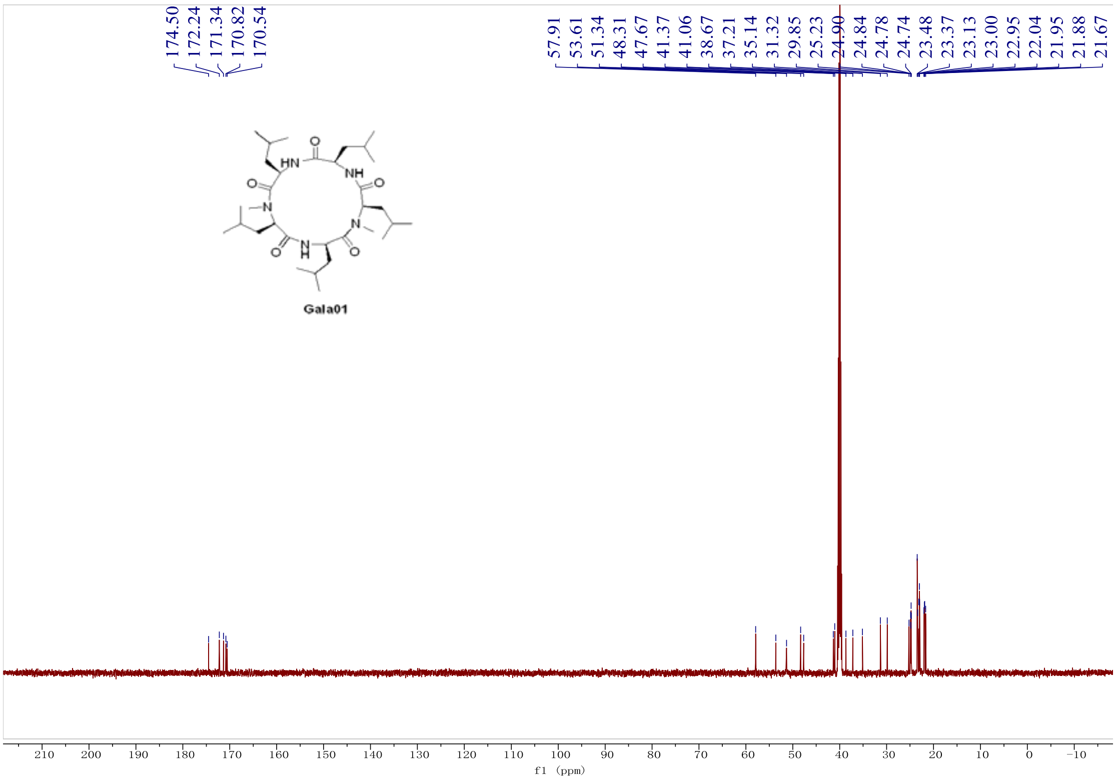

^{1}H NMR (DMSO, 600 MHz) and ^{13}C NMR (DMSO, 151 MHz) for **Gala02**

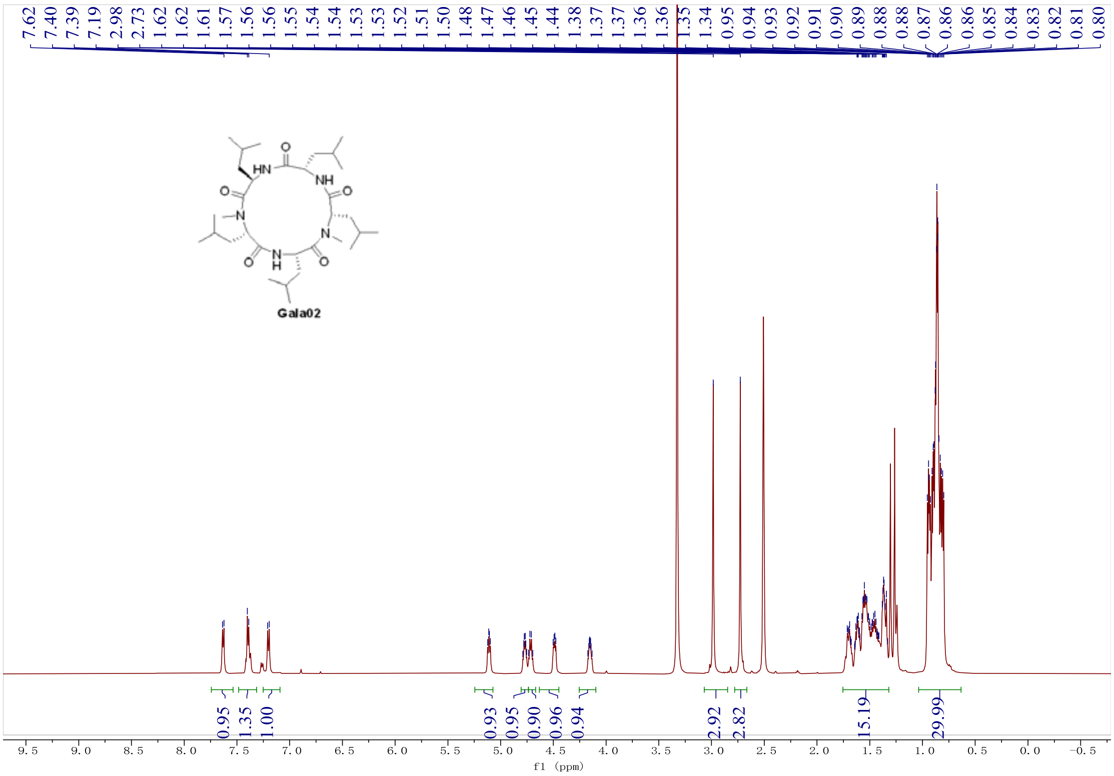

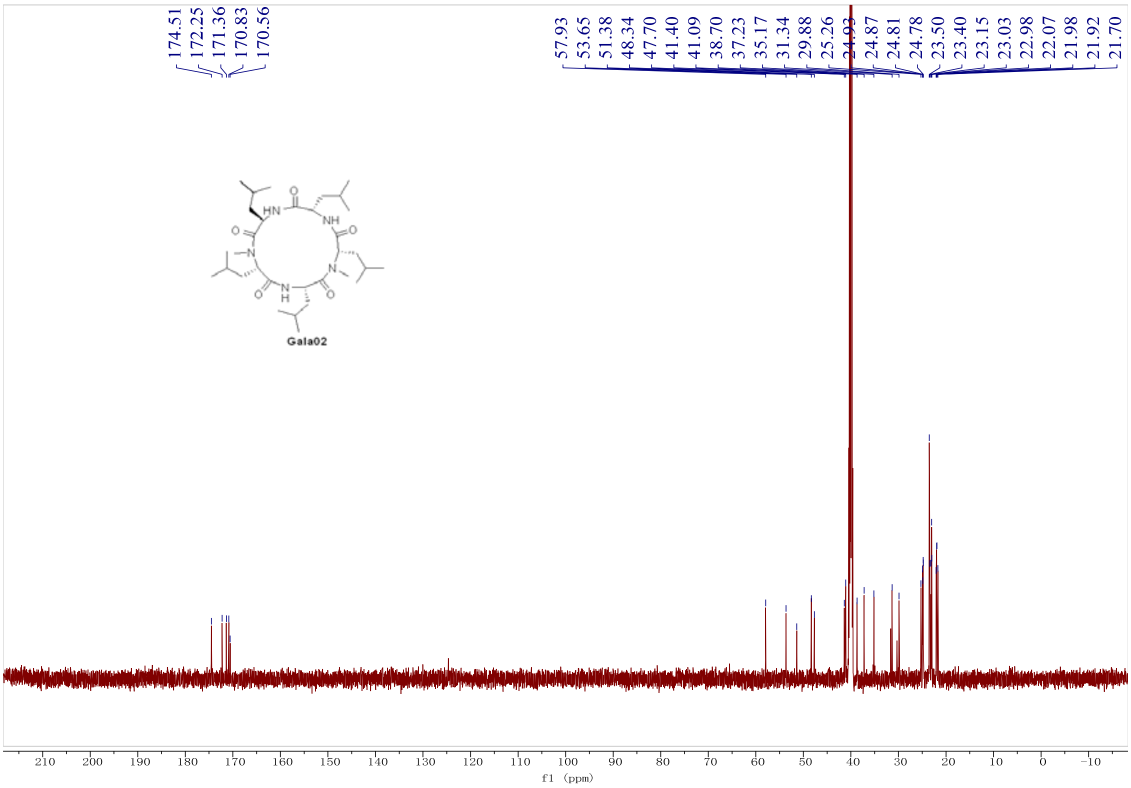

^{1}H NMR (DMSO, 600 MHz) and ^{13}C NMR (DMSO, 151 MHz) for **Gala03**

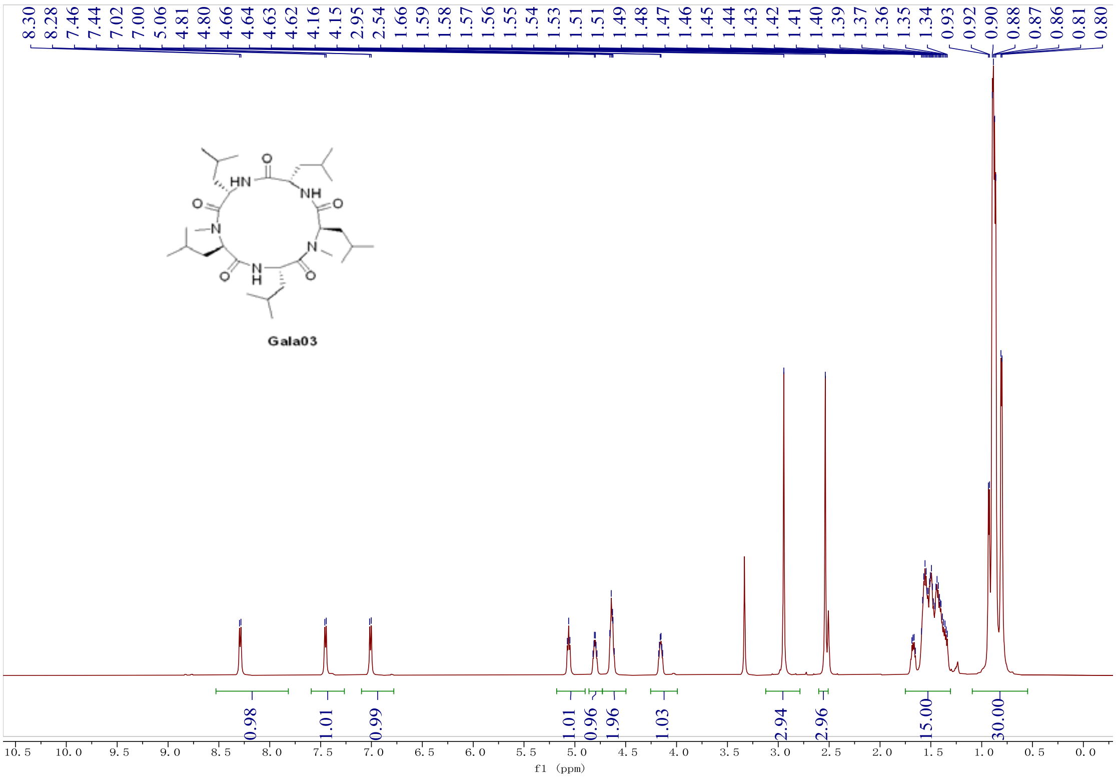

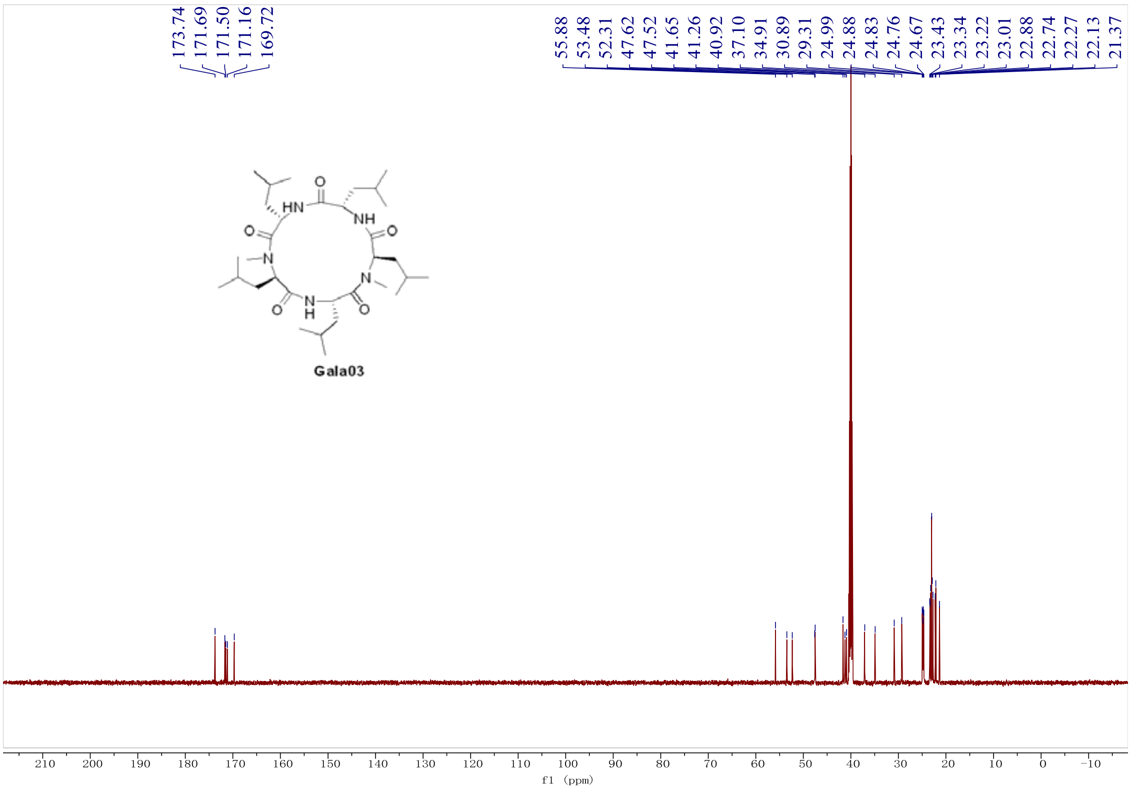

^{1}H NMR (DMSO, 600 MHz) and ^{13}C NMR (DMSO, 151 MHz) for **Gala04**

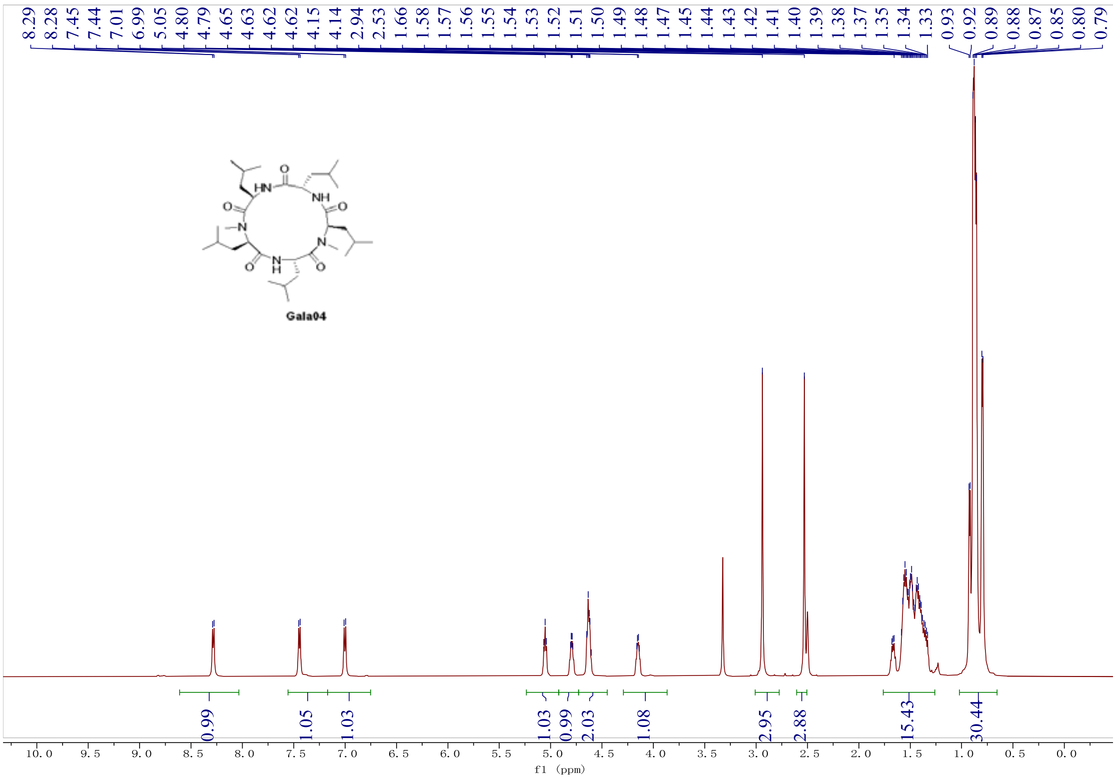

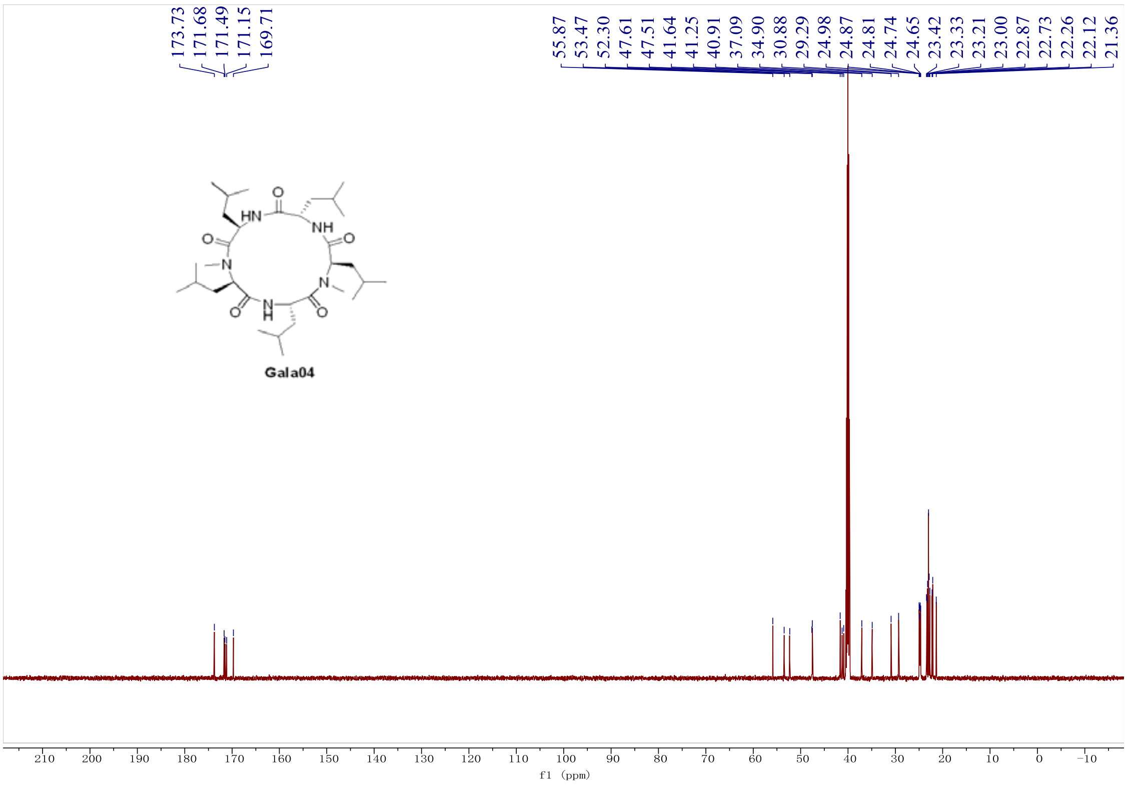

^{1}H NMR (DMSO, 600 MHz) and ^{13}C NMR (DMSO, 151 MHz) for **Gala05**

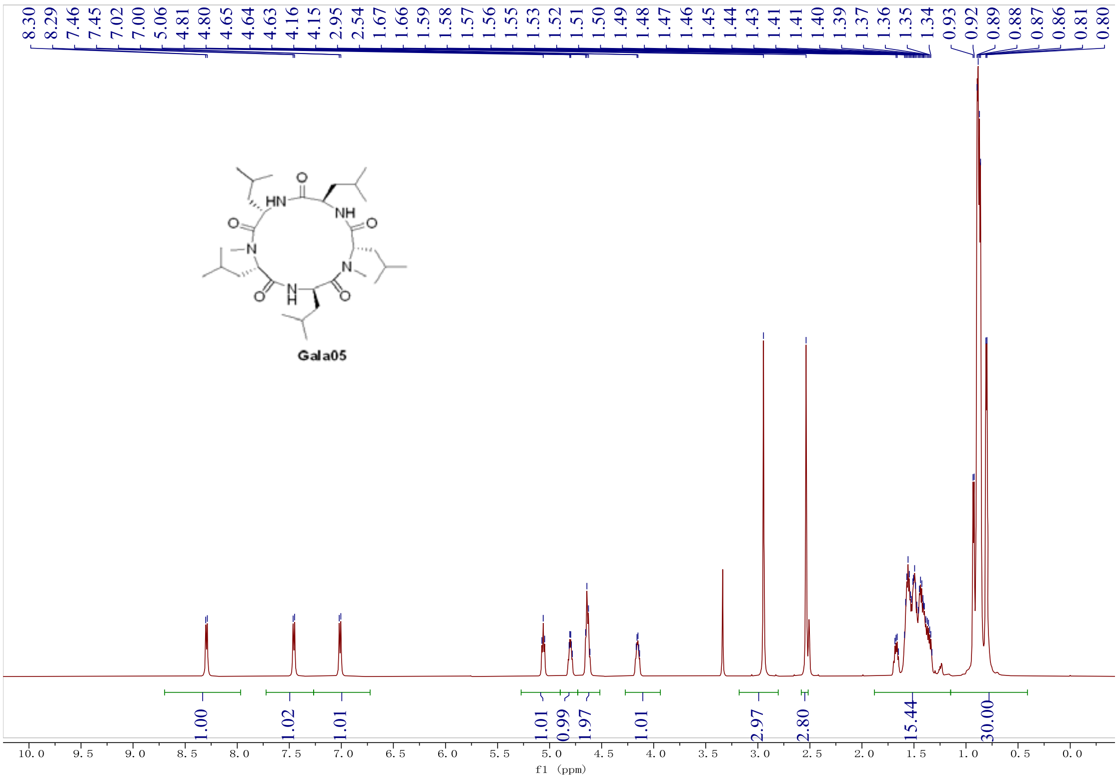

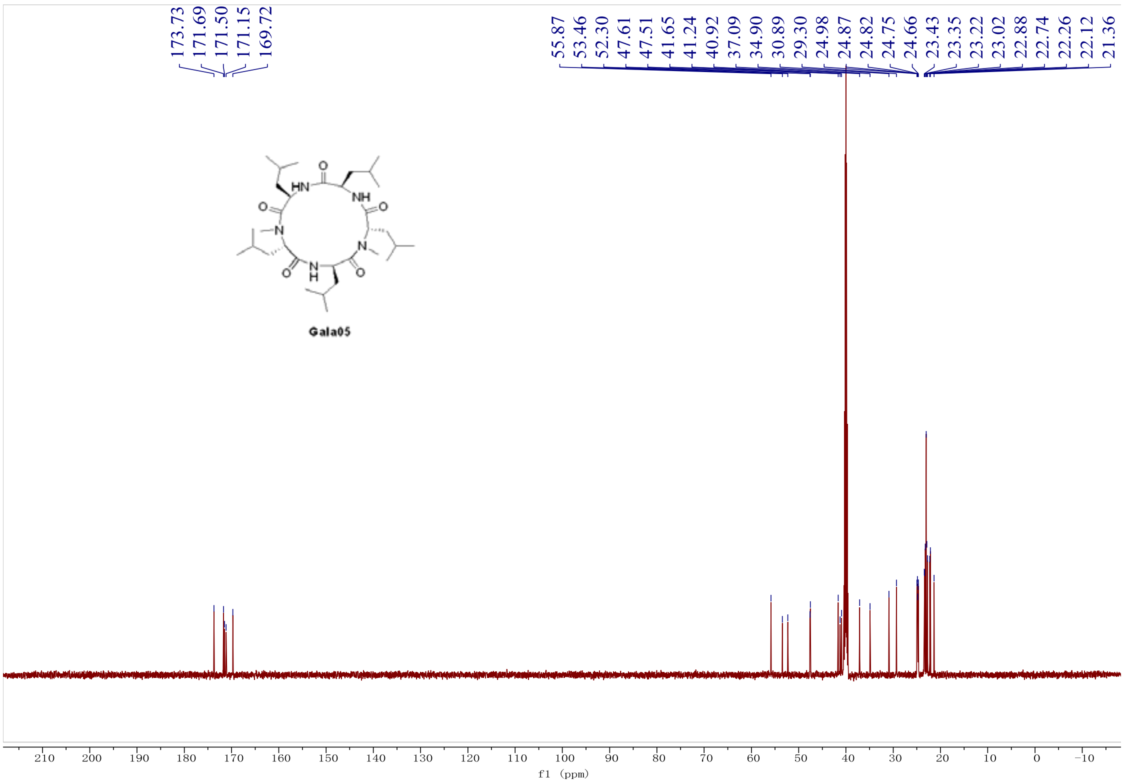

^{1}H NMR (DMSO, 600 MHz) and ^{13}C NMR (DMSO, 151 MHz) for **Gala06**

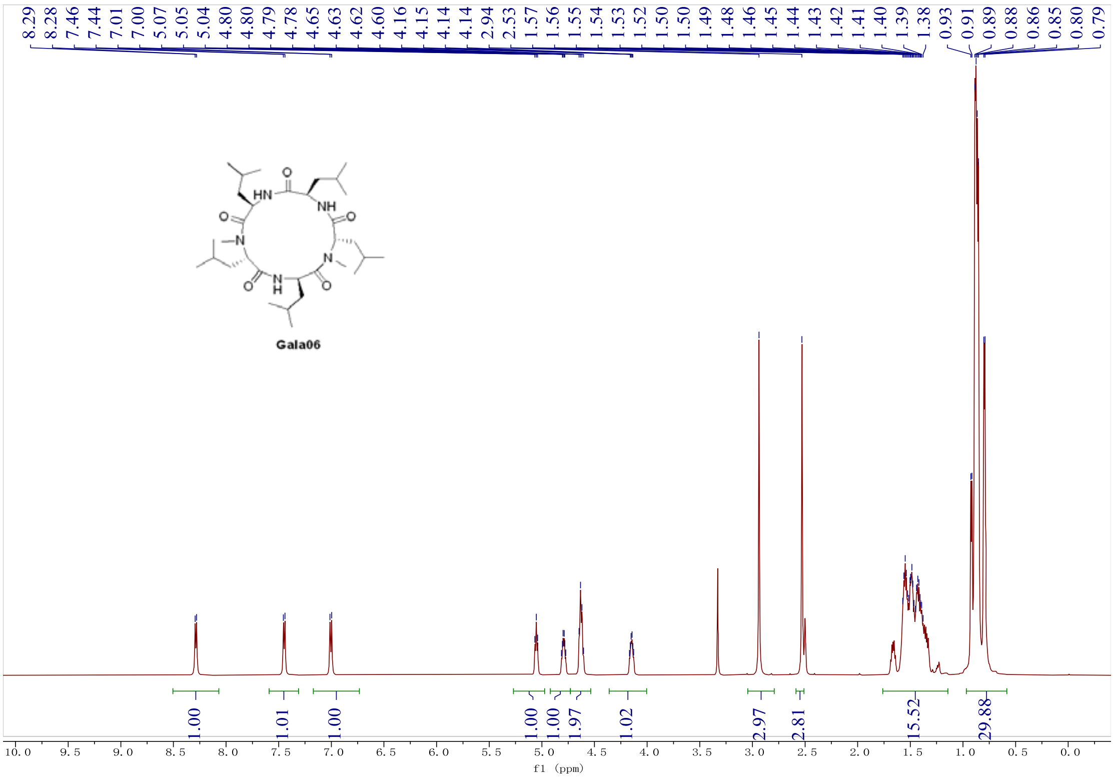

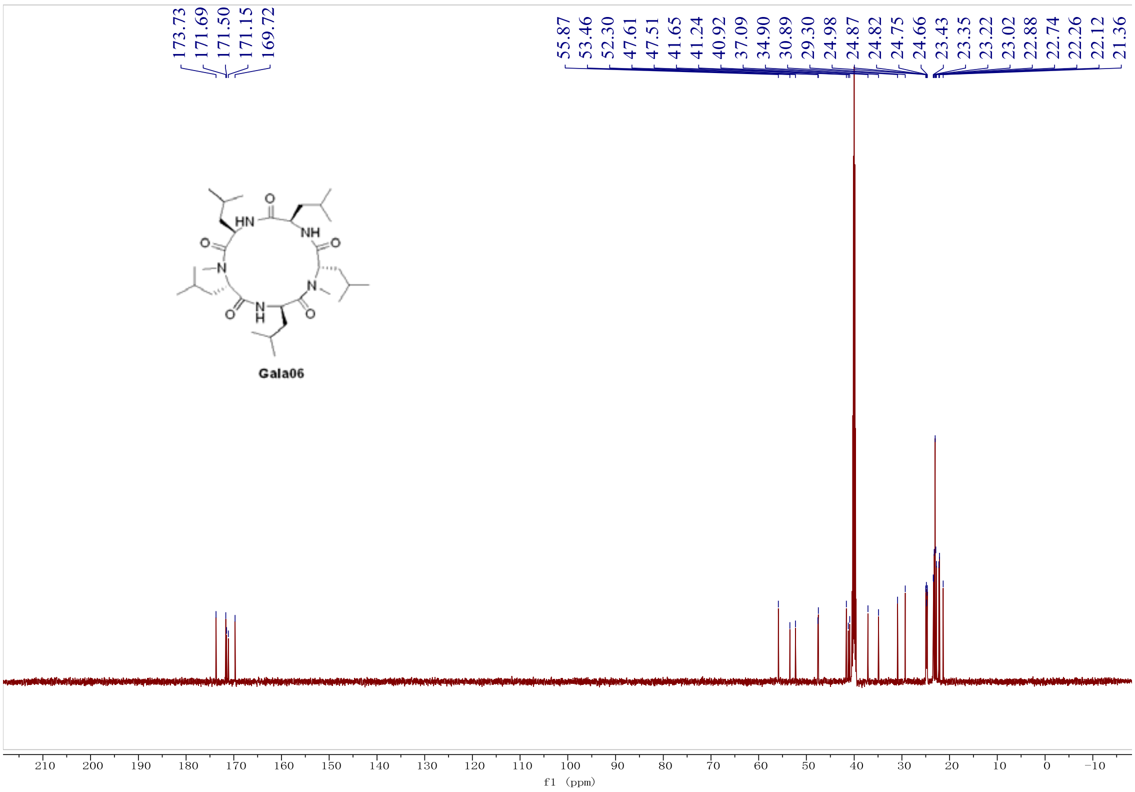

^{1}H NMR (DMSO, 600 MHz) and ^{13}C NMR (DMSO, 151 MHz) for **Gala07**

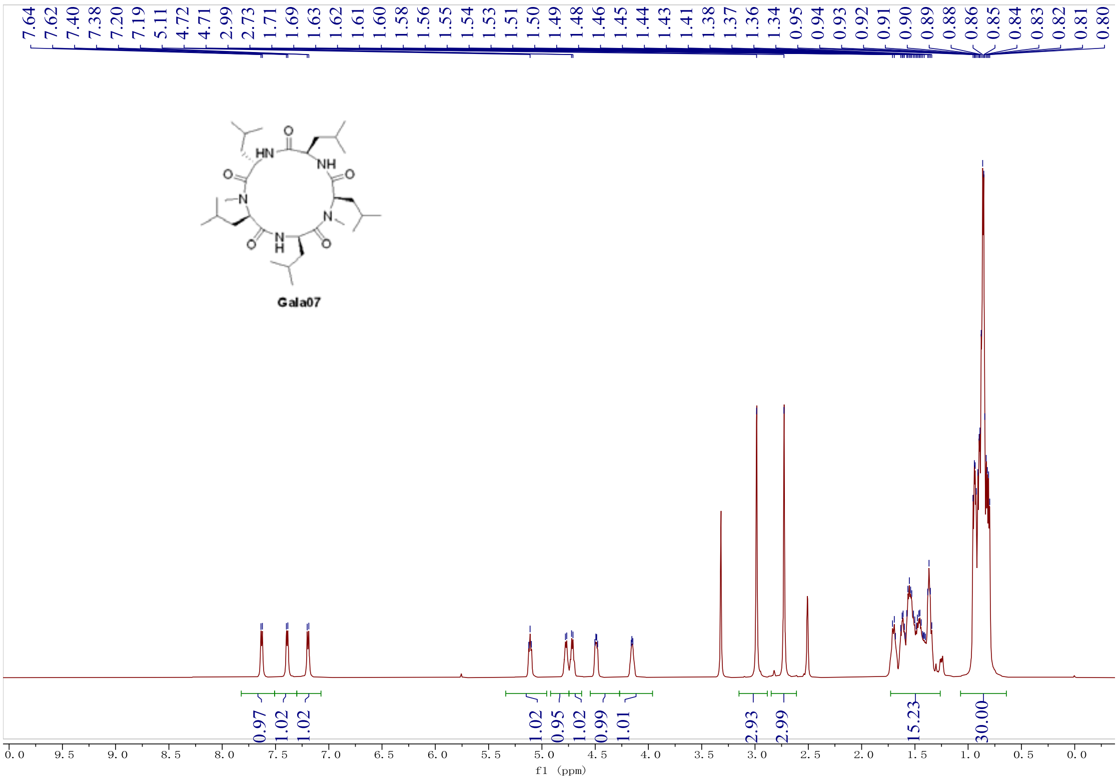

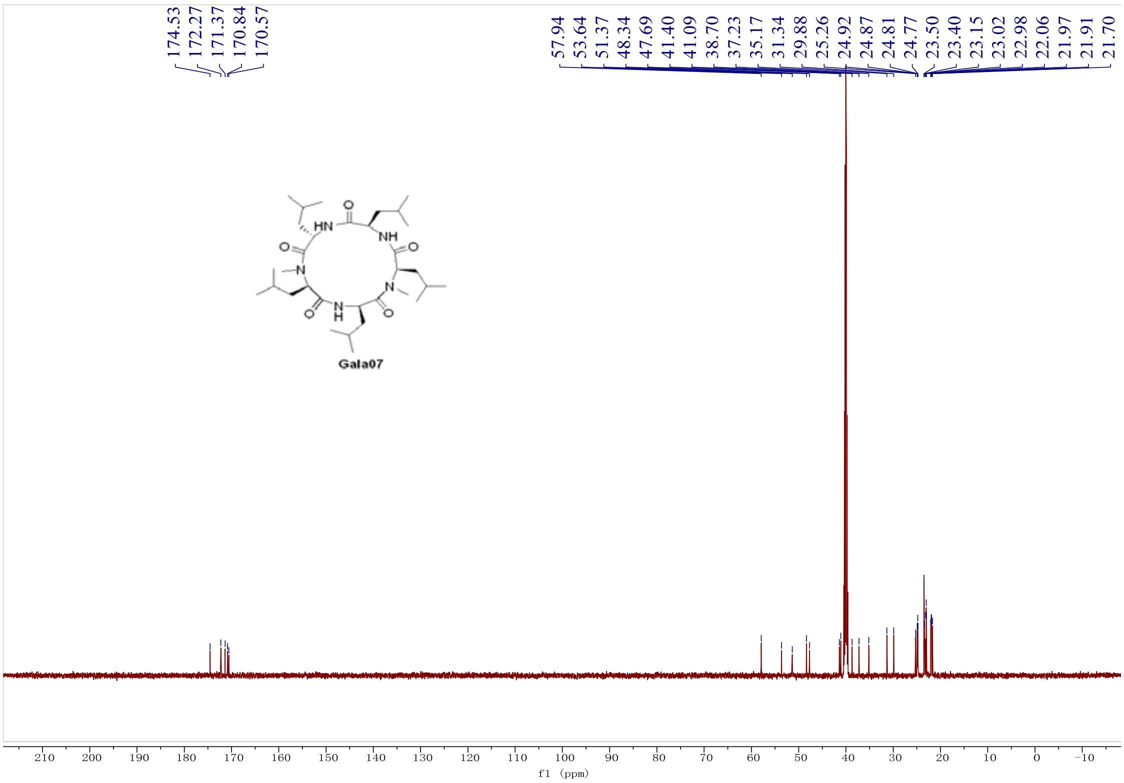

^{1}H NMR (DMSO, 600 MHz) and ^{13}C NMR (DMSO, 151 MHz) for **Gala08**

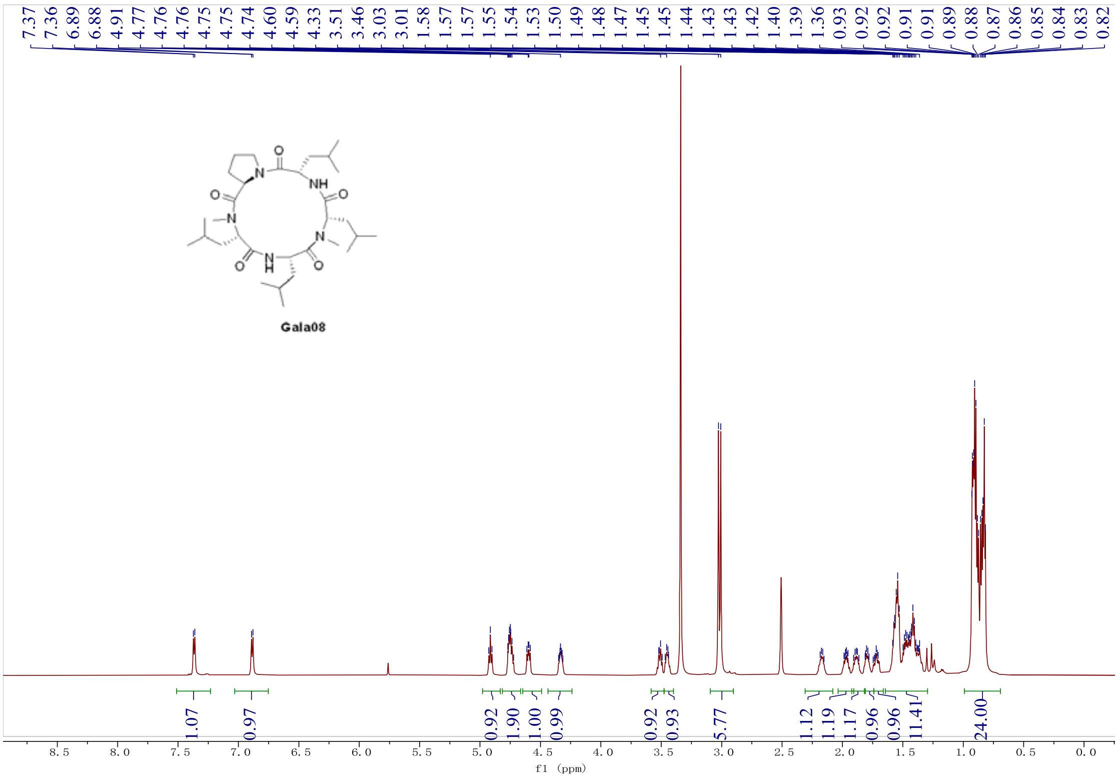

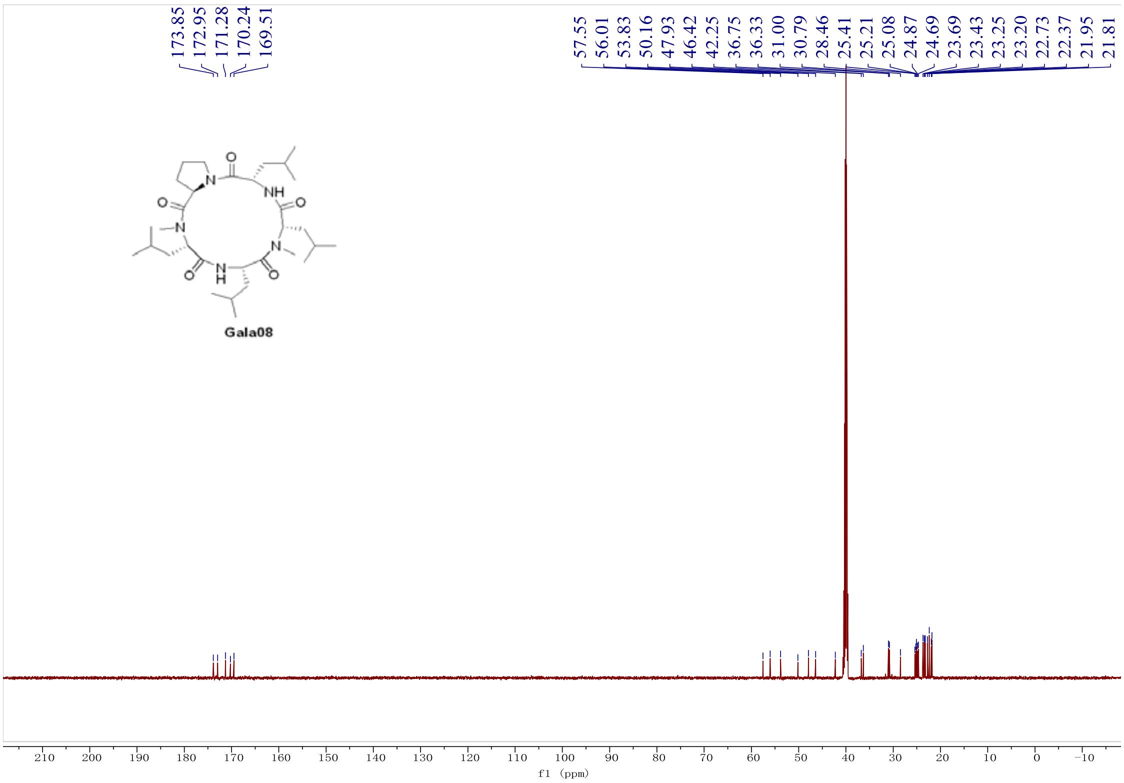

^{1}H NMR (DMSO, 600 MHz) and ^{13}C NMR (DMSO, 151 MHz) for **Gala09**

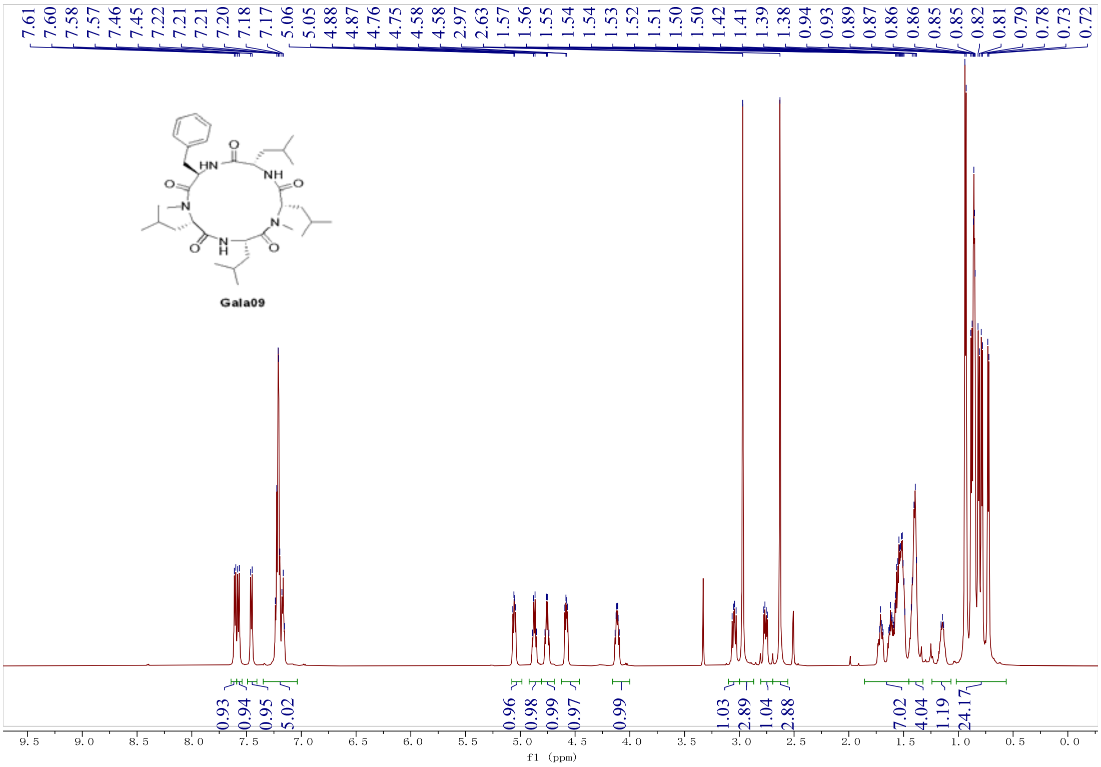

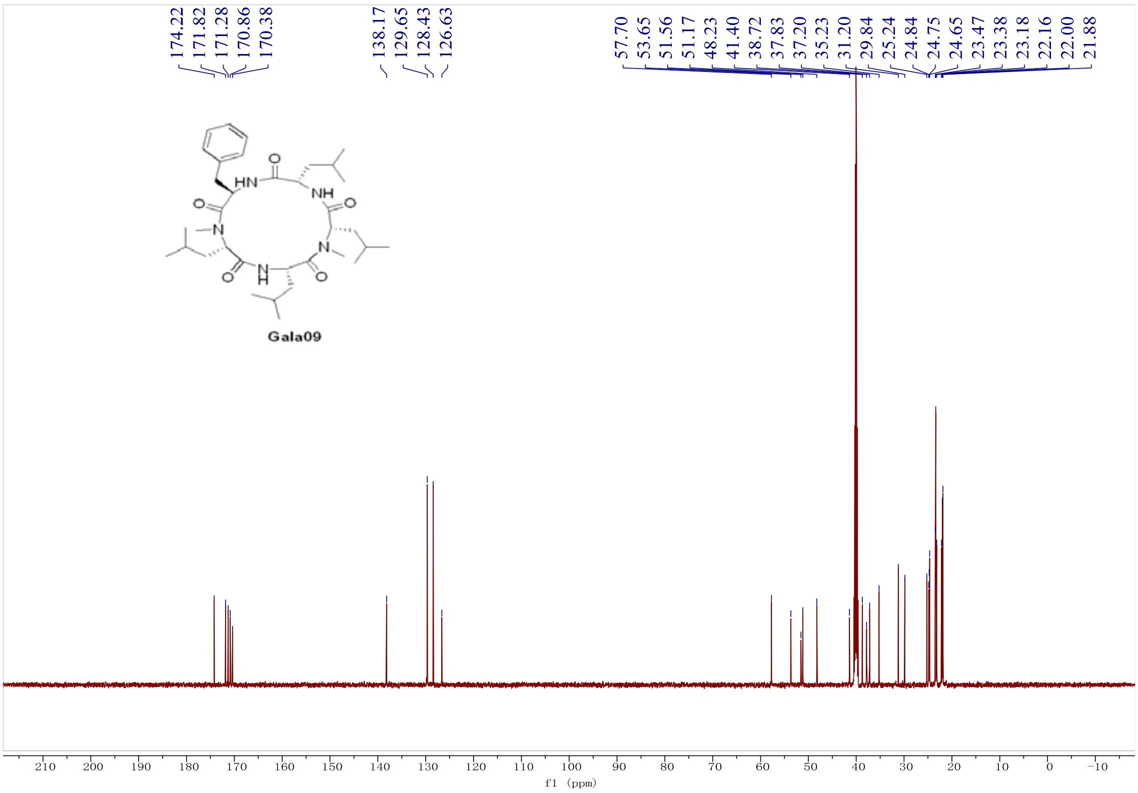

^{1}H NMR (DMSO, 600 MHz) and ^{13}C NMR (DMSO, 151 MHz) for **Gala10**

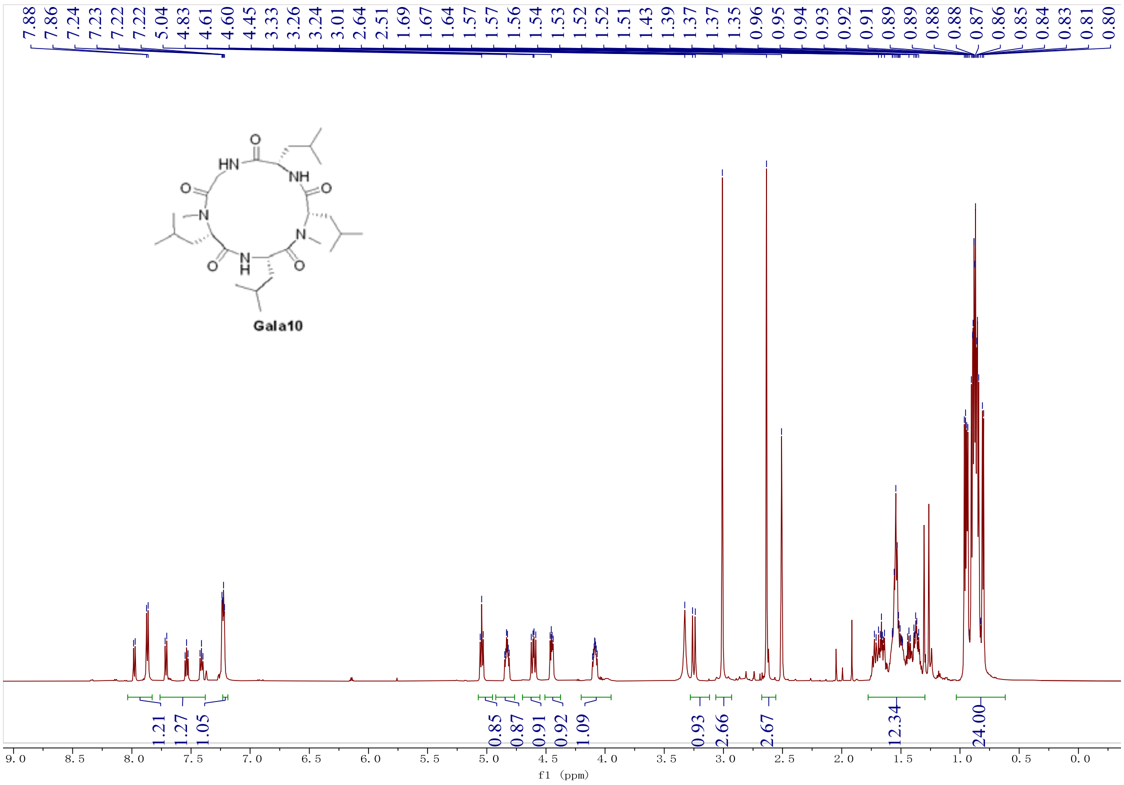

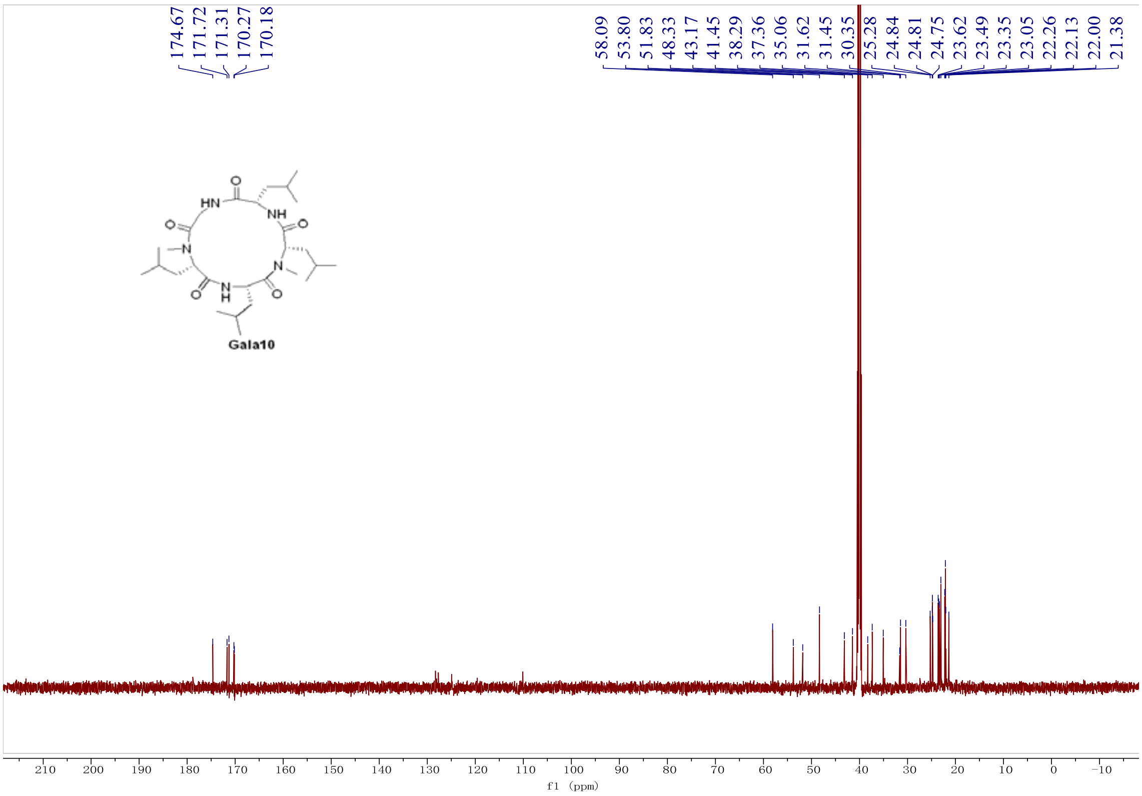

^{1}H NMR (DMSO, 600 MHz) and ^{13}C NMR (DMSO, 151 MHz) for **Gala11**

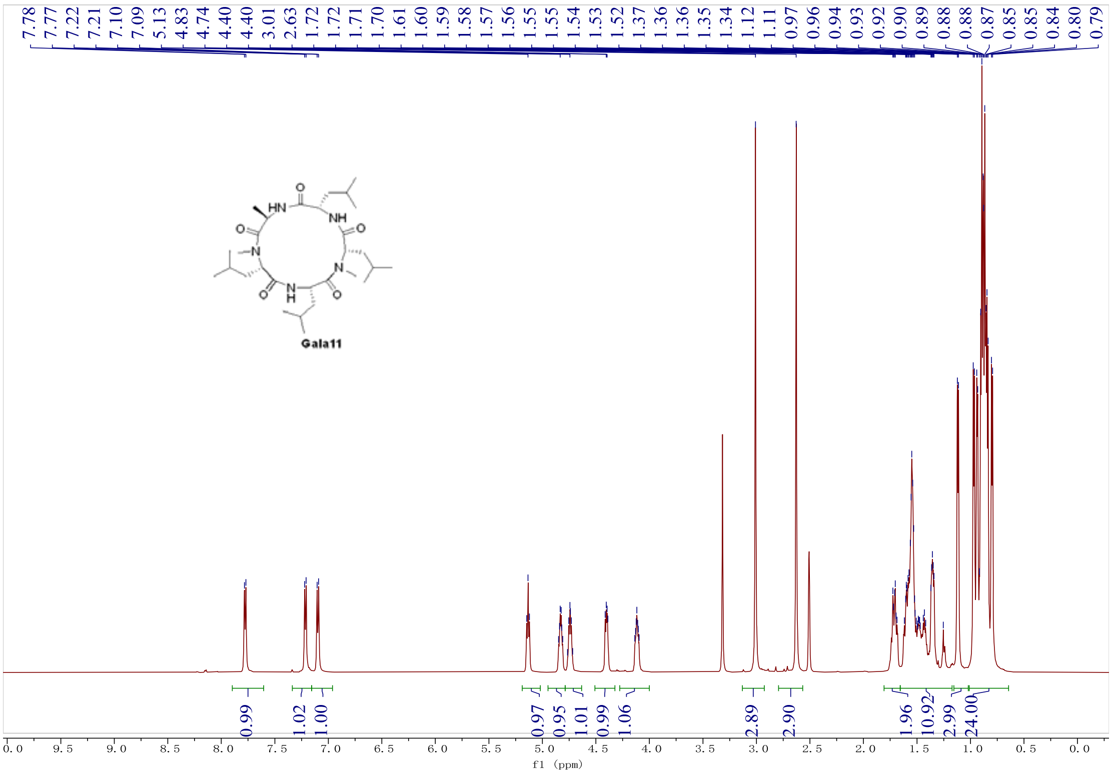

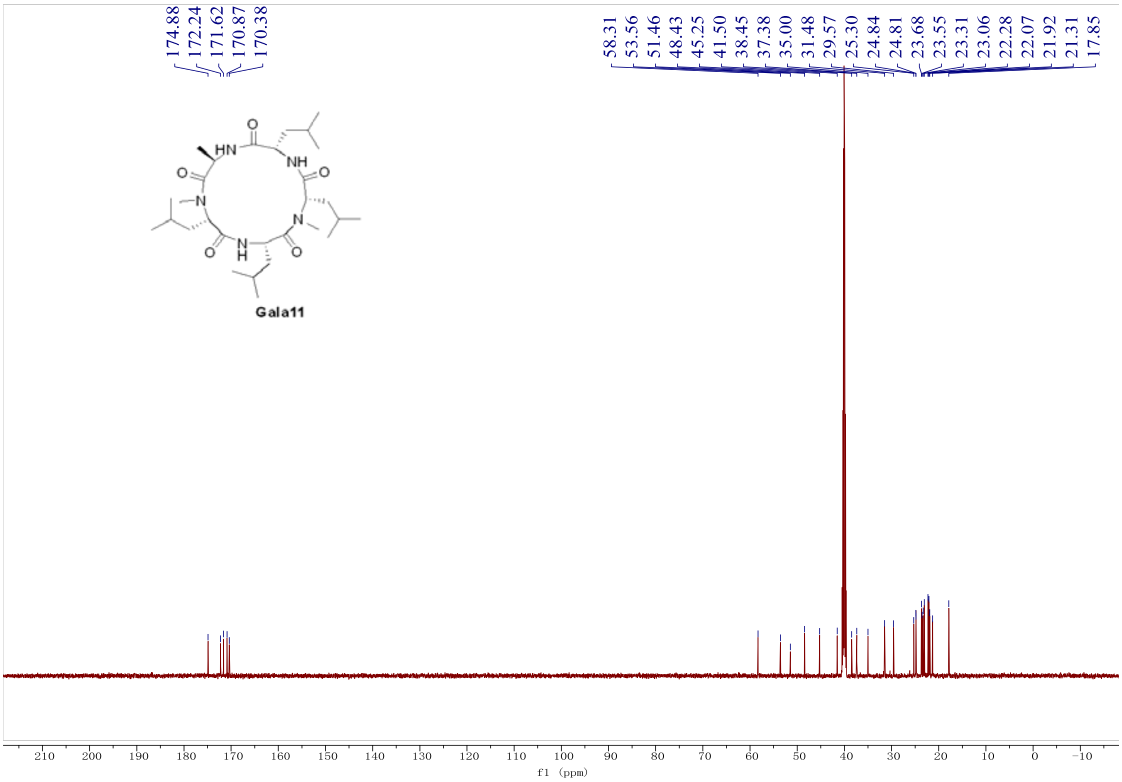

^{1}H NMR (DMSO, 600 MHz) and ^{13}C NMR (DMSO, 151 MHz) for **Gala12**

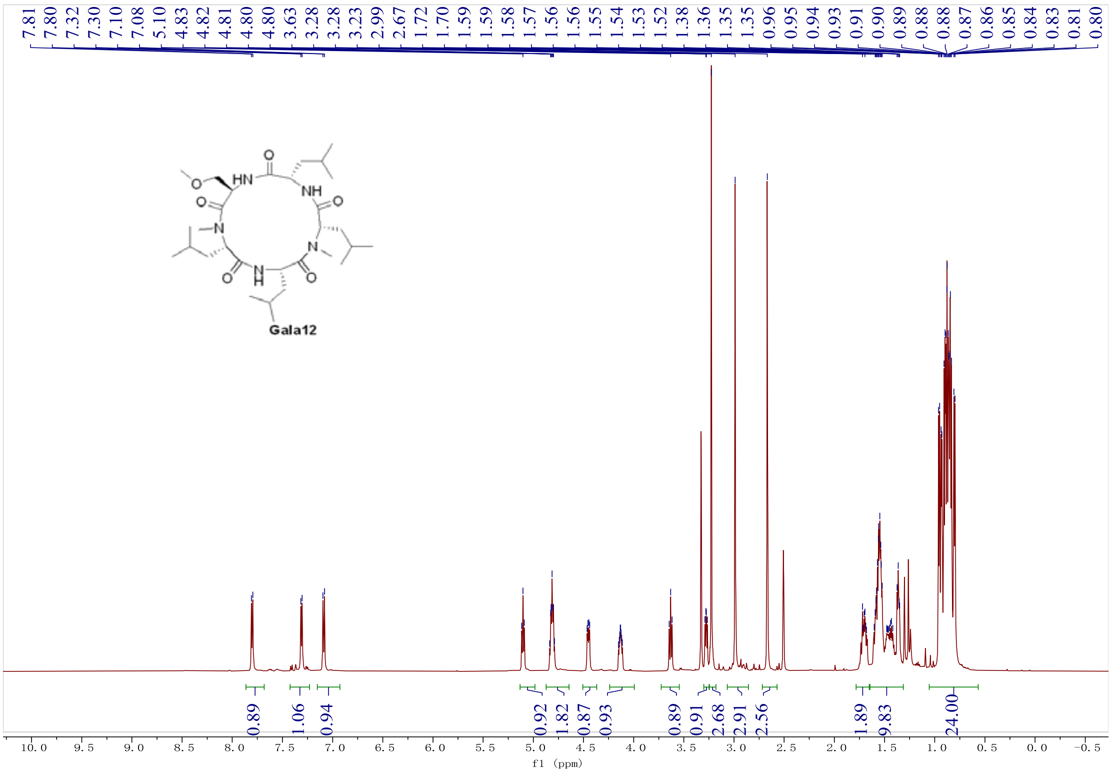

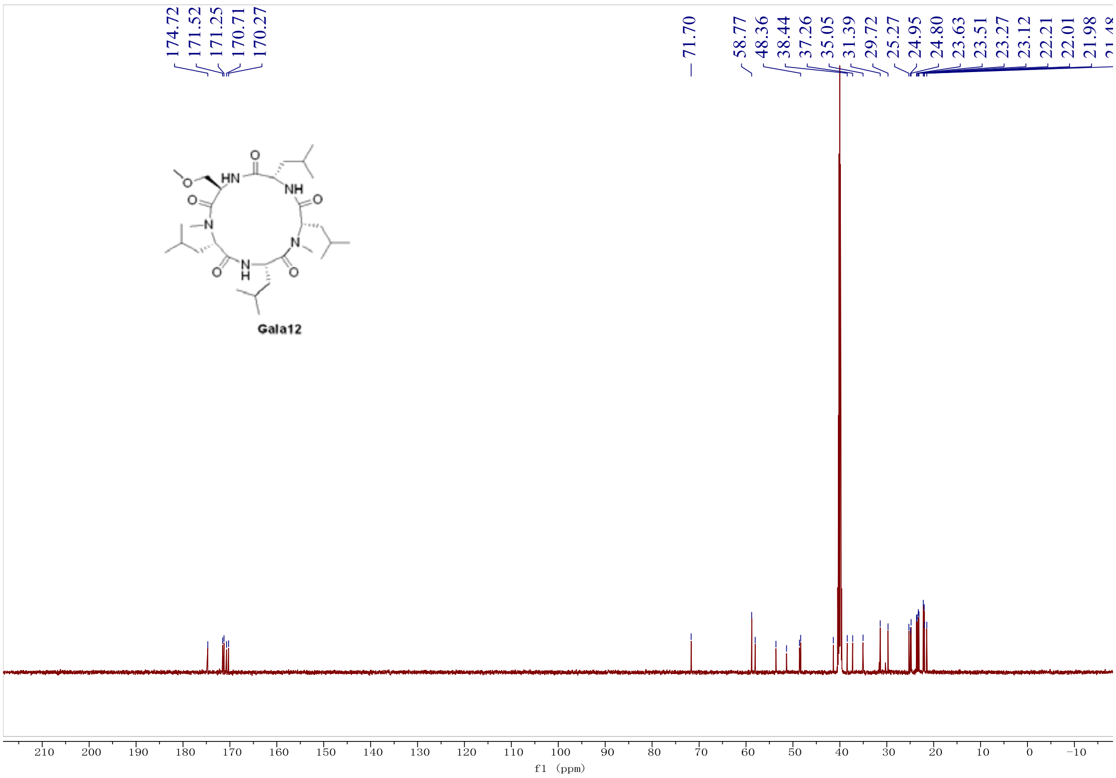

^{1}H NMR (DMSO, 600 MHz) and ^{13}C NMR (DMSO, 151 MHz) for **Gala13**

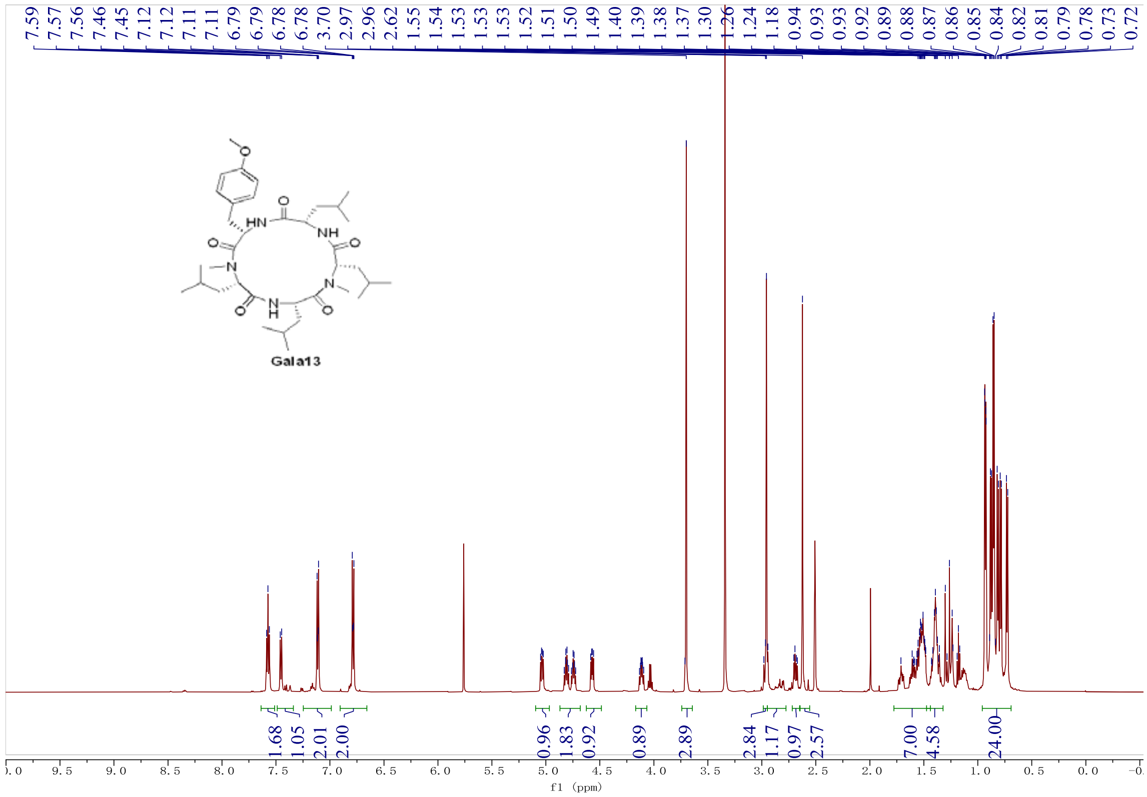

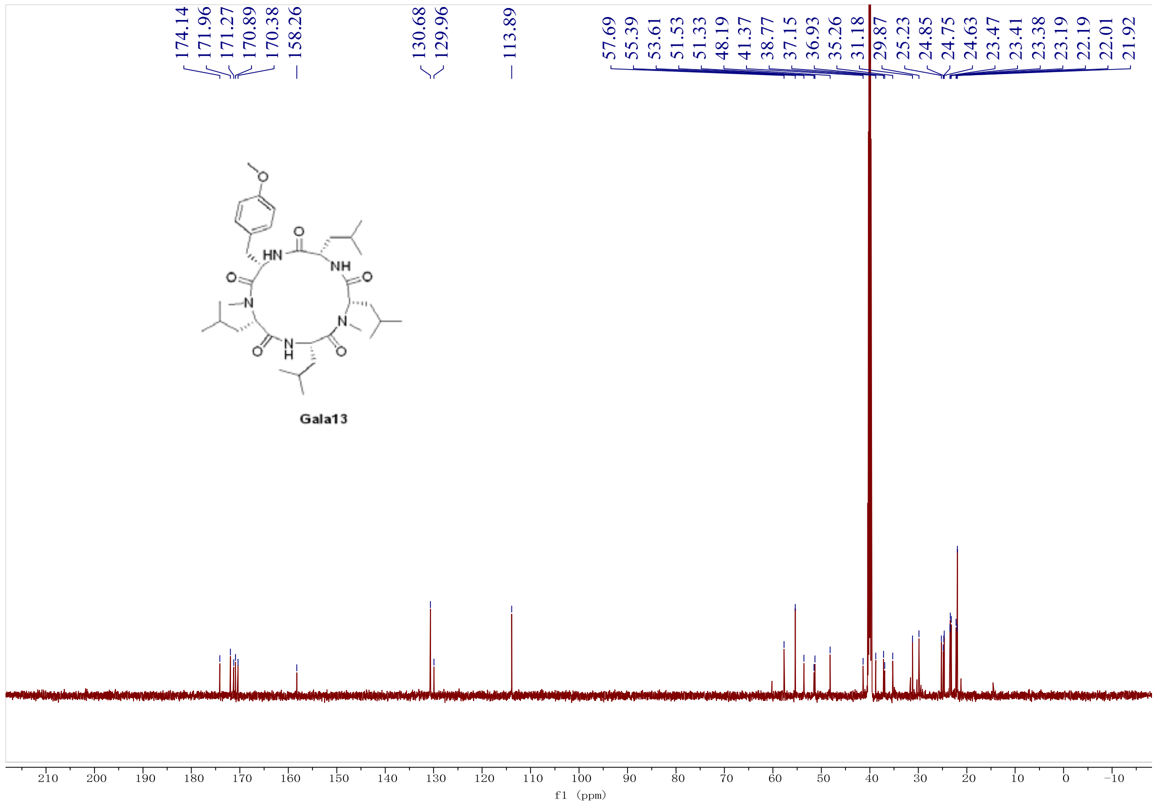

^{1}H NMR (DMSO, 600 MHz) and ^{13}C NMR (DMSO, 151 MHz) for **Gala14**

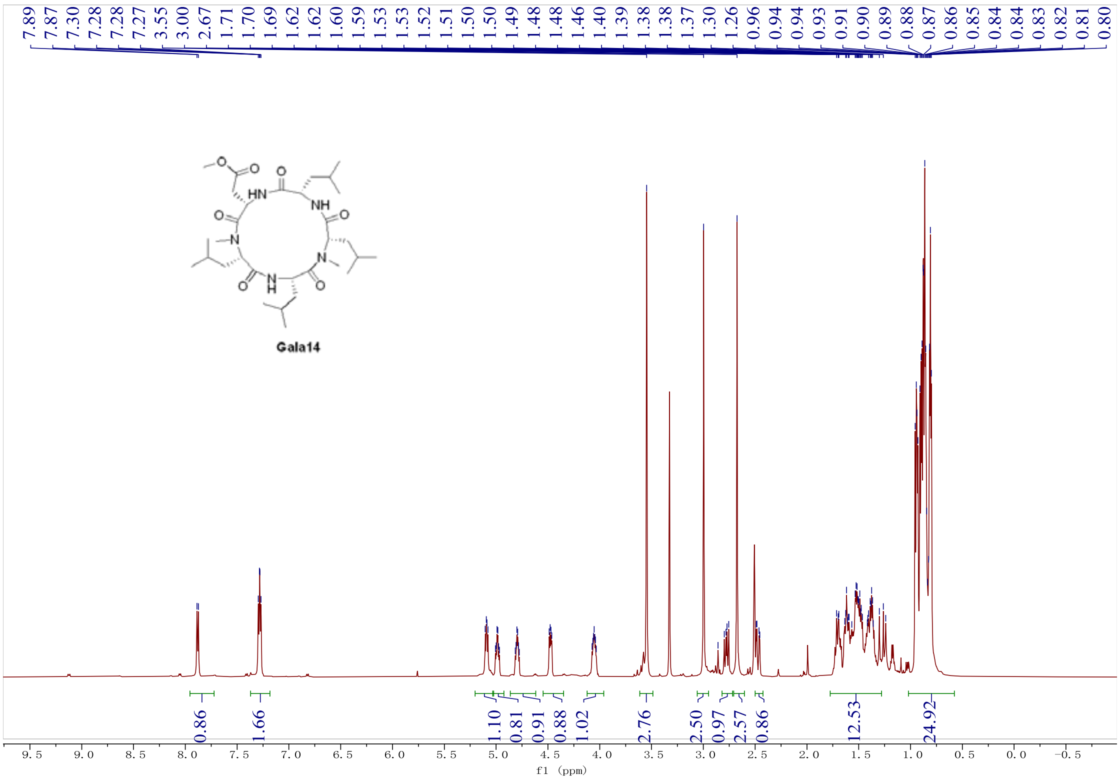

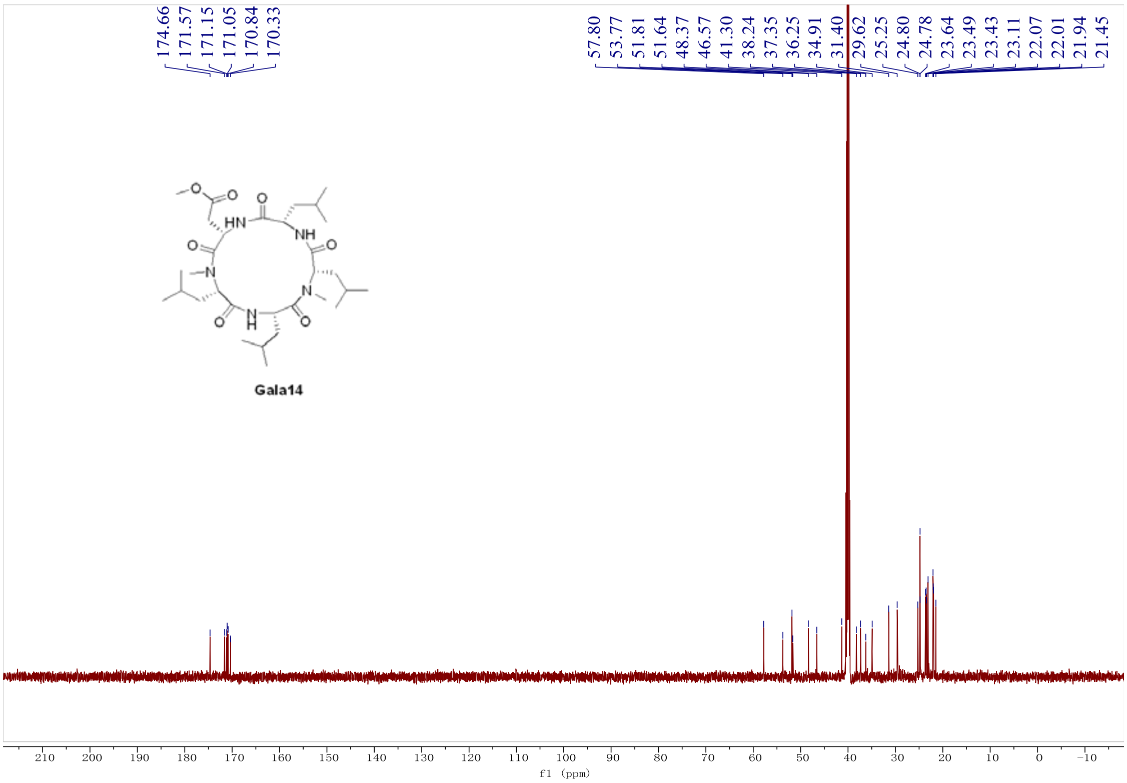

^{1}H NMR (DMSO, 600 MHz) and ^{13}C NMR (DMSO, 151 MHz) for **Gala15**

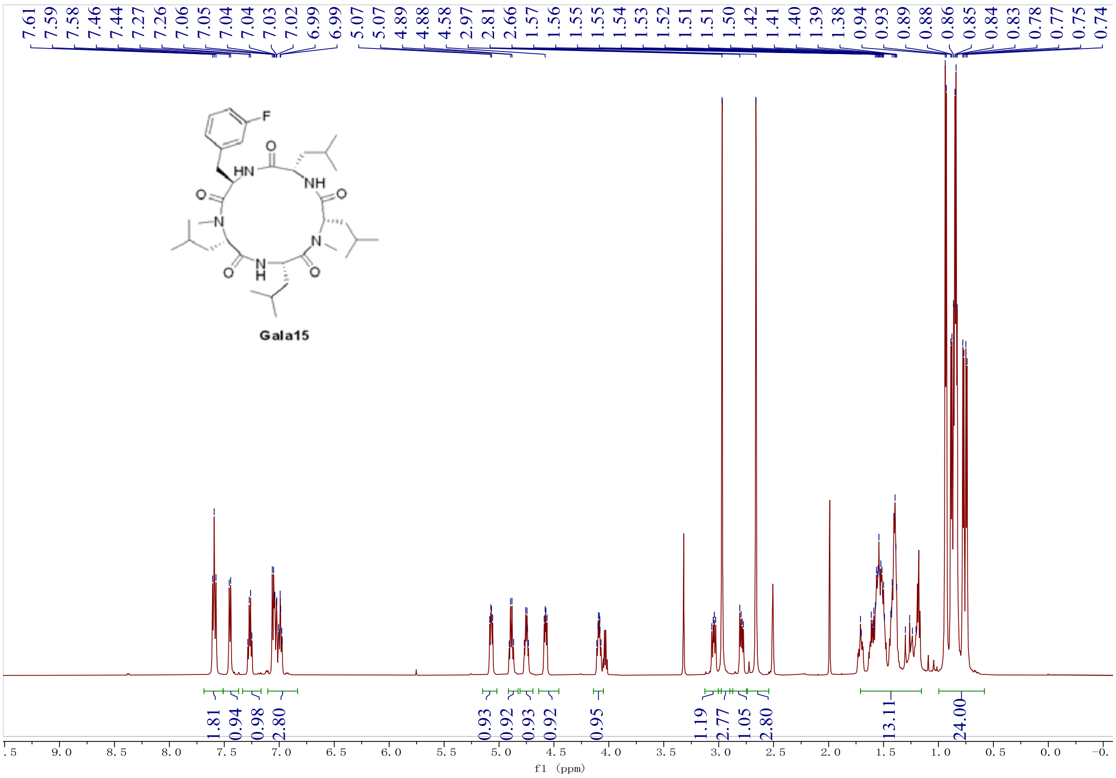

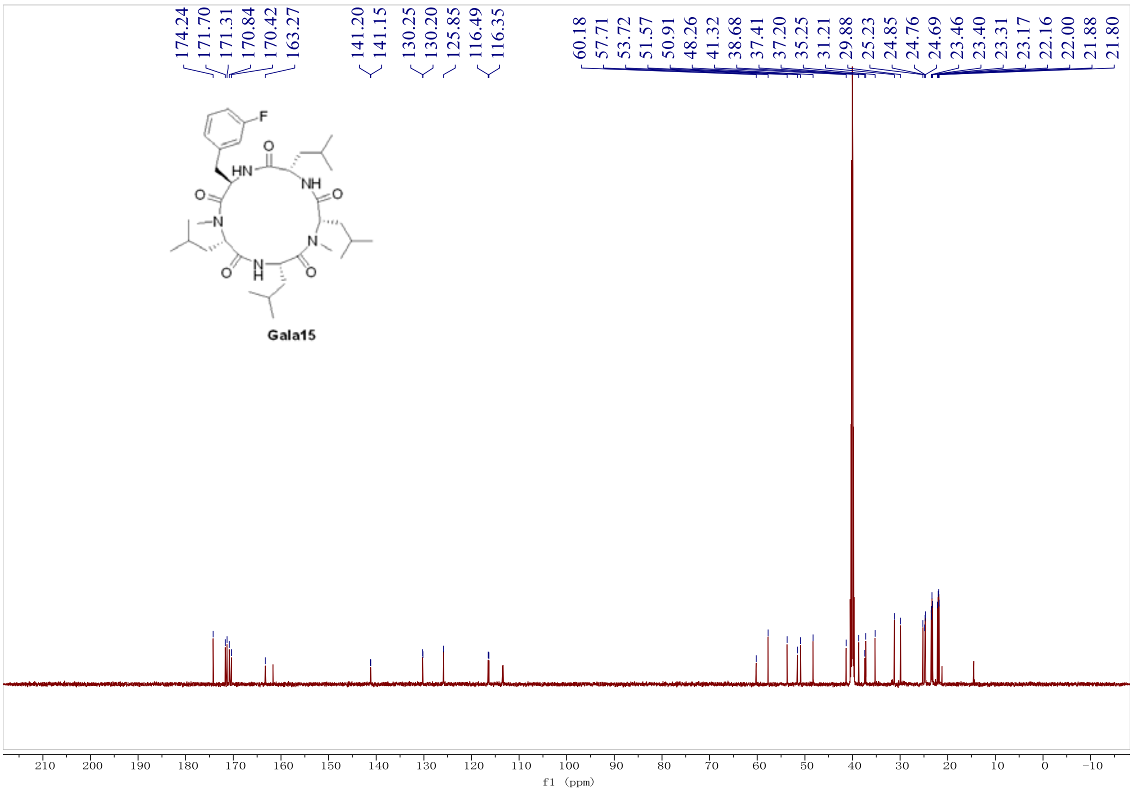

^{1}H NMR (DMSO, 600 MHz) and ^{13}C NMR (DMSO, 151 MHz) for **Gala16**

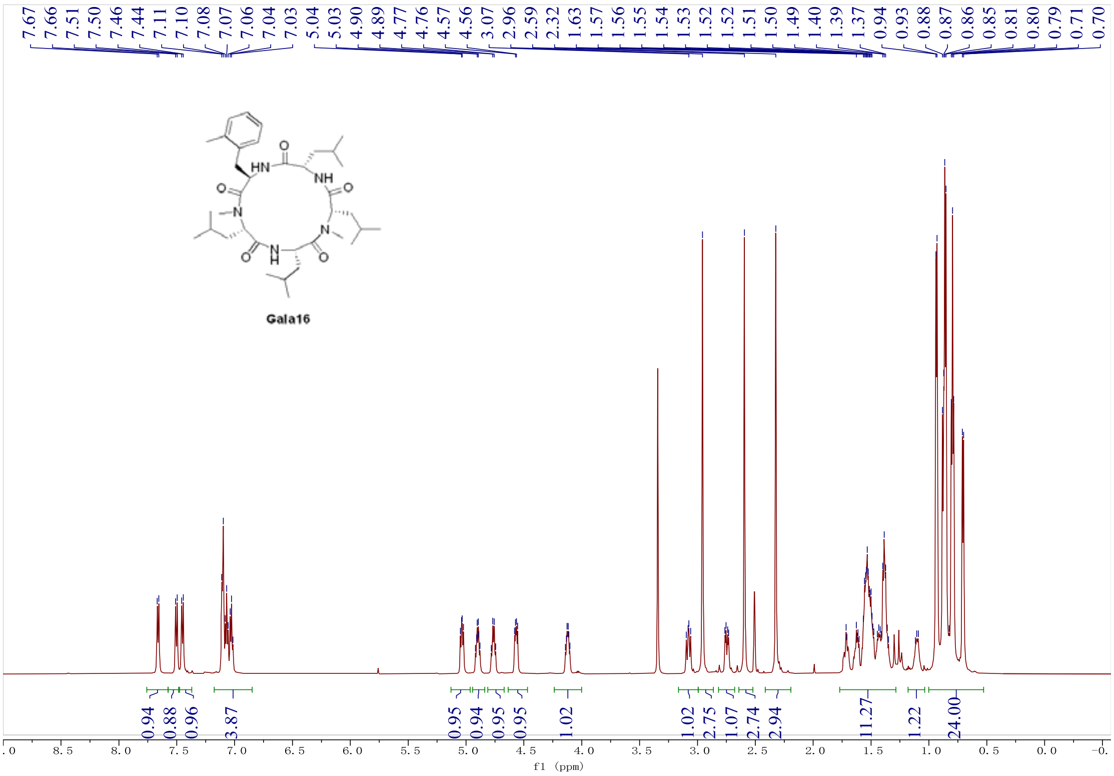

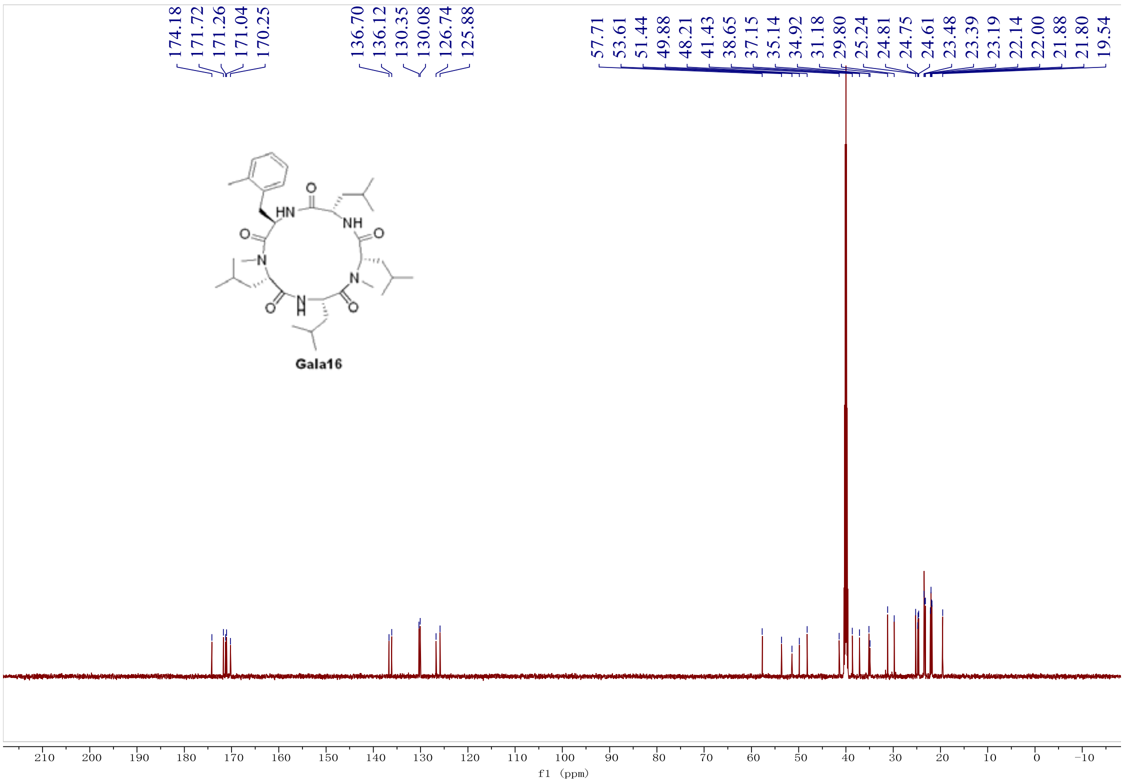

^{1}H NMR (DMSO, 600 MHz) and ^{13}C NMR (DMSO, 151 MHz) for **Gala17**

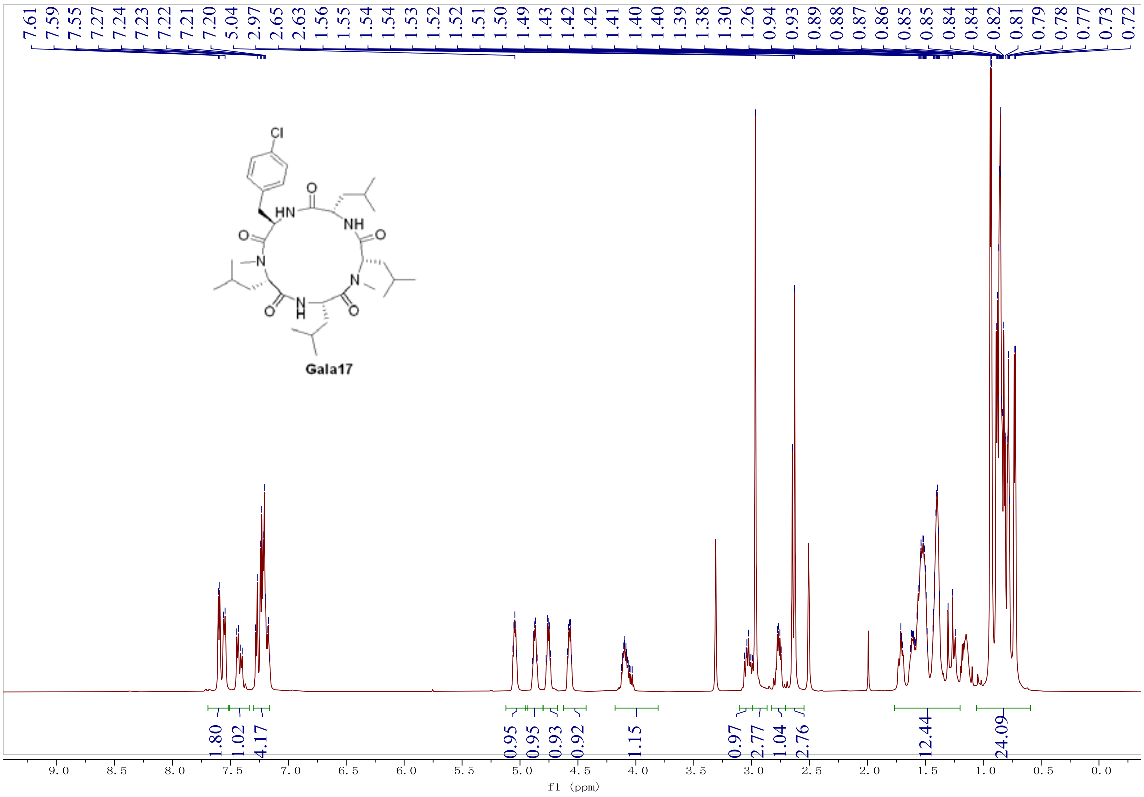

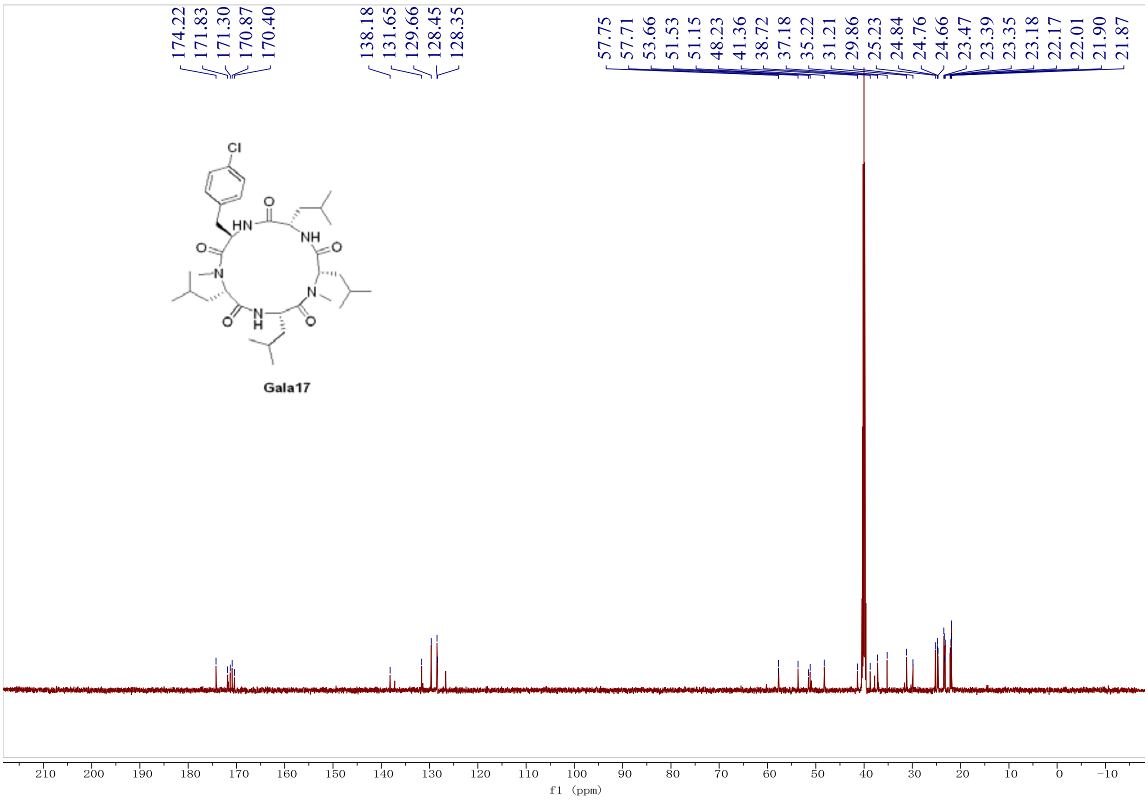

^{1}H NMR (DMSO, 600 MHz) and ^{13}C NMR (DMSO, 151 MHz) for **Gala18**

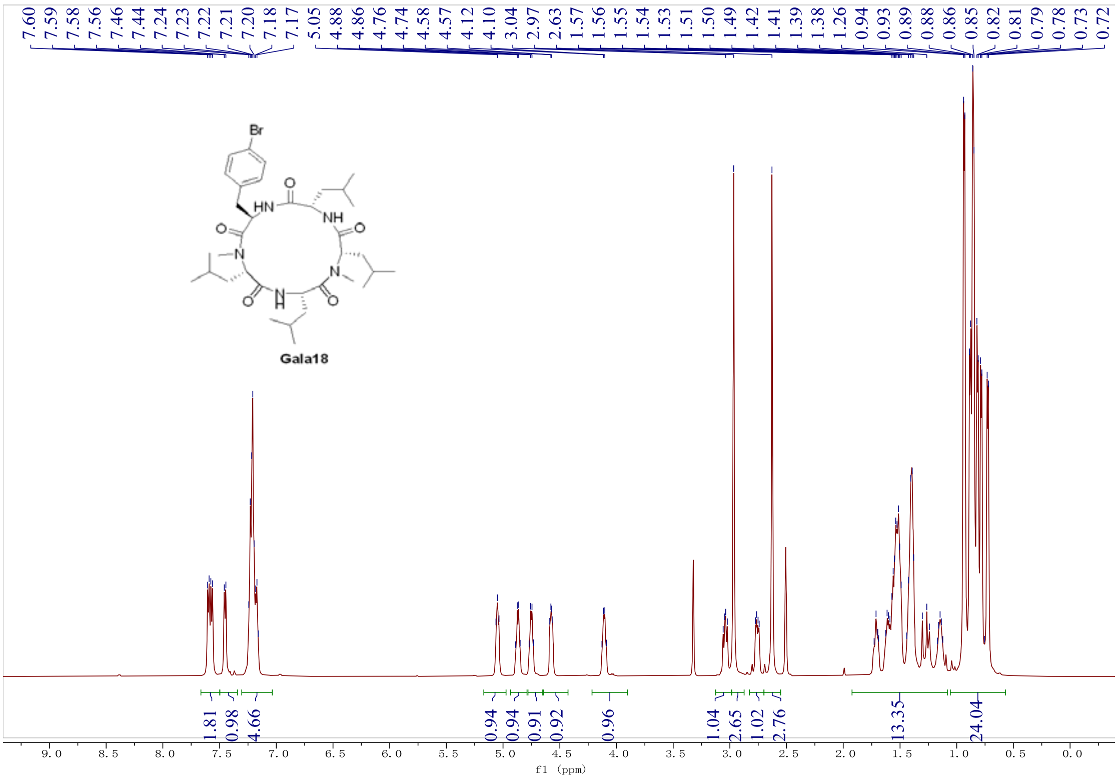

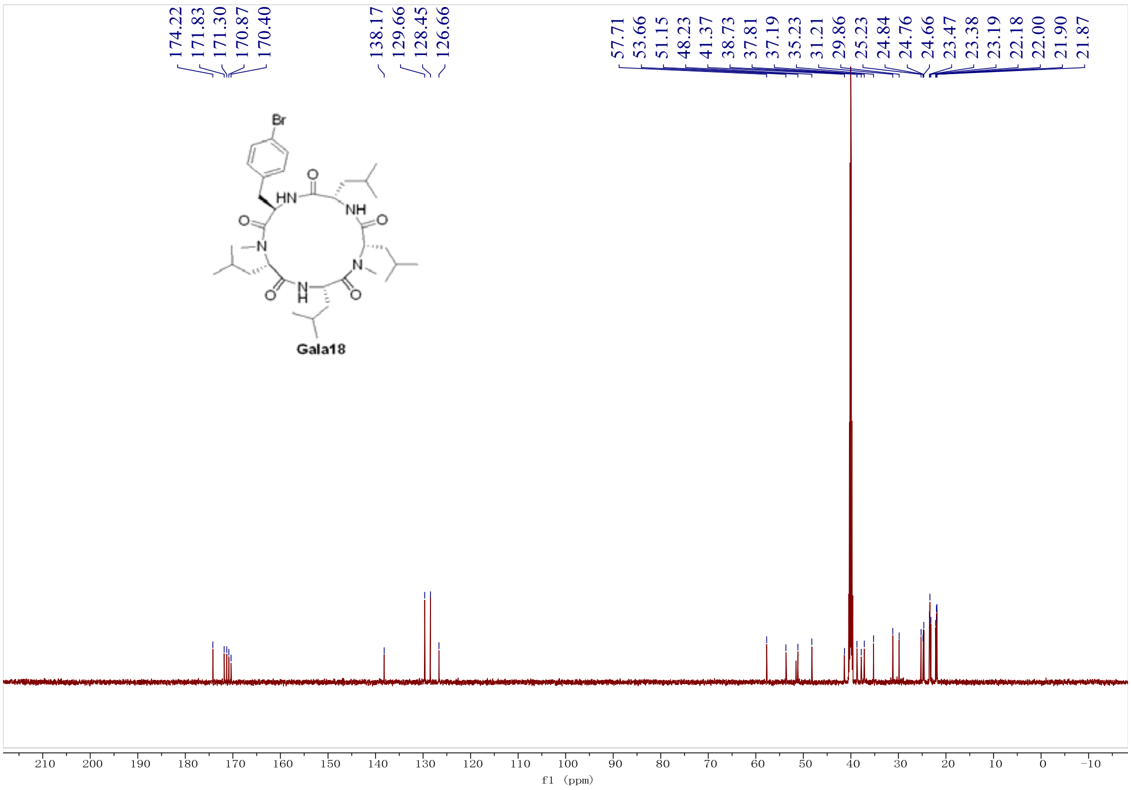

^{1}H NMR (DMSO, 600 MHz) and ^{13}C NMR (DMSO, 151 MHz) for **Gala19**

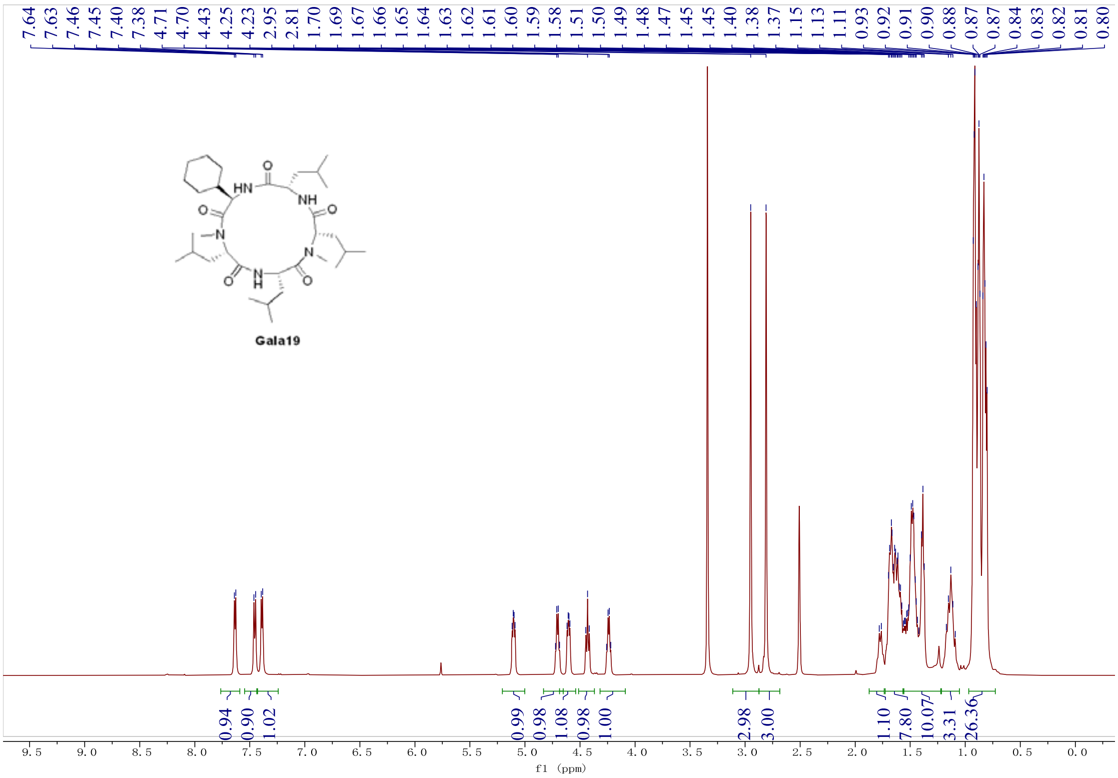

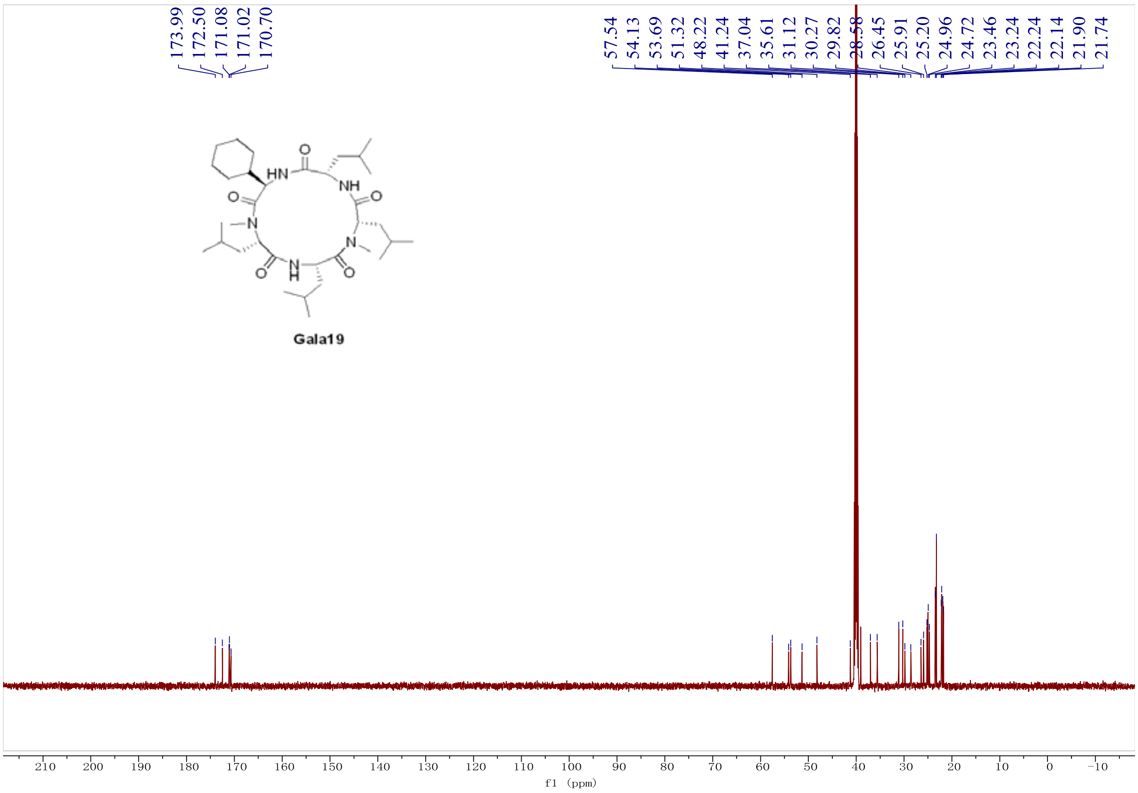

^{1}H NMR (DMSO, 600 MHz) and ^{13}C NMR (DMSO, 151 MHz) for **Gala20**

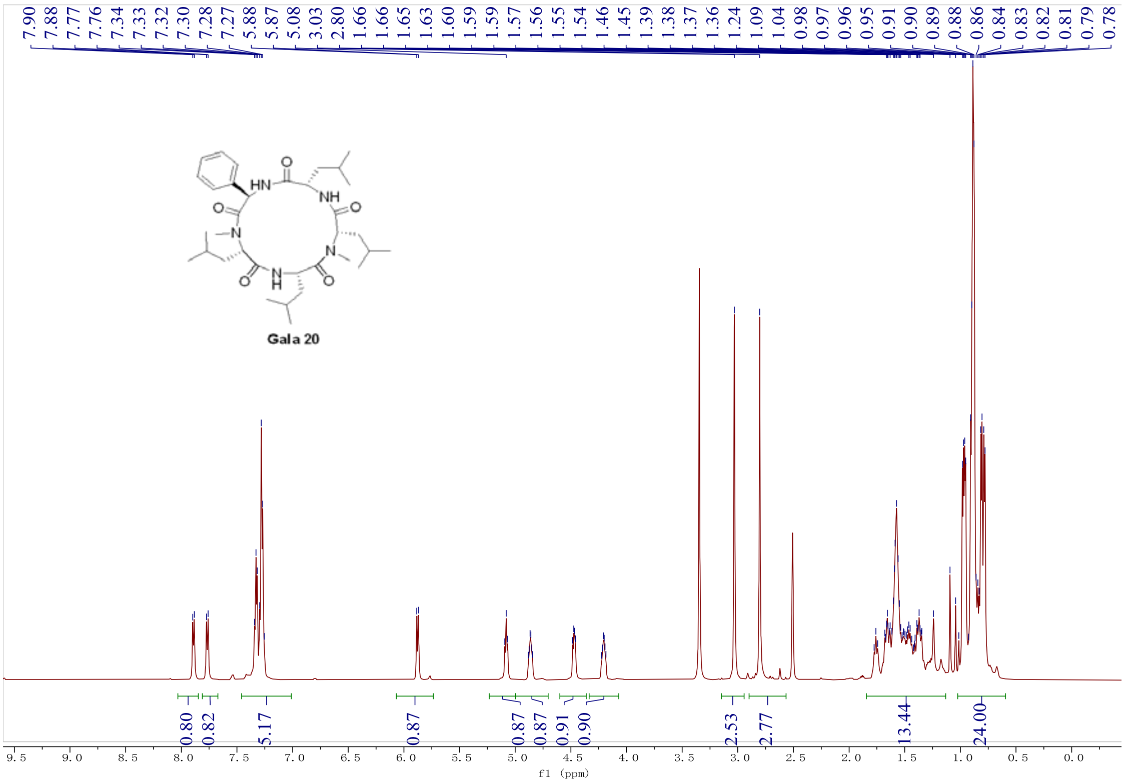

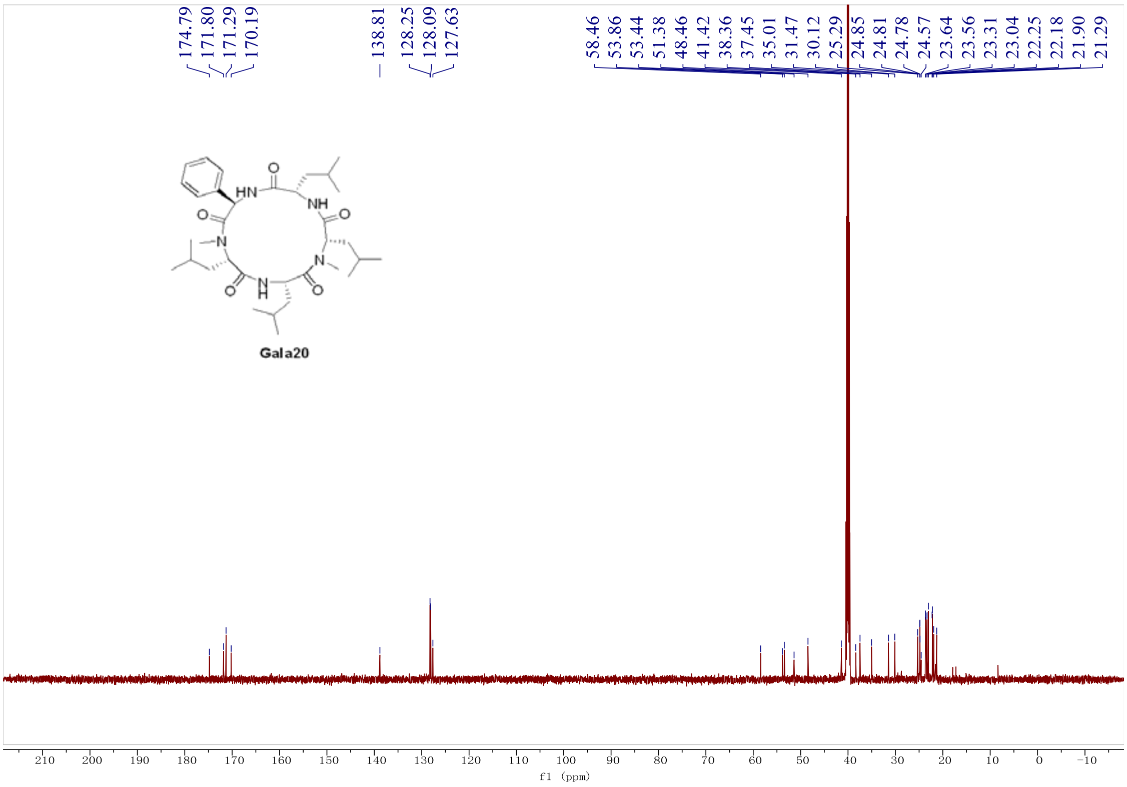

^{1}H NMR (DMSO, 600 MHz) and ^{13}C NMR (DMSO, 151 MHz) for **Gala21**

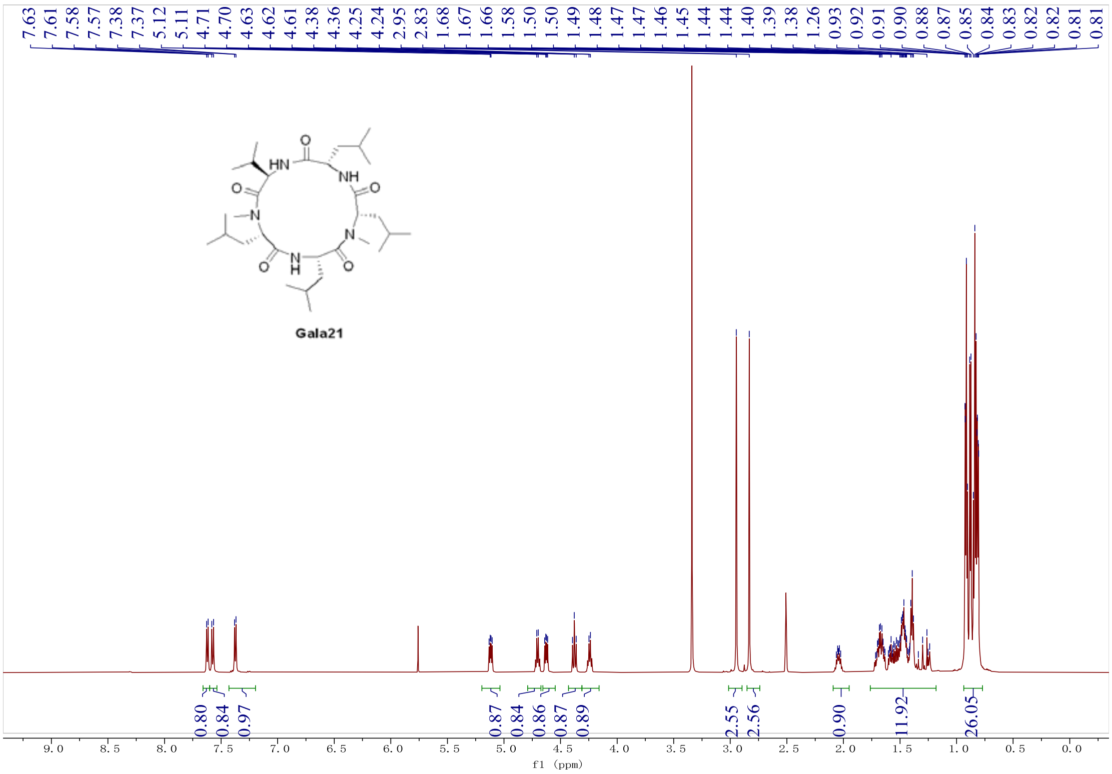

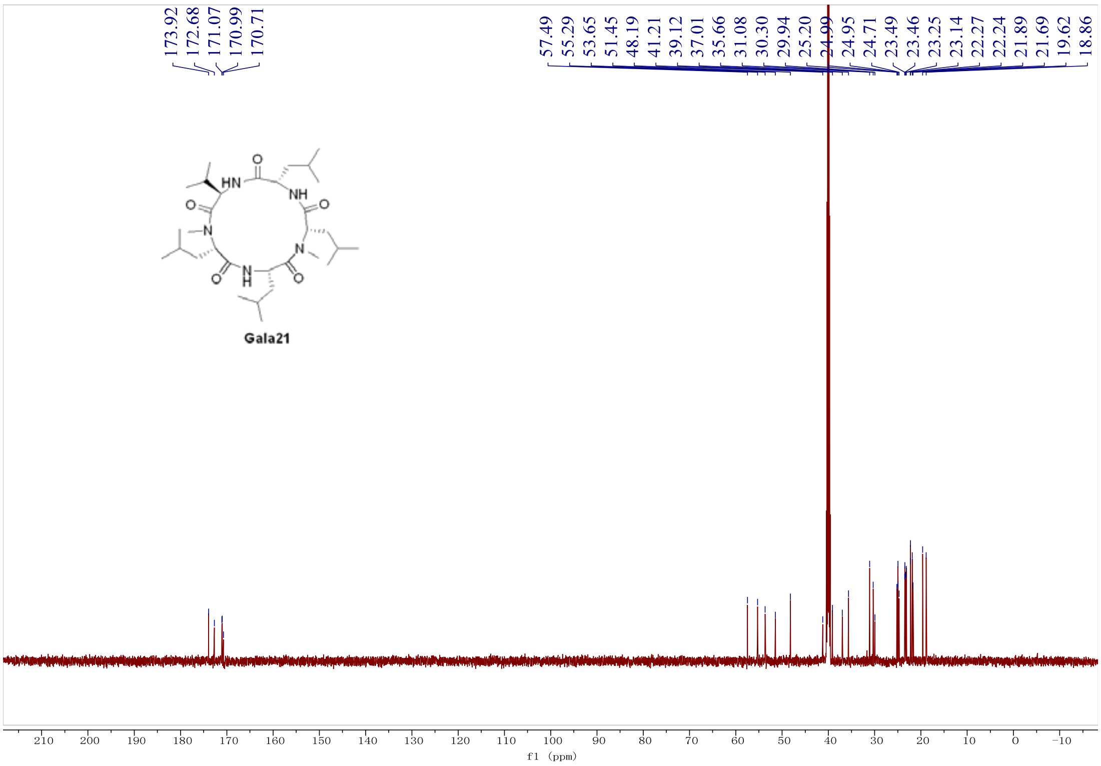

^{1}H NMR (DMSO, 600 MHz) and ^{13}C NMR (DMSO, 151 MHz) for **Gala22**

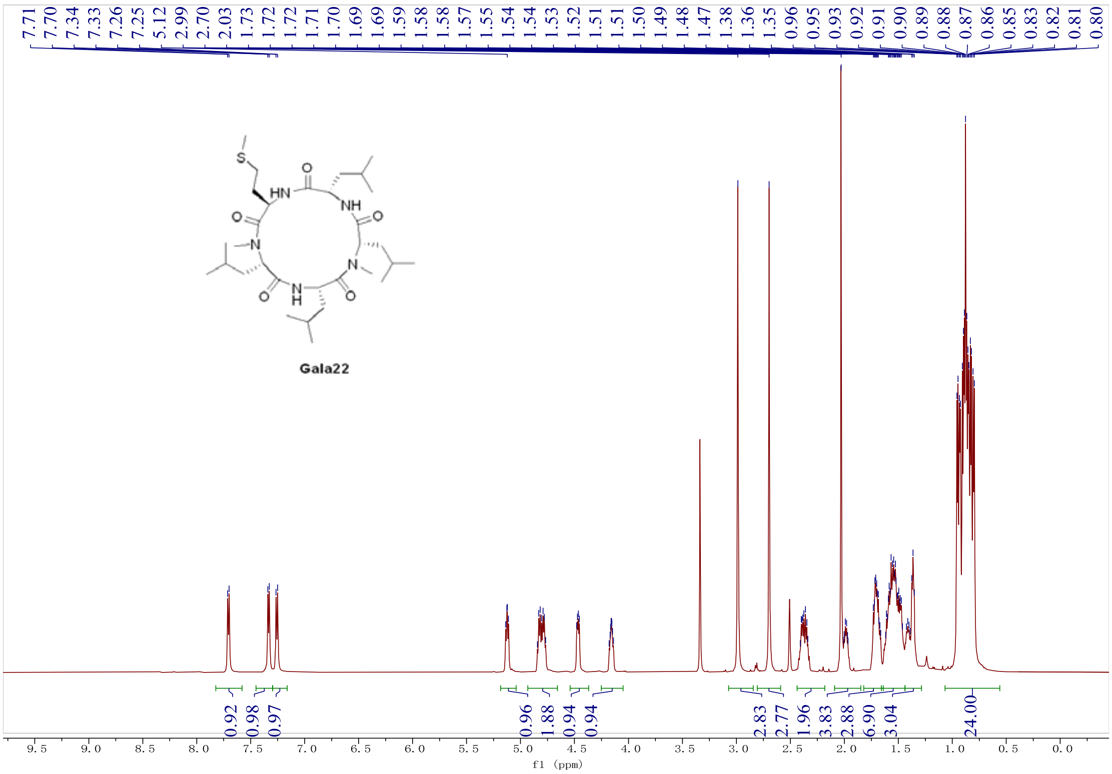

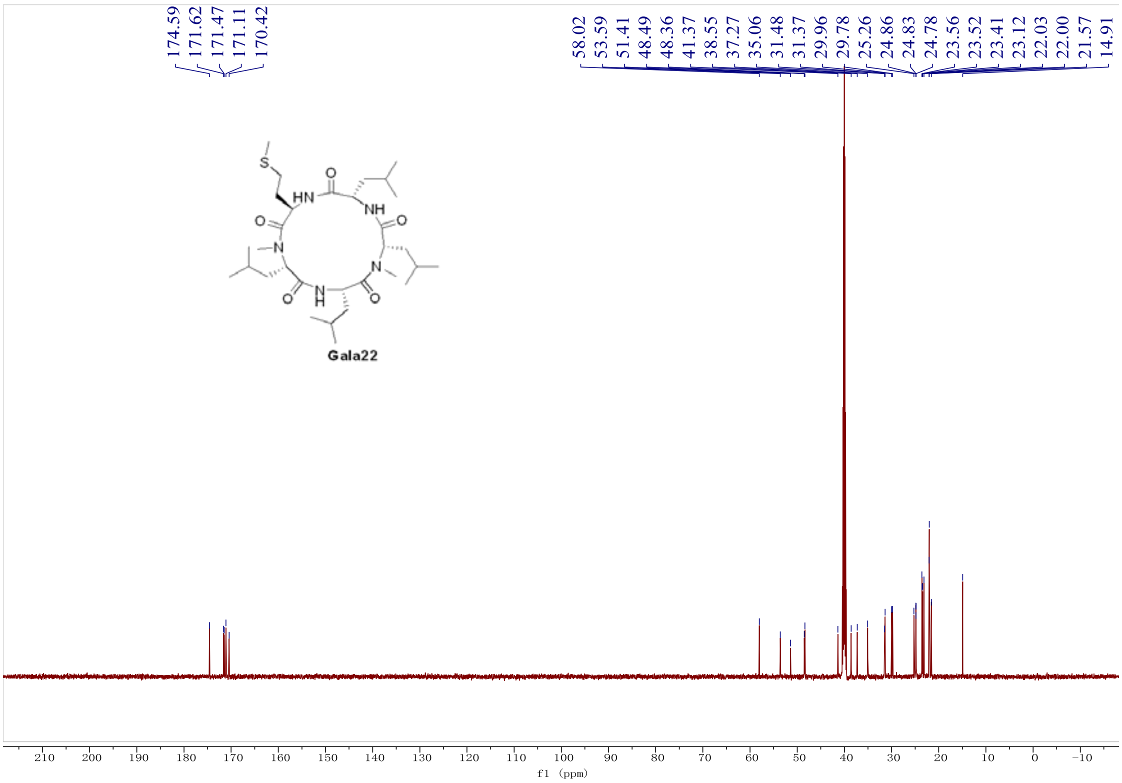

^{1}H NMR (DMSO, 600 MHz) and ^{13}C NMR (DMSO, 151 MHz) for **Gala23**

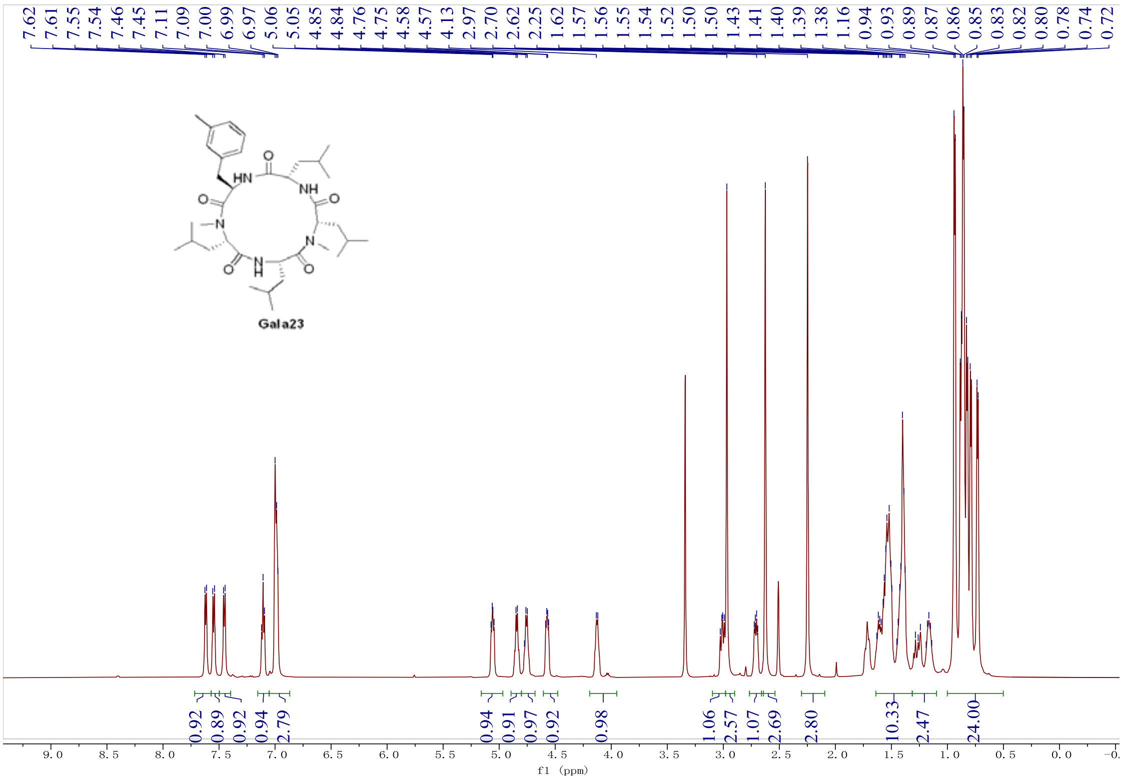

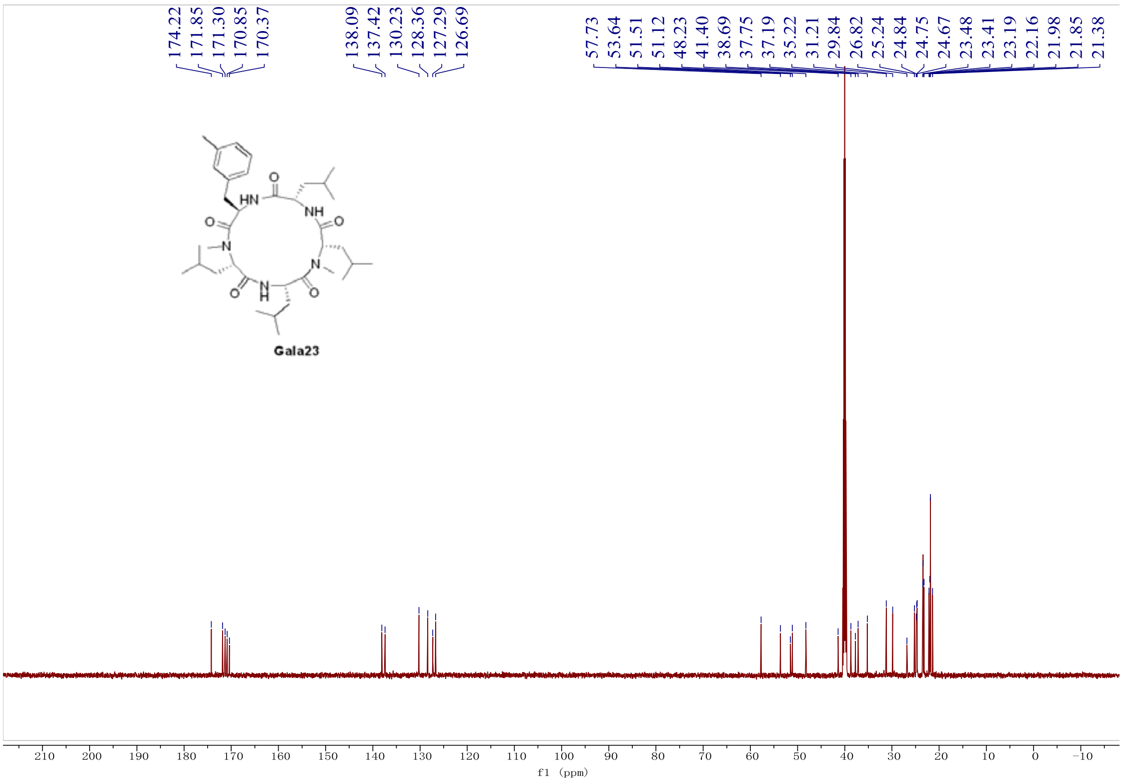

^{1}H NMR (DMSO, 600 MHz) and ^{13}C NMR (DMSO, 151 MHz) for **Gala24**

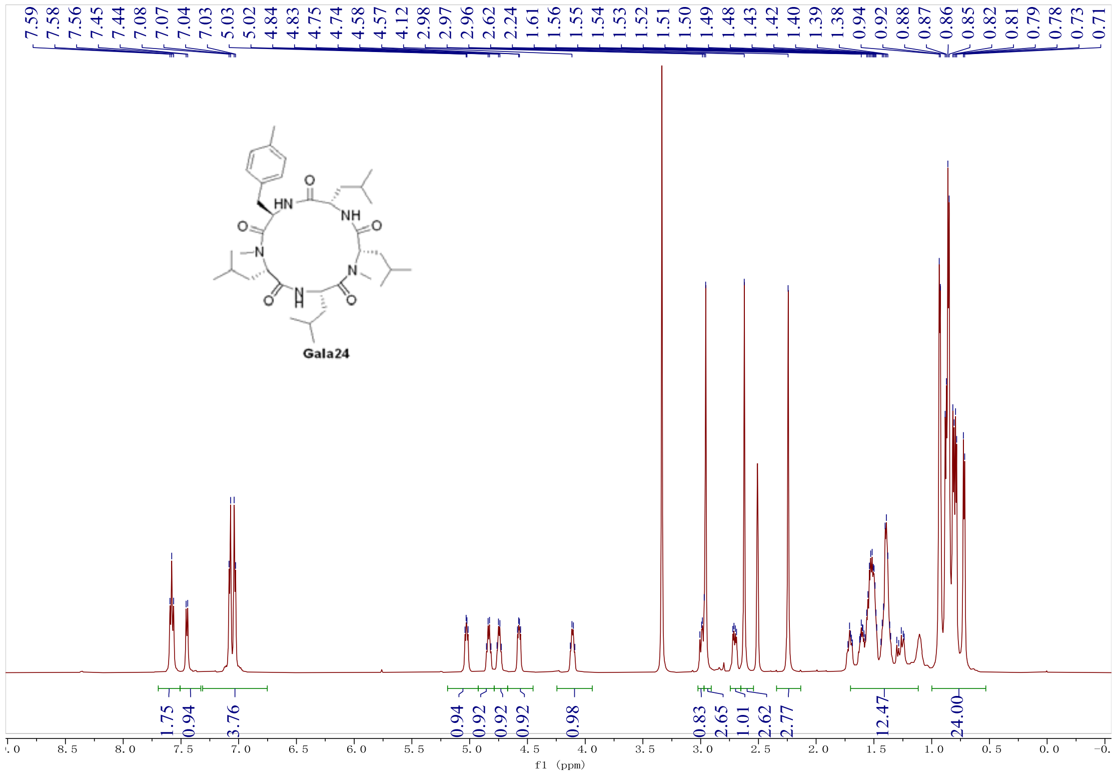

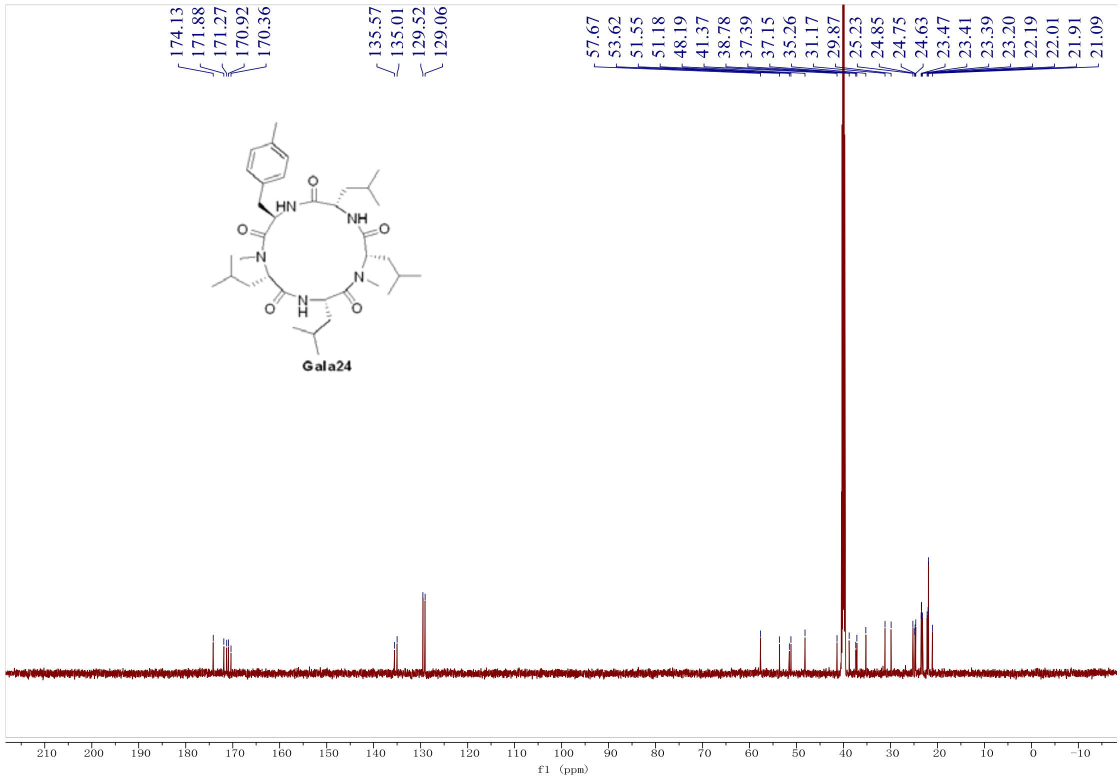

Supplement: Supplementary file 1 [file molecules-30-02362-s001.zip › molecules-3638066-supplementary.pdf]
